# Phenotypic characteristics and variability in CHARGE syndrome: a PRISMA compliant systematic review and meta-analysis

**DOI:** 10.1186/s11689-022-09459-5

**Published:** 2022-08-31

**Authors:** Andrea T. Thomas, Jane Waite, Caitlin A. Williams, Jeremy Kirk, Chris Oliver, Caroline Richards

**Affiliations:** 1 School of Psychology, University of Birmingham, Edgbaston, Birmingham, UK; 2 Cerebra Network for Neurodevelopmental Disorders, Birmingham, UK; 3 Aston University, Birmingham, UK; 4 Centre for Educational Development, Appraisal and Research (CEDAR), University of Warwick, Coventry, UK; 5 Birmingham Women’s and Children’s NHS Foundation Trust, Birmingham, UK

**Keywords:** CHARGE syndrome, Sensory impairment, Behavioural phenotype, Aggressive behaviour, Self-injurious behaviour, Sleep, Intellectual disability

## Abstract

**Background:**

CHARGE syndrome (OMIM #214800) is a phenotypically complex genetic condition characterised by multi-system, multi-sensory impairments. Behavioural, psychological, cognitive and sleep difficulties are not well delineated and are likely associated with biopsychosocial factors.

**Methods:**

This meta-analysis investigated the prevalence of clinical features, physical characteristics and conditions, behavioural, psychological, cognitive and sleep characteristics in CHARGE syndrome, and statistically evaluated directional associations between these characteristics. Pooled prevalence estimates were calculated using reliable, prespecified quality weighting criteria, and meta-regression was conducted to identify associations between characteristics.

**Results:**

Of the 42 eligible studies, data could be extracted for 1675 participants. Prevalence estimates were highest for developmental delay (84%), intellectual disability (64%), aggressive behaviour (48%), self-injurious behaviour (44%) and sleep difficulties (45%). Meta-regression indicated significant associations between intellectual disability and choanal atresia, intellectual disability and inner ear anomalies, sleep difficulties and growth deficiency, and sleep difficulties and gross motor difficulties.

**Conclusions:**

Our comprehensive review of clinical features, behavioural, psychological, cognitive and physical characteristics, conditions and comorbidities in CHARGE syndrome provides an empirically based foundation to further research and practice.

**Supplementary Information:**

The online version contains supplementary material available at 10.1186/s11689-022-09459-5.

## Background

CHARGE syndrome (CS) is a highly variable multisystemic condition, with an estimated prevalence of 1 in 8500 live births [1]. The acronym refers to the prominent congenital malformations first used to delineate the syndrome: Coloboma, Heart defects, Atresia choanae, Retardation of growth and development, Genital abnormalities and Ear anomalies [2] (see Table 1).

**Table 1:** Diagnostic criteria for CHARGE syndrome

	Pagon et al. [2]	Blake et al. [3]	Verloes [4]	Hale et al. [5]
**Major criteria**	Choanal atresia Ocular coloboma	Ocular coloboma Choanal atresia or stenosis Cranial nerve anomalies Characteristic ears anomalies	Ocular Coloboma Choanal atresia Hypoplasia of the semicircular canals	Coloboma Choanal atresia or cleft pallet Abnormal external, middle, or inner ears Pathogenic CHD7 variant
**Minor criteria**	Heart defect Retardation^a^ (of growth or development) Genital anomalies Ear anomalies	Characteristic CHARGE facies Cardiovascular malformations Tracheoesophageal fistula Growth deficiency Genital hypoplasia Cleft lip or palate Developmental delay	Rhombencephalic dysfunction Hypothalamo-hypophyseal dysfunction Abnormal external or internal ear Malformation of mediastinal organs Mental retardation^a^	Cranial nerve dysfunction (including hearing loss) Dysphagia or feeding difficulties Structural brain anomalies Developmental delay, intellectual disability, or autism Hypothalamo-hypophyseal dysfunction, genital anomalies Heart or oesophageal malformation Renal anomalies skeletal or limb anomalies
**Occasional findings**		Renal anomalies Spinal anomalies Hand anomalies Neck/shoulder anomalies		
**Inclusion rule**	Four criteria, including one major criteria	Four major criteria or three major and three minor criteria	Typical CHARGE: Three major or two major and two minor criteria Partial CHARGE: Two major and one minor criteria Atypical CHARGE: Two major but no minor or one major and two minor criteria	Two major and any minor criteria

^a^“Mental retardation” is an archaism superseded by DSM-5 intellectual disability/intellectual developmental disorder or ICD-11 disorders of intellectual development

Heterozygous variants in the chromodomain helicase DNA binding protein 7 (CHD7) cause CS [6]. Mechanistically, CHD7 is essential for the differentiation of gene expression at thousands of sites in the human genome [7]. The prevailing hypothesis is that the dynamic role of CHD7 during gene expression and neural crest development may account for the pleiotropic signs and symptoms of CS [7]. Prospective investigation of genotype-phenotype correlations has been performed [8–10] with an association between truncating CHD7 variants and more severe heart defects being identified [10]. However, given the rarity of CS and the spectrum of clinical findings, better delineation of genotype-phenotype associations requires pooling of data across data sets [11].

CS is associated with many disparate physical conditions requiring health monitoring throughout life [12]. Trider et al. [12] developed a comprehensive checklist for proactive monitoring of common or critical physical conditions and characteristics. These conditions will likely have a deleterious impact on emotional and psychological wellbeing. Identifying and understanding these impacts can help build resilience and early support strategies utilising multidisciplinary practices.

While physical health in CS has been extensively documented [12] research on development and behaviour is sparce. Developmental delay (DD) and intellectual disability (ID) have received the most attention and feature in all diagnostic algorithms (Table 1). Direct cognitive assessments are rarely appropriate as performance requires adequate communication and minimal sensory impairment [13]. Consequently, ID is often based on informant measures of adaptive behaviour that might not correlate well with cognitive performance [14–16].

Moreover, sleep problems, anxiety, emotional dysregulation, aggression, self-injurious behaviour and tactile defensiveness are reported in adolescents and adults with CS [17]. Psychiatric diagnoses in children and adults include anxiety, obsessive-compulsive disorder, attention deficit disorder, and autism [17–19]. Data reporting cognitive, behavioural, and psychiatric features in CS warrant synthesis to definitively describe the behavioural phenotype in the condition.

Diagnostic criteria have been revised several times to accommodate new insights (e.g. [3–5] see Table 1). Before the identification of the molecular etiology of CS in 2004, individuals were diagnosed solely based on clinical characteristics. Around 90% of individuals that meet clinical criteria for CS have an identifiable CHD7 variant [7]. However, there remains substantial heterogeneity in phenotypic presentation and CHD7 variants. A meta-analytic strategy would be informative to generate pooled prevalence estimates for clinical features based on a diagnosis of CHARGE syndrome (see Table 1). This would enable quantification of phenotypic characteristics and variability between individuals and further evaluation of moderating and co-occurring characteristics to assist in the exploration of potential subgroups within the clinically diagnosed CHARGE syndrome phenotype.

In this study, we apply meta-analytic techniques to synthesise prevalence estimates across published studies. Given the challenges of assessing behavioural, cognitive and sleep characteristics in CS and potential for varying methodological quality, studies are quality weighted prior to meta-analysis. Pooled prevalence estimates facilitate subgroup meta-regression analyses to elucidate and quantify interrelated characteristics. The aims of this study are the following:

1. To provide pooled prevalence estimates for clinical features for CS.
2. To calculate pooled prevalence data for any physical characteristics and conditions frequently reported in the literature as associated with a diagnosis of CS.
3. To provide quality adjusted prevalence estimates for behavioural, psychological, cognitive and sleep characteristics in CS.
4. To conduct an exploratory meta-regression to systematically evaluate:
  - The extent to which clinical features and physical characteristics and conditions can explain variability between cohort studies reporting on developmental delay, behavioural, psychological, cognitive and sleep characteristics in CS.
  - The extent to which developmental delay, behavioural psychological, cognitive and sleep characteristics co-occur in CS.
5. To systematically quantify and explore genotype-phenotype associations for evidence of potential subgroups within the clinically diagnosed CHARGE syndrome phenotype as follows:
  - To calculate the pooled prevalence of a CHD7 positive status in CS
  - To calculate pooled prevalence estimates for truncating and non-truncating mutations within individuals with CS that have a CHD7 positive status.
  - To explore the extent to which developmental, psychological, cognitive and sleep characteristics can explain the variability between individuals with or without an identified CHD7 variant, or between truncating and non-truncating CHD7 mutations.

## Methods

### Comprehensive search strategy

The reporting of this systematic review was guided by the standards of the Preferred Reporting Items for Systematic Review and Meta-Analysis (PRISMA) [20] (see Appendix 1 of Supplementary materials 1; S1 [21]. The databases PubMed, Ovid MEDLINE, PsycINFO and Embase were searched from inception until January 12, 2021, using search terms for CS generated from OMIM. Search terms included MeSH terms and “All Fields” advanced searches for: CHARGE syndrome; CHARGE association; coloboma, heart anomaly, choanal atresia, genital anomalies and ear anomalies; Hall Hittner syndrome; CHD7; and SEMA3A. Details of search syntax are available in Appendix 2 (S1 [21]). Manual searches of reference lists from recent review articles [12, 22, 23], gene review knowledge bases (GeneReviews®, UniProtKB) and contents pages of key journals (American Journal of Medical Genetics Part A (1979-2021), B (2003-2021) and C (2003-2021)) were also conducted to facilitate a comprehensive investigation. Details of manual searches are available in Appendix 3 (S1 [21]).

### Selection criteria

Study selection was completed by the first author. Inclusion criteria permitted any peer-reviewed study reporting on the prevalence of behavioural, psychological, cognitive or sleep characteristics in a sample of participants with a clinical diagnosis of CS. Studies with less than five participants and case-series reports were excluded (details are available in Appendix 4 (S1 [21]).

### Data extraction

The first author independently extracted all data. Participant-level data were extracted for year of publication, recruitment of sample and sample size, participant age and gender, clinical features, CHD7 status and classification of CHD7 variant, enduring or recurrent physical characteristics and conditions, and behavioural, psychological, cognitive and sleep characteristics.

### Quality appraisal

The quality framework used (see Table 2) was adapted from Richards et al. [24] and Surtees et al. [25] to control for the risk of methodological bias between individual studies included in the meta-analysis. Good inter-rater reliability was obtained for the quality framework, using a 25% random sample of the eligible studies. Details are available in Appendix 5 (S1 [21]). In summary, scores ranging from 1 (poor) to 4 (excellent) were awarded based on sample identification, confirmation of syndrome and assessment of behaviour, cognition or sleep.

**Table 2:** Quality framework

	***1-Poor***	***2-Adequate***	***3-Good***	***4-Excellent***
**Sample identification**	Not specified/reported	Single, restricted or non-random sample (e.g. from a specialist clinic or previous research study)	Multiple restricted or non-random sample (e.g. multiregional specialist clinics)	Random or total population sample
**Confirmation of syndrome**	Not reported or reported without reference to the criteria used (e.g. [2–5])	Specific clinical criteria reported (e.g. [2–5]) without genetic assessment	Specific clinical criteria reported (e.g. [2–5]) with CHD7 status reported retrospectively	Specific clinical criteria reported (e.g. [2–5]) alongside prospective genetic assessment
**Behavioural/psychological assessment**	Descriptions of behaviour based on non-standardised informant report or review of clinical information	Standardised informant report measure (e.g. the RBQ^a^ ADI-R^b^) or clinical judgement based on DSM^c^ or ICD^d^ criteria	Standardised behavioural or observational assessment (e.g., neuropsychiatric evaluation, ADOS^e^	Consensus drawn from multiple assessments, including one or more standardised behavioural or observational assessment
**Cognitive assessment**	Description or estimation of cognitive ability based on non-standardised informant report or review of clinical information	Standardised informant report measure (e.g. VABS)^f^	Standardised behavioural or observational assessment (e.g. neuropsychiatric evaluation, BSID)^g^	Consensus drawn from multiple assessments, including one or more standardised behavioural or observational assessment
**Sleep assessment**	Response to a single question or response to a non-standardised questionnaire	Standardised sleep questionnaire (e.g. SDSC)^h^	Informant report diary or direct clinical observation (e.g. video footage or observation made within a clinical or hospital setting, not polysomnography or actigraphy)	Use of polysomnography or actigraphy

Quality criteria must apply to 90%

^a^Repetitive Behaviour Questionnaire [26]

^b^Autism Diagnostic Interview–Revised [27]

^c^Diagnostic and Statistical Manual of Mental Disorders

^d^International Classification of Diseases

^e^Autism Diagnostic Observation Schedule [28]

^f^Vineland Adaptive Behavior Scales [29]

^g^Bayley Scales of Infant Development [30]

^h^Sleep Disturbances Scale for Children [31]

### Data synthesis

The effect size index for meta-analysis was derived from raw proportions and corresponding standard errors.

The raw proportion (PR) is given by

$$\mathrm{PR}=\left(\mathrm{Exp}.\mathrm{case}\right)/\left(\mathrm{Exp}.\mathrm{sample}\right)$$

where Exp.case is the number of individuals experiencing the characteristic of interest, and Exp.sample is the number of individuals sampled.

The standard error (SE) of the raw proportion is given by:

$$\mathrm{SE}=\surd \left(\left(\mathrm{PR}\times \left(1-\mathrm{PR}\right)\right)/\left(\mathrm{Exp}.\mathrm{sample}\right)\right)$$

Given the anticipated small sample size indicative of rare syndrome research, a pragmatic decision was made to exclude studies with less than five participants or an effect size of zero under the assumption that the sample size would not afford accurate estimation of the true event rate.

Where multiple measures of the same construct were reported across multiple subgroups, data were combined into one quantitative outcome. When computationally appropriate (i.e. where five or more study effects could be synthesised) characteristics were subdivided.

### Meta-analysis

Analyses were conducted in R version 3.62. R code is available in Supplementary material 2 [21]. Pooled prevalence estimates and 95% confidence intervals (CIs) were calculated using the inverse variance method [32] assuming a random-effects model (REM). The assumption is that the synthesised studies vary randomly under a common distribution and the REM estimates the mean of the assumed distribution [32, 33]. Visualisation of Quantile Quantile (QQ) plots were used to estimate the distribution of study effects for each REM. Where study effect sizes followed an approximate normal distribution, the DerSimonian-Laird estimate (DL) [33] was used to calculate between studies variance (tau). Where QQ plots suggested a non-Gaussian distribution, the restricted maximum-likelihood (ReML) estimator was used. ReML avoids over-fitting, providing an efficient estimator of tau when effects are not normally distributed [34].

To address methodological differences quality weightings were used to extend the random-effects model (QEM; quality weighted random effects model). REMs were limited to opportunities where five or more study effects could be synthesised. This threshold is the minimum k studies to allow implementation of exact permutation testing to reach statistical significance (i.e., *p* ≤ .05). The test permutes the effect size outcome and calculates a (two-sided) *p* value which is equal to the proportion of times that the absolute value of the test statistic under the permuted data is as extreme or more extreme than the observed data [35]. Where REMs were not statistically significant (*p* < .05) following permutation testing, pooled prevalence estimates were reported using a fixed effect model (FEM). To prevent bias, no studies were included more than once in a single meta-analysis.

Potential sources of heterogeneity were investigated using the *I* squared (*I*^2^) [36] statistic. Values of the *I*^2^ index of 25%, 50%, and 75% were considered respectively as low, medium and high degrees of heterogeneity. Sensitivity was evaluated using the funnel plot, Baujat plot [37] fail-safe *N* [38] and leave one out procedures, and the impact of varying methodological quality was further investigated through a series of subgroup analyses. Details are provided in Appendix 6 (S1 [21]).

Rainforest plots were used to visualise statistically amalgamated studies. The rainforest plot is a variation of the traditional forest plot proposed by Schild and Voracek [39]. This alternative plot visually emphasises larger studies with short confidence intervals (*CI*s) and small studies with wider *CI*s are less visually dominant. Therefore, the rainforest plot enhances the interpretability of the traditional forest plot.

### Meta-regression

Associations were appraised systematically within and between behavioural, psychological, cognitive and sleep characteristics, and between these features and each clinical feature and physical characteristic and condition included in the meta-analysis. Meta-regressions were also conducted to explore genotype-phenotype correlations (associations between CHD7 positive status and characteristics included in the meta-analysis, and truncating CHD7 mutations and characteristics included in the meta-analysis). Meta-regressions were conducted when ≥ 5 study effects could be analysed. Due to the high number of regressions, the Benjamini-Hochberg adjustment for multiple comparisons was used in the first instance [40] followed by permutation testing for studies with a *p* value of 0.05 or above.

## Results

### Comprehensive literature search

**Fig. 1:**
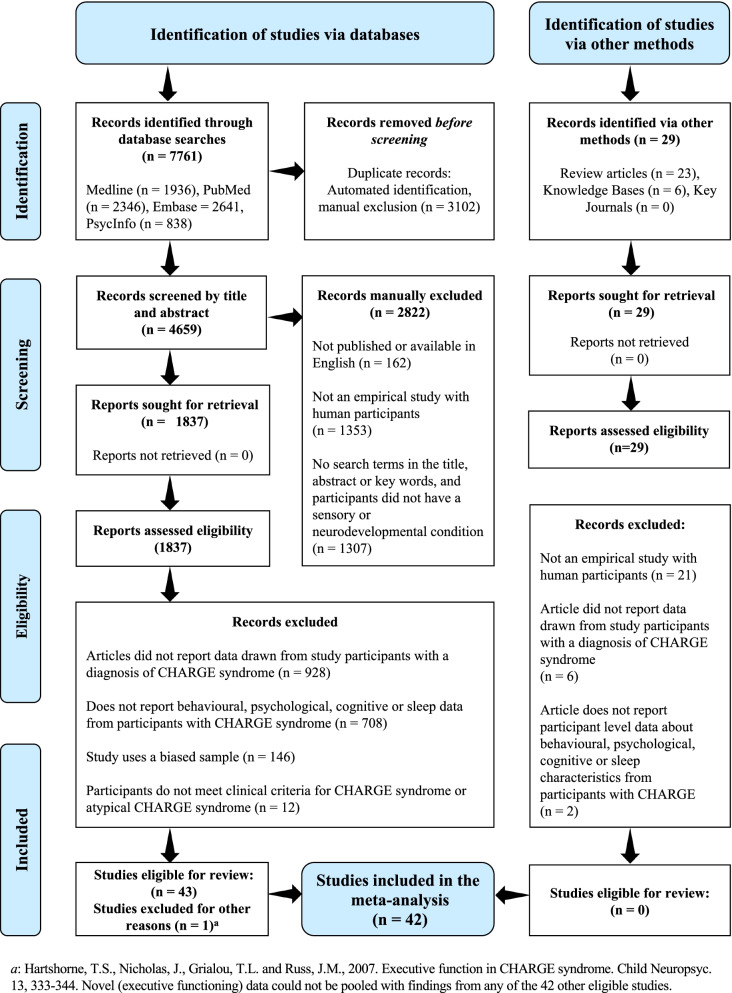
PRISMA diagram of papers included and excluded at each stage of the review process

A PRISMA flowchart summarising study selection is presented in Fig. 1. The search yielded 7761 citations and a further 29 studies were identified through manual searches. A total of 42 studies were eligible for meta-analysis.

### Descriptive data

Table 3 presents the descriptive data and clinical features for CS extracted from eligible studies. In the total sample, 1556 participants were reported to have typical or atypical CS, and 362 diagnoses were confirmed genetically. Studies were published between 1979 and 2020. The mean age of participants was 9.5 years (range < 1 to 53 years) and 51% of participants were male.

**Table 3:** Sample characteristics and clinical features of CHARGE syndrome reported in the eligible studies

	Sample Size (Male)	Mean Age (Range)	CHD7 positive	Coloboma	Choanal Atresia	Anosmia	Facial Palsy	Feeding and Swallowing Difficulties	External Ear Anomaly	Middle Ear Anomaly
Abadie et al. [15]	64 (29)	10.7 (.75-30)	80% (51/64)	75% (46/61)	37% (22/59)	-	46% (22/48)	-	-	-
Asher et al. [41]	16 (-)	-	-	-	100% (16/16)	-	-	-	-	-
Bernstein and Denno [42]	29 (-)	- (3-21)	-	-	-	-	-	-	-	-
Blake and Brown [43]	39 (22)	6 (1.5-20)	-	85% (33/39)	64% (25/39)	-	56% (22/39)	85% (33/39)	87% (34/39)	62% (23/37)
Blake et al. [44]	50 (29)	-	-	91% (43/47)	60% (28/47)	-	45% (21/47)	88% (44/50)	100% (50/50)	66% (33/50)
Blake et al. [19]	30 (14)	17.6 (13-30)	-	90% (27/30)	63% (19/30)	-	-	-	-	-
Cheng et al. [45]	12 (10)	11.9 (.25-21)	100% (12/12)	67% (6/9)	0% (0/10)	-	30% (3/10)	70% (7/10)	83% (10/12)	0% (0/4)
Dammeyer [46]	17 (6)	9 (3-15)	-	-	-	-	-	-	-	-
Davenport et al. [47]	9 (6)	12.2 (1.5-27)	-	89% (8/9)	29% (2/7)	33% (1/3)	29% (2/7)	60% (3/5)	78% (7/9)	50% (1/2)
Deuce et al. [48]	44 (-)	7.9 (1-15)	64% (18/28)	95% (40/42)	55% (23/42)	100% (20/20)	52% (22/42)	84% (37/44)	25% (11/44)	-
Deuce [49]	52 (-)	-	-	-	-	-	-	-	-	-
Dobbelsteyn et al. [50]	39 (24)	6.8 (0.5-17)	-	86% (32/37)	41% (16/39)	-	59% (23/39)	90% (35/39)	-	-
Hale et al. [8]	24 (15)	-	67% (16/24)	78% (18/23)	42% (10/24)	-	-	83% (19/23)	96% (23/24)	-
Hartshorne et al. [51]	87 (52)	11.1 (6-18)	50% (16/32)	84% (73/87)	60% (52/87)	40% (35/87)	48% (42/87)	70% (61/87)	-	-
Hartshorne et al. [18]	53 (33)	- (13-39)	-	77% (41/53)	64% (34/53)	-	-	-	-	-
Hartshorne and Cypher [52]	100 (43)	7^*^ (1-30)	-	86% (86/100)	54% (54/100)	-	48% (48/100)	74% (74/100)	-	-
Hartshorne et al. [53]	160 (85)	10.9 (3-33)	-	85% (136/160)	64% (102/160)	-	48% (77/160)	79% (126/160)	-	-
Harvey et al. [54]	17 (8)	- (0.5-15)	-	88% (15/17)	82% (14/17)	41% (7/17)	100% (9/9)	94% (16/17)	-	100% (9/9)
Hittner et al. [55]	10 (5)	-	-	100% (10/10)	-	-	60% (6/10)	-	100% (10/10)	-
Hsu et al. [56]	20 (6)	9 (0.6-18)	95% (19/20)	95% (19/20)	60% (12/20)	-	-	-	-	-
Husu et al. [57]	18 (6)	8.3 (1-15)	100% (18/18)	94% (16/17)	41% (7/17)	29% (2/7)	40% (6/15)	67% (12/15)	88% (15/17)	-
Issekutz et al. [1]	77 (40)	-	-	77% (59/77)	64% (49/77)	-	49% (38/77)	70% (54/77)	75% (12/16)	81% (13/16)
Johansson et al. [58]	31 (15)	8.9 (0.1-31)	-	-	-	-	39% (12/31)	-	-	23% (7/31)
Jongmans et al. [10]	46 (22)	11.2 (0–40)	100% (46/46)	72% (33/46)	37% (17/46)	-	22% (10/46)	-	-	-
Lasserre et al. [59]	8 (7)	9.8 (7-13)	-	63% (5/8)	-	-	-	-	-	-
Legendre et al. [11]	119 (57)	11 (<1-53)	79% (93/118)	72% (82/117)	43% (49/114)	82% (23/28)	70% (54/77)	84% (65/77)	87% (100/115)	39% (45/115)
Oley et al. [60]	20 (14)	- (0-14)	-	85% (17/20)	65% (13/20)	-	60% (12/20)	25% (5/20)	80% (16/20)	-
Raqbi et al. [61]	21 (11)	8.6 (5-12)	-	90% (19/21)	33% (7/21)	22% (2/9)	76% (16/21)	81% (17/21)	-	-
Reda and Hartshorne [62]	25 (11)	- (1-4.5)	-	88% (22/25)	60% (15/25)	20% (5/25)	36% (9/25)	88% (22/25)	84% (21/25)	-
Roger et al. [63]	45 (23)	-	-	69% (31/45)	60% (27/45)	-	27% (12/45)	70% (31/44)	84% (36/43)	-
Salem-Hartshorne and Jacob [64]	100 (50)	-	-	83% (83/100)	65% (65/100)	-	-	-	-	-
Shiohama et al. [65]	10 (7)	14.7 (-)	-	70% (7/10)	60% (6/10)	60% (6/10)	-	-	-	-
Shoji et al. [66]	25 (13)	9.7 (1-25)	81% (17/21)	56% (14/25)	12 (3/25)	24% (6/25)	24% (6/25)	-	52% (13/25)	-
Smith et al. [67]	13 (8)	9 (3-24)	-	85% (11/13)	62% (8/13)	-	55% (6/11)	83% (10/12)	100% (12/12)	92% (12/13)
Sohn et al. [68]	18 (8)	4.7 (0-20)	100% (18/18)	72% (13/18)	22% (4/18)	50% (2/4)	78% (14/18)	72% (13/18)	100% (18/18)	54% (7/13)
Souriau et al. [69]	71 (-)	8 (<1-30)	-	-	-	55% (17/31)	-	-	-	-
Strömland et al. [70]	31 (15)	8.1 (0.1-31)	-	90% (28/31)	35% (11/31)	-	32% (10/31)	81% (25/31)	90% (28/31)	23% (7/31)
Tellier et al. [71]	47 (17)	-	-	79% (37/47)	57% (27/47)	-	36% (17/47)	96% (45/47)	100% (47/47)	27% (3/11)
Thelin and Fussner [72]	28 (11)	7^*^ (3-27)	-	-	75% (21/28)	11% (3/28)	-	86% (24/28)	-	-
Wessels et al. [73]	13 (7)	5.5 (<1-19)	62% (8/13)	62% (8/13)	54% (7/13)	-	-	-	-	-
Wincent et al. [74]	15 (8)	-	60% (9/15)	80% (12/15)	27% (4/15)	-	21% (3/14)	-	87% (13/15)	-
Wulffaert et al. [17]	22 (16)	11 (2-22)	95% (21/22)	-	-	-	-	-	-	-

	Inner Ear Anomaly	Heart Defect	Growth Deficiency	Genital Hypoplasia	Cleft lip or Palate	Tracheoesophageal Fistula	Brain Anomaly	Characteristic Face	Hearing Impairment
Abadie et al. [15]	95% (61/64)	62% (38/61)	46% (25/55)	51% (24/47)	17% (10/58)	29% (14/48)	50% (26/52)	-	81% (46/57)
Asher et al. [41]	-	-	-	-	-	-	25% (3/12)	-	-
Bernstein and Denno [42]	-	-	-	-	-	-	-	-	100% (29/29)
Blake and Brown [43]	70% (26/37)	79% (31/39)	64% (25/39)	51% (20/39)	18% (7/39)	-	-	-	92% (34/37)
Blake et al. [44]	38% (19/50)	84% (42/50)	91% (39/43)	93% (25/27)	16% (8/50)	11% (5/44)	38% (18/47)	-	74% (37/50)
Blake et al. [19]	-	77% (23/30)	70% (21/30)	73% (22/30)	57% (17/30)	27% (8/30)	-	-	-
Cheng et al. [45]	100% (5/5)	83% (10/12)	63% (5/8)	67% (8/12)	10% (1/10)	-	71% (5/7)	-	100% (12/12)
Dammeyer [46]	-	-	-	-	-	-	-	-	89% (17/19)
Davenport et al. [47]	-	56% (5/9)	67% (6/9)	100% (9/9)	25% (2/8)	33% (1/3)	40% (2/5)	-	100% (8/8)
Deuce et al. [48]	82% (27/33)	30% (13/44)	18% (8/44)	9% (6/44)	7% (3/44)	-	-	5% (2/44)	93% (41/44)
Deuce [49]	-	-	-	-	-	-	-	-	94% (49/52)
Dobbelsteyn et al. [50]	-	79% (31/39)	69% (25/36)	70% (21/30)	26% (10/39)	8% (3/39)	-	59% (17/29)	-
Hale et al. [8]	100% (17/17)	83% (20/24)	70% (16/23)	64% (14/22)	29% (7/24)	17% (4/24)	47 (9/19)	-	96% (23/24)
Hartshorne et al. [51]	-	78% (68/87)	82% (71/87)	62% (54/87)	29% (25/87)	22% (19/87)	-	-	85% (74/87)
Hartshorne et al. [18]	-	74% (39/53)	85% (45/53)	-	42% (22/53)	9% (5/53)	-	75% (40/53)	95% (50.5/53)
Hartshorne and Cypher [52]	-	75% (75/100)	62% (62/100)	39% (39/100)	27% (27/100)	14% (14/100)	-	-	91% (86/94)
Hartshorne et al. [53]	-	80% (128/160)	74% (118/160)	56% (90/160)	29% (46/160)	22% (35/160)	-	-	-
Harvey et al. [54]	-	82% (14/17)	100% (10/10)	56% (5/9)	35% (6/17)	29% (5/17)	24 (4/17)	71% (12/17)	47% (8/17)
Hittner et al. [55]	-	100% (10/10)	-	-	-	-	100% (3/3)	-	100% (10/10)
Hsu et al. [56]	95% (19/20)	-	-	-	-	-	60% (12/20)	-	-
Husu et al. [57]	90% (9/10)	78% (14/18)	60% (9/15)	28% (5/18)	50% (6/18)	19% (3/16)	-	94% (17/18)	93% (14/15)
Issekutz et al. [1]	90% (9/10)	84% (65/77)	58% (45/77)	38% (29/77)	18% (14/77)	19% (15/77)	54% (36/67)	55% (42/77)	58% (15/26)
Johansson et al. [58]	68% (21/31)	-	-	-	-	-	-	-	-
Jongmans et al. [10]	77% (20/26)	65% (30/46)	68% (21/31)	39% (18/46)	37% (17/46)	17% (8/46)	9% (4/46)	-	90% (36/40)
Lasserre et al. [59]	100% (8/8)	-	-	-	-	-	-	-	100% (8/8)
Legendre et al. [11]	95% (107/113)	65% (76/117)	34% (23/67)	53% (54/101)	18% (21/117)	20% (21/107)	49% (50/102)	81% (92/113)	93% (105/113)
Oley et al. [60]	-	80% (16/20)	85% (17/20)	70% (14/20)	-	-	-	-	95% (19/20)
Raqbi et al. [61]	-	76% (16/21)	67% (14/21)	48% (10/21)	14% (3/21)	-	-	-	100% (21/21)
Reda and Hartshorne [62]	-	88% (22/25)	60% (15/25)	48% (12/25)	12% (3/25)	28% (7/25)	-	-	84% (21/25)
Roger et al. [63]	-	78% (35/45)	78% (31/40)	-	13% (6/45)	11% (4/45)	18% (5/28)	-	86% (37/43)
Salem-Hartshorne and Jacob [64]	-	81% (81/100)	80% (80/100)	61% (61/100)	32% (32/100)	25% (25/100)	-	-	82% (82/100)
Shiohama et al. [65]	50% (5/10)	80% (8/10)	90% (9/10)	60% (6/10)	40% (4/10)	20% (2/10)	30% (3/10)	100% (10/10)	-
Shoji et al. [66]	48% (12/25)	76% (19/25)	72% (18/25)	40% (10/25)	44% (11/25)	24 (6/25)	8% (2/25)	-	96% (24/25)
Smith et al. [67]	92% (12/13)	69% (9/13)	62% (8/13)	67% (8/12)	23% (3/13)	17 (2/12)	-	-	-
Sohn et al. [68]	72% (13/18)	72% (13/18)	56% (10/18)	83% (15/18)	-	0% (0/18)	28% (5/18)	-	100% (18/18)
Souriau et al. [69]	-	-	-	-	-	-	-	-	100% (71/71)
Strömland et al. [70]	~ 63% (29/46)	52% (16/31)	48% (15/31)	39% (12/31)	19% (6/31)	16% (5/31)	87% (27/31)	-	74% (23/31)
Tellier et al. [71]	100% (12/12)	85% (40/47)	71% (5/7)	34% (16/47)	36% (17/47)	15% (7/47)	83% (39/47)	81% (38/47)	62% (23/37)
Thelin and Fussner [72]	-	75% (21/28)	57% (16/28)	-	32% (9/28)	-	-	-	96% (27/28)
Wessels et al. [73]	100% (1/1)	69% (9/13)	57% (4/7)	73% (8/11)	25% (3/12)	25% (3/12)	67% (6/9)	43% (3/7)	-
Wincent et al. [74]	83% (5/6)	80% (12/15)	79% (11/14)	57% (8/14)	27% (4/15)	-	-	-	64% (7/11)
Wulffaert et al. [17]	-	-	-	-	-	-	-	-	77% (17/22)

The following studies had overlapping datasets: Smith et al. [67] and Issekutz et al. [1], Johansson et al. [58] and Strömland et al. [70], Hartshorne et al. [17] and Salem-Hartshorne and Jacob [64], and Wincent et al. [74] and Strömland et al. [70]. In this scenario, earlier data sets were given precedence for meta-synthesis, with later studies contributing only original previously unpublished data. Six participants were omitted from Davenport et al. [47] that were previously reported as a familial case series. Four participants from Hale et al. [5], one participant from Jongmans et al. [9] and five participants from Wessels et al. [73] were excluded because they did not meet clinical criteria for CS.

### Clinical features

**Fig. 2:**
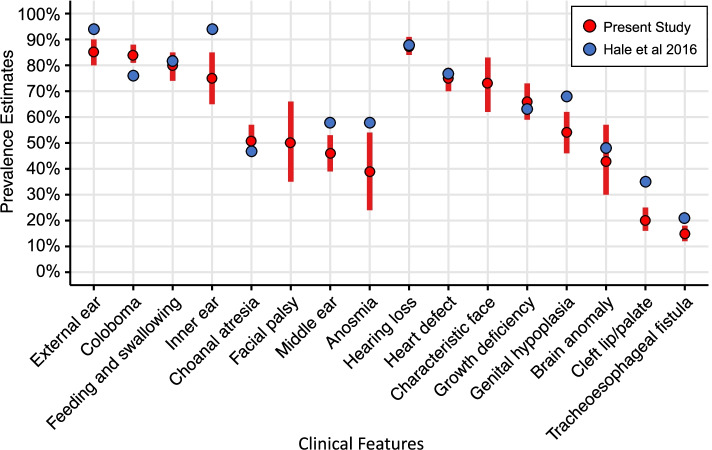
Prevalence of Clinical Features in CHARGE by Hale et al. (2016) and the present study

Figure 2 presents the pooled prevalence estimates and 95% CIs for clinical features of CS drawn from the eligible studies. For comparison, these estimates are presented alongside the largest and most recent review of individuals with a clinical diagnosis by Hale et al. [5]. The prevalence of coloboma were higher and the prevalence of ear anomalies, anosmia, genital hypoplasia, facial clefts and tracheoesophageal fistula were lower in the present study than in Hale et al. [5]. Sufficient data were also available to calculate subcategories of coloboma, choanal atresia, heart defects and structural brain anomalies (see Appendix 7, S1 [21]).

### Physical characteristics and conditions

**Fig. 3:**
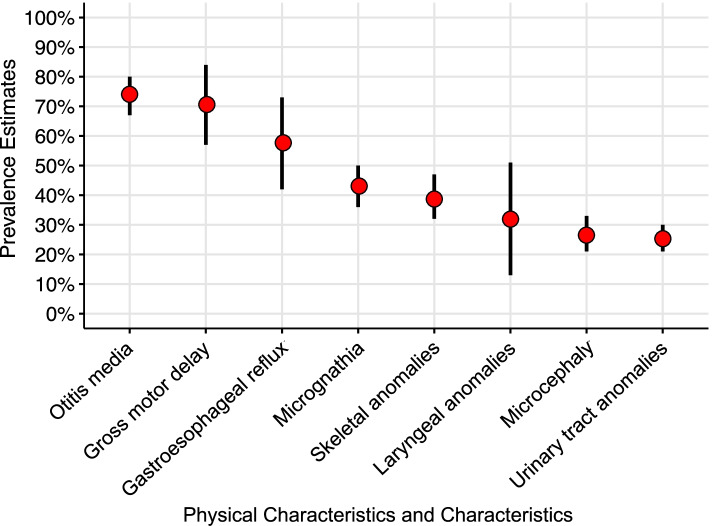
Pooled prevalence estimates and 95% confidence intervals for physical characteristics and conditions

Figure 3 presents pooled prevalence estimates and 95% CIs for physical characteristics and conditions. Estimates included otitis media (74% [95% CI = 67–80]), gross motor difficulties (71% [CI = 13–51%]), gastrointestinal reflux (58% [CI = 42–73]), micrognathia (43% [CI = 57–84%]), microcephaly (43% [CI = 21–33%]) and a 32% (CI = 13–51%) prevalence of laryngeal anomalies. Sufficient data were available to explore types of skeletal anomalies: spinal anomalies (including scoliosis, 28% [CI = 19–36%]) and hand anomalies (15% [11–20%]) (see Appendix 8 and 10, S1 [21].

### Behavioural, psychological, cognitive and sleep characteristics

Table 4 presents data on the study-level prevalence of behavioural, psychological, cognitive and sleep characteristics including: DD, ID, autism, aggression, self-injurious behaviour, obsessive or compulsive behaviour, tactile defensiveness and sleep problems. Two studies used a whole population sample [1, 14], with 25 studies (60%) using single restricted or non-random samples, and 15 studies (36%) using multiple restricted or non-random samples. Seven studies (17%) reported details of clinical diagnosis and genetic testing, with 3 studies (7%) confirming these findings at the time of data collection. Of the remaining 32 articles, 16 (38%) reported which clinical diagnosis participants were assessed against, and 16 (38%) did not. Assessment methods were typically ‘poor’ (64%) with 22% rated as ‘adequate’, 11% as ‘good’ and 4% were rated ‘excellent’.

**Table 4:** Occurrence and quality of behavioural, psychological, cognitive and sleep characteristics reported in CHARGE syndrome

	Sample Identification	Confirmation of Syndrome	Assessment of Cognition	Assessment of Behaviour	Assessment of Sleep	Quality Weighting: Cognition	Quality weighting: Behaviour	Quality Weighting: Sleep
Abadie et al. [15]	4	*2*	2	*NA*	*NA*	67%	67%	*NA*
Asher et al. [41]	2	1	1	*NA*	*NA*	33%	*NA*	*NA*
Bernstein and Denno [42]	2	1	1	2	*NA*	33%	42%	*NA*
Blake and Brown [43]	2	2	1	*NA*	*NA*	42%	*NA*	*NA*
Blake et al. [44]	3	2	1	*NA*	*NA*	50%,	*NA*	*NA*
Blake et al. [19]	3	1	1	1	1	42%	42%	42%
Cheng et al. [45]	2	3	1	1	1	50%	50%	50%
Dammeyer [46]	2	1	3	*NA*	1	50%	*NA*	33%
Davenport et al. [47]	3	2	2	*NA*	*NA*	58%	*NA*	*NA*
Deuce et al. [48]	3	1	1	1	1	*NA*	42%	42%
Deuce [49]	2	1	1	*NA*	1	33%	*NA*	33%
Dobbelsteyn et al. [50]	3	1	1	*NA*	*NA*	42%	*NA*	*NA*
Hale et al. [8]	2	4	1	*NA*	*NA*	58%	*NA*	*NA*
Hartshorne et al. [51]	2	1	*NA*	*NA*	2	*NA*	*NA*	42%
Hartshorne et al. [18]	3	1	1	1	1	42%	42%	42%
Hartshorne and Cypher [52]	2	1	*NA*	1	*NA*	*NA*	33%	*NA*
Hartshorne et al. [53]	2	1	*NA*	2	*NA*	*NA*	42%	*NA*
Harvey et al. [54]	2	2	2	*NA*	*NA*	50%	42%	*NA*
Hittner et al. [55]	2	1	2	*NA*	*NA*	42%	*NA*	*NA*
Hsu et al. [56]	2	4	1	*NA*	*NA*	58%	*NA*	*NA*
Husu et al. [57]	3	3	1	*NA*	*NA*	58%	*NA*	*NA*
Issekutz et al. [1]	4	2	*NA*	1	1	*NA*	58%	58%
Johansson et al. [58]	2	2	2	3	1	50%	58%	42%
Jongmans et al. [10]	2	3	1	*NA*	*NA*	50%	*NA*	*NA*
Lasserre et al. [59]	2	2	3	*NA*	*NA*	58%	*NA*	*NA*
Legendre et al. [11]	3	3	1	*NA*	*NA*	58%	*NA*	*NA*
Oley et al. [60]	3	2	3	*NA*	*NA*	50%	*NA*	*NA*
Raqbi et al. [61]	2	2	1	*NA*	*NA*	42%	*NA*	*NA*
Reda and Hartshorne [62]	2	1	*NA*	2	*NA*	*NA*	42%	*NA*
Roger et al. [63]	3	2	1	*NA*	4	50%	*NA*	75%
Salem-Hartshorne and Jacob [64]	2	1	2	*NA*	*NA*	42%	*NA*	*NA*
Shiohama et al. [65]	2	2	1	*NA*	*NA*	42%	*NA*	*NA*
Shoji et al. [66]	2	2	1	*NA*	*NA*	42%	*NA*	*NA*
Smith et al. [67]	3	2	1	1	*NA*	50%	50%	*NA*
Sohn et al. [68]	3	4	3	*NA*	*NA*	83%	*NA*	*NA*
Souriau et al. [69]	3	1	*NA*	1	*NA*	*NA*	42%	*NA*
Strömland et al. [70]	2	2	2	3	*NA*	50%	58%	*NA*
Tellier et al. [71]	3	2	1	*NA*	4	50%	*NA*	75%
Thelin and Fussner [72]	2	1	*NA*	1	*NA*	*NA*	33%	*NA*
Wessels et al. [73]	2	3	1	*NA*	*NA*	50%	*NA*	*NA*
Wincent et al. [74]	2	3	1	*NA*	*NA*	50%	*NA*	*NA*
Wulffaert et al. [17]	3	3	2	*NA*	*NA*	67%	*NA*	*NA*

	Developmental Delay	Intellectual Disability (ID)	Mild or Moderate ID	Severe or Profound ID	Autism Diagnosis	Aggression	Self-Injurious Behaviour	Obsessive or Compulsive Behaviour	Tactile Defensiveness	Sleep Problems
Abadie et al. [15]	-	70% (45/64)	55% (35/64)	16% (10/64)	54% (25/46)	-	-	-	-	-
Asher et al. [41]	-	100% (14/14)	50% (7/14)	50% (7/14)	-	-	-	-	-	-
Bernstein and Denno [42]	-	90% (26/29)	66% (19/29)	24% (7/29)	-	-	-	72% (21/29)	-	-
Blake and Brown [43]	-	63% (19/30)	-	33% (10/30)	-	-	-	-	-	-
Blake et al. [44]	76% (26/34)	-	-	-	-	-	-	-	-	-
Blake et al. [19]	100% (30/30)	-	-	-	23% (7/30)	53% (16/30)	50% (15/30)	43% (13/30)	40% (12/31)	50% (15/30)
Cheng et al. [45]	-	90% (9/10)	78% (7/9)	11% (1/9)	22% (2/9)	-	-	-	-	17% (2/12)
Dammeyer [46]	-	18% (3/17)	18% (3/17)	-	-	-	-	-	-	88% (15/17)
Davenport et al. [47]	-	100% (7/7)	-	-	-	-	-	-	-	-
Deuce et al. [48]	-	-	-	-	-	-	-	9% (4/44)	-	14% (6/44)
Deuce [49]	-	40% (21/52)	-	-	-	-	-	-	-	52% (27/52)
Dobbelsteyn et al. [50]	95% (35/37)	-	-	-	-	-	-	-	-	-
Hale et al. [8]	100% (23/23)	-	-	-	-	-	-	-	-	-
Hartshorne et al. [51]	-	-	-	-	-	-	-	-	-	57% (50/87)
Hartshorne et al. [18]	85% (45/53)	-	-	-	26% (14/53)	51% (27/53)	47% (25/53)	49% (26/53)	51% (27/53)	58% (31/53)
Hartshorne and Cypher [52]	-	-	-	-	6% (6/100)	38% (38/100)	34% (34/100)	3% (3/100)	53% (53/100)	-
Hartshorne et al. [53]	-	-	-	-	28% (44/160)	-	-	-	-	-
Harvey et al. [54]	-	29% (2/7)	14% (1/7)	14% (1/7)	-	-	-	-	29% (2/7)	-
Hittner et al. [55]	-	100% (7/7)	-	71% (5/7)	-	-	-	-	-	-
Hsu et al. [56]	100% (20/20)	-	-	-	-	-	-	-	-	-
Husu et al. [57]	85% (11/13)	-	-	-	-	-	-	-	-	-
Issekutz et al. [1]	-	-	-	-	-	-	-	44% (7/16)	-	31% (5/16)
Johansson et al. [58]	-	79% (22/28)	36% (10/28)	43% (12/28)	-	68% (19/28)	54% (15/28)	-	-	29% (8/28)
Jongmans et al. [10]	-	74% (23/31)	-	-	37% (17/46)	-	-	-	-	-
Lasserre et al. [59]	-	63% (5/8)	63% (5/8)	-	-	-	-	-	-	-
Legendre et al. [11]	-	66% (63/96)	-	-	18% (21/117)	-	-	-	-	-
Oley et al. [60]	-	60% (12/20)	-	15% (3/20)	-	-	-	-	-	-
Raqbi et al. [61]	-	53% (11/21)	29% (6/21)	24% (5/21)	14% (3/21)	-	-	-	-	-
Reda and Hartshorne [62]	-	-	-	-	12% (3/25)	-	-	-	40% (10/25)	-
Roger et al. [63]	74% (28/38)	-	-	-	13% (6/45)	-	-	-	-	55% (24/44)
Salem-Hartshorne and Jacob [64]	-	46% (46/100)	-	-	32% (32/100)	-	-	-	-	-
Shiohama et al. [65]	100% (10/10)	-	-	-	-	-	-	-	-	-
Shoji et al. [66]	-	100% (25/25)	-	-	-	-	-	-	-	-
Smith et al. [67]	100% (8/8)	100% (11/11)	-	-	60% (6/10)	-	-	-	-	-
Sohn et al. [68]	-	94% (17/18)	-	-	-	-	-	-	-	-
Souriau et al. [69]	-	-	-	-	-	42% (27/67)	44% (28/63)	-	-	-
Strömland et al. [70]	82% (23/28)	-	-	-	20% (5/25)	-	-	-	-	-
Tellier et al. [71]	100% (47/47)	-	-	-	-	-	-	-	-	43% (3/7)
Thelin and Fussner [72]	-	-	-	-	-	46% (13/28)	-	-	-	-
Wessels et al. [73]	100% (9/9)	-	-	-	-	-	-	-	-	-
Wincent et al. [74]	-	-	-	-	-	-	-	-	-	-
Wulffaert et al. [17]	78% (7/9)	75% (15/20)	35% (7/20)	40% (8/20)	-	-	-	-	-	-

Quality weighting scores for cognition, behaviour and sleep represent the total quality rating (sample identification rating + confirmation of syndrome rating + assessment rating [cognition OR behaviour OR sleep]) as a percentage of the total possible rating (4+4+4 = 12)

**Fig. 4:**
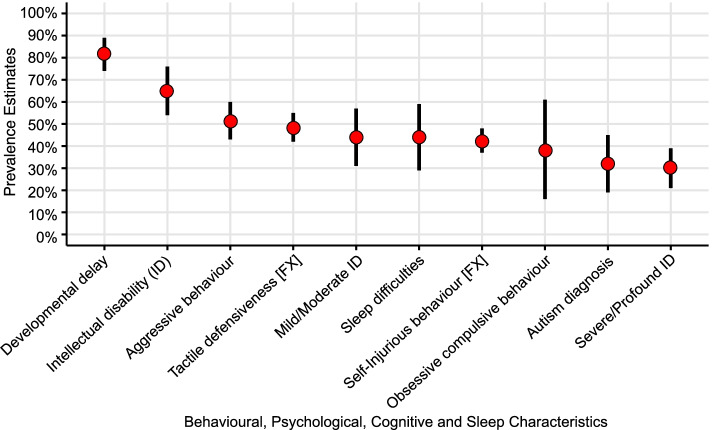
Pooled prevalence estimates and 95% confidence intervals for behavioural, psychological, cognitive and sleep characteristics

We conducted quality weighted random effects meta-analyses for cognitive, behavioural, psychological and sleep characteristics. Results are summarised in Fig. 4 and detailed in Appendix 10 and 11 (S1 [21]). Pooled prevalence estimates included aggression (48% [CI = 40–57%]), tactile defensiveness (48% [CI = 42–55%]), sleep problems (45% [CI = 31–59%]), self-injurious behaviour (44% [CI = 36–51%]), obsessive or compulsive behaviours (36% [CI = 14–57%]) and autism (28% [CI = 16–41%]), DD (84% [CI = 77–91%]), ID (64% [CI = 54–75%]), mild to moderate ID (43% [CI = 31–56%]) and severe or profound ID (28% [CI = 19–37%]). A subgroup difference was identified for studies rated ‘good’ or ‘excellent’ and studies rated ‘poor’ or ‘adequate’ for confirmation of syndrome in the meta-analysis of ID. Full details are included in Appendix 10 (S1 [21]).

### Meta-regression

**Fig. 5:**
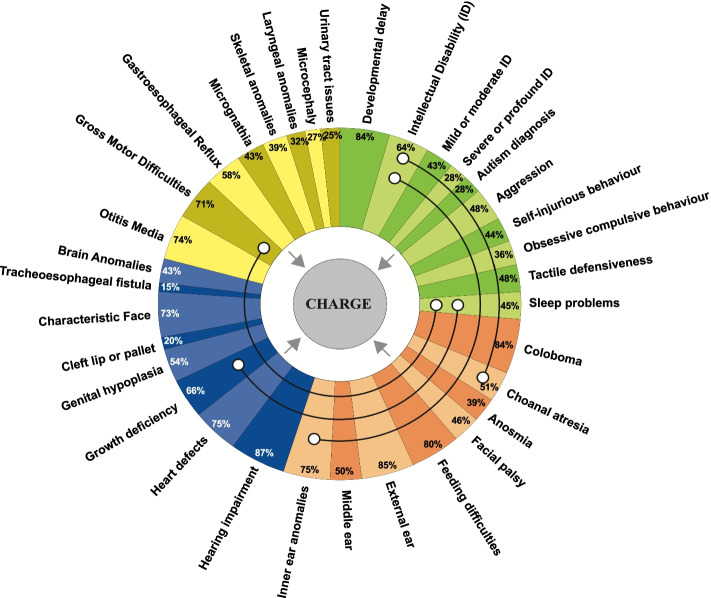
Prevalence estimates and co-occurrence of characteristics in CHARGE syndrome. Legend: pooled prevalence estimates (represented as section size), major clinical features (orange), minor clinical features (blue), physical characteristics and conditions associated with a diagnosis of CHARGE syndrome (yellow), and behavioural, psychological, cognitive and sleep characteristics (green), with association estimates depicted with black lines

Statistically significant associations included more ID and less choanal atresia (*p* = 0.014), more ID and less inner ear anomalies (*p* = 0.014), more sleep problems and more growth deficiency (*p* = 0.001) and more sleep difficulties and more gross motor difficulties (*p* = 0.033). Details are provided in Appendix 12 (S1 [21]). A summary of results is presented in Fig. 5.

### Genotype-phenotype associations

An estimated 84% (*k* = 10; 95% CI = 75–93%) of individuals with CS that had received genetic testing had an identifiable CHD7 mutation. Eleven studies reported prevalence rates for truncating (deletions, frameshift, nonsense and splice site) CHD7 mutations. These studies were amalgamated in a REM, resulting in a pooled prevalence estimate of 80% (95% CI = 71–89%). Prevalence estimates for deletions, frameshift, nonsense and splice site CHD7 mutations were 8%, 24%, 32% and 15% respectively.

A series of meta-regressions were calculated to evaluate evidence for genotype-phenotype associations. There were no statistically significant associations in this series of meta-regressions (details provided in Appendix 13, S1 [21]).

Finally, to supplement our findings, we ran a meta-regression for each characteristic using year of publication as a moderator variable. Significantly more inner ear anomalies (*p* = 0.018) and atrial septal defects were reported over the years (details provided in Appendix 14).

## Discussion

In this comprehensive systematic review and meta-analysis, results indicate that cognitive, behavioural, psychological and sleep difficulties are prevalent in CS. There is substantial variability in the quality of studies and significant differences between study estimates. These prevalence estimates have enabled investigation of relationships between characteristics, facilitating a comprehensive method for describing CS.

### Clinical features

Pooled prevalence estimates of clinical features were largely consistent with previous reports by Hale et al. [5], with the greatest discrepancy being lower estimates for inner ear anomalies. Hale et al. [5] estimates were drawn from studies published between 2005 and 2016, whereas our meta-analysis incorporated research from 1979 to 2020. As inner ear anomalies were not recognised as a clinical feature of CS until 2001 [75], it is possible that the prevalence estimate is conservative. However, there was no significant difference between prevalence rates reported before or after 2001 in our analysis (*p* = 0.111). Therefore, in line with the first aim of the study, we present our results as an up-to-date prevalence estimate of clinical features for future clinical and research practice.

### Physical characteristics and conditions

Of the physical conditions identified in accordance with the second aim of the study, the highest prevalence estimates were for otitis media (74%) and gastroesophageal reflux (58%). There is an established causal link between otitis media and gastroesophageal reflux in typical development [76], but there were too few reports in the current review to assess this association. Gastroesophageal reflux is associated with failure to thrive and with significant mortality in young children with CS [1, 44, 77]. In Cornelia de Lange Syndrome, behavioural indicators of gastroesophageal reflux include night-time agitation, hyperactivity and self-injurious behaviour [78]. Research is required to prospectively identify specific behavioural markers of reflux in CS.

Additional physical characteristics reported by five or more eligible studies included: gross motor difficulties (71%), micrognathia (43%), skeletal anomalies (39%), laryngeal anomalies (32%) and microcephaly (27%). Micrognathia was the only feature of the characteristic CHARGE face described by Blake et al. [3] that was frequently reported as an independent observation. Similarly, almost one third of individuals with CS were estimated to have laryngeal anomalies. These presentations are worthy of consideration in clinical contexts as laryngeal anomalies and micrognathia are known to increase the burden of respiratory and therefore sleep disordered breathing for example in 22q11.2 deletion [79], Treacher Collins and Nager syndromes [80].

### Behavioural, psychological, cognitive and sleep characteristics

In addressing the third aim of the study, quality adjusted prevalence estimates for behavioural, psychological, cognitive and sleep characteristics. The estimated prevalence rates for aggression and self-injurious behaviour are concerning, given the likely impact on parenting stress [16] and quality of life [17]. Once present, self-injury and aggression often persist [81, 82]. Comparable incidence rates of self-injury and aggression have been reported in fragile X (51% and 52%) and Prader-Willi (52% and 43%) [83], and gaps between service need and service provision are reported, despite the availability of evidence-based treatment [84]. This is an area that requires careful monitoring in the CS community. Future research should aim to determine the intensity, frequency and duration of aggression and self-injurious behaviour, through comparison with different genetic syndromes that have shared characteristics. Such research provides the groundwork for tailored interventions based on the specific strengths and difficulties of the individual with CS.

Obsessive-compulsive behaviour was detailed in one of eight studies in which it was reported, despite these behaviours being described as a pervasive manifestation in CS [85]. Given the salience of obsessive-compulsive behaviour, a pragmatic decision was made to include both a clinical diagnosis of obsessive-compulsive disorder [18, 52] and observations reported as obsessive-compulsive behaviour [1, 17, 42, 48]. While estimates did not significantly differ between obsessive-compulsive behaviour and obsessive-compulsive disorder (*p* = 0.414), the quality of assessments was poor for all but one study and estimates ranged from 3 to 72%.

Study estimates for a clinical diagnosis of autism were variable, ranging from 6 to 50%, and this may reflect the range of assessment strategies. Four studies included in the meta-analysis assessed autism, each with a different measure or combination of measures. Autism could not be reliably assessed in 8–12% of participants in two of these studies due to severe sensory impairment and ID [67, 70]. This is concerning, given the increased likelihood of autistic behaviour in this sub-group [58]. The evidence indicates a need for the development of assessments and interventions for autism in CS that are sensitive to the spectrum of reported autistic behaviours.

Given the detrimental effect of poor sleep on learning, behaviour regulation, physical, psychological, and social wellbeing [86], the estimated 45% prevalence of sleep difficulties in CS should not be overlooked. A more nuanced understanding of the characteristics and aetiology of sleep difficulties is required to develop proactive assessment and timely interventions.

The quality weighted pooled prevalence estimate for DD was 84%, with a 64% pooled estimate for ID with an estimated 28% of people with CS experiencing severe or profound ID. While prevalence estimates were characterised by wide CIs, they do suggest greater potential for cognitive development than has been described in previous reviews [8, 22].

### Exploration of co-occurring characteristics

A series of exploratory meta-regression analysis were conducted to explore co-occurring characteristics in accordance with the fourth aim of the study. Meta-regression analysis revealed associations between sleep problems and gross motor difficulties, and sleep problems and growth deficiency. The association between sleep problems and growth deficiency in CS is likely to be multifaceted. For example, growth can be limited by feeding difficulties and chronic illness that may cause pain or necessitate overnight monitoring, compromising sleep [12]. There is also an association between obstructive sleep apnoea and growth failure [87]. Where this condition is due to enlarged tonsils and adenoids, improvement in growth has been reported following adenotonsillectomy [87]. Growth hormone deficiency is also associated with CS [88] and monitoring is recommended as part of multidisciplinary care [12]. Disordered growth hormone secretion can be a consequence of disordered sleep because most growth hormone secretion is triggered by the onset of slow-wave sleep [89, 90]. As such, pain and discomfort, obstructive sleep apnoea and a sleep-disorder-related growth hormone deficit are worthy of consideration in the workup and management of growth deficiency in CS.

With reference to the associations between sleep problems and gross motor difficulties, it is notable that sleep disordered breathing has been shown to have a negative impact on motor development in Down syndrome [91]. Furthermore, children with more gross motor difficulties are likely to walk at a later age. A later age of walking in CS is associated with more ‘challenging behaviour’ [52], ‘autistic behaviour’ [53] and adaptive functioning limitations [64]. This evidence suggests that gross motor development could be a key intervention target for multidisciplinary assessment, including otolaryngology, occupational therapy and developmental paediatrics. In summary, the bidirectional association between sleep and gross motor difficulties, potentially predicted by a later age of walking, warrants further investigation.

The relationship between ID and less choanal atresia seemed unlikely given links between early psychomotor delay and severe respiratory distress [61]. However, as reported in Tellier et al. [71], 48% of infants with bilateral choanal atresia died in the first year of life, before ID could be assessed. Therefore, the association between ID and choanal atresia may simply be an artefact of the data.

### Exploration of genotype-phenotype associations

Consistent with previous reports [7], an estimated 84% of study participants that received genetic testing had an identifiable CHD7 variant. To address aim five, a series of meta-analysis and meta-regression were run with these CHD7-positive participants to evaluate evidence for genotype-phenotype associations.

There is some evidence to suggest that truncating mutations are associated with a more severe CS phenotype [8, 10]. We identified no such association. Based on our findings, and the available literature, we can make no inference to genetically mediated sub-groups within the clinically diagnosed CHARGE syndrome population. However, given the pleotropic nature of CHD7, it is conceivable that the available data was not detailed enough to capture genotype phenotype interactions. Further exploration of CHD7 function through gene expression studies may advance our understanding of Genotype-Phenotype Associations and the pathogenesis of CHARGE syndrome.

## Limitations

This study has some limitations. First, we excluded participants with CHD7 disorder that did not fulfil the clinical criteria for CS. We may therefore have excluded participants with milder CS phenotypes. Conversely, including CS participants for whom clinical features were not reported may have led to the inclusion of non-CS participants. As such, our findings should be treated as preliminary. However, there was no statistical difference between studies that did or did not detail the CS diagnosis. Second, synthesis of the CS literature was hampered by the large number of idiosyncratic descriptions used. For example, the 60% prevalence of ‘increased levels of stress and anxiety’ [49] and 35% incidence of ‘often seemed anxious’ [69] could not be reliably pooled with the 37% and 45% prevalence of anxiety diagnosis reported by Blake et al. [18] and Hartshorne et al. [17] respectively. Anxiety is a multifaceted construct that requires a fine-grained appraisal to facilitate meta-synthesis. Edwards et al. (unpublished results) have used such an approach and report a 37% (95% CI 10–64%; *k* = 2) quality weighted pooled prevalence estimate for anxiety in CHARGE syndrome. A statement on the use of specific, explicit, and appropriate definitions for behaviour in CS should be developed through multidisciplinary collaboration to enable data sharing and pooling. Availability of such data, particularly longitudinal data, would allow researchers to go beyond co-occurring characteristics to understand varying developmental trajectories. Lastly, meta-analytic estimates were limited by the paucity of available data and the wide CIs for the pooled prevalence estimates were not fully explained by meta-regression or subgroup analysis. As such our findings and recommendations should be considered as preliminary. Similarly, the use of univariate analysis to understand causal pathways in a heterogeneous syndrome such as CHARGE is less than adequate. However, multivariate analysis was precluded by the paucity of data. It is feasible that co-occurring characteristics may arise independently, and we emphasise that our findings and recommendations should be interpreted with caution.

## Conclusion

Cognitive, behavioural, psychological and sleep difficulties are highly prevalent in CHARGE syndrome. Future research should address the conceptualisation and description of behaviour in CHARGE syndrome, the development of valid and reliable instruments for psychological diagnosis, and an observational checklist for behavioural signs of gastrointestinal reflux. Future research should use cross-syndrome comparison to understand the clinical presentation of CS. The data from this systematic review and meta-analysis support the ongoing efforts of family support groups, researchers, and practitioners to strengthen understanding and develop appropriate interventions and supports for individuals with CHARGE syndrome, their families and professionals involved in their care.

## Supplementary Information

**Additional file 1:** Appendix 1–14.

**Additional file 2:** R code used for data analysis.

## Data Availability

The supplementary materials supporting the conclusions of this article are available at the Mendeley repository, 10.17632/29kyrfy2dr.5; https://data.mendeley.com/datasets/29kyrfy2dr

## Abbreviations

CS: CHARGE syndrome
CHD7: Chromodomain helicase DNA binding protein 7
DD: Developmental delay
ID: Intellectual development
S1: Supplementary materials 1
CI: Confidence interval
REM: Random effects model
*I*^2^: *I* squared
