# Supplementary material for: Phenotypic characteristics and variability in CHARGE syndrome: a PRISMA compliant systematic review and meta-analysis
Source: J Neurodev Disord. 2022 Aug 31;14:49. doi: 10.1186/s11689-022-09459-5 (PMC9429597; doi:10.1186/s11689-022-09459-5)
Supplement: Supplementary file 1 — Additional file 1: Appendix 1–14. [file 11689_2022_9459_MOESM1_ESM.docx]

**Supplementary Material 1**

**Table of Contents**

[Appendix 1. PRISMA (2020) checklist 11](#_Toc109993004)

[Appendix 2. Detailed search strategy 14](#_Toc109993005)

[**Databases** 14](#_Toc109993006)

[Appendix 3. Reference list of studies identified through manual searches 18](#_Toc109993007)

[**Knowledge base searches:** 18](#_Toc109993008)

[**Review Articles** 20](#_Toc109993009)

[Appendix 4. Inclusion and exclusion criteria used for study selection 24](#_Toc109993010)

[Appendix 5. Quality appraisal 25](#_Toc109993011)

[Appendix 6. Sensitivity assessments 27](#_Toc109993012)

[**The Funnel Plot** 27](#_Toc109993013)

[**The Trim and Fill procedure [2]** 28](#_Toc109993014)

[**The fail-safe *N* [3]** 28](#_Toc109993015)

[**Baujat Plot [4]** 29](#_Toc109993016)

[**Leave one out procedure** 29](#_Toc109993017)

[Appendix 7. Forest plots and sensitivity analysis for ~~diagnostic signs~~ clinical features 30](#_Toc109993018)

[**Coloboma** 30](#_Toc109993019)

[**Choanal Atresia** 35](#_Toc109993020)

[**Anosmia** 39](#_Toc109993021)

[**Facial Palsy** 43](#_Toc109993022)

[**Feeding and Swallowing Difficulties** 47](#_Toc109993023)

[**External Ear Anomalies** 51](#_Toc109993024)

[**Middle Ear Anomalies** 56](#_Toc109993025)

[**Inner Ear Anomalies** 60](#_Toc109993026)

[**Heart Defects** 64](#_Toc109993027)

[**Growth Deficiency** 68](#_Toc109993028)

[**Genital Hypoplasia** 72](#_Toc109993029)

[**Cleft Lip or Palate** 76](#_Toc109993030)

[**Tracheoesophageal Fistula** 80](#_Toc109993031)

[**Brain Anomalies** 84](#_Toc109993032)

[**Characteristic Face** 88](#_Toc109993033)

[**Hearing Impairment** 93](#_Toc109993034)

[**Iris Coloboma** 97](#_Toc109993035)

[**Posterior segment coloboma** 101](#_Toc109993036)

[**Bilateral Coloboma** 105](#_Toc109993037)

[**Bilateral Choanal Atresia** 109](#_Toc109993038)

[Patent Ductus Arteriosus 113](#_Toc109993039)

[**Atrial Septal Defect** 117](#_Toc109993040)

[**Ventricular septal defect** 121](#_Toc109993041)

[**Cerebellar Anomalies** 125](#_Toc109993042)

[**Corpus Callosum** 129](#_Toc109993043)

[**Severe or Profound Hearing Impairment** 133](#_Toc109993044)

[Appendix 8. Incidence of physical characteristics and conditions associated with CHARGE syndrome reported in eligible studies 137](#_Toc109993045)

[Appendix 9. Forest plots and sensitivity analysis for physical characteristics and conditions 147](#_Toc109993046)

[**Recurrent Otitis Media** 147](#_Toc109993047)

[**Gross Motor Difficulties** 151](#_Toc109993048)

[**Gastroesophageal Reflux** 155](#_Toc109993049)

[**Micrognathia** 159](#_Toc109993050)

[**Skeletal Anomalies** 163](#_Toc109993051)

[**Laryngeal Anomalies** 167](#_Toc109993052)

[Microcephaly 171](#_Toc109993053)

[Appendix 10. Details of Quality Weighted Pooled Prevalence Estimates and Subgroup and Sensitivity Analysis for Cognitive, Behavioural, Psychological and Sleep Characteristics 175](#_Toc109993054)

[**Developmental Delay** 175](#_Toc109993055)

[**Intellectual disability** 182](#_Toc109993056)

[**Autism diagnosis** 202](#_Toc109993057)

[**Aggressive Behaviours** 208](#_Toc109993058)

[**Self-Injurious Behaviour** 214](#_Toc109993059)

[**Obsessive or Compulsive Behaviour** 218](#_Toc109993060)

[**Tactile Defensiveness** 223](#_Toc109993061)

[**Sleep Difficulties** 228](#_Toc109993062)

[Appendix 11. Operational Definitions for Behavioural, Psychological, Cognitive and Sleep Characteristics in CHARGE Syndrome 240](#_Toc109993063)

[Appendix 12. Meta-Regression Estimates of The Effects of Co-Occurring Characteristics on Behavioural, Psychological, Cognitive and Sleep Characteristics in CHARGE Syndrome. Statistical Significance Evaluated Using Benjamini-Hochberg Adjustment for Multiple Comparisons 241](#_Toc109993064)

[Appendix 13. Meta-Regression Estimates of Genotype-Phenotype Correlations in CHARGE Syndrome. Statistical Significance Evaluated Using Benjamini-Hochberg Adjustment for Multiple Comparisons 243](#_Toc109993065)

[Appendix 14. Meta-Regression Estimates for Each Characteristic Identified to be Associated with CHARGE syndrome using Year of Publication as the Moderator Variable. Statistical Significance Evaluated Using Benjamini-Hochberg Adjustment for Multiple Comparisons 244](#_Toc109993066)

**Table of Figures**

[**Figure A7.1** QQ Plot of The Distribution of Study Effects and Theoretical Quantities Based on A Normal Distribution Under the Random Effects Model for Studies Reporting Coloboma 31](#_Toc101871455)

[**Figure A7.2** Random Effects Models of The Pooled Prevalence Estimate for Coloboma in CHARGE Syndrome 32](#_Toc101871456)

[**Figure A7.3** Funnel Plot of Standard Error by Prevalence of Coloboma Following the Trim and Fill Procedure 33](#_Toc101871457)

[**Figure A7.4** Baujat Plot of Contribution to Heterogeneity by Influence on Overall Effect for Studies Reporting Coloboma 33](#_Toc101871458)

[**Figure A7.5** Leave-One-Out Random Effects Model for Studies Reporting Coloboma 34](#_Toc101871459)

[**Figure A7.6** QQ Plot of The Distribution of Study Effects and Theoretical Quantities Based on A Normal Distribution Under the Random Effects Model for Studies Reporting Choanal Atresia 35](#_Toc101871460)

[**Figure A7.7** Random Effects Models of The Pooled Prevalence Estimate for Choanal Atresia in CHARGE Syndrome 36](#_Toc101871461)

[**Figure A7.8** Funnel Plot of Standard Error by Prevalence of Choanal Atresia 37](#_Toc101871462)

[**Figure A7.9** Baujat Plot of Contribution to Heterogeneity by Influence on Overall Effect for Studies Reporting Choanal Atresia 37](#_Toc101871463)

[**Figure A7.10** Leave-One-Out Random Effects Model for Studies Reporting Choanal Atresia 38](#_Toc101871464)

[**Figure A7.11** QQ Plot of The Distribution of Study Effects and Theoretical Quantities Based on A Normal Distribution Under the Random Effects Model for Studies Reporting Anosmia 39](#_Toc101871465)

[**Figure A7.12** Random Effects Models of The Pooled Prevalence Estimate for Anosmia in CHARGE Syndrome 40](#_Toc101871466)

[**Figure A7.13** Funnel Plot of Standard Error by Prevalence of Anosmia 41](#_Toc101871467)

[**Figure A7.14** Baujat Plot of Contribution to Heterogeneity by Influence on Overall Effect for Studies Reporting Anosmia 41](#_Toc101871468)

[**Figure A7.15** Leave-One-Out Random Effects Model for Studies Reporting Anosmia 42](#_Toc101871469)

[**Figure A7.16** QQ Plot of The Distribution of Study Effects and Theoretical Quantities Based on A Normal Distribution Under the Random Effects Model for Studies Reporting Facial Palsy 43](#_Toc101871470)

[**Figure A7.17** Random Effects Models of The Pooled Prevalence Estimate for Facial Palsy in CHARGE Syndrome 44](#_Toc101871471)

[**Figure A7.18** Funnel Plot of Standard Error by Prevalence of Facial Palsy 45](#_Toc101871472)

[**Figure A7.19** Baujat Plot of Contribution to Heterogeneity by Influence on Overall Effect for Studies Reporting Facial Palsy 45](#_Toc101871473)

[**Figure A7.20** Leave-One-Out Random Effects Model for Studies Reporting Facial Palsy 46](#_Toc101871474)

[**Figure A7.21** QQ Plot of The Distribution of Study Effects and Theoretical Quantities Based on A Normal Distribution Under the Random Effects Model for Studies Reporting Feeding and Swallowing Difficulties 47](#_Toc101871475)

[**Figure A7.22** Random Effects Models of The Pooled Prevalence Estimate for Feeding and Swallowing Difficulties in CHARGE Syndrome 48](#_Toc101871476)

[**Figure A7.23** Funnel Plot of Standard Error by Prevalence of Feeding and Swallowing Difficulties 49](#_Toc101871477)

[**Figure A7.24** Baujat Plot of Contribution to Heterogeneity by Influence on Overall Effect for Studies Reporting Feeding and Swallowing Difficulties 49](#_Toc101871478)

[**Figure A7.25** Leave-One-Out Random Effects Model for Studies Reporting Feeding and Swallowing Difficulties 50](#_Toc101871479)

[**Figure A7.26** QQ Plot of The Distribution of Study Effects and Theoretical Quantities Based on A Normal Distribution Under the Random Effects Model for Studies Reporting External Ear Anomalies 51](#_Toc101871480)

[**Figure A7.27** Random Effects Models of The Pooled Prevalence Estimate for External Ear Anomalies in CHARGE Syndrome 52](#_Toc101871481)

[**Figure A7.28** Funnel Plot of Standard Error by Prevalence of External Ear Anomalies 53](#_Toc101871482)

[**Figure A7.29** Baujat Plot of Contribution to Heterogeneity by Influence on Overall Effect for Studies Reporting External Ear Anomalies 53](#_Toc101871483)

[**Figure A7.30** Leave-One-Out Random Effects Model for Studies Reporting External Ear Anomalies 54](#_Toc101871484)

[**Figure A7.31** Random Effects Models of The Pooled Prevalence Estimate for External Ear Anomalies in CHARGE Syndrome 55](#_Toc101871485)

[**Figure A7.32** QQ Plot of The Distribution of Study Effects and Theoretical Quantities Based on A Normal Distribution Under the Random Effects Model for Studies Reporting Middle Ear Anomalies 56](#_Toc101871486)

[**Figure A7.33** Random Effects Models of The Pooled Prevalence Estimate for Middle Ear Anomalies in CHARGE Syndrome 57](#_Toc101871487)

[**Figure A7.34** Baujat Plot of Contribution to Heterogeneity by Influence on Overall Effect for Studies Reporting Middle Ear Anomalies 58](#_Toc101871488)

[**Figure A7.35** Leave-One-Out Random Effects Model for Studies Reporting Middle Ear Anomalies 59](#_Toc101871489)

[**Figure A7.36** QQ Plot of The Distribution of Study Effects and Theoretical Quantities Based on A Normal Distribution Under the Random Effects Model for Studies Reporting Inner Ear Anomalies 60](#_Toc101871490)

[**Figure A7.37** Random Effects Models of The Pooled Prevalence Estimate for Inner Ear Anomalies in CHARGE Syndrome 61](#_Toc101871491)

[**Figure A7.38** Funnel Plot of Standard Error by Prevalence of Inner Ear Anomalies Following the Trim and Fill Procedure 62](#_Toc101871492)

[**Figure A7.39** Baujat Plot of Contribution to Heterogeneity by Influence on Overall Effect for Studies Reporting Inner Ear Anomalies 62](#_Toc101871493)

[**Figure A7.40** Leave-One-Out Random Effects Model for Studies Reporting Inner Ear Anomalies 63](#_Toc101871494)

[**Figure A7.41** QQ Plot of The Distribution of Study Effects and Theoretical Quantities Based on A Normal Distribution Under the Random Effects Model for Studies Reporting Heart Defects 64](#_Toc101871495)

[**Figure A7.42** Random Effects Models of The Pooled Prevalence Estimate for Heart Defects in CHARGE Syndrome 65](#_Toc101871496)

[**Figure A7.43** Funnel Plot of Standard Error by Prevalence of Heart Defects 66](#_Toc101871497)

[**Figure A7.44** Baujat Plot of Contribution to Heterogeneity by Influence on Overall Effect for Studies Reporting Heart Defects 66](#_Toc101871498)

[**Figure A7.45** Leave-One-Out Random Effects Model for Studies Reporting Heart Defects 67](#_Toc101871499)

[**Figure A7.46** QQ Plot of The Distribution of Study Effects and Theoretical Quantities Based on A Normal Distribution Under the Random Effects Model for Studies Reporting Growth Deficiency 68](#_Toc101871500)

[**Figure A7.47** Random Effects Models of The Pooled Prevalence Estimate for Growth Deficiency in CHARGE Syndrome 69](#_Toc101871501)

[**Figure A7.48** Funnel Plot of Standard Error by Prevalence of Growth Deficiency 70](#_Toc101871502)

[**Figure A7.49** Baujat Plot of Contribution to Heterogeneity by Influence on Overall Effect for Studies Reporting Growth Deficiency 70](#_Toc101871503)

[**Figure A7.50** Leave-One-Out Random Effects Model for Studies Reporting Growth Deficiency 71](#_Toc101871504)

[**Figure A7.51** QQ Plot of The Distribution of Study Effects and Theoretical Quantities Based on A Normal Distribution Under the Random Effects Model for Studies Reporting Genital Hypoplasia 72](#_Toc101871505)

[**Figure A7.52** Random Effects Models of The Pooled Prevalence Estimate for Genital Hypoplasia in CHARGE Syndrome 73](#_Toc101871506)

[**Figure A7.53** Funnel Plot of Standard Error by Prevalence of Genital Hypoplasia 74](#_Toc101871507)

[**Figure A7.54** Baujat Plot of Contribution to Heterogeneity by Influence on Overall Effect for Studies Reporting Genital Hypoplasia 74](#_Toc101871508)

[**Figure A7.55** Leave-One-Out Random Effects Model for Studies Reporting Genital Hypoplasia 75](#_Toc101871509)

[**Figure A7.56** QQ Plot of The Distribution of Study Effects and Theoretical Quantities Based on A Normal Distribution Under the Random Effects Model for Studies Reporting Cleft Lip or Palate 76](#_Toc101871510)

[**Figure A7.57** Random Effects Models of The Pooled Prevalence Estimate for Cleft Lip or Palate in CHARGE Syndrome 77](#_Toc101871511)

[**Figure A7.58** Funnel Plot of Standard Error by Prevalence of Cleft Lip or Palate Following the Trim and Fill Procedure 78](#_Toc101871512)

[**Figure A7.59** Baujat Plot of Contribution to Heterogeneity by Influence on Overall Effect for Studies Reporting Cleft Lip or Palate 78](#_Toc101871513)

[**Figure A7.60** Leave-One-Out Random Effects Model for Studies Reporting Cleft Lip or Palate 79](#_Toc101871514)

[**Figure A7.61** QQ Plot of The Distribution of Study Effects and Theoretical Quantities Based on A Normal Distribution Under the Random Effects Model for Studies Reporting Tracheoesophageal Fistula 80](#_Toc101871515)

[**Figure A7.62** Random Effects Models of The Pooled Prevalence Estimate for Tracheoesophageal Fistula in CHARGE Syndrome 81](#_Toc101871516)

[**Figure A7.63** Funnel Plot of Standard Error by Prevalence of Tracheoesophageal Fistula Following the Trim and Fill Procedure 82](#_Toc101871517)

[**Figure A7.64** Baujat Plot of Contribution to Heterogeneity by Influence on Overall Effect for Studies Reporting Tracheoesophageal Fistula 82](#_Toc101871518)

[**Figure A7.65** Leave-One-Out Random Effects Model for Studies Reporting Tracheoesophageal Fistula 83](#_Toc101871519)

[**Figure A7.66** QQ Plot of The Distribution of Study Effects and Theoretical Quantities Based on A Normal Distribution Under the Random Effects Model for Studies Reporting Brain Anomalies 84](#_Toc101871520)

[**Figure A7.67** Random Effects Models of The Pooled Prevalence Estimate for Studies Reporting Brain Anomalies in CHARGE Syndrome 85](#_Toc101871521)

[**Figure A7.68** Funnel Plot of Standard Error by Prevalence of Brain Anomalies 86](#_Toc101871522)

[**Figure A7.69** Baujat Plot of Contribution to Heterogeneity by Influence on Overall Effect for Studies Reporting Brain Anomalies 86](#_Toc101871523)

[**Figure A7.70** Leave-One-Out Random Effects Model for Studies Reporting Brain Anomalies 87](#_Toc101871524)

[**Figure A7.71** QQ Plot of The Distribution of Study Effects and Theoretical Quantities Based on A Normal Distribution Under the Random Effects Model for Studies Reporting Characteristic Face 88](#_Toc101871525)

[**Figure A7.72** Random Effects Models of The Pooled Prevalence Estimate for Studies Reporting Characteristic Face in CHARGE Syndrome 89](#_Toc101871526)

[**Figure A7.73** Baujat Plot of Contribution to Heterogeneity by Influence on Overall Effect for Studies Reporting Characteristic Face 90](#_Toc101871527)

[**Figure A7.74** Leave-One-Out Random Effects Model for Studies Reporting Characteristic Face 91](#_Toc101871528)

[**Figure A7.75** Random Effects Models of The Pooled Prevalence Estimate for Studies Reporting Characteristic Face in CHARGE Syndrome 92](#_Toc101871529)

[**Figure A7.76** QQ Plot of The Distribution of Study Effects and Theoretical Quantities Based on A Normal Distribution Under the Random Effects Model for Studies Reporting Hearing Impairment 93](#_Toc101871530)

[**Figure A7.77** Random Effects Models of The Pooled Prevalence Estimate for Studies Reporting Hearing Impairment in CHARGE Syndrome 94](#_Toc101871531)

[**Figure A7.78** Funnel Plot of Standard Error by Prevalence of Hearing Impairment 95](#_Toc101871532)

[**Figure A7.79** Baujat Plot of Contribution to Heterogeneity by Influence on Overall Effect for Studies Reporting Hearing Impairment 95](#_Toc101871533)

[**Figure A7.80** Leave-One-Out Random Effects Model for Studies Reporting Hearing Impairment 96](#_Toc101871534)

[**Figure A7.81** QQ Plot of The Distribution of Study Effects and Theoretical Quantities Based on A Normal Distribution Under the Random Effects Model for Studies Reporting Iris Coloboma 97](#_Toc101871535)

[**Figure A7.82** Random Effects Models of The Pooled Prevalence Estimate for Studies Reporting Iris Coloboma in CHARGE Syndrome 98](#_Toc101871536)

[**Figure A7.83** Funnel Plot of Standard Error by Prevalence of Iris Coloboma 99](#_Toc101871537)

[**Figure A7.84** Baujat Plot of Contribution to Heterogeneity by Influence on Overall Effect for Studies Reporting Iris Coloboma 99](#_Toc101871538)

[**Figure A7.85** Leave-One-Out Random Effects Model for Studies Reporting Iris Coloboma 100](#_Toc101871539)

[**Figure A7.86** QQ Plot of The Distribution of Study Effects and Theoretical Quantities Based on A Normal Distribution Under the Random Effects Model for Studies Reporting Posterior Segment Coloboma 101](#_Toc101871540)

[**Figure A7.87** Random Effects Models of The Pooled Prevalence Estimate for Studies Reporting Posterior Segment Coloboma in CHARGE Syndrome 102](#_Toc101871541)

[**Figure A7.88** Funnel Plot of Standard Error by Prevalence of Posterior Segment Coloboma 103](#_Toc101871542)

[**Figure A7.89** Baujat Plot of Contribution to Heterogeneity by Influence on Overall Effect for Studies Reporting Posterior Segment Coloboma 103](#_Toc101871543)

[**Figure A7.90** Leave-One-Out Random Effects Model for Studies Reporting Posterior Segment Coloboma 104](#_Toc101871544)

[**Figure A7.91** QQ Plot of The Distribution of Study Effects and Theoretical Quantities Based on A Normal Distribution Under the Random Effects Model for Studies Reporting Bilateral Coloboma 105](#_Toc101871545)

[**Figure A7.92** Random Effects Models of The Pooled Prevalence Estimate for Studies Reporting Bilateral Coloboma in CHARGE Syndrome 106](#_Toc101871546)

[**Figure A7.93** Funnel Plot of Standard Error by Prevalence of Bilateral Coloboma 107](#_Toc101871547)

[**Figure A7.94** Baujat Plot of Contribution to Heterogeneity by Influence on Overall Effect for Studies Reporting Bilateral Coloboma 107](#_Toc101871548)

[**Figure A7.95** Leave-One-Out Random Effects Model for Studies Reporting Bilateral Coloboma 108](#_Toc101871549)

[**Figure A7.96** QQ Plot of The Distribution of Study Effects and Theoretical Quantities Based on A Normal Distribution Under the Random Effects Model for Studies Reporting Bilateral Choanal Atresia 109](#_Toc101871550)

[**Figure A7.97** Random Effects Models of The Pooled Prevalence Estimate for Studies Reporting Bilateral Choanal Atresia in CHARGE Syndrome 110](#_Toc101871551)

[**Figure A7.98** Funnel Plot of Standard Error by Prevalence of Bilateral Choanal Atresia Following the Trim and Fill Procedure 111](#_Toc101871552)

[**Figure A7.99** Baujat Plot of Contribution to Heterogeneity by Influence on Overall Effect for Studies Reporting Bilateral Choanal Atresia 111](#_Toc101871553)

[**Figure A7.100** Leave-One-Out Random Effects Model for Studies Reporting Bilateral Choanal Atresia 112](#_Toc101871554)

[**Figure A7.101** QQ Plot of The Distribution of Study Effects and Theoretical Quantities Based on A Normal Distribution Under the Random Effects Model for Studies Reporting Patent Ductus Arteriosus 113](#_Toc101871555)

[**Figure A7.102** Random Effects Models of The Pooled Prevalence Estimate for Studies Reporting Patent Ductus Arteriosus in CHARGE Syndrome 114](#_Toc101871556)

[**Figure A7.103** Funnel Plot of Standard Error by Prevalence of Patent Ductus Arteriosus Following the Trim and Fill Procedure 115](#_Toc101871557)

[**Figure A7.104** Baujat Plot of Contribution to Heterogeneity by Influence on Overall Effect for Studies Reporting Patent Ductus Arteriosus 115](#_Toc101871558)

[**Figure A7.105** Leave-One-Out Random Effects Model for Studies Reporting Patent Ductus Arteriosus 116](#_Toc101871559)

[**Figure A7.106** QQ Plot of The Distribution of Study Effects and Theoretical Quantities Based on A Normal Distribution Under the Random Effects Model for Studies Reporting Atrial Septal Defect 117](#_Toc101871560)

[**Figure A7.107** Random Effects Models of The Pooled Prevalence Estimate for Studies Reporting Atrial Septal Defect in CHARGE Syndrome 118](#_Toc101871561)

[**Figure A7.108** Funnel Plot of Standard Error by Prevalence of Atrial Septal Defect 119](#_Toc101871562)

[**Figure A7.109** Baujat Plot of Contribution to Heterogeneity by Influence on Overall Effect for Studies Reporting Atrial Septal Defect 119](#_Toc101871563)

[**Figure A7.110** Leave-One-Out Random Effects Model for Studies Reporting Atrial Septal Defect 120](#_Toc101871564)

[**Figure A7.111** QQ Plot of The Distribution of Study Effects and Theoretical Quantities Based on A Normal Distribution Under the Random Effects Model for Studies Reporting Ventricular Septal Defect 121](#_Toc101871565)

[**Figure A7.112** Random Effects Models of The Pooled Prevalence Estimate for Studies Reporting Ventricular Septal Defect in CHARGE Syndrome 122](#_Toc101871566)

[**Figure A7.113** Funnel Plot of Standard Error by Prevalence of Ventricular Septal Defect 123](#_Toc101871567)

[**Figure A7.114** Baujat Plot of Contribution to Heterogeneity by Influence on Overall Effect for Studies Reporting Ventricular Septal Defect 123](#_Toc101871568)

[**Figure A7.115** Leave-One-Out Random Effects Model for Studies Reporting Ventricular Septal Defect 124](#_Toc101871569)

[**Figure A7.116** QQ Plot of The Distribution of Study Effects and Theoretical Quantities Based on A Normal Distribution Under the Random Effects Model for Studies Reporting Cerebellar Anomalies 125](#_Toc101871570)

[**Figure A7.117** Random Effects Models of The Pooled Prevalence Estimate for Studies Reporting Cerebellar Anomalies in CHARGE Syndrome 126](#_Toc101871571)

[**Figure A7.118** Funnel Plot of Standard Error by Prevalence of Cerebellar Anomalies Following the Trim and Fill Procedure 127](#_Toc101871572)

[**Figure A7.119** Baujat Plot of Contribution to Heterogeneity by Influence on Overall Effect for Studies Reporting Cerebellar Anomalies 127](#_Toc101871573)

[**Figure A7.120** Leave-One-Out Random Effects Model for Studies Reporting Cerebellar Anomalies 128](#_Toc101871574)

[**Figure A7.121** QQ Plot of The Distribution of Study Effects and Theoretical Quantities Based on A Normal Distribution Under the Random Effects Model for Studies Reporting Corpus Callosum Anomalies 129](#_Toc101871575)

[**Figure A7.122** Random Effects Models of The Pooled Prevalence Estimate for Studies Reporting Corpus Callosum Anomalies in CHARGE Syndrome 130](#_Toc101871576)

[**Figure A7.123** Funnel Plot of Standard Error by Prevalence of Corpus Callosum Anomalies Following the Trim and Fill Procedure 131](#_Toc101871577)

[**Figure A7.124** Baujat Plot of Contribution to Heterogeneity by Influence on Overall Effect for Studies Reporting Corpus Callosum Anomalies 131](#_Toc101871578)

[**Figure A7.125** Leave-One-Out Random Effects Model for Studies Reporting Corpus Callosum Anomalies 132](#_Toc101871579)

[**Figure A7.126** QQ Plot of The Distribution of Study Effects and Theoretical Quantities Based on A Normal Distribution Under the Random Effects Model for Studies Reporting Severe or Profound Hearing Impairment 133](#_Toc101871580)

[**Figure A7.127** Random Effects Models of The Pooled Prevalence Estimate for Studies Reporting Severe or Profound Hearing Impairment in CHARGE Syndrome 134](#_Toc101871581)

[**Figure A7.128** Funnel Plot of Standard Error by Prevalence of Severe or Profound Hearing Impairment 135](#_Toc101871582)

[**Figure A7.129** Baujat Plot of Contribution to Heterogeneity by Influence on Overall Effect for Studies Reporting Severe or Profound Hearing Impairment 135](#_Toc101871583)

[**Figure A7.130** Leave-One-Out Random Effects Model for Studies Reporting Severe or Profound Hearing Impairment 136](#_Toc101871584)

[**Figure A9.1** QQ Plot of The Distribution of Study Effects and Theoretical Quantities Based on A Normal Distribution Under the Random Effects Model for Studies Reporting Recurrent Otitis Media 147](#_Toc101871585)

[**Figure A9.2** Random Effects Models of The Pooled Prevalence Estimate for Studies Reporting Recurrent Otitis Media in CHARGE Syndrome 148](#_Toc101871586)

[**Figure A9.3** Baujat Plot of Contribution to Heterogeneity by Influence on Overall Effect for Studies Reporting Recurrent Otitis Media 149](#_Toc101871587)

[**Figure A9.4** Leave-One-Out Random Effects Model for Studies Reporting Recurrent Otitis Media 150](#_Toc101871588)

[**Figure A9.5** QQ Plot of The Distribution of Study Effects and Theoretical Quantities Based on A Normal Distribution Under the Random Effects Model for Studies Reporting Gross Motor Difficulties 151](#_Toc101871589)

[**Figure A9.6** Random Effects Models of The Pooled Prevalence Estimate for Studies Reporting Recurrent Gross Motor Difficulties in CHARGE Syndrome 152](#_Toc101871590)

[**Figure A9.7** Funnel Plot of Standard Error by Prevalence of Gross Motor Difficulties Following the Trim and Fill Procedure 153](#_Toc101871591)

[**Figure A9.8** Baujat Plot of Contribution to Heterogeneity by Influence on Overall Effect for Studies Reporting Gross Motor Difficulties 153](#_Toc101871592)

[**Figure A9.9** Leave-One-Out Random Effects Model for Studies Reporting Gross Motor Difficulties 154](#_Toc101871593)

[**Figure A9.10** QQ Plot of The Distribution of Study Effects and Theoretical Quantities Based on A Normal Distribution Under the Random Effects Model for Studies Reporting Gastroesophageal Reflux 155](#_Toc101871594)

[**Figure A9.11** Random Effects Models of The Pooled Prevalence Estimate for Studies Reporting Recurrent Gastroesophageal Reflux in CHARGE Syndrome 156](#_Toc101871595)

[**Figure A9.12** Baujat Plot of Contribution to Heterogeneity by Influence on Overall Effect for Studies Reporting Gastroesophageal Reflux 157](#_Toc101871596)

[**Figure A9.13** Leave-One-Out Random Effects Model for Studies Reporting Gastroesophageal Reflux 158](#_Toc101871597)

[**Figure A9.14** QQ Plot of The Distribution of Study Effects and Theoretical Quantities Based on A Normal Distribution Under the Random Effects Model for Studies Reporting Micrognathia 159](#_Toc101871598)

[**Figure A9.15** Random Effects Models of The Pooled Prevalence Estimate for Studies Reporting Micrognathia in CHARGE Syndrome 160](#_Toc101871599)

[**Figure A9.16** QQ Plot of The Distribution of Study Effects and Theoretical Quantities Based on A Normal Distribution Under the Fixed Effects Model for Studies Reporting Micrognathia 161](#_Toc101871600)

[**Figure A9.17** Fixed Effects Models of The Pooled Prevalence Estimate for Studies Reporting Micrognathia in CHARGE Syndrome 162](#_Toc101871601)

[**Figure A9.18** QQ Plot of The Distribution of Study Effects and Theoretical Quantities Based on A Normal Distribution Under the Random Effects Model for Studies Reporting Skeletal Anomalies 163](#_Toc101871602)

[**Figure A9.19** Random Effects Models of The Pooled Prevalence Estimate for Studies Reporting Skeletal Anomalies in CHARGE Syndrome 164](#_Toc101871603)

[**Figure A7.20** Funnel Plot of Standard Error by Prevalence of Skeletal Anomalies 165](#_Toc101871604)

[**Figure A7.21** Baujat Plot of Contribution to Heterogeneity by Influence on Overall Effect for Studies Reporting Skeletal Anomalies 165](#_Toc101871605)

[**Figure A9.22** Leave-One-Out Random Effects Model for Studies Reporting Skeletal Anomalies 166](#_Toc101871606)

[**Figure A9.23** QQ Plot of The Distribution of Study Effects and Theoretical Quantities Based on A Normal Distribution Under the Random Effects Model for Studies Reporting Laryngeal Anomalies 167](#_Toc101871607)

[**Figure A9.24** Random Effects Models of The Pooled Prevalence Estimate for Studies Reporting Laryngeal Anomalies in CHARGE Syndrome 168](#_Toc101871608)

[**Figure A7.25** Baujat Plot of Contribution to Heterogeneity by Influence on Overall Effect for Studies Reporting Laryngeal Anomalies 169](#_Toc101871609)

[**Figure A9.26** Leave-One-Out Random Effects Model for Studies Reporting Laryngeal Anomalies 170](#_Toc101871610)

[**Figure A9.27** QQ Plot of The Distribution of Study Effects and Theoretical Quantities Based on A Normal Distribution Under the Random Effects Model for Studies Reporting Microcephaly 171](#_Toc101871611)

[**Figure A9.28** Random Effects Models of The Pooled Prevalence Estimate for Studies Reporting Microcephaly in CHARGE Syndrome 172](#_Toc101871612)

[**Figure A7.29** Baujat Plot of Contribution to Heterogeneity by Influence on Overall Effect for Studies Reporting Microcephaly 173](#_Toc101871613)

[**Figure A9.30** Leave-One-Out Random Effects Model for Studies Reporting Microcephaly 174](#_Toc101871614)

[**Figure A10.1** QQ Plot of The Distribution of Study Effects and Theoretical Quantities Based on A Normal Distribution Under the Random Effects Model for Studies Reporting Developmental Delay 176](#_Toc101871615)

[**Figure A10.2** Random Effects Models of The Pooled Prevalence Estimate for Studies Reporting Developmental Delay in CHARGE Syndrome 177](#_Toc101871616)

[**Figure A10.3** Baujat Plot of Contribution to Heterogeneity by Influence on Overall Effect for Studies Reporting Developmental Delay 178](#_Toc101871617)

[**Figure A10.4** Leave-One-Out Random Effects Model for Studies Reporting Developmental Delay 179](#_Toc101871618)

[**Figure A10.5** Subgroup Analysis of Studies Reporting Developmental Delay that were Rated Adequate and Studies Rated Good for Sample Identification 180](#_Toc101871619)

[**Figure A10.6** Subgroup Analysis of Studies Reporting Developmental Delay that were Rated Adequate and Studies Rated Good for Sample Identification 181](#_Toc101871620)

[**Figure A10.7** QQ Plot of The Distribution of Study Effects and Theoretical Quantities Based on A Normal Distribution Under the Random Effects Model for Studies Reporting Intellectual Disability 183](#_Toc101871621)

[**Figure A10.8** Random Effects Models of The Pooled Prevalence Estimate for Studies Reporting Intellectual Disability in CHARGE Syndrome 184](#_Toc101871622)

[**Figure A10.9** Funnel Plot of Standard Error by Prevalence of Intellectual Disability 185](#_Toc101871623)

[**Figure A10.10** Baujat Plot of Contribution to Heterogeneity by Influence on Overall Effect for Studies Reporting Intellectual Disability 185](#_Toc101871624)

[**Figure A10.11** Leave-One-Out Random Effects Model for Studies Reporting Intellectual Disability 186](#_Toc101871625)

[**Figure A10.12** Subgroup Analysis of Studies Reporting Intellectual Disability that were Rated Adequate and Studies Rated Good/Excellent for Sample Identification 187](#_Toc101871626)

[**Figure A10.13** Subgroup Analysis of Studies Reporting Intellectual Disability that were Rated Poor/Adequate and Studies Rated Good for Method of Assessment 188](#_Toc101871627)

[**Figure A10.14** QQ Plot of The Distribution of Study Effects and Theoretical Quantities Based on A Normal Distribution Under the Random Effects Model for Studies Reporting Mild or Moderate Intellectual Disability 189](#_Toc101871628)

[**Figure A10.15** Random Effects Models of The Pooled Prevalence Estimate for Studies Reporting Mild or Moderate Intellectual Disability in CHARGE Syndrome 190](#_Toc101871629)

[Figure A10.16 Funnel Plot of Standard Error by Prevalence of Mild or Moderate Intellectual Disability 191](#_Toc101871630)

[Figure A10.17 Baujat Plot of Contribution to Heterogeneity by Influence on Overall Effect for Studies Reporting Mild or Moderate Intellectual Disability 191](#_Toc101871631)

[**Figure A10.18** Leave-One-Out Random Effects Model for Studies Reporting Mild or Moderate Intellectual Disability 192](#_Toc101871632)

[**Figure A10.19** Subgroup Analysis of Studies Reporting Mild or Moderate Intellectual Disability that were Rated Adequate and Studies Rated Good/Excellent for Sample Identification 193](#_Toc101871633)

[**Figure A10.20** Subgroup Analysis of Studies Reporting Mild or Moderate Intellectual Disability that were Rated Poor/Adequate and Studies Rated Good for Confirmation of Syndrome 194](#_Toc101871634)

[**Figure A10.21** Subgroup Analysis of Studies Reporting Mild or Moderate Intellectual Disability that were Rated Poor/Adequate and Studies Rated Good for Method of Assessment 195](#_Toc101871635)

[**Figure A10.22** QQ Plot of The Distribution of Study Effects and Theoretical Quantities Based on A Normal Distribution Under the Random Effects Model for Studies Reporting Severe or Profound Intellectual Disability 196](#_Toc101871636)

[**Figure A10.23** Random Effects Models of The Pooled Prevalence Estimate for Studies Reporting Severe or Profound Intellectual Disability in CHARGE Syndrome 197](#_Toc101871637)

[**Figure A10.24** Funnel Plot of Standard Error by Prevalence of Severe or Profound Intellectual Disability Following the Trim and Fill Procedure 198](#_Toc101871638)

[**Figure A10.25** Baujat Plot of Contribution to Heterogeneity by Influence on Overall Effect for Studies Reporting Severe or Profound Intellectual Disability 198](#_Toc101871639)

[**Figure A10.26** Leave-One-Out Random Effects Model for Studies Reporting Severe or Profound Intellectual Disability 199](#_Toc101871640)

[**Figure A10.27** Subgroup Analysis of Studies Reporting Severe or Profound Intellectual Disability that were Rated Adequate and Studies Rated Good/Excellent for Sample Identification 200](#_Toc101871641)

[**Figure A10.28** Subgroup Analysis of Studies Reporting Severe or Profound Intellectual Disability that were Rated Poor/Adequate and Studies Rated Good for Confirmation of Syndrome 201](#_Toc101871642)

[**Figure A10.29** QQ Plot of The Distribution of Study Effects and Theoretical Quantities Based on A Normal Distribution Under the Random Effects Model for Studies Reporting Autism 203](#_Toc101871643)

[**Figure A10.30** Random Effects Models of The Pooled Prevalence Estimate for Studies Reporting Autism in CHARGE Syndrome 204](#_Toc101871644)

[**Figure A10.31** Baujat Plot of Contribution to Heterogeneity by Influence on Overall Effect for Studies Reporting Autism 205](#_Toc101871645)

[**Figure A10.32** Leave-One-Out Random Effects Model for Studies Reporting Autism 206](#_Toc101871646)

[**Figure A10.33** Subgroup Analysis of Studies Reporting Autism that were Rated Adequate and Studies Rated Good/Excellent for Sample Identification 207](#_Toc101871647)

[**Figure A10.34** QQ Plot of The Distribution of Study Effects and Theoretical Quantities Based on A Normal Distribution Under the Random Effects Model for Studies Reporting Aggressive Behaviour 209](#_Toc101871648)

[**Figure A10.35** Random Effects Models of The Pooled Prevalence Estimate for Studies Reporting Aggressive Behaviour in CHARGE Syndrome 210](#_Toc101871649)

[**Figure A10.36** Baujat Plot of Contribution to Heterogeneity by Influence on Overall Effect for Studies Reporting Aggressive Behaviour 211](#_Toc101871650)

[**Figure A10.37** Leave-One-Out Random Effects Model for Studies Reporting Aggressive Behaviour 212](#_Toc101871651)

[**Figure A10.38** Subgroup Analysis of Studies Reporting Aggressive Behaviour that were Rated Adequate and Studies Rated Good for Sample Identification 213](#_Toc101871652)

[**Figure A10.39** QQ Plot of The Distribution of Study Effects and Theoretical Quantities Based on A Normal Distribution Under the Random Effects Model for Studies Reporting Self-Injurious Behaviour 214](#_Toc101871653)

[**Figure A10.40** Random Effects Models of The Pooled Prevalence Estimate for Studies Reporting Self-Injurious Behaviour in CHARGE Syndrome 215](#_Toc101871654)

[**Figure A10.41** QQ Plot of The Distribution of Study Effects and Theoretical Quantities Based on A Normal Distribution Under the Fixed Effects Model for Studies Reporting Self-Injurious Behaviour 216](#_Toc101871655)

[**Figure A10.42** Fixed Effects Models of The Pooled Prevalence Estimate for Studies Reporting Self-Injurious Behaviour in CHARGE Syndrome 217](#_Toc101871656)

[**Figure A10.43** QQ Plot of The Distribution of Study Effects and Theoretical Quantities Based on A Normal Distribution Under the Random Effects Model for Studies Reporting Obsessive or Compulsive Behaviour 218](#_Toc101871657)

[**Figure A10.44** Random Effects Models of The Pooled Prevalence Estimate for Studies Reporting Obsessive or Compulsive Behaviour in CHARGE Syndrome 219](#_Toc101871658)

[**Figure A10.45** Baujat Plot of Contribution to Heterogeneity by Influence on Overall Effect for Studies Reporting Obsessive or Compulsive Behaviour 220](#_Toc101871659)

[**Figure A10.46** Leave-One-Out Random Effects Model for Studies Reporting Obsessive or Compulsive Behaviour 221](#_Toc101871660)

[**Figure A10.47** Subgroup Analysis of Studies Reporting Obsessive or Compulsive Behaviour that were Rated Adequate and Studies Rated Good/Excellent for Sample Identification 222](#_Toc101871661)

[**Figure A10.48** QQ Plot of The Distribution of Study Effects and Theoretical Quantities Based on A Normal Distribution Under the Random Effects Model for Studies Reporting Tactile Defensiveness 224](#_Toc101871662)

[**Figure A10.49** Random Effects Models of The Pooled Prevalence Estimate for Studies Reporting Tactile Defensiveness in CHARGE Syndrome 225](#_Toc101871663)

[**Figure A10.50** QQ Plot of The Distribution of Study Effects and Theoretical Quantities Based on A Normal Distribution Under the Fixed Effects Model for Studies Reporting Tactile Defensiveness 226](#_Toc101871664)

[**Figure A10.51** Fixed Effects Models of The Pooled Prevalence Estimate for Studies Reporting Tactile Defensiveness in CHARGE Syndrome 227](#_Toc101871665)

[**Figure A10.52** QQ Plot of The Distribution of Study Effects and Theoretical Quantities Based on A Normal Distribution Under the Random Effects Model for Studies Reporting Sleep Difficulties 229](#_Toc101871666)

[**Figure A10.53** Random Effects Models of The Pooled Prevalence Estimate for Studies Reporting Sleep Difficulties in CHARGE Syndrome 230](#_Toc101871667)

[**Figure A10.54** Funnel Plot of Standard Error by Prevalence of Sleep Difficulties Following the Trim and Fill Procedure 231](#_Toc101871668)

[**Figure A10.55** Baujat Plot of Contribution to Heterogeneity by Influence on Overall Effect for Studies Reporting Sleep Difficulties 231](#_Toc101871669)

[**Figure A10.56** Leave-One-Out Random Effects Model for Studies Reporting Sleep Difficulties 232](#_Toc101871670)

[**Figure A10.57** Subgroup Analysis of Studies Reporting Sleep Difficulties that were Rated Adequate and Studies Rated Good/Excellent for Sample Identification 233](#_Toc101871671)

[**Figure A10.58** Subgroup Analysis of Studies Reporting Sleep Difficulties that were Rated Poor/Adequate and Studies Rated Excellent for Method of Sleep Assessment 234](#_Toc101871672)

**Table of Tables**

[**Table A1.1** PRISMA 2020 Checklist 12](#_Toc101871364)

[**Table A2.** Inclusion and exclusion criteria used for study selection 25](#_Toc101871365)

[**Table A8.1** Physical Characteristics and Conditions Associated with CHARGE Syndrome in the Eligible Studies 137](#_Toc101871366)

[**Table A11.1** Operational Definitions Used to Identify Behavioural, Psychological, Cognitive and Sleep Characteristics Reported in the Literature 240](#_Toc101871367)

[**Table A12.1** Meta-Regression Estimates for of the Effects of Co-Occurring Characteristics on Behavioural, Psychological, Cognitive and Sleep Characteristics in the Eligible CHARGE Syndrome Literature 241](#_Toc101871368)

[**Table A13.1** Meta-Regression Estimates of Genotype-Phenotype Correlations in the Eligible CHARGE Syndrome Literature 243](#_Toc101871369)

[**Table A14.1** Meta-Regression Estimates for Each Characteristic Identified to be Associated with CHARGE syndrome using Year of Publication as the Moderator Variable 244](#_Toc101871370)

# **
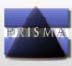
Appendix 1.** PRISMA (2020) checklist

##### **Table A1.1** PRISMA 2020 Checklist

| **Section and Topic** | **Item** | **Checklist item** | **Location where item is reported** |
| --- | --- | --- | --- |
| **TITLE** | | |  |
| Title | 1 | Identify the report as a systematic review. | Line 1&2 |
| **ABSTRACT** | | |  |
| Abstract | 2 | See the PRISMA 2020 for Abstracts checklist. | Line 5-24 |
| **INTRODUCTION** | | |  |
| Rationale | 3 | Describe the rationale for the review in the context of existing knowledge. | Line 30-68 |
| Objectives | 4 | Provide an explicit statement of the objective(s) or question(s) the review addresses. | Line 70-94 |
| **METHODS** | | |  |
| Eligibility criteria | 5 | Specify the inclusion and exclusion criteria for the review and how studies were grouped for the syntheses. | Inclusion and exclusion criteria for the systematic review are detailed in Appendix 2-4 (S1). Grouping for syntheses is outlined in Appendix 6 |
| Information sources | 6 | Specify all databases, registers, websites, organisations, reference lists and other sources searched or consulted to identify studies. Specify the date when each source was last searched or consulted. | See Appendix 2 & Appendix 3 (S1) for full details |
| Search strategy | 7 | Present the full search strategies for all databases, registers and websites, including any filters and limits used. | See Appendix 2 & Appendix 3 (S1) for full details |
| Selection process | 8 | Specify the methods used to decide whether a study met the inclusion criteria of the review, including how many reviewers screened each record and each report retrieved, whether they worked independently, and if applicable, details of automation tools used in the process. | Lines 109-112 & Appendix 4 (S1) |
| Data collection process | 9 | Specify the methods used to collect data from reports, including how many reviewers collected data from each report, whether they worked independently, any processes for obtaining or confirming data from study investigators, and if applicable, details of automation tools used in the process. | Lines 115-118 |
| Data items | 10a | List and define all outcomes for which data were sought. Specify whether all results that were compatible with each outcome domain in each study were sought (e.g. for all measures, time points, analyses), and if not, the methods used to decide which results to collect. | Lines 115 -118 & Appendix 6 (S1) |
|  | 10b | List and define all other variables for which data were sought (e.g. participant and intervention characteristics, funding sources). Describe any assumptions made about any missing or unclear information. | Lines 115 -118 Also Appendix 6 (S1) |
| Study risk of bias assessment | 11 | Specify the methods used to assess risk of bias in the included studies, including details of the tool(s) used, how many reviewers assessed each study and whether they worked independently, and if applicable, details of automation tools used in the process. | Line 121-132 Also see details in Appendix 5 & 7 |
| Effect measures | 12 | Specify for each outcome the effect measure(s) (e.g. risk ratio, mean difference) used in the synthesis or presentation of results. | Line 134-141 for meta-analysis, 144-152 for meta-regression.  Also see details in Appendix 6 (S1) |
| Synthesis methods | 13a | Describe the processes used to decide which studies were eligible for each synthesis (e.g. tabulating the study intervention characteristics and comparing against the planned groups for each synthesis (item #5)). | Details in Appendix 6 |
|  | 13b | Describe any methods required to prepare the data for presentation or synthesis, such as handling of missing summary statistics, or data conversions. | Details in Appendix 6 |
|  | 13c | Describe any methods used to tabulate or visually display results of individual studies and syntheses. | Details in Appendix 6 |
|  | 13d | Describe any methods used to synthesize results and provide a rationale for the choice(s). If meta-analysis was performed, describe the model(s), method(s) to identify the presence and extent of statistical heterogeneity, and software package(s) used. | Detailed in Appendix 6 |
|  | 13e | Describe any methods used to explore possible causes of heterogeneity among study results (e.g. subgroup analysis, meta-regression). | Detailed in Appendix 7 |
|  | 13f | Describe any sensitivity analyses conducted to assess robustness of the synthesized results. | Detailed in Appendix 7 |
| Reporting bias assessment | 14 | Describe any methods used to assess risk of bias due to missing results in a synthesis (arising from reporting biases). | Detailed in Appendix 7 |
| Certainty assessment | 15 | Describe any methods used to assess certainty (or confidence) in the body of evidence for an outcome. | Appendix 6 and Appendix 7 |
| **RESULTS** | | |  |
| Study selection | 16a | Describe the results of the search and selection process, from the number of records identified in the search to the number of studies included in the review, ideally using a flow diagram. | Figure 1 |
|  | 16b | Cite studies that might appear to meet the inclusion criteria, but which were excluded, and explain why they were excluded. | Figure 1, footnote a. |
| Study characteristics | 17 | Cite each included study and present its characteristics. | Table 3, Table 4, Appendix 8 |
| Risk of bias in studies | 18 | Present assessments of risk of bias for each included study. | Appendix 8, 10 and 11 |
| Results of individual studies | 19 | For all outcomes, present, for each study: (a) summary statistics for each group (where appropriate) and (b) an effect estimate and its precision (e.g. confidence/credible interval), ideally using structured tables or plots. | Appendix 8, 10 and 11 |
| Results of syntheses | 20a | For each synthesis, briefly summarise the characteristics and risk of bias among contributing studies. | Appendix 8, 10 and 11 |
|  | 20b | Present results of all statistical syntheses conducted. If meta-analysis was done, present for each the summary estimate and its precision (e.g. confidence/credible interval) and measures of statistical heterogeneity. If comparing groups, describe the direction of the effect. | Appendix 8, 10 and 11 |
|  | 20c | Present results of all investigations of possible causes of heterogeneity among study results. | Appendix 8, 10 and 11 |
|  | 20d | Present results of all sensitivity analyses conducted to assess the robustness of the synthesized results. | Appendix 8, 10 and 11 |
| Reporting biases | 21 | Present assessments of risk of bias due to missing results (arising from reporting biases) for each synthesis assessed. | Appendix 8, 10 and 11 |
| Certainty of evidence | 22 | Present assessments of certainty (or confidence) in the body of evidence for each outcome assessed. | Appendix 8, 10 and 11 |
| **DISCUSSION** | | |  |
| Discussion | 23a | Provide a general interpretation of the results in the context of other evidence. | Line 242-343 |
|  | 23b | Discuss any limitations of the evidence included in the review. | Line 346-362 |
|  | 23c | Discuss any limitations of the review processes used. | Line 346-362 |
|  | 23d | Discuss implications of the results for practice, policy, and future research. | Line 365-373 |
| **OTHER INFORMATION** | | |  |
| Registration and protocol | 24a | Provide registration information for the review, including register name and registration number, or state that the review was not registered. | The review was not registered |
|  | 24b | Indicate where the review protocol can be accessed, or state that a protocol was not prepared. | A protocol was not prepared |
|  | 24c | Describe and explain any amendments to information provided at registration or in the protocol. | N/A |
| Support | 25 | Describe sources of financial or non-financial support for the review, and the role of the funders or sponsors in the review. | Line 639 |
| Competing interests | 26 | Declare any competing interests of review authors. | Line 636 |
| Availability of data, code and other materials | 27 | Report which of the following are publicly available and where they can be found: template data collection forms; data extracted from included studies; data used for all analyses; analytic code; any other materials used in the review. | Line 386-387 |

S = Supplementary material

# **Appendix 2.** Detailed search strategy

## **Databases**

**1. Database: Pubmed; Date of search: 12-01-2021**

Search: (((((("CHARGE Syndrome*") OR "CHARGE Association*") OR (((((Coloboma) AND Heart anomaly) AND Choanal Atresia) AND Genital Anomalies) AND Ear Anomalies)) OR "Hall-Hittner Syndrome*") OR CHD7) OR SEMA3A)

("charge syndrome*"[All Fields] OR "charge association*"[All Fields] OR (("coloboma"[MeSH Terms] OR "coloboma"[All Fields] OR "colobomas"[All Fields]) AND ("heart defects, congenital"[MeSH Terms] OR ("heart"[All Fields] AND "defects"[All Fields] AND "congenital"[All Fields]) OR "congenital heart defects"[All Fields] OR ("heart"[All Fields] AND "anomaly"[All Fields]) OR "heart anomaly"[All Fields]) AND ("choanal atresia"[MeSH Terms] OR ("choanal"[All Fields] AND "atresia"[All Fields]) OR "choanal atresia"[All Fields]) AND (("genitalia"[MeSH Terms] OR "genitalia"[All Fields] OR "genital"[All Fields] OR "genitals"[All Fields] OR "genitally"[All Fields]) AND ("abnormalities"[MeSH Subheading] OR "abnormalities"[All Fields] OR "anomalies"[All Fields] OR "anomalie"[All Fields] OR "anomaly"[All Fields])) AND (("ear"[MeSH Terms] OR "ear"[All Fields]) AND ("abnormalities"[MeSH Subheading] OR "abnormalities"[All Fields] OR "anomalies"[All Fields] OR "anomalie"[All Fields] OR "anomaly"[All Fields]))) OR "hall hittner syndrome*"[All Fields] OR "CHD7"[All Fields] OR ("semaphorin 3a"[MeSH Terms] OR "semaphorin 3a"[All Fields] OR "sema3a"[All Fields]))

**Translations**

- Coloboma: "coloboma"[MeSH Terms] OR "coloboma"[All Fields] OR "colobomas"[All Fields]
- Heart anomaly: "heart defects, congenital"[MeSH Terms] OR ("heart"[All Fields] AND "defects"[All Fields] AND "congenital"[All Fields]) OR "congenital heart defects"[All Fields] OR ("heart"[All Fields] AND "anomaly"[All Fields]) OR "heart anomaly"[All Fields]
- Choanal Atresia: "choanal atresia"[MeSH Terms] OR ("choanal"[All Fields] AND "atresia"[All Fields]) OR "choanal atresia"[All Fields]
- Genital: "genitalia"[MeSH Terms] OR "genitalia"[All Fields] OR "genital"[All Fields] OR "genitals"[All Fields] OR "genitally"[All Fields]
- Anomalies: "abnormalities"[Subheading] OR "abnormalities"[All Fields] OR "anomalies"[All Fields] OR "anomalie"[All Fields] OR "anomaly"[All Fields]
- Ear: "ear"[MeSH Terms] OR "ear"[All Fields]
- Anomalies: “abnormalities”[Subheading] OR “abnormalities”[All Fields] OR “anomalies”[All Fields] OR “anomalie”[All Fields] OR “anomaly”[All Fields]
- SEMA3A: "semaphorin-3a"[MeSH Terms] OR "semaphorin-3a"[All Fields] OR "sema3a"[All Fields]

Sort by: Best Match

**Number of hits: 2,346**

**Publication dates: no restrictions**

**2. Database: Embase, through Ovid^®^; Date of search: 12-01-2021**

Search: ("CHARGE Syndrome*" or "CHARGE Association*" or (Coloboma and Heart anomaly and Choanal Atresia and Genital Anomalies and Ear Anomalies) or "Hall-Hittner Syndrome*" or CHD7 or SEMA3A).af

**Number of hits: 2,641**

**Publication dates: no restrictions**

**3. Database: Medline, through Ovid^®^; Date of search: 12-01-2021**

Search: ("CHARGE Syndrome*" or "CHARGE Association*" or (Coloboma and Heart anomaly and Choanal Atresia and Genital Anomalies and Ear Anomalies) or "Hall-Hittner Syndrome*" or CHD7 or SEMA3A).af

**Number of hits: 1,936**

**Publication dates: no restrictions**

**4. Database: PsycInfo, through Ovid^®^; Date of search: 12-01-2021**

Search: ("CHARGE Syndrome*" or "CHARGE Association*" or (Coloboma and Heart anomaly and Choanal Atresia and Genital Anomalies and Ear Anomalies) or "Hall-Hittner Syndrome*" or CHD7 or SEMA3A).af

**Number of hits: 838**

**Publication dates: no restrictions**

# **Appendix 3.** Reference list of studies identified through manual searches

## **Knowledge base searches:**

**1. GeneReviews®:**

van Ravenswaaij-Arts CMA, Hefner M, Blake K, Martin DM. CHD7 Disorder - GeneReviews® - NCBI Bookshelf. 2020;:1–32. <https://www.ncbi.nlm.nih.gov/sites/books/NBK1117/> Accessed 12 Jan 2021.

Reference list:

Andersen SL, Lönn S, Vestergaard P, Törring O. Birth defects after use of antithyroid drugs in early pregnancy: A Swedish nationwide study. Eur J Endocrinol. 2017;177:369–378. <https://doi.org/10.1530/EJE-17-0314>

Andersen SL, Olsen J, Wu CS, Laurberg P. Birth defects after early pregnancy use of antithyroid drugs: a Danish nationwide study. J Clin Endocrinol. Metab. 2013;98:4373–4381. <https://doi.org/https://doi.org/10.1210/jc.2013-2831>

Komoike Y, Matsuoka M, Kosaki K. Potential teratogenicity of methimazole: Exposure of zebrafish embryos to methimazole causes similar developmental anomalies to human methimazole embryopathy. Birth Defects Res Part B Dev Reprod Toxicol 2013;98:222–229. <https://doi.org/10.1002/bdrb.21057>

Lammer EJ, Chen DT, Hoar RM, Agnish ND, Benke PJ. Braun JT, et al. Retinoic acid embryopathy. N Engl J Med. 1985. <https://doi.org/10.1056/NEJM198510033131401>

Richards S, Aziz N, Bale S, Bick D, Das S, Gastier-Foster J, et al. Standards and guidelines for the interpretation of sequence variants: A joint consensus recommendation of the American College of Medical Genetics and Genomics and the Association for Molecular Pathology. Genet Med. 2015;17:405–424. <https://doi.org/10.1038/gim.2015.30>

**2. UniProtKB:Entry Q9P2D1 (CHD7_HUMAN)**

**The UniProt Consortium. CHD7 - Chromodomain-helicase-DNA-binding protein 7 - Homo sapiens (Human) - CHD7 gene & protein. 2021. UniProtKB/Swiss-Prot.** [**https://www.uniprot.org/uniprot/Q9P2D1**](https://www.uniprot.org/uniprot/Q9P2D1) **Accessed 12 Jan 2021.**

**Reference list:**

Ota T, Suzuki Y, Nishikawa T, Otsuki T, Sugiyama T. Irie R, et al. 2004. Complete sequencing and characterization of 21,243 full-length human cDNAs. Nat Genet. 2004:36;40–45.

##

## **Review Articles**

**1.** **Hsu P, Ma A, Wilson M, Williams G, Curotta J, Munns CF, et al. CHARGE syndrome: A review. J Paediatr Child Health. 2014;50:504-511** [**https://doi.org/10.1111/jpc.12497**](https://doi.org/10.1111/jpc.12497)**.**

**Reference list:**

Houben CH, Curry JI. Current status of prenatal diagnosis, operative management and outcome of esophageal atresia/tracheo-esophageal fistula. Prenat Diagn. 2008:7;667-675. <https://doi.org/10.1002/pd.1938>

Khadilkar VV, Cameron FJ, Stanhope R. Growth failure and pituitary function in CHARGE and VATER associations. Arch Dis Child. 1999;80:167–170. <https://doi.org/10.1136/adc.80.2.167>

Markert ML, Devlin BH, Alexieff MJ, Li J, McCarthy EA, Gupton SE, et al. Review of 54 patients with complete DiGeorge anomaly enrolled in protocols for thymus transplantation: Outcome of 44 consecutive transplants. Blood 2007;109:4539–4547. <https://doi.org/10.1182/blood-2006-10-048652>

**2. Trider CL, Arra-Robar A, van Ravenswaaij-Arts C, Blake K. Developing a CHARGE syndrome checklist: Health supervision across the lifespan (from head to toe). Am J Med Genet Part A 2017;173:684–691.** [**https://doi.org/10.1002/ajmg.a.38085**](https://doi.org/10.1002/ajmg.a.38085)

**Reference list:**

Gasalberti D. Alternative therapies for children and youth with special health care needs. J Pediatr Heal Care. 2006;20:133–136. <https://doi.org/10.1016/j.pedhc.2005.12.015>

Quint EH. Adolescents with Special Needs: Clinical Challenges in Reproductive Health Care. J. Pediatr. Adolesc. Gynecol. 2016;29:2–6. <https://doi.org/10.1016/j.jpag.2015.05.003>

**3.** **van Ravenswaaij-Arts C, Martin DM. New insights and advances in CHARGE syndrome: Diagnosis, etiologies, treatments, and research discoveries. Am J Med Genet Part C Semin Med Genet. 2017;175:397-406** [**https://doi.org/10.1002/ajmg.c.31592**](https://doi.org/10.1002/ajmg.c.31592)

**Reference list:**

Alavizadeh A, Kiernan A, Nolan P, Lo C, Steel KP, Bucan M. The Wheels mutation in the mouse causes vascular, hindbrain, and inner ear defects. Dev Biol. 2001;234:244–260. <https://doi.org/doi:10.1006/dbio.2001.0241>

Angelman H. Syndrome of coloboma with multiple congenital abnormalities in infancy. Br Med J. 1961;1:1212–1214. <https://doi.org/10.1136/bmj.1.5234.1212>

Bustamante-Aragonés A, Rodríguez de Alba M, Perlado S, Trujillo-Tiebas MJ, Arranz JP, Díaz-Recasens J, Troyano-Luque J, et al. Non-invasive prenatal diagnosis of single-gene disorders from maternal blood. Gene 2012;504:144–149. <https://doi.org/10.1016/j.gene.2012.04.045>

Daley R, Hill M, Chitty LS. Non-invasive prenatal diagnosis: Progress and potential. Arch Dis Child Fetal Neonatal Ed. 2014;99:F426–F430. <https://doi.org/10.1136/archdischild-2013-304828>

Daubresse G, Deuring R, Moore L, Papoulas O, Zakrajsek I, Waldrip WR, et al. The Drosophila kismet gene is related to chromatin-remodeling factors and is required for both segmentation and segment identity. Development. 1999;126:1175–1187.

de Ligt J, Boone PM, Pfundt R, Vissers LELM, de Leeuw N, Shaw C, et al. Platform comparison of detecting copy number variants with microarrays and whole-exome sequencing. Genomics Data. 2014;2:144–146. <https://doi.org/10.1016/j.gdata.2014.06.009>

Dorighi KM, Tamkun JW. The trithorax group proteins Kismet and ASH1 promote H3K36 dimethylation to counteract Polycomb group repression in Drosophila. Dev. 2013;140:4182–4192. <https://doi.org/10.1242/dev.095786>

Flanagan JF, Blus BJ, Kim D, Clines KL, Rastinejad F, Khorasanizadeh S. Molecular Implications of Evolutionary Differences in CHD Double Chromodomains. J Mol Biol. 2007;369:334–342. <https://doi.org/10.1016/j.jmb.2007.03.024>

Hawker K, Fuchs H, Angelis MH, Steel KP, 2005. Two new mouse mutants with vestibular defects that map to the highly mutable locus on chromosome 4. Int J Audiol. 2005;44:171–177. <https://doi.org/10.1080/14992020500057434>

Kiernan AE, Erven A, Voegeling S, Peters J, Nolan P, Hunter J, et al. ENU mutagenesis reveals a highly mutable locus on mouse Chromosome 4 that affects ear morphogenesis. Mamm Genome. 2002;13:142–148. <https://doi.org/10.1007/bf02684018>

Nolan PM, Sollars PJ, Bohne BA, Ewens WJ, Pickard GE, Bucan M. Heterozygosity mapping of partially congenic lines: Mapping of a semidominant neurological mutation, Wheels (Whl), on mouse chromosome 4. Genetics. 1995;140: 245–254. <https://doi.org/10.1093/genetics/140.1.245>

Pau H, Hawker K, Fuchs H, De Angelis MH, Steel KP. Characterization of a new mouse mutant, flouncer, with a balance defect and inner ear malformation. Otol Neurotol. 2004;25:707–713. <https://doi.org/10.1097/00129492-200409000-00010>

Pickard GE, Sollars PJ, Rinchik EM, Nolan PM, Bucan M. Mutagenesis and behavioral screening for altered circadian activity identifies the mouse mutant, Wheels. Brain Res. 1995;705:255–266. <https://doi.org/10.1016/0006-8993(95)01171-4>

Srinivasan S, Armstrong JA, Deuring R, Dahlsveen IK, McNeill H, Tamkun JW. The Drosophila trithorax group protein kismet facilitates an early step in transcriptional elongation by RNA polymerase II. Development. 2005;132;1623–1635. <https://doi.org/10.1242/dev.01713>

Talkowski ME, Rosenfeld JA, Blumenthal I, Pillalamarri V, Chiang C, Heilbut A, et al. Sequencing chromosomal abnormalities reveals neurodevelopmental loci that confer risk across diagnostic boundaries. Cell. 2012;149:525–537. <https://doi.org/https://doi.org/10.1016/j.cell.2012.03.028>

Terriente-Félix A, Molnar C, Gómez-Skarmeta Jose Luis JL, De Celis JF. A conserved function of the chromatin ATPase Kismet in the regulation of hedgehog expression. Dev Biol. 2011;350:382–392. <https://doi.org/10.1016/j.ydbio.2010.12.003>

Woodage T, Basrai MA, Baxevanis AD, Hieter P, Collins FS. Characterization of the CHD family of proteins. Proc Natl Acad Sci U S A 1997;94:11472–11477. <https://doi.org/10.1073/pnas.94.21.11472>

Zentner GE, Scacheri PC. The chromatin fingerprint of gene enhancer elements. J Biol Chem. 2012;287:30888–30896. https://doi.org/10.1074/jbc.R111.296491

# **Appendix 4.** Inclusion and exclusion criteria used for study selection

##### **Table A2.** Inclusion and exclusion criteria used for study selection

| **Inclusion Criteria** | **Exclusion criteria** |
| --- | --- |
| **Title and abstract screening** |  |
| Articles published or available in English | Main text (excluding abstract) in only available in a non-English language. |
| An original empirical study with human participants | Review articles, review chapters or published conference abstracts. Animal studies or genetics papers without person details |
| Includes at least one of the search terms in the title, abstract or key words, or reports data from participants with a sensory or neurodevelopmental disorder | Not relevant to CHARGE syndrome (e.g. without any of the OMIM terms for CHARGE syndrome in the title abstract or key words) and does not include data on individuals with sensory or neurodevelopmental disorder. (e.g. impairment of one or more of the five senses, or a neurodevelopmental disorder as specified in the DSM-5 [1] |
| **Full text screening** |  |
| Articles reporting data drawn from study participants with a diagnosis of CHARGE syndrome | Articles reporting no novel data about CHARGE syndrome or reporting on a sample of participants with do not include any individuals with a diagnosis of CHARGE syndrome |
| Article reports participant level data about behavioural, psychological, cognitive or sleep characteristics from participants with CHARGE syndrome | Article does not report data about behavioural, psychological, cognitive or sleep characteristics in CHARGE syndrome, or reports data at summary level only (e.g., mean scores) |
| Reports on an unbiased participant population | Participants were recruited to the study based on the presence of a specific characteristic that is not (or was not) understood to be ubiquitous in CHARGE syndrome. Article is a case study, case series or cohort studies with less than five participants with CHARGE syndrome |
| At least five participants have a clinical diagnosis of CHARGE syndrome, partial CHARGE syndrome or atypical CHARGE syndrome (see Table 1). | Less than five participants from the study sample meet clinical diagnosis for CHARGE syndrome. Participants were diagnosed on the basis of a CHD7 mutation, and it is unclear if participants reach clinical criteria for diagnosis. |

1. American Psychiatric Association. Diagnostic and Statistical Mental Disorders DSM-5. 5th edition. Arlington, VA: American Psychiatric Publishing; 2013.

# **Appendix 5.** Quality appraisal

Following data extraction, an appropriate quality framework was developed to control for the risk of methodological bias between the individual studies included in the meta-analysis. The framework used was adapted from those previously used in meta-analyses of rare syndromes and intellectual disability by Richards et al. [1] and Surtees et al. [2]. Inter-rater reliability was good to excellent for the original versions (r(52) = 0.78, p < 0.001; Richards et al. [1]: α = .94; Surtees et al. [2]. For sample identification, confirmation of syndrome and quality of autism assessment, the framework provided independent quality scores (from poor, weighted 0, to excellent, weighted 3). Criteria were developed for studies reporting on the prevalence of autism in genetic syndromes, and therefore required adaptation for the present study. Confirmation of syndrome was adapted to reflect the ambiguity of CHD7 positive diagnosis in the absence of clinical CHARGE characteristics. The original quality weighting framework for the assessment of autism [1] was generalised to include assessment of behavioural and psychological characteristics. A new quality weighting framework was developed for the assessment of cognitive characteristics. A quality weighting for sleep assessment was also included based on Surtees et al. [2].

The adapted quality criteria are presented in Table 2.3. In summary, papers including participants whose clinical and genetic status were confirmed and reported in detail at the time of the study were weighted higher than those using clinical or unspecified clinical criteria alone. Sampling method was rated highest for random or total population studies, with lower scores awarded to single or multiple restricted or non-random samples. Given the questionable validity of many standardised instruments for individuals with sensory impairments [3], assessment of behaviour, psychological and cognition using standardised tools, supported by additional assessment were given the highest scores. Standardised and non-standardised informant reports were awarded scores of two and one respectively. Studies reporting incomplete measurement of sleep, for example a single question, were weighted lower than papers using standardised questionnaires. Studies using direct sleep assessments received a score of three for observational, or four for objective measurement. Good inter-rater reliability was ascertained by a second researcher (weighted Kappa 85% [4]) using a 25% random sample of the eligible studies.

**References:**

1. Richards C, Jones C, Groves L, Moss J, Oliver C. Prevalence of autism spectrum disorder phenomenology in genetic disorders: A systematic review and meta-analysis. The Lancet Psychiatry. 2015;2:909–16. doi:10.1016/S2215-0366(15)00376-4.

2. Surtees ADR, Oliver C, Jones CA, Evans DL, Richards C. Sleep duration and sleep quality in people with and without intellectual disability: A meta-analysis. Sleep Medicine Reviews. 2018;40:135–50. doi:10.1016/j.smrv.2017.11.003.

3. Rowland C, Fried-Oken M. Communication Matrix: A clinical and research assessment tool targeting children with severe communication disorders. J Pediatr Rehabil Med. 2010;3:319–29. doi:10.3233/PRM-2010-0144.

4. Cohen J. A Coefficient of Agreement for Nominal Scales. Educ Psychol Meas. 1960;20:37–46.

# **Appendix 6.** Sensitivity assessments

##

## **The Funnel Plot**

The funnel plot is used to detect systematic heterogeneity resulting from small study bias. The funnel plot is a scatter plot of standard error by prevalence estimates for each of the studies included in the meta-analysis. Where bias is not present, studies with high precision are plotted near the centre (representing the meta-analytic synthesis) and studies with less precision present in equal numbers on either side with data following a symmetric inverted funnel shape. Visual inspection of the funnel plot is supplemented by Egger’s regression test for which the standard error is regressed against the prevalence estimate [1].

## **The Trim and Fill procedure [2]**

The trim and fill method [2] is designed to address the effect of deviations from the symmetric inverted funnel shape plot. This procedure uses an iterative algorithm to remove outlying effect size values identified on the side of the funnel plot that indicates positive effects. The algorithm then adds the original studies back into the analysis and imputes a mirror image for each on the side associated with negative effects to retain symmetry.

## **The fail-safe *N* [3]**

The fail-safe *N* [3] provides an estimation of how many zero effect size studies could be added to the omnibus test before the associated p-value is no longer significant. If the fail-safe *N* is greater than the number of effects included than the omnibus test can be considered robust to publication bias.

## **Baujat Plot [4]**

The Baujat plot [4] is a graphical tool for detecting outliers and sources of residual heterogeneity in meta-analysis. The plot shows the contribution of each study to the *Q*-statistic (the squared Pearson residual for each study) on the x-axis versus the influence of each study (defined as the standardised squared difference between the overall study estimate with and without the study included in the model fitting) on the y-axis. Hence, studies in the top right have a greater influence on the overall results and a greater contribution to heterogeneity.

## **Leave one out procedure**

The leave one out procedure is the process of iteratively omitting one study at a time from the meta-analysis and recalculating the omnibus test. Results can then be evaluated to assess whether the pooled prevalence estimate is driven by the effect of any single study.

**Sub-group analysis**

Where ≥ 2 studies could be included in each group, studies were stratified by *Poor* or *Adequate* quality and *Good* or *Excellent* quality with separate analyses for sample identification, confirmation of syndrome and quality of assessment. Subgroup analysis was evaluated by comparison of 95% CIs. Findings were reported using the Q and p-value (α = 0.05).

**References**

1. Egger M, Smith GD, Schneider M, Minder C. Bias in meta-analysis detected by a simple, graphical test. Br Med J. 1997;315:629–34.

2. Duval S, Tweedie R. Trim and fill: A simple funnel-plot-based method of testing and adjusting for publication bias in meta-analysis. Biometrics. 2000;56:455–63.

3. Rosenthal R. The file drawer problem and tolerance for null results. Psychol Bull. 1979;86:638–41. doi:10.1037/0033-2909.86.3.638.

4. Baujat B, Mahé C, Pignon JP, Hill C. A graphical method for exploring heterogeneity in meta-analyses: Application to a meta-analysis of 65 trials. Stat Med. 2002;21:2641-2652.

# **Appendix 7.** Forest plots and sensitivity analysis for ~~diagnostic signs~~ clinical features

## **Coloboma**

###### **Figure A7.1** QQ Plot of The Distribution of Study Effects and Theoretical Quantities Based on A Normal Distribution Under the Random Effects Model for Studies Reporting Coloboma

**Note:** Visual inspection of the QQ plot suggests an approximate normal distribution of study effects for the 31 studies reporting coloboma in CHARGE Syndrome. On this basis the DerSimonian-Laird estimate was used to calculate between studies variance in the random-effects model

###### **Figure A7.2** Random Effects Models of The Pooled Prevalence Estimate for Coloboma in CHARGE Syndrome

**Note:** The pooled prevalence estimate for coloboma in CHARGE syndrome is 83% (95% CI, 74-86%; permuted *p-*value = 0.001; *k* = 31) with moderate heterogeneity (I^2^ = 58%). Random-effects model calculated using the inverse variance method and the DerSimonian-Laird estimator for τ^2^. Rosenthal Fail-safe N = 64553 suggests that the observed effect is robust to potential publication biases.

| **Figure A7.3** Funnel Plot of Standard Error by Prevalence of Coloboma Following the Trim and Fill Procedure | **Figure A7.4** Baujat Plot of Contribution to Heterogeneity by Influence on Overall Effect for Studies Reporting Coloboma |
| --- | --- |
|  |  |
| **Note:** Publication bias [small study effect] was identified (Egger’s test p = 0.011). Using the trim and fill procedure it was estimated that 6 (SE = 3.71) studies were missing on the right side. Adjusted estimate = 84%, (95% *CI* = 81-89%, *p* = <.001; τ^2^ = 0.076, I^2^ = 61%) | **Note:** Studies in the top right quartile have the greatest contribution to overall heterogeneity and the greatest influence on the overall effect. Legendre et al. (2017) had the greatest contribution to overall heterogeneity and Shoji et al. (2014) had the greatest influence on the overall effect |

###### **Figure A7.5** Leave-One-Out Random Effects Model for Studies Reporting Coloboma


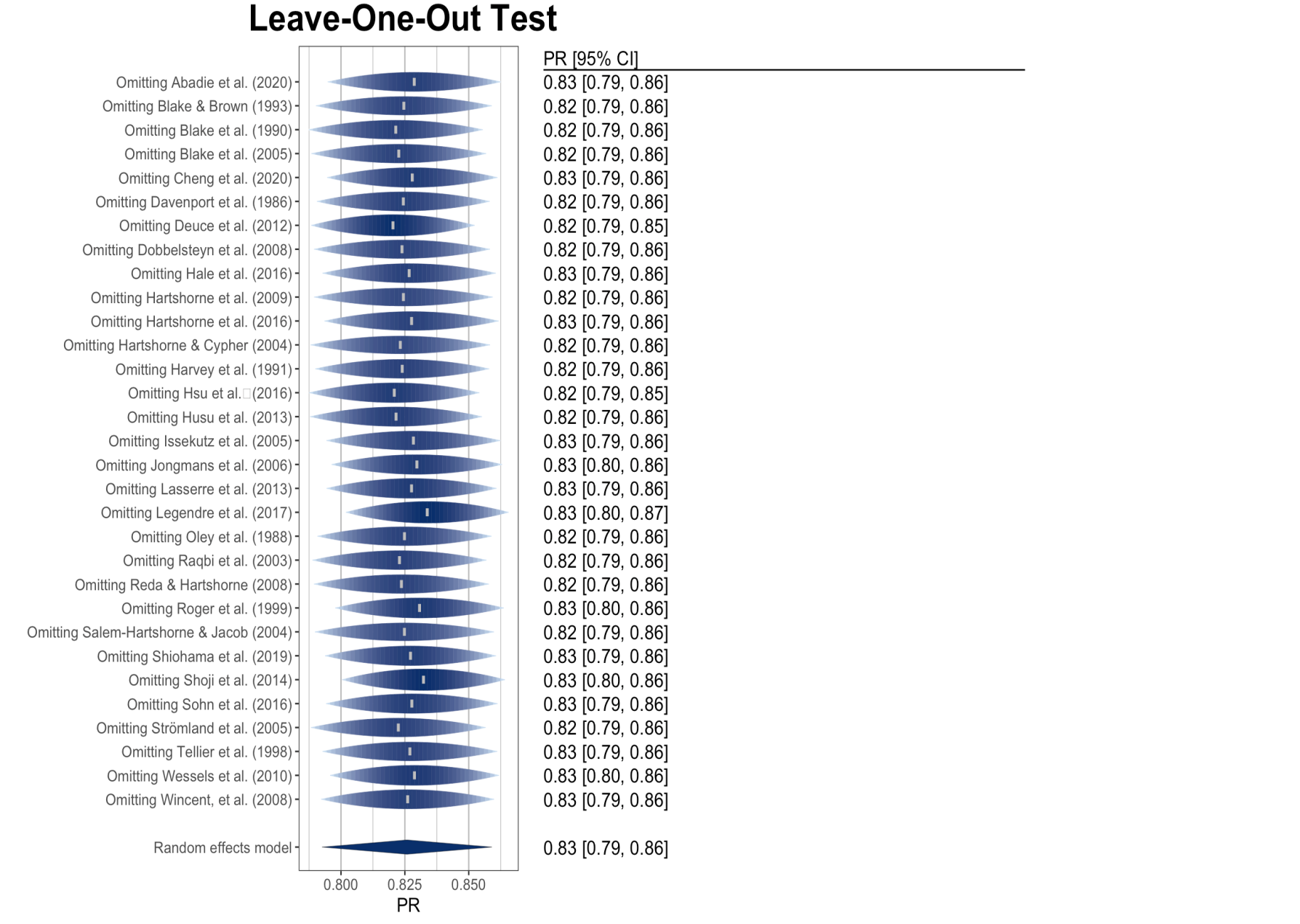


**Note:** Leave one out analysis indicating that no single study is exerting a disproportional influence on the pooled prevalence estimate

## **Choanal Atresia**

###### **Figure A7.6** QQ Plot of The Distribution of Study Effects and Theoretical Quantities Based on A Normal Distribution Under the Random Effects Model for Studies Reporting Choanal Atresia

**Note:** Visual inspection of the *QQ* plot suggests an approximate normal distribution of study effects for the 30 studies reporting choanal atresia in CHARGE Syndrome. On this basis the DerSimonian-Laird estimate was used to calculate between studies variance in the random-effects model

###### **Figure A7.7** Random Effects Models of The Pooled Prevalence Estimate for Choanal Atresia in CHARGE Syndrome

**Note:** The pooled prevalence estimate for choanal atresia in CHARGE syndrome is 51% (95% CI, 45-57%; permuted *p-*value = 0.001; *k* = 30) with high heterogeneity (I^2^ = 77%). Random-effects model calculated using the inverse variance method and the DerSimonian-Laird estimator for τ^2^. Rosenthal Fail-safe N = 14252 suggests that the observed effect is robust to potential publication biases.

| **Figure A7.8** Funnel Plot of Standard Error by Prevalence of Choanal Atresia | **Figure A7.9** Baujat Plot of Contribution to Heterogeneity by Influence on Overall Effect for Studies Reporting Choanal Atresia |
| --- | --- |
|  |  |
| **Note:** Visual inspection of the funnel plot conforms to normal expectations and there is weak evidence of substantial publication bias (Egger’s test p = 0.528) | **Note:** Shoji et al. (2014) had the greatest contribution to overall heterogeneity and the greatest influence on the overall effect. |

###### **Figure A7.10** Leave-One-Out Random Effects Model for Studies Reporting Choanal Atresia


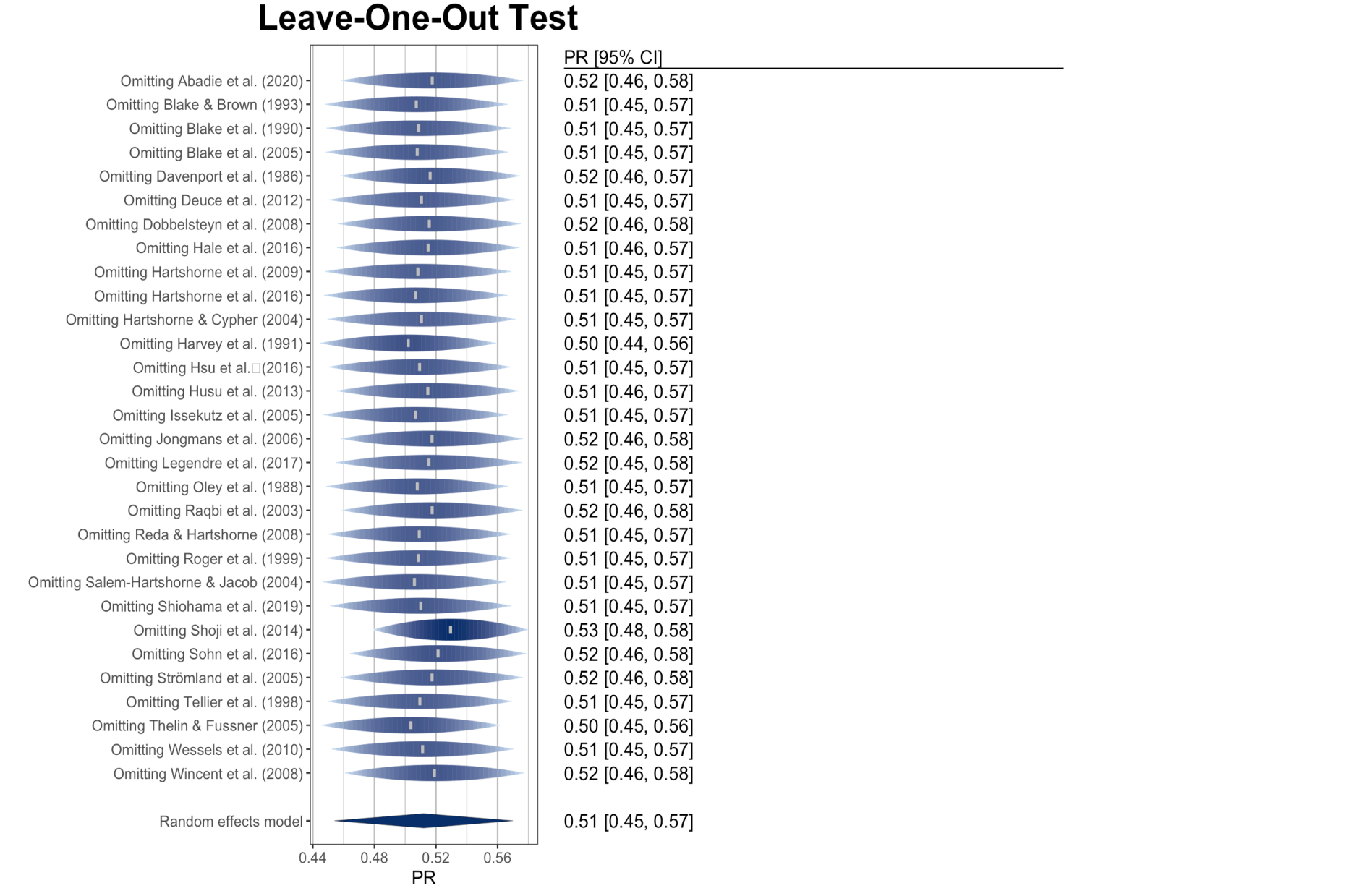


**Note:** Leave one out analysis indicating that no single study is exerting a disproportional influence on the pooled prevalence estimate000

## **Anosmia**

###### **Figure A7.11** QQ Plot of The Distribution of Study Effects and Theoretical Quantities Based on A Normal Distribution Under the Random Effects Model for Studies Reporting Anosmia

**Note:** Visual inspection of the *QQ* plot suggests an approximate normal distribution of study effects for the 11 studies reporting Anosmia in CHARGE Syndrome. On this basis the DerSimonian-Laird estimate was used to calculate between studies variance in the random-effects model.

###### **Figure A7.12** Random Effects Models of The Pooled Prevalence Estimate for Anosmia in CHARGE Syndrome

**Note:** The pooled prevalence estimate for anosmia in CHARGE syndrome is 39% (95% CI, 24-54%; permuted *p-*value = 0.002; *k* = 11) with high heterogeneity (I^2^ = 87%). Random-effects model calculated using the inverse variance method and the DerSimonian-Laird estimator for τ^2^. Rosenthal Fail-safe N = 734 suggests that the observed effect is robust to potential publication biases.

| **Figure A7.13** Funnel Plot of Standard Error by Prevalence of Anosmia | **Figure A7.14** Baujat Plot of Contribution to Heterogeneity by Influence on Overall Effect for Studies Reporting Anosmia |
| --- | --- |
|  |  |
| **Note:** Visual inspection of the funnel plot conforms to normal expectations and there is weak evidence of substantial publication bias (Egger’s test p = 0.741) | **Note:** Legendre et al. (2017) had the greatest contribution to overall heterogeneity and the greatest influence on the overall effect. |

###### **Figure A7.15** Leave-One-Out Random Effects Model for Studies Reporting Anosmia

**Note:** Leave one out analysis indicating that no single study is exerting a disproportional influence on the pooled prevalence estimate

## **Facial Palsy**

###### **Figure A7.16** QQ Plot of The Distribution of Study Effects and Theoretical Quantities Based on A Normal Distribution Under the Random Effects Model for Studies Reporting Facial Palsy

**Note:** Visual inspection of the *QQ* plot suggests an approximate normal distribution of study effects for the 23 studies reporting facial palsy in CHARGE Syndrome. On this basis the DerSimonian-Laird estimate was used to calculate between studies variance in the random-effects model.

###### **Figure A7.17** Random Effects Models of The Pooled Prevalence Estimate for Facial Palsy in CHARGE Syndrome

**Note:** The pooled prevalence estimate for facial palsy in CHARGE syndrome is 46% (95% CI, 39-53%; permuted *p-*value = 0.001; *k* = 23) with high heterogeneity (I^2^ = 77%). Random-effects model calculated using the inverse variance method and the DerSimonian-Laird estimator for τ2. Rosenthal Fail-safe N = 6435 suggests that the observed effect is robust to potential publication biases.

| **Figure A7.18** Funnel Plot of Standard Error by Prevalence of Facial Palsy | **Figure A7.19** Baujat Plot of Contribution to Heterogeneity by Influence on Overall Effect for Studies Reporting Facial Palsy |
| --- | --- |
|  |  |
| **Note:** Visual inspection of the funnel plot conforms to normal expectations and there is weak evidence of substantial publication bias (Egger’s test p = 0.655) | **Note:** Studies in the top right quartile have the greatest contribution to overall heterogeneity and the greatest influence on the overall effect. Legendre et al. (2017) had the greatest contribution to overall heterogeneity. Sohn et al. (2016) had the greatest influence on the overall effect. |

###### **Figure A7.20** Leave-One-Out Random Effects Model for Studies Reporting Facial Palsy


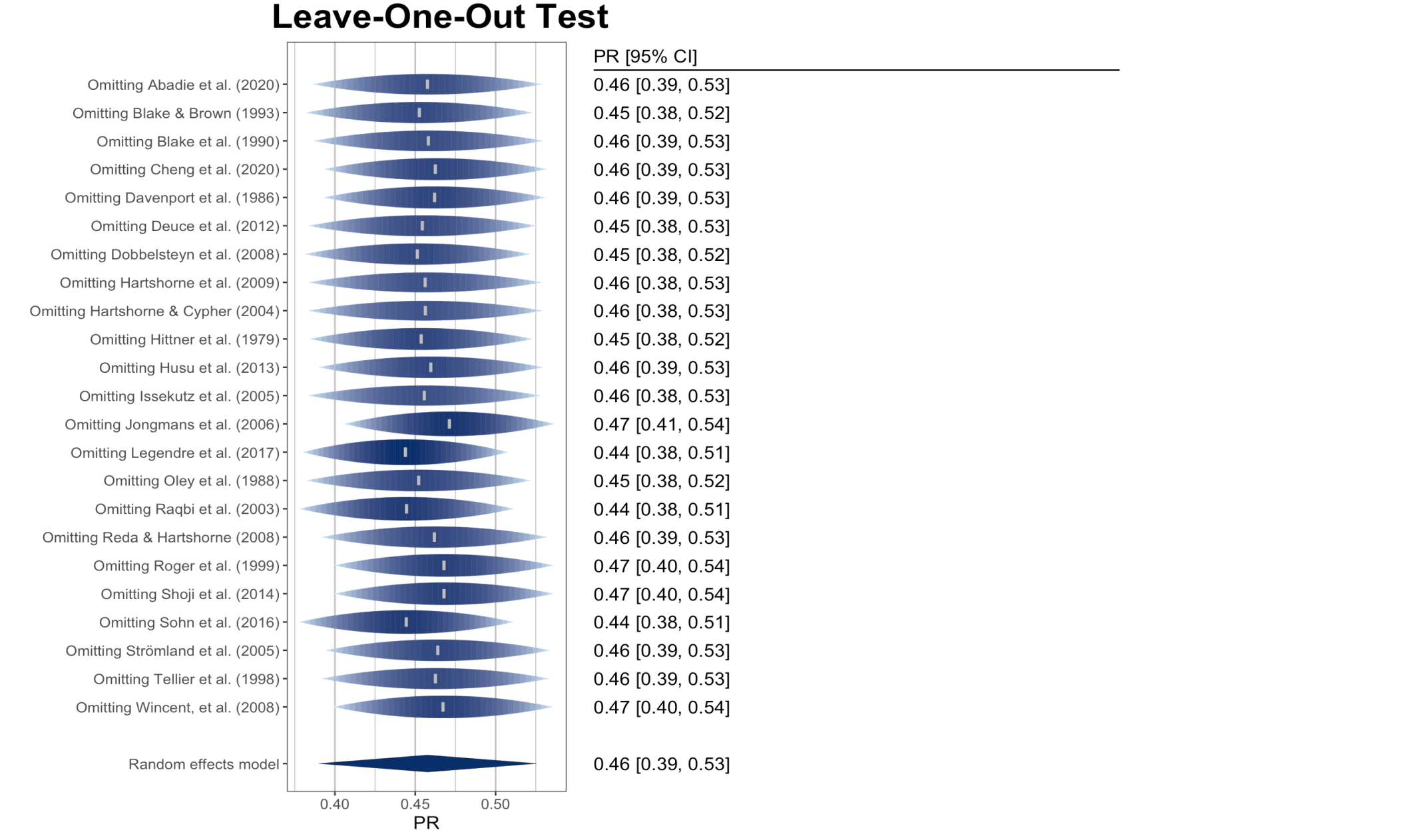


**Note:** Leave one out analysis indicating that no single study is exerting a disproportional influence on the pooled prevalence estimate

## **Feeding and Swallowing Difficulties**

###### **Figure A7.21** QQ Plot of The Distribution of Study Effects and Theoretical Quantities Based on A Normal Distribution Under the Random Effects Model for Studies Reporting Feeding and Swallowing Difficulties

**Note:** Visual inspection of the *QQ* plot suggests an approximate normal distribution of study effects for the 21 studies reporting feeding and swallowing difficulties in CHARGE Syndrome. On this basis the DerSimonian-Laird estimate was used to calculate between studies variance in the random-effects model.

###### **Figure A7.22** Random Effects Models of The Pooled Prevalence Estimate for Feeding and Swallowing Difficulties in CHARGE Syndrome

**Note:** The pooled prevalence estimate for feeding and swallowin0g difficulties in CHARGE syndrome is 80% (95% CI, 74-85%; permuted p-value = 0.001; k = 21) with high heterogeneity (I^2^ = 77%). Random-effects model calculated using the inverse variance method and the DerSimonian-Laird estimator for τ2. Rosenthal Fail-safe N = 27790 suggests that the observed effect is robust to potential publication biases.

| **Figure A7.23** Funnel Plot of Standard Error by Prevalence of Feeding and Swallowing Difficulties | **Figure A7.24** Baujat Plot of Contribution to Heterogeneity by Influence on Overall Effect for Studies Reporting Feeding and Swallowing Difficulties |
| --- | --- |
|  |  |
| **Note:** Publication bias [small study effect] was identified (Egger’s test p = 0.018). The trim and fill procedure did not impute any missing studies | **Note:** Oley et al. (1988) had the greatest contribution to overall heterogeneity and the greatest influence on the overall effect |

###### **Figure A7.25** Leave-One-Out Random Effects Model for Studies Reporting Feeding and Swallowing Difficulties


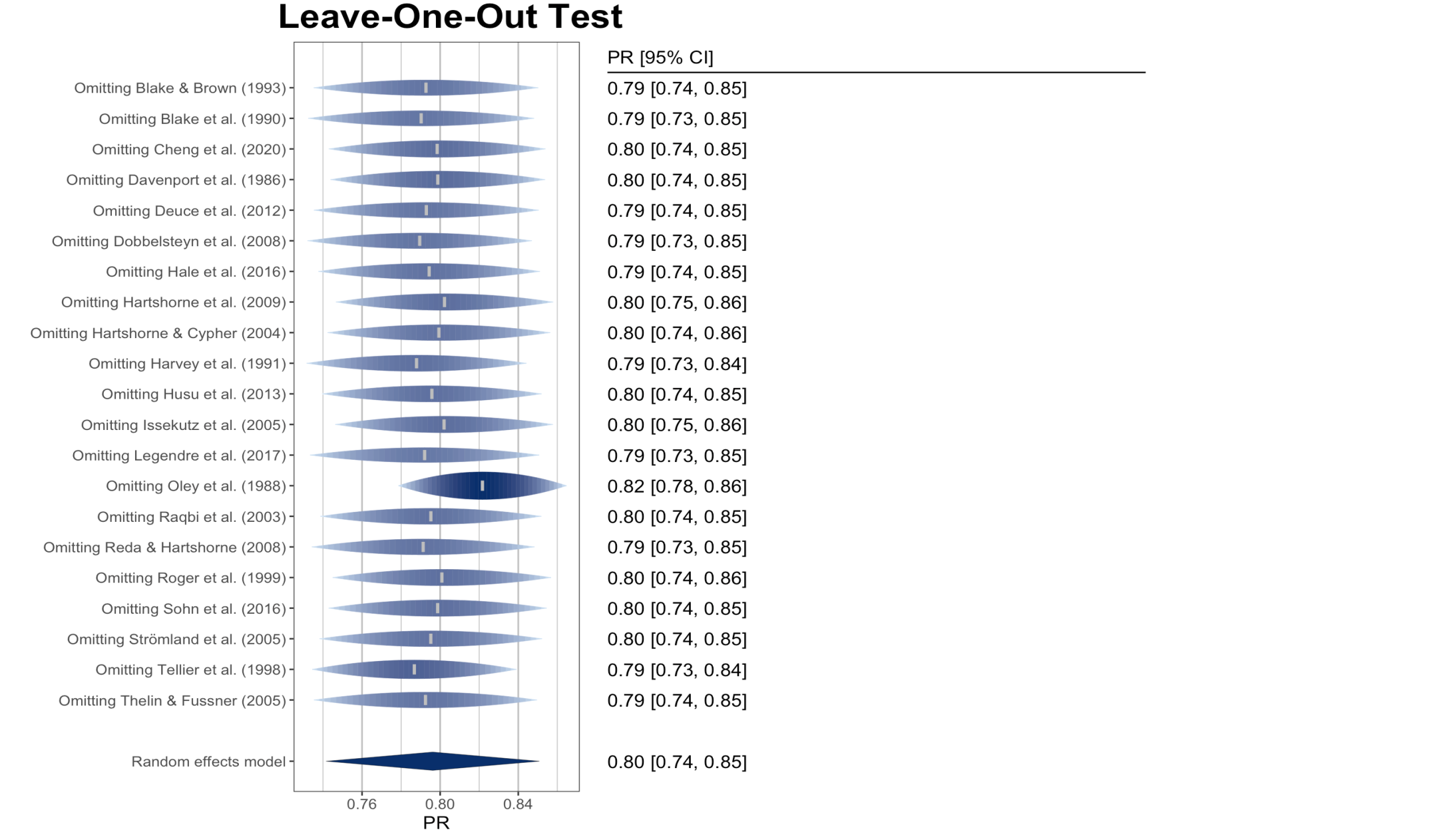


**Note:** Leave one out analysis indicating that no single study is exerting a disproportional influence on the pooled prevalence estimate

## **External Ear Anomalies**

###### **Figure A7.26** QQ Plot of The Distribution of Study Effects and Theoretical Quantities Based on A Normal Distribution Under the Random Effects Model for Studies Reporting External Ear Anomalies

**Note:** Visual inspection of the *QQ* plot suggests an approximate normal distribution of study effects for the 14 studies reporting external ear anomalies in CHARGE Syndrome. On this basis the DerSimonian-Laird estimate was used to calculate between studies variance in the random-effects model.

###### **Figure A7.27** Random Effects Models of The Pooled Prevalence Estimate for External Ear Anomalies in CHARGE Syndrome

**Note:** The pooled prevalence estimate for external ear anomalies in CHARGE syndrome, based on above model, is 79% (95% CI, 69-88%; permuted p-value = 0.001; k = 14) with high heterogeneity (I^2^ = 88%). Random-effects model calculated using the inverse variance method and the DerSimonian-Laird estimator for τ^2^. Rosenthal Fail-safe N = 10698 suggests that the observed effect is robust to potential publication biases.

| **Figure A7.28** Funnel Plot of Standard Error by Prevalence of External Ear Anomalies | **Figure A7.29** Baujat Plot of Contribution to Heterogeneity by Influence on Overall Effect for Studies Reporting External Ear Anomalies |
| --- | --- |
|  |  |
| **Note:** Visual inspection of the funnel plot conforms to normal expectations and there is weak evidence of substantial publication bias (Egger’s test p = 0.227) | **Note:** Deuce et al. (2012) had the greatest contribution to overall heterogeneity and the greatest influence on the overall effect. |

###### **Figure A7.30** Leave-One-Out Random Effects Model for Studies Reporting External Ear Anomalies


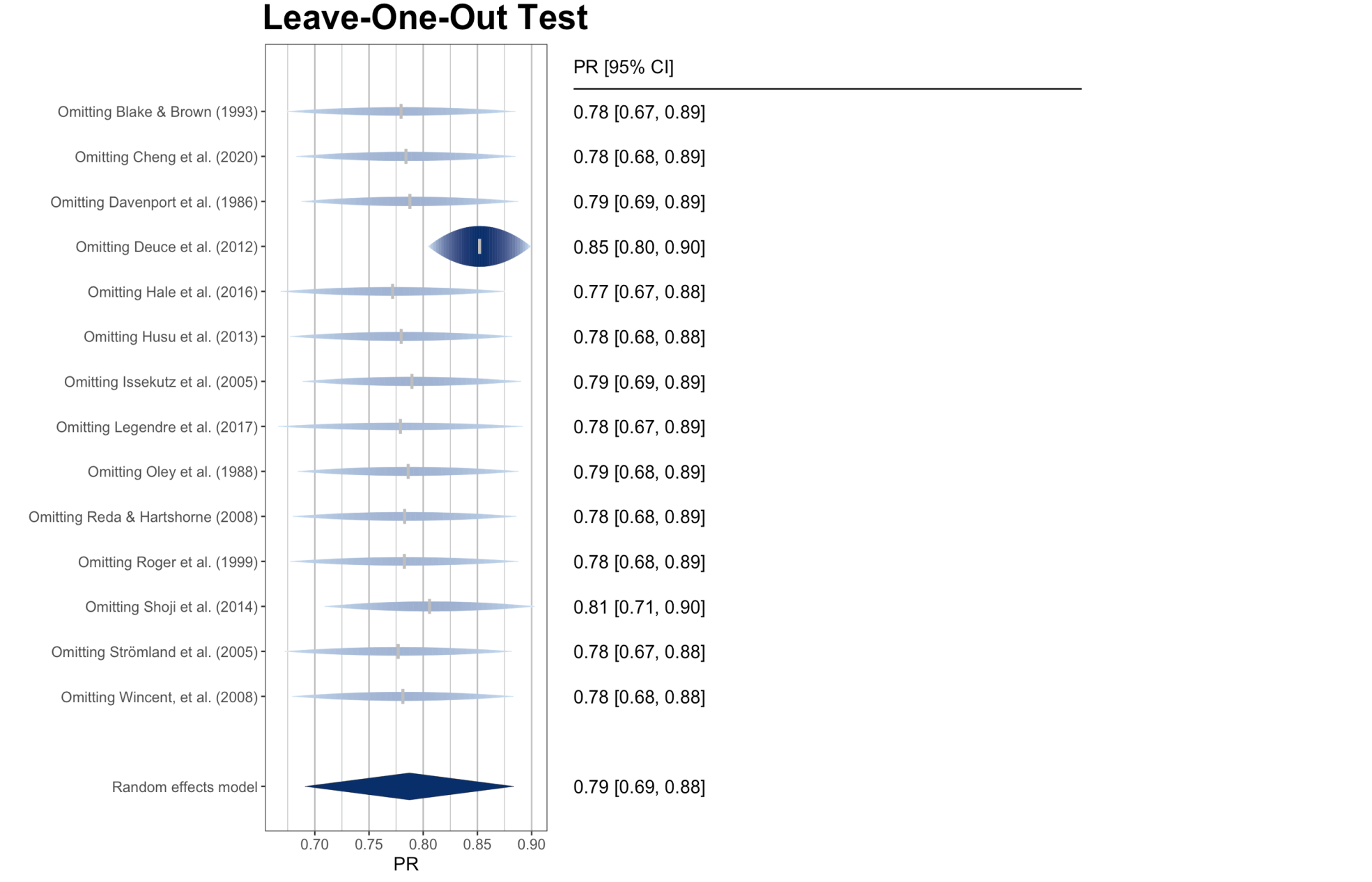


**Note:** Leave one out analysis indicating that the Deuce et al. (2012) study is exerting a disproportional influence on the pooled prevalence estimate

###### **Figure A7.31** Random Effects Models of The Pooled Prevalence Estimate for External Ear Anomalies in CHARGE Syndrome

**Note:** Based on the results of the sensitivity analysis Deuce *et al* (2012) was removed from the final model. The pooled prevalence estimate for external ear anomalies in CHARGE syndrome is 85% (95% CI, 80-90%; permuted p-value = 0.002; k = 13) with moderate heterogeneity (I^2^ = 41%). Random-effects model calculated using the inverse variance method and the DerSimonian-Laird estimator for τ^2^.

## **Middle Ear Anomalies**

###### **Figure A7.32** QQ Plot of The Distribution of Study Effects and Theoretical Quantities Based on A Normal Distribution Under the Random Effects Model for Studies Reporting Middle Ear Anomalies

**Note:** Visual inspection of the *QQ* plot suggests an approximate normal distribution of study effects for the 7 studies reporting middle ear anomalies in CHARGE Syndrome. On this basis the DerSimonian-Laird estimate was used to calculate between studies variance in the random-effects model.

###### **Figure A7.33** Random Effects Models of The Pooled Prevalence Estimate for Middle Ear Anomalies in CHARGE Syndrome

**Note:** The pooled prevalence estimate for middle ear anomalies in CHARGE syndrome is 50% (95% CI, 35-66%; permuted p-value = 0.016; k = 7) with high heterogeneity (I^2^ = 85%). Random-effects model calculated using the inverse variance method and the DerSimonian-Laird estimator for τ^2^.

###### **Figure A7.34** Baujat Plot of Contribution to Heterogeneity by Influence on Overall Effect for Studies Reporting Middle Ear Anomalies

**Note:** Issekutz et al. (2005) had the greatest contribution to overall heterogeneity and the greatest influence on the overall effect

###### **Figure A7.35** Leave-One-Out Random Effects Model for Studies Reporting Middle Ear Anomalies


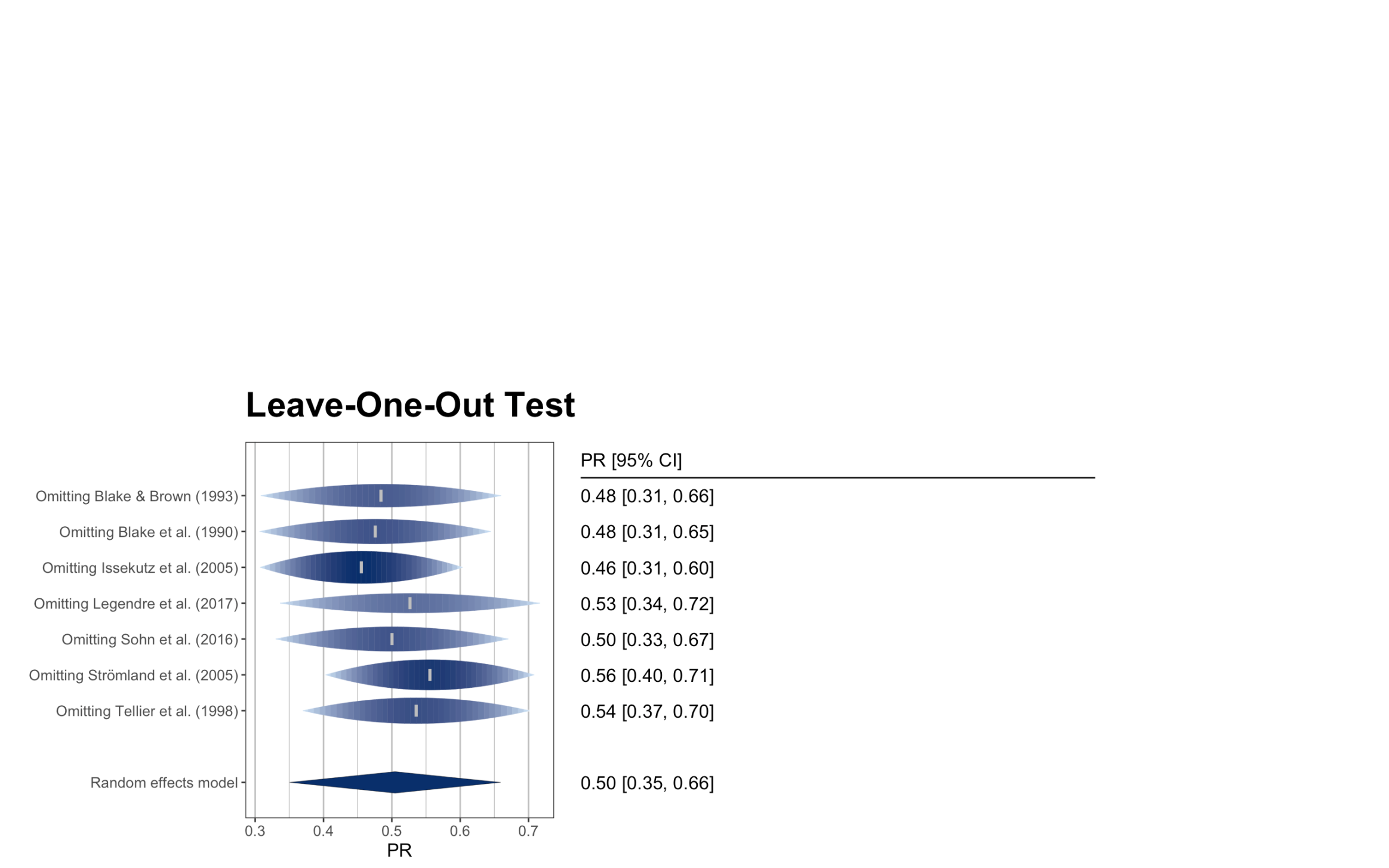


**Note:** Leave one out analysis indicating that no single study is exerting a disproportional influence on the pooled prevalence estimate

## **Inner Ear Anomalies**

###### **Figure A7.36** QQ Plot of The Distribution of Study Effects and Theoretical Quantities Based on A Normal Distribution Under the Random Effects Model for Studies Reporting Inner Ear Anomalies

**Note:** Visual inspection of the *QQ* plot suggests an approximate normal distribution of study effects for the 13 studies reporting inner ear anomalies in CHARGE Syndrome. On this basis the DerSimonian-Laird estimate was used to calculate between studies variance in the random-effects model.

###### **Figure A7.37** Random Effects Models of The Pooled Prevalence Estimate for Inner Ear Anomalies in CHARGE Syndrome

**Note:** The pooled prevalence estimate for inner ear anomalies in CHARGE syndrome is 75% (95% CI, 65-85%; permuted p-value = 0.003; k = 13) with high heterogeneity (I^2^ = 90%). Random-effects model calculated using the inverse variance method and the DerSimonian-Laird estimator for τ^2^. Rosenthal Fail-safe N = 11368 suggests that the observed effect is robust to potential publication biases.

| **Figure A7.38** Funnel Plot of Standard Error by Prevalence of Inner Ear Anomalies Following the Trim and Fill Procedure | **Figure A7.39** Baujat Plot of Contribution to Heterogeneity by Influence on Overall Effect for Studies Reporting Inner Ear Anomalies |
| --- | --- |
|  |  |
| **Note:** Publication bias [small study effect] was identified (Egger’s test p = 0.006). The trim and fill procedure did not impute any missing studies. | **Note:** Blake et al. (1990) had the greatest contribution to overall heterogeneity and the greatest influence on the overall effect. |

###### **Figure A7.40** Leave-One-Out Random Effects Model for Studies Reporting Inner Ear Anomalies


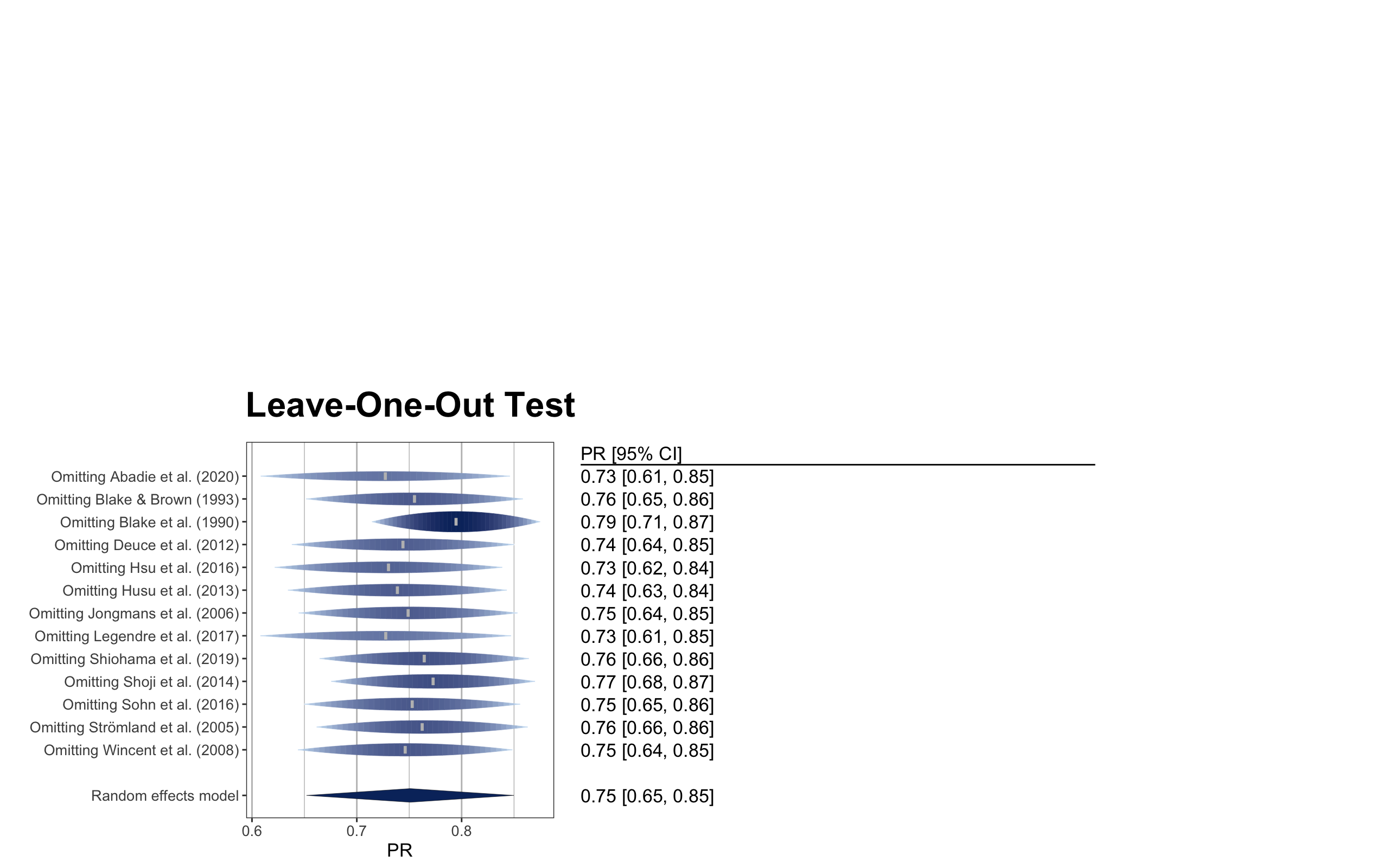


**Note:** Leave one out analysis indicating that no single study is exerting a disproportional influence on the pooled prevalence estimate

## **Heart Defects**

###### **Figure A7.41** QQ Plot of The Distribution of Study Effects and Theoretical Quantities Based on A Normal Distribution Under the Random Effects Model for Studies Reporting Heart Defects

**Note:** Visual inspection of the *QQ* plot suggests an approximate normal distribution of study effects for the 30 studies reporting heart defects in CHARGE Syndrome. On this basis the DerSimonian-Laird estimate was used to calculate between studies variance in the random-effects model.

###### **Figure A7.42** Random Effects Models of The Pooled Prevalence Estimate for Heart Defects in CHARGE Syndrome

**Note:** The pooled prevalence estimate for heart defects in CHARGE syndrome is 75% (95% CI, 70-79%; permuted p-value = 0.001; k = 30) with moderate heterogeneity (I2 = 67%). Random-effects model calculated using the inverse variance method and the DerSimonian-Laird estimator for τ^2^. Rosenthal Fail-safe N = 39228 suggests that the observed effect is robust to potential publication biases.

| **Figure A7.43** Funnel Plot of Standard Error by Prevalence of Heart Defects | **Figure A7.44** Baujat Plot of Contribution to Heterogeneity by Influence on Overall Effect for Studies Reporting Heart Defects |
| --- | --- |
|  |  |
| **Note:** Visual inspection of the funnel plot conforms to normal expectations and there is weak evidence of substantial publication bias (Egger’s test p = 0.348) | **Note:** Deuce et al. (2012) had the greatest contribution to overall heterogeneity and the greatest influence on the overall effect. |

###### **Figure A7.45** Leave-One-Out Random Effects Model for Studies Reporting Heart Defects


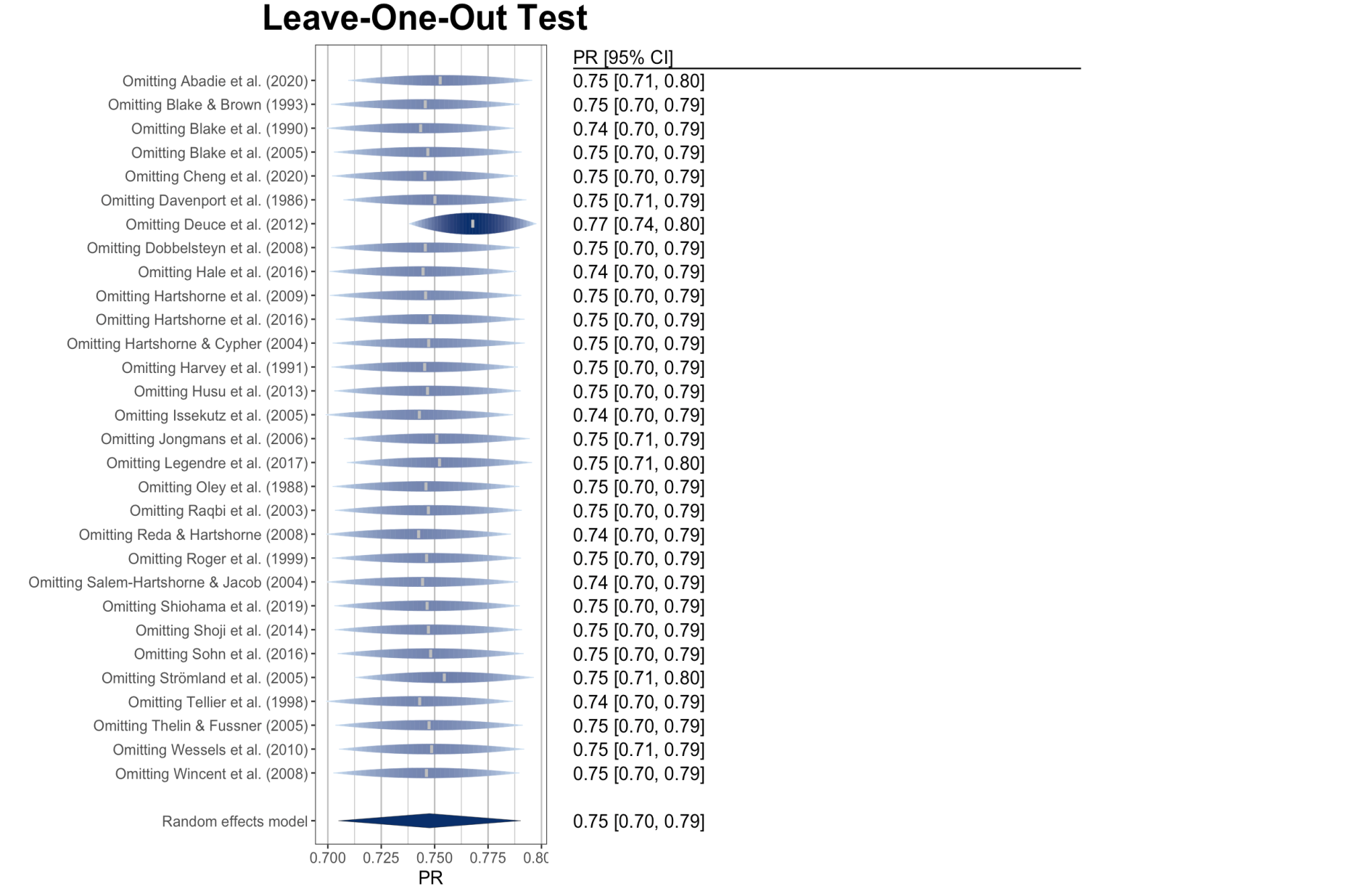


**Note:** Leave one out analysis indicating that no single study is exerting a disproportional influence on the pooled prevalence estimate

## **Growth Deficiency**

###### **Figure A7.46** QQ Plot of The Distribution of Study Effects and Theoretical Quantities Based on A Normal Distribution Under the Random Effects Model for Studies Reporting Growth Deficiency

**Note:** Visual inspection of the *QQ* plot suggests an approximate normal distribution of study effects for the 29 studies reporting growth deficiency in CHARGE Syndrome. On this basis the DerSimonian-Laird estimate was used to calculate between studies variance in the random-effects model.

###### **Figure A7.47** Random Effects Models of The Pooled Prevalence Estimate for Growth Deficiency in CHARGE Syndrome

**Note:** The pooled prevalence estimate for growth deficiency in CHARGE syndrome is 66% (95% CI, 59-73%; permuted p-value = 0.031; k = 29) with high heterogeneity (I^2^ = 86%). Random-effects model calculated using the inverse variance method and the DerSimonian-Laird estimator for τ^2^. Rosenthal Fail-safe N = 23693 suggests that the observed effect is robust to potential publication biases

| **Figure A7.48** Funnel Plot of Standard Error by Prevalence of Growth Deficiency | **Figure A7.49** Baujat Plot of Contribution to Heterogeneity by Influence on Overall Effect for Studies Reporting Growth Deficiency |
| --- | --- |
|  |  |
| **Note:** Visual inspection of the funnel plot conforms to normal expectations and there is weak evidence of substantial publication bias (Egger’s test p = 0.267) | **Note:** Deuce et al. (2012) had the greatest contribution to overall heterogeneity and the greatest influence on the overall effect. |

###### **Figure A7.50** Leave-One-Out Random Effects Model for Studies Reporting Growth Deficiency


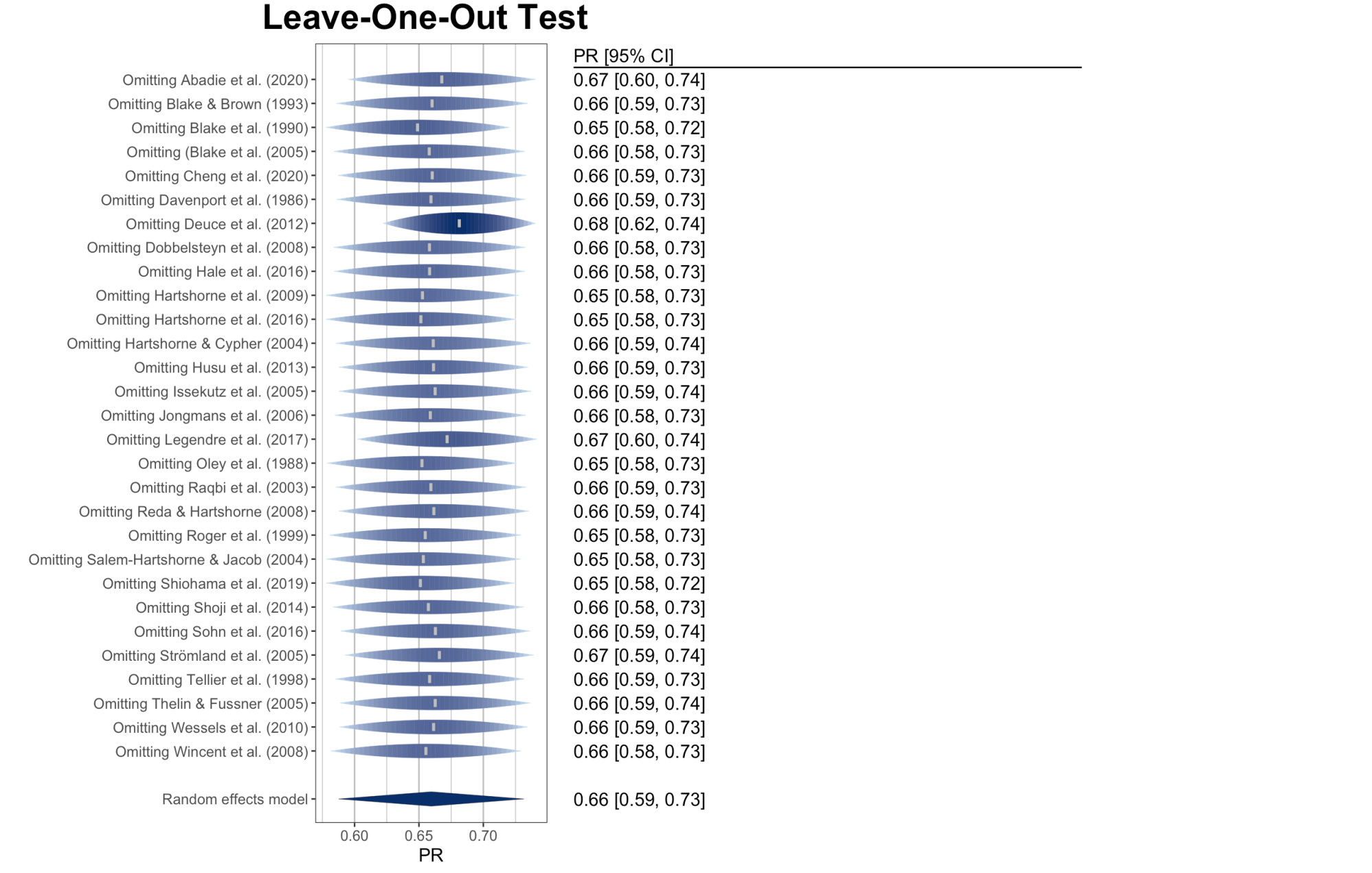


**Note:** Leave one out analysis indicating that no single study is exerting a disproportional influence on the pooled prevalence estimate

## **Genital Hypoplasia**

###### **Figure A7.51** QQ Plot of The Distribution of Study Effects and Theoretical Quantities Based on A Normal Distribution Under the Random Effects Model for Studies Reporting Genital Hypoplasia

**Note:** Visual inspection of the *QQ* plot suggests an approximate normal distribution of study effects for the 26 studies reporting genital hypoplasia in CHARGE Syndrome. On this basis the DerSimonian-Laird estimate was used to calculate between studies variance in the random-effects model.

###### **Figure A7.52** Random Effects Models of The Pooled Prevalence Estimate for Genital Hypoplasia in CHARGE Syndrome

**Note:** The pooled prevalence estimate for genital hypoplasia in CHARGE syndrome is 54% (95% CI, 46-62%; permuted p-value = 0.037; k = 26) with high heterogeneity (I^2^ = 87%). Random-effects model calculated using the inverse variance method and the DerSimonian-Laird estimator for τ^2^. Rosenthal Fail-safe N = 11548 suggests that the observed effect is robust to potential publication biases.

| **Figure A7.53** Funnel Plot of Standard Error by Prevalence of Genital Hypoplasia | **Figure A7.54** Baujat Plot of Contribution to Heterogeneity by Influence on Overall Effect for Studies Reporting Genital Hypoplasia |
| --- | --- |
|  |  |
| **Note:** Visual inspection of the funnel plot conforms to normal expectations and there is weak evidence of substantial publication bias (Egger’s test p = 0.654). | **Note:** Blake et al. (1990) had the greatest contribution to overall heterogeneity and the greatest influence on the overall effect. |

###### **Figure A7.55** Leave-One-Out Random Effects Model for Studies Reporting Genital Hypoplasia


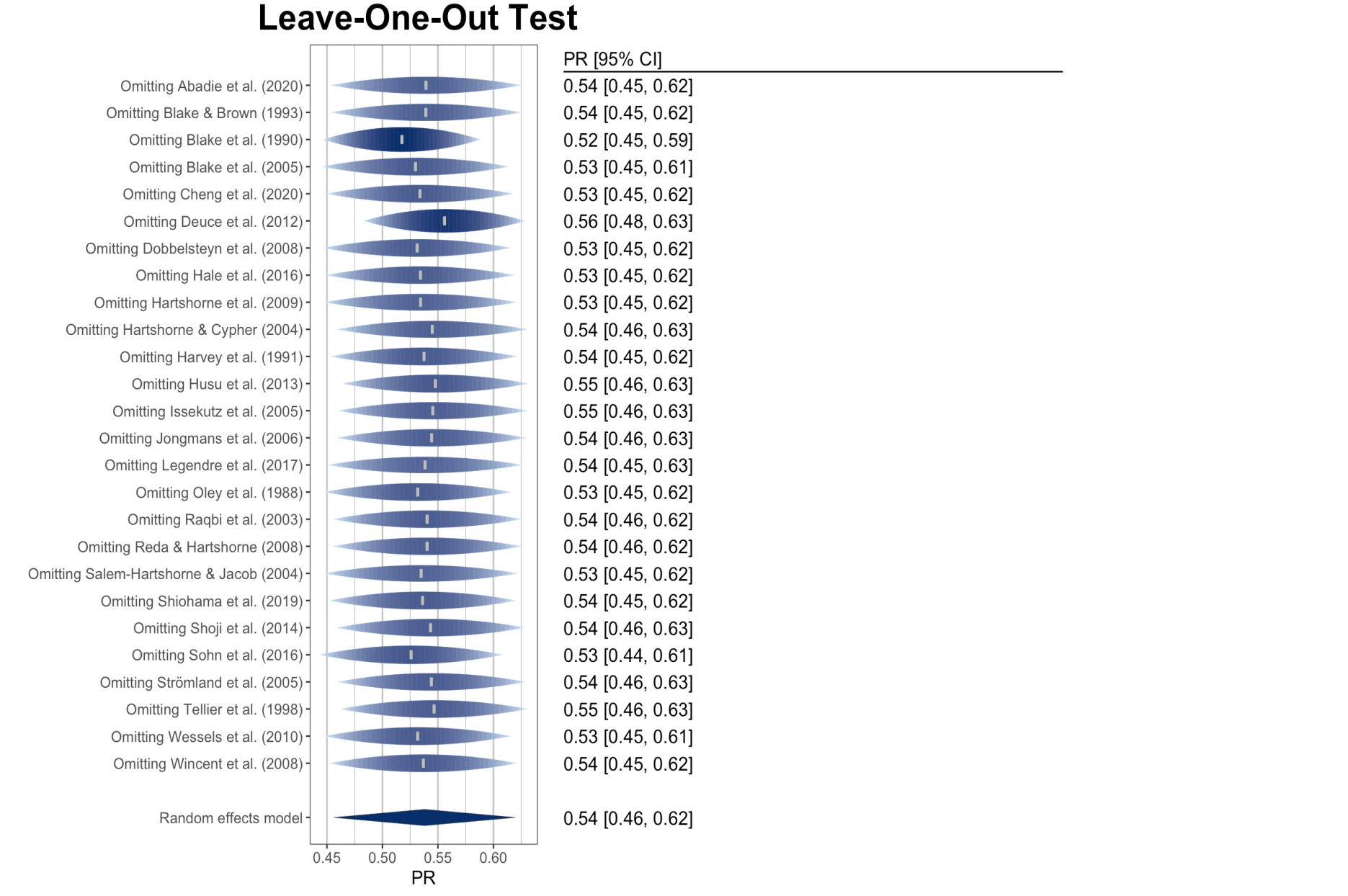


**Note:** Leave one out analysis indicating that no single study is exerting a disproportional influence on the pooled prevalence estimate

## **Cleft Lip or Palate**

###### **Figure A7.56** QQ Plot of The Distribution of Study Effects and Theoretical Quantities Based on A Normal Distribution Under the Random Effects Model for Studies Reporting Cleft Lip or Palate

**Note:** Visual inspection of the *QQ* plot suggests an approximate normal distribution of study effects for the 28 studies reporting cleft lip or palate in CHARGE Syndrome. On this basis the DerSimonian-Laird estimate was used to calculate between studies variance in the random-effects model.

###### **Figure A7.57** Random Effects Models of The Pooled Prevalence Estimate for Cleft Lip or Palate in CHARGE Syndrome

**Note:** The pooled prevalence estimate for cleft lip or palate in CHARGE syndrome is 25% (95% CI, 21-29%; permuted p-value = 0.001; k = 28) with moderate heterogeneity (I^2^ = 66%). Random-effects model calculated using the inverse variance method and the DerSimonian-Laird estimator for τ^2^. Rosenthal Fail-safe N = 3795 suggests that the observed effect is robust to potential publication biases

| **Figure A7.58** Funnel Plot of Standard Error by Prevalence of Cleft Lip or Palate Following the Trim and Fill Procedure | **Figure A7.59** Baujat Plot of Contribution to Heterogeneity by Influence on Overall Effect for Studies Reporting Cleft Lip or Palate |
| --- | --- |
|  |  |
| **Note:** Publication bias [small study effect] was identified (Egger’s test p = 0.013). Using the trim and fill procedure it was estimated that 8 (SE = 3.47) studies were missing on the left side. Adjusted estimate = 20%, (95% CI = 16-25%, p = <.001; τ2 = 0.111, I2 = 74%). | **Note:** Blake et al. (1990) had the greatest contribution to overall heterogeneity and the greatest influence on the overall effect. |

###### **Figure A7.60** Leave-One-Out Random Effects Model for Studies Reporting Cleft Lip or Palate


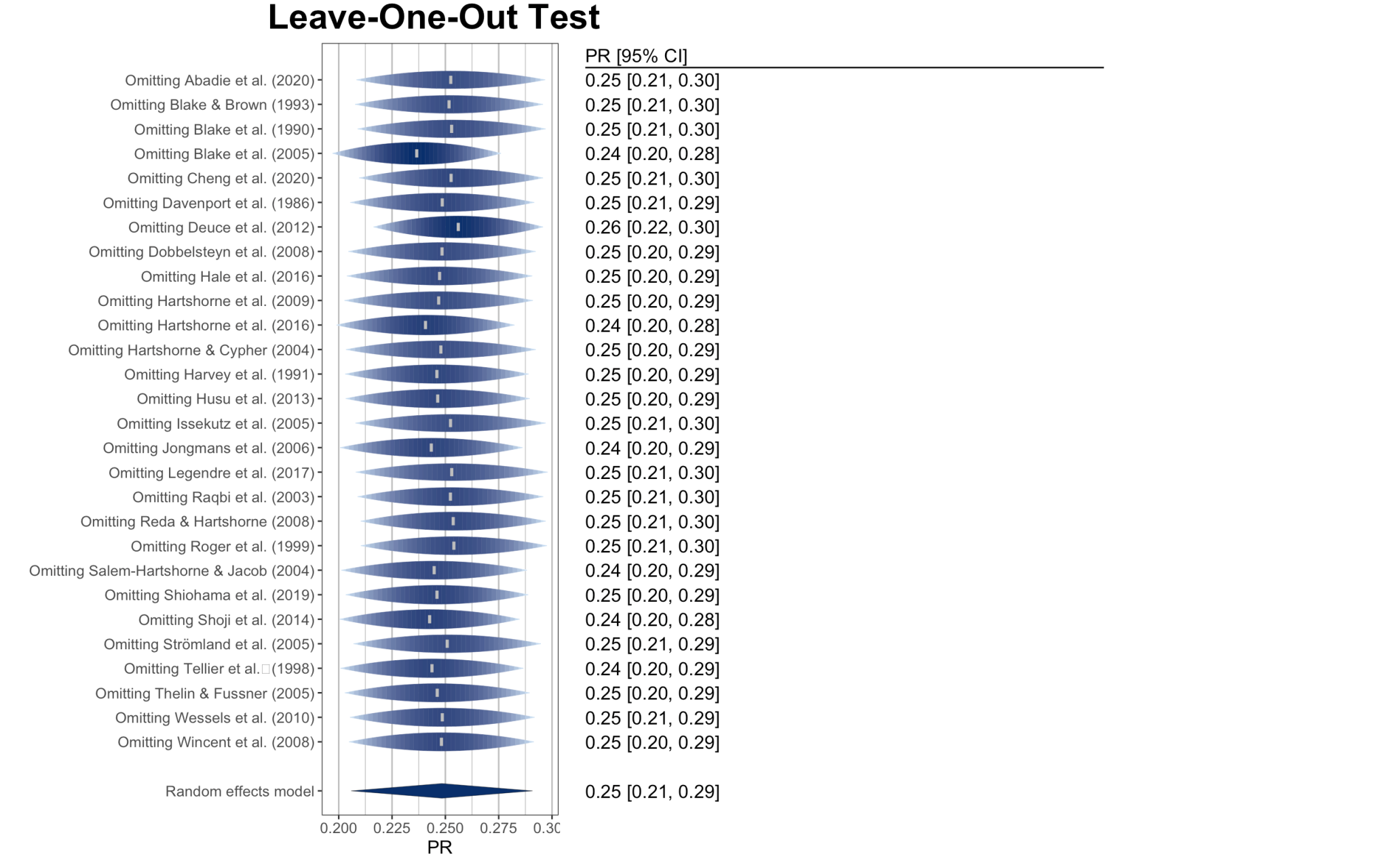


**Note:** Leave one out analysis indicating that no single study is exerting a disproportional influence on the pooled prevalence estimate

## **Tracheoesophageal Fistula**

###### **Figure A7.61** QQ Plot of The Distribution of Study Effects and Theoretical Quantities Based on A Normal Distribution Under the Random Effects Model for Studies Reporting Tracheoesophageal Fistula

**Note:** Visual inspection of the QQ plot suggests an approximate normal distribution of study effects for the 21 studies reporting tracheoesophageal fistula in CHARGE Syndrome. On this basis the DerSimonian-Laird estimate was used to calculate between studies variance in the random-effects model.

###### **Figure A7.62** Random Effects Models of The Pooled Prevalence Estimate for Tracheoesophageal Fistula in CHARGE Syndrome

**Note:** The pooled prevalence estimate for tracheoesophageal fistula in CHARGE syndrome is 17% (95% CI, 14-20%; permuted p-value = 0.001; k = 21) with low heterogeneity (I2 = 30%). Random-effects model calculated using the inverse variance method and the DerSimonian-Laird estimator for τ2. Rosenthal Fail-safe N = 1553 suggests that the observed effect is robust to potential publication biases

| **Figure A7.63** Funnel Plot of Standard Error by Prevalence of Tracheoesophageal Fistula Following the Trim and Fill Procedure | **Figure A7.64** Baujat Plot of Contribution to Heterogeneity by Influence on Overall Effect for Studies Reporting Tracheoesophageal Fistula |
| --- | --- |
|  |  |
| **Note:** Publication bias [small study effect] was identified (Egger’s test p = 0.035). Using the trim and fill procedure it was estimated that 7 (SE = 2.95) studies were missing on the left side. Adjusted estimate = 15%, (95% *CI* = 12-18%, *p* = <.001; τ^2^ = 0.003, I^2^ = 47%). | **Note:** Studies in the top right quartile have the greatest contribution to overall heterogeneity and the greatest influence on the overall effect. Salem-Hartshorne & Jacob (2004) had the greatest contribution to overall heterogeneity and Dobbelsteyn et al. (2008) had the greatest influence on the overall effect. |

###### **Figure A7.65** Leave-One-Out Random Effects Model for Studies Reporting Tracheoesophageal Fistula


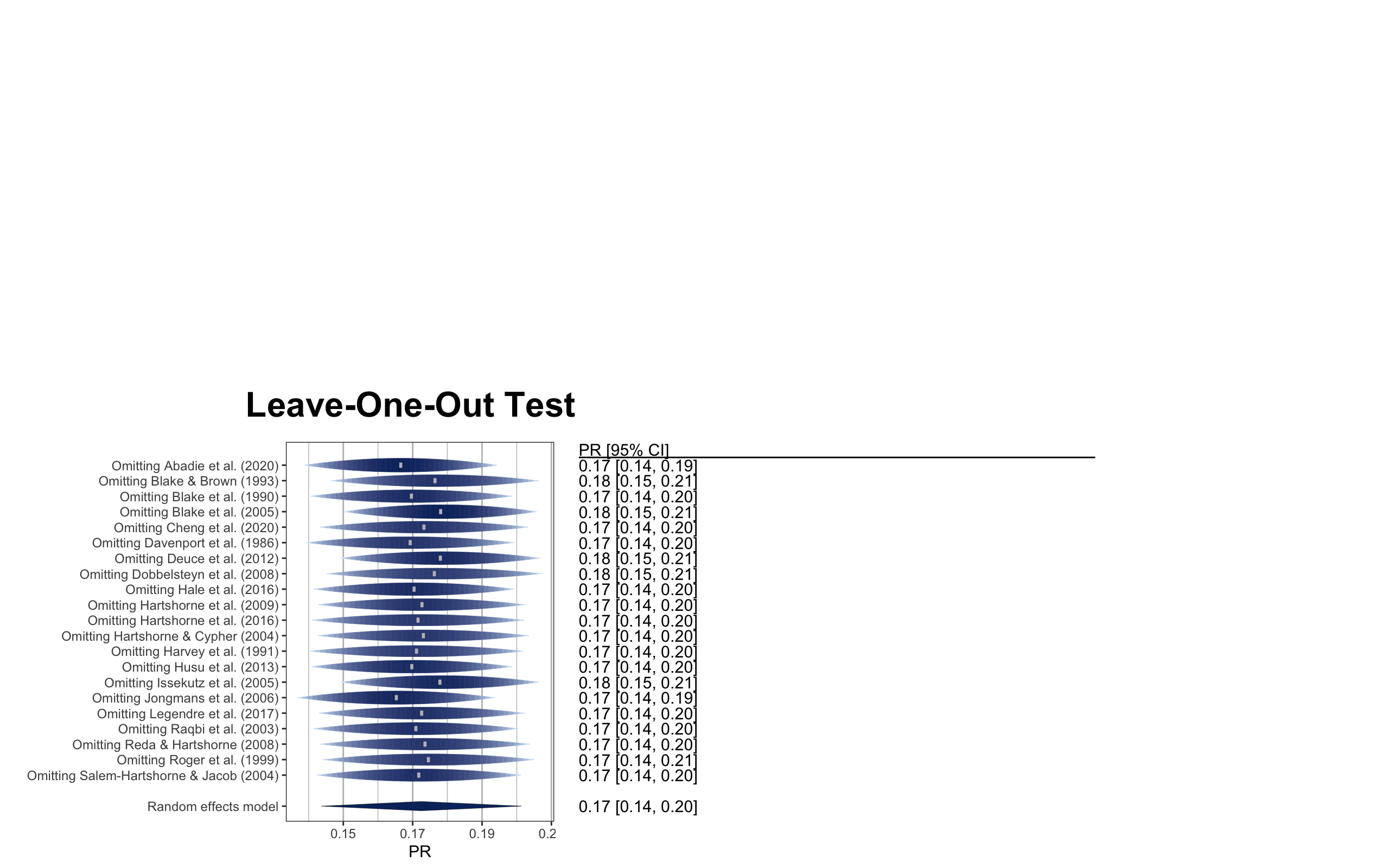


**Note:** Leave one out analysis indicating that no single study is exerting a disproportional influence on the pooled prevalence estimate.

## **Brain Anomalies**

###### **Figure A7.66** QQ Plot of The Distribution of Study Effects and Theoretical Quantities Based on A Normal Distribution Under the Random Effects Model for Studies Reporting Brain Anomalies

**Note:** Visual inspection of the *QQ* plot suggests an approximate normal distribution of study effects for the 18 studies reporting brain anomalies in CHARGE Syndrome. On this basis the DerSimonian-Laird estimate was used to calculate between studies variance in the random-effects model.

###### **Figure A7.67** Random Effects Models of The Pooled Prevalence Estimate for Studies Reporting Brain Anomalies in CHARGE Syndrome

**Note:** The pooled prevalence estimate for brain anomalies in CHARGE syndrome is 43% (95% CI, 30-57%; permuted p-value = 0.001; k = 18) with high heterogeneity (I^2^ = 93%). Random-effects model calculated using the inverse variance method and the DerSimonian-Laird estimator for τ^2^. Rosenthal Fail-safe N = 3377 suggests that the observed effect is robust to potential publication biases.

| **Figure A7.68** Funnel Plot of Standard Error by Prevalence of Brain Anomalies | **Figure A7.69** Baujat Plot of Contribution to Heterogeneity by Influence on Overall Effect for Studies Reporting Brain Anomalies |
| --- | --- |
|  |  |
| **Note:** Visual inspection of the funnel plot conforms to normal expectations and there is weak evidence of substantial publication bias (Egger’s test p = 0.61). | **Note:** Strömland et al. (2005) had the greatest contribution to overall heterogeneity and the greatest influence on the overall effect. |

###### **Figure A7.70** Leave-One-Out Random Effects Model for Studies Reporting Brain Anomalies


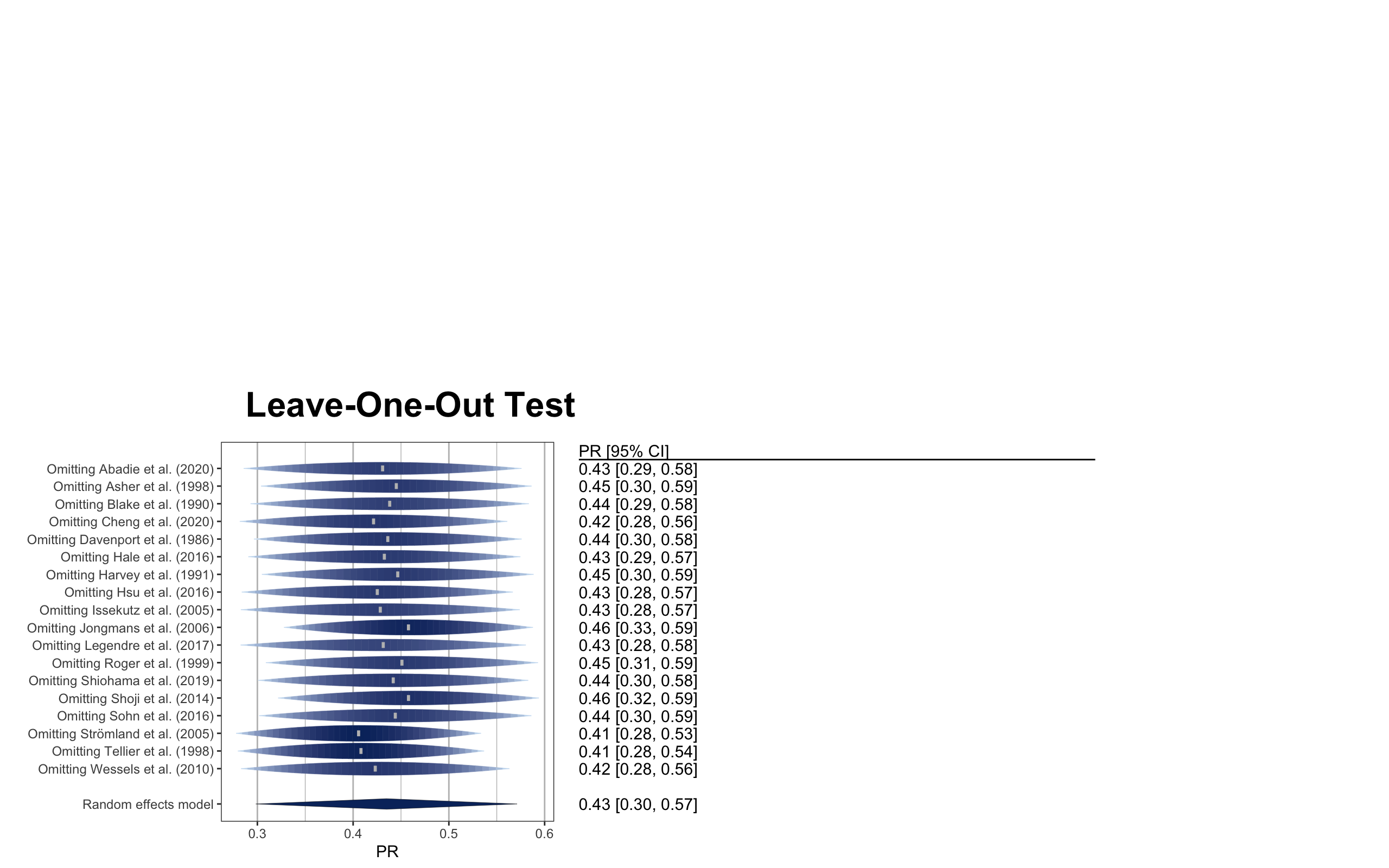


**Note:** Leave one out analysis indicating that no single study is exerting a disproportional influence on the pooled prevalence estimate

## **Characteristic Face**

###### **Figure A7.71** QQ Plot of The Distribution of Study Effects and Theoretical Quantities Based on A Normal Distribution Under the Random Effects Model for Studies Reporting Characteristic Face

**Note:** Visual inspection of the QQ plot suggests an approximate normal distribution of study effects for the 9 studies reporting characteristic face in CHARGE Syndrome. On this basis the DerSimonian-Laird estimate was used to calculate between studies variance in the random-effects model.

###### **Figure A7.72** Random Effects Models of The Pooled Prevalence Estimate for Studies Reporting Characteristic Face in CHARGE Syndrome

**Note:** The pooled prevalence estimate for characteristic face in CHARGE syndrome, based on the model above, is 63% (95% CI, 37-89%; permuted p-value = 0.003; k = 9) with high heterogeneity (I^2^ = 98%). Random-effects model calculated using the inverse variance method and the DerSimonian-Laird estimator for τ^2^

###### **Figure A7.73** Baujat Plot of Contribution to Heterogeneity by Influence on Overall Effect for Studies Reporting Characteristic Face

**Note:** Deuce et al. (2012) had the greatest contribution to overall heterogeneity and the greatest influence on the overall effect.

###### **Figure A7.74** Leave-One-Out Random Effects Model for Studies Reporting Characteristic Face


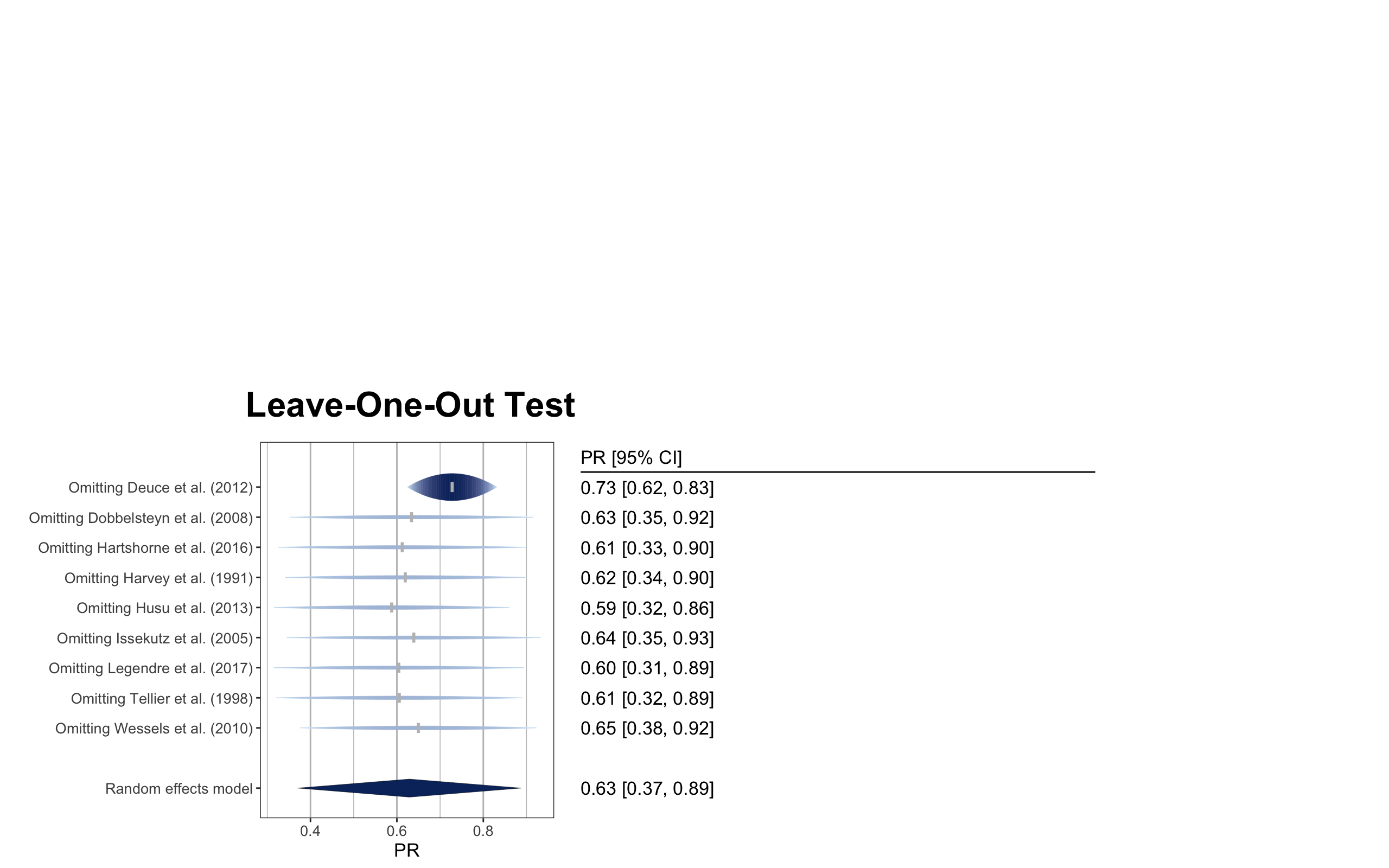


**Note:** Leave one out analysis indicating that the Deuce et al. (2012) study is exerting a disproportional influence on the pooled prevalence estimate

###### **Figure A7.75** Random Effects Models of The Pooled Prevalence Estimate for Studies Reporting Characteristic Face in CHARGE Syndrome

**Note:** Based on the results of the sensitivity analysis Deuce *et al* (2012) was removed from the final model. The pooled prevalence estimate for characteristic face in CHARGE syndrome is 73% (95% CI, 62-83%; permuted p-value = 0.008; k = 8) with high heterogeneity (I^2^ = 80%). Random-effects model calculated using the inverse variance method and the DerSimonian-Laird estimator for τ^2^.

## **Hearing Impairment**

###### **Figure A7.76** QQ Plot of The Distribution of Study Effects and Theoretical Quantities Based on A Normal Distribution Under the Random Effects Model for Studies Reporting Hearing Impairment

**Note:** Visual inspection of the *QQ* plot suggests an approximate normal distribution of study effects for the 25 studies reporting hearing impairment in CHARGE Syndrome. On this basis the DerSimonian-Laird estimate was used to calculate between studies variance in the random-effects model.

###### **Figure A7.77** Random Effects Models of The Pooled Prevalence Estimate for Studies Reporting Hearing Impairment in CHARGE Syndrome

**Note:** The pooled prevalence estimate for hearing impairment in CHARGE syndrome is 87% (95% CI, 84-91%; permuted p-value = 0.001; k = 25) with moderate heterogeneity (I^2^ = 70%). Random-effects model calculated using the inverse variance method and the DerSimonian-Laird estimator for τ^2^. Rosenthal Fail-safe N = 77214 suggests that the observed effect is robust to potential publication biases.

| **Figure A7.78** Funnel Plot of Standard Error by Prevalence of Hearing Impairment | **Figure A7.79** Baujat Plot of Contribution to Heterogeneity by Influence on Overall Effect for Studies Reporting Hearing Impairment |
| --- | --- |
|  |  |
| **Note:** Visual inspection of the funnel plot conforms to normal expectations and there is weak evidence of substantial publication bias (Egger’s test p = 2.018) | **Note:** Studies in the top right quartile have the greatest contribution to overall heterogeneity and the greatest influence on the overall effect. Tellier et al. (1998) had the greatest contribution to overall heterogeneity and Harvey et al. (1991) had the greatest influence on the overall effect. |

###### **Figure A7.80** Leave-One-Out Random Effects Model for Studies Reporting Hearing Impairment


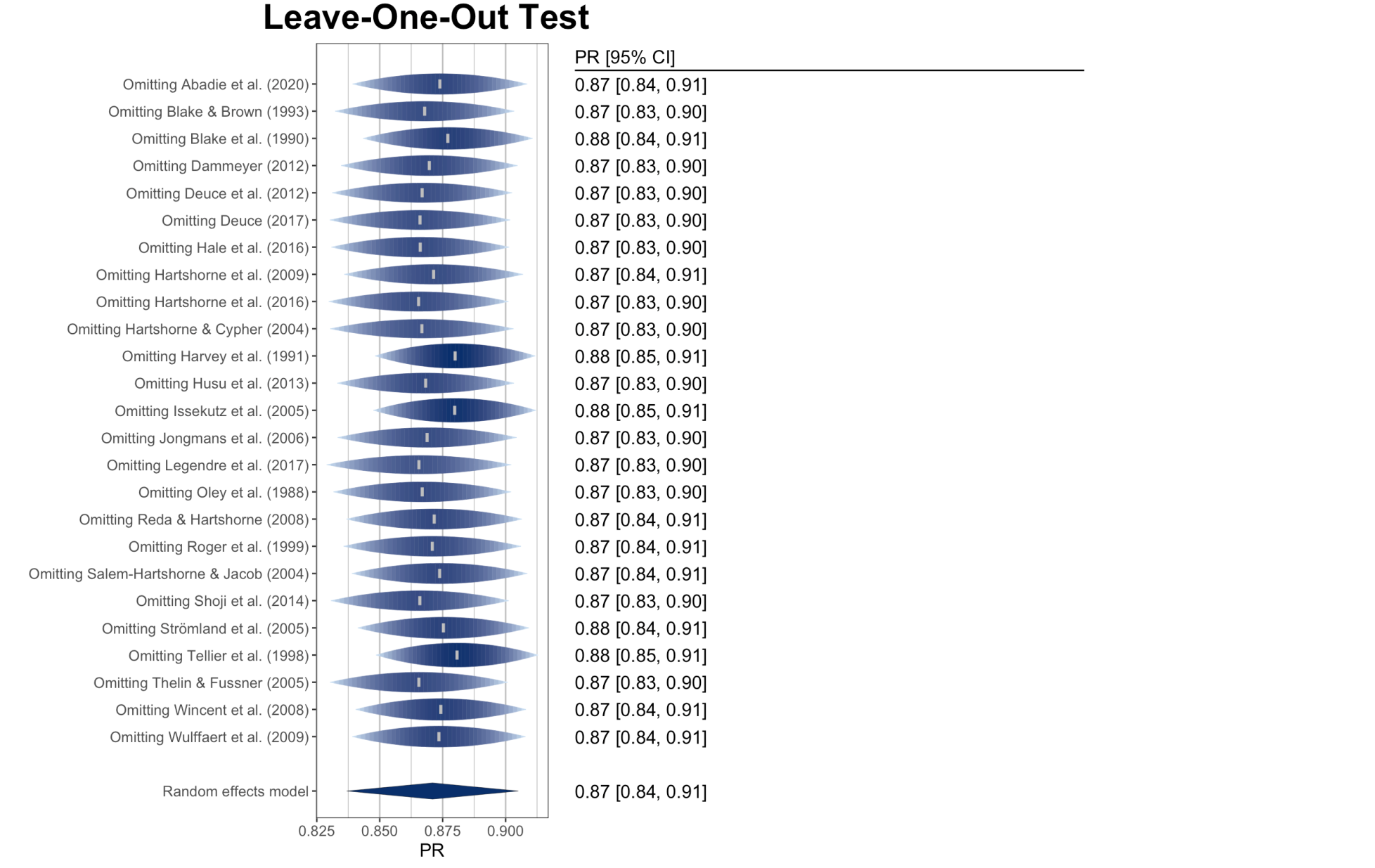


**Note:** Leave one out analysis indicating that no single study is exerting a disproportional influence on the pooled prevalence estimate.

## **Iris Coloboma**

###### **Figure A7.81** QQ Plot of The Distribution of Study Effects and Theoretical Quantities Based on A Normal Distribution Under the Random Effects Model for Studies Reporting Iris Coloboma

**Note:** Visual inspection of the *QQ* plot suggests an approximate normal distribution of study effects for the 10 studies reporting iris coloboma in CHARGE Syndrome. On this basis the DerSimonian-Laird estimate was used to calculate between studies variance in the random-effects model.

###### **Figure A7.82** Random Effects Models of The Pooled Prevalence Estimate for Studies Reporting Iris Coloboma in CHARGE Syndrome

**Note:** The pooled prevalence estimate for iris coloboma in CHARGE syndrome is 22% (95% CI, 13-32%; permuted p-value = 0.003; k = 10) with high heterogeneity (I^2^ = 82%). Random-effects model calculated using the inverse variance method and the DerSimonian-Laird estimator for τ^2^. Rosenthal Fail-safe N = 325 suggests that the observed effect is robust to potential publication biases.

| **Figure A7.83** Funnel Plot of Standard Error by Prevalence of Iris Coloboma | **Figure A7.84** Baujat Plot of Contribution to Heterogeneity by Influence on Overall Effect for Studies Reporting Iris Coloboma |
| --- | --- |
|  |  |
| **Note:** Visual inspection of the funnel plot conforms to normal expectations and there is weak evidence of substantial publication bias (Egger’s test p = 0.034) | **Note:** Blake and Brown (1993) had the greatest contribution to overall heterogeneity and the greatest influence on the overall effect. |

###### **Figure A7.85** Leave-One-Out Random Effects Model for Studies Reporting Iris Coloboma


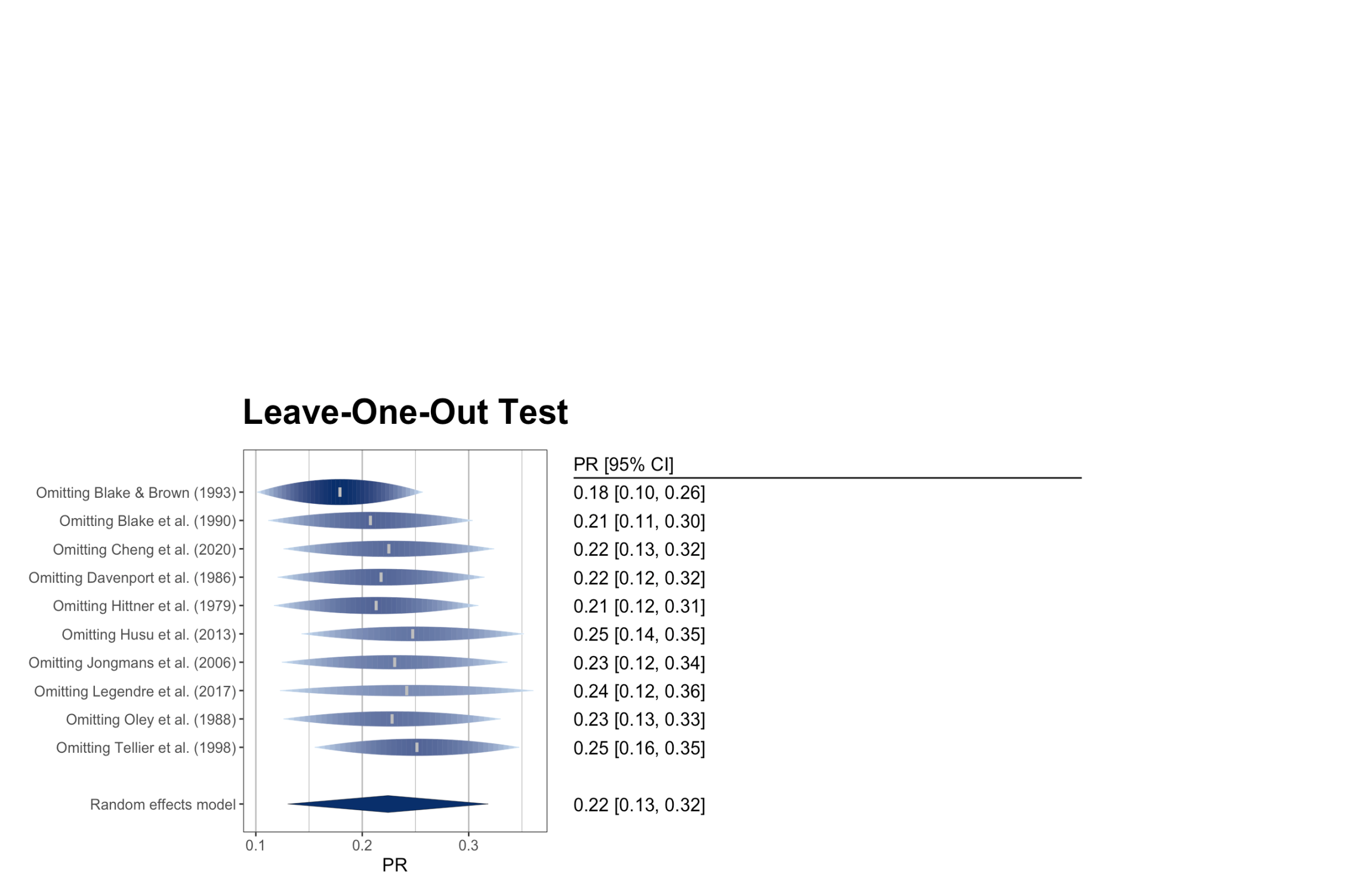


**Note:** Leave one out analysis indicating that no single study is exerting a disproportional influence on the pooled prevalence estimate

## **Posterior segment coloboma**

###### **Figure A7.86** QQ Plot of The Distribution of Study Effects and Theoretical Quantities Based on A Normal Distribution Under the Random Effects Model for Studies Reporting Posterior Segment Coloboma

**Note:** Visual inspection of the *QQ* plot suggests an approximate normal distribution of study effects for the 11 studies reporting posterior segment coloboma in CHARGE Syndrome. On this basis the DerSimonian-Laird estimate was used to calculate between studies variance in the random-effects model.

###### **Figure A7.87** Random Effects Models of The Pooled Prevalence Estimate for Studies Reporting Posterior Segment Coloboma in CHARGE Syndrome

**Note:** The pooled prevalence estimate for posterior segment coloboma in CHARGE syndrome is 77% (95% CI, 70-84%; permuted p-value = 0.002; k = 11) with moderate heterogeneity (I^2^ = 67%). Random-effects model calculated using the inverse variance method and the DerSimonian-Laird estimator for τ^2^. Rosenthal Fail-safe N = 5940 suggests that the observed effect is robust to potential publication biases.

| **Figure A7.88** Funnel Plot of Standard Error by Prevalence of Posterior Segment Coloboma | **Figure A7.89** Baujat Plot of Contribution to Heterogeneity by Influence on Overall Effect for Studies Reporting Posterior Segment Coloboma |
| --- | --- |
|  |  |
| **Note:** Visual inspection of the funnel plot conforms to normal expectations and there is weak evidence of substantial publication bias (Egger’s test p = 0.659) | **Note:** Tellier et al. (1998) had the greatest contribution to overall heterogeneity and the greatest influence on the overall effect. |

###### **Figure A7.90** Leave-One-Out Random Effects Model for Studies Reporting Posterior Segment Coloboma


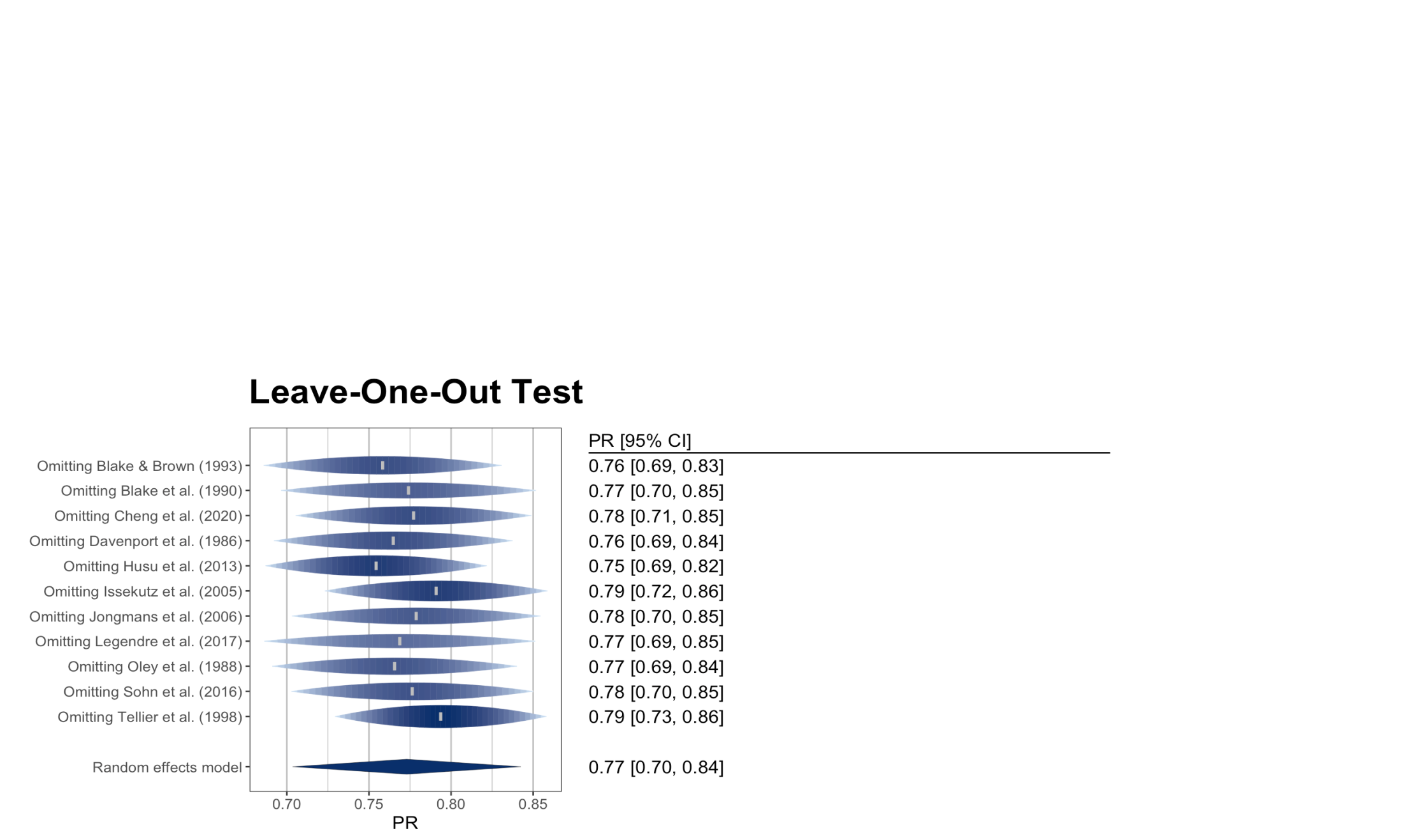


**Note:** Leave one out analysis indicating that no single study is exerting a disproportional influence on the pooled prevalence estimate

## **Bilateral Coloboma**

###### **Figure A7.91** QQ Plot of The Distribution of Study Effects and Theoretical Quantities Based on A Normal Distribution Under the Random Effects Model for Studies Reporting Bilateral Coloboma

**Note:** Visual inspection of the *QQ* plot suggests an approximate normal distribution of study effects for the 12 studies reporting bilateral coloboma in CHARGE Syndrome. On this basis the DerSimonian-Laird estimate was used to calculate between studies variance in the random-effects model.

###### **Figure A7.92** Random Effects Models of The Pooled Prevalence Estimate for Studies Reporting Bilateral Coloboma in CHARGE Syndrome

**Note:** The pooled prevalence estimate for bilateral coloboma in CHARGE syndrome is 59% (95% CI, 50-68%; permuted p-value = 0.002; k = 12) with moderate heterogeneity (I^2^ = 72%). Random-effects model calculated using the inverse variance method and the DerSimonian-Laird estimator for τ^2^. Rosenthal Fail-safe N = 2390 suggests that the observed effect is robust to potential publication biases.

| **Figure A7.93** Funnel Plot of Standard Error by Prevalence of Bilateral Coloboma | **Figure A7.94** Baujat Plot of Contribution to Heterogeneity by Influence on Overall Effect for Studies Reporting Bilateral Coloboma |
| --- | --- |
|  |  |
| **Note:** Visual inspection of the funnel plot conforms to normal expectations and there is weak evidence of substantial publication bias (Egger’s test p = 0.761 | **Note:** Shoji et al. (2014) had the greatest contribution to overall heterogeneity and the greatest influence on the overall effect |

###### **Figure A7.95** Leave-One-Out Random Effects Model for Studies Reporting Bilateral Coloboma


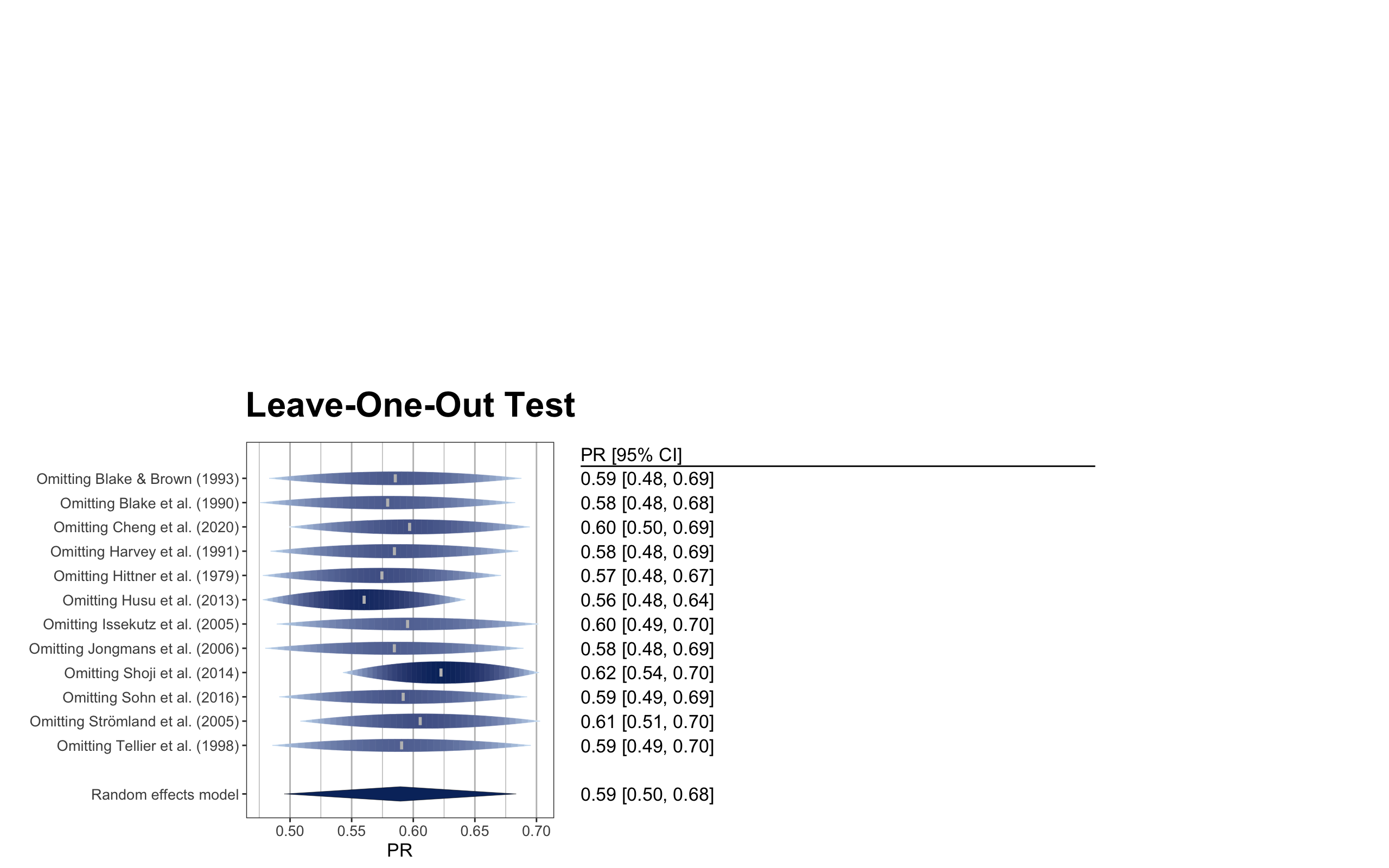


**Note:** Leave one out analysis indicating that no single study is exerting a disproportional influence on the pooled prevalence estimate

## **Bilateral Choanal Atresia**

###### **Figure A7.96** QQ Plot of The Distribution of Study Effects and Theoretical Quantities Based on A Normal Distribution Under the Random Effects Model for Studies Reporting Bilateral Choanal Atresia

**Note:** Visual inspection of the *QQ* plot suggests an approximate normal distribution of study effects for the 12 studies reporting bilateral choanal atresia in CHARGE Syndrome. On this basis the DerSimonian-Laird estimate was used to calculate between studies variance in the random-effects model.

###### **Figure A7.97** Random Effects Models of The Pooled Prevalence Estimate for Studies Reporting Bilateral Choanal Atresia in CHARGE Syndrome

**Note:** The pooled prevalence estimate for bilateral choanal atresia in CHARGE syndrome is 36% (95% CI, 23-48%; permuted p-value = 0.001; k = 12) with high heterogeneity (I^2^ = 92%). Random-effects model calculated using the inverse variance method and the DerSimonian-Laird estimator for τ^2^. Rosenthal Fail-safe N = 1224 suggests that the observed effect is robust to potential publication biases.

| **Figure A7.98** Funnel Plot of Standard Error by Prevalence of Bilateral Choanal Atresia Following the Trim and Fill Procedure | **Figure A7.99** Baujat Plot of Contribution to Heterogeneity by Influence on Overall Effect for Studies Reporting Bilateral Choanal Atresia |
| --- | --- |
|  |  |
| **Note:** Publication bias [small study effect] was identified (Egger’s test p = 0.011). The trim and fill procedure did not impute any missing studies. | **Note:** Asher et al. (1990) had the greatest contribution to overall heterogeneity and the greatest influence on the overall effect |

###### **Figure A7.100** Leave-One-Out Random Effects Model for Studies Reporting Bilateral Choanal Atresia


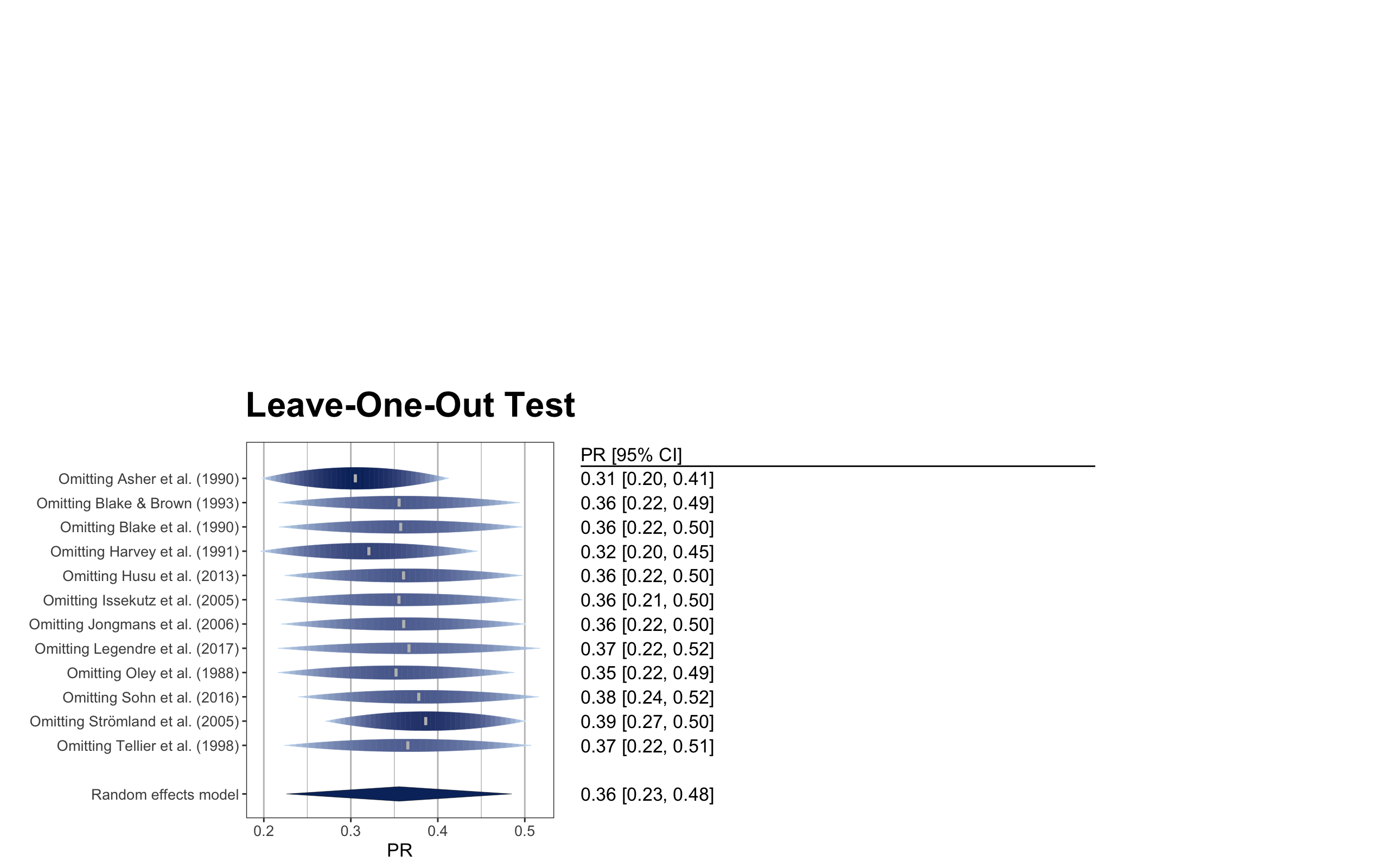


**Note:** Leave one out analysis indicating that no single study is exerting a disproportional influence on the pooled prevalence estimate

## Patent Ductus Arteriosus

###### **Figure A7.101** QQ Plot of The Distribution of Study Effects and Theoretical Quantities Based on A Normal Distribution Under the Random Effects Model for Studies Reporting Patent Ductus Arteriosus

**Note:** Visual inspection of the *QQ* plot suggests an approximate normal distribution of study effects for the 12 studies reporting patent ductus arteriosus in CHARGE Syndrome. On this basis the DerSimonian-Laird estimate was used to calculate between studies variance in the random-effects model.

###### **Figure A7.102** Random Effects Models of The Pooled Prevalence Estimate for Studies Reporting Patent Ductus Arteriosus in CHARGE Syndrome

**Note:** The pooled prevalence estimate for patent ductus arteriosus in CHARGE syndrome is 33% (95% CI, 25-41%; permuted p-value = 0.001; k = 12) with moderate heterogeneity (I^2^ = 73%). Random-effects model calculated using the inverse variance method and the DerSimonian-Laird estimator for τ^2^. Rosenthal Fail-safe N = 942 suggests that the observed effect is robust to potential publication biases.

| **Figure A7.103** Funnel Plot of Standard Error by Prevalence of Patent Ductus Arteriosus Following the Trim and Fill Procedure | **Figure A7.104** Baujat Plot of Contribution to Heterogeneity by Influence on Overall Effect for Studies Reporting Patent Ductus Arteriosus |
| --- | --- |
|  |  |
| **Note:** Publication bias [small study effect] was identified (Egger’s test p = 0.041). Using the trim and fill procedure it was estimated that 3 (SE = 2.37) studies were missing on the left side. Adjusted estimate = 28%, (95% CI = 20-37%, p = <.001; τ2 = 0.017, I2 = 74%). | **Note:** Sohn et al. (2016) had the greatest contribution to overall heterogeneity and the greatest influence on the overall effect |

###### **Figure A7.105** Leave-One-Out Random Effects Model for Studies Reporting Patent Ductus Arteriosus


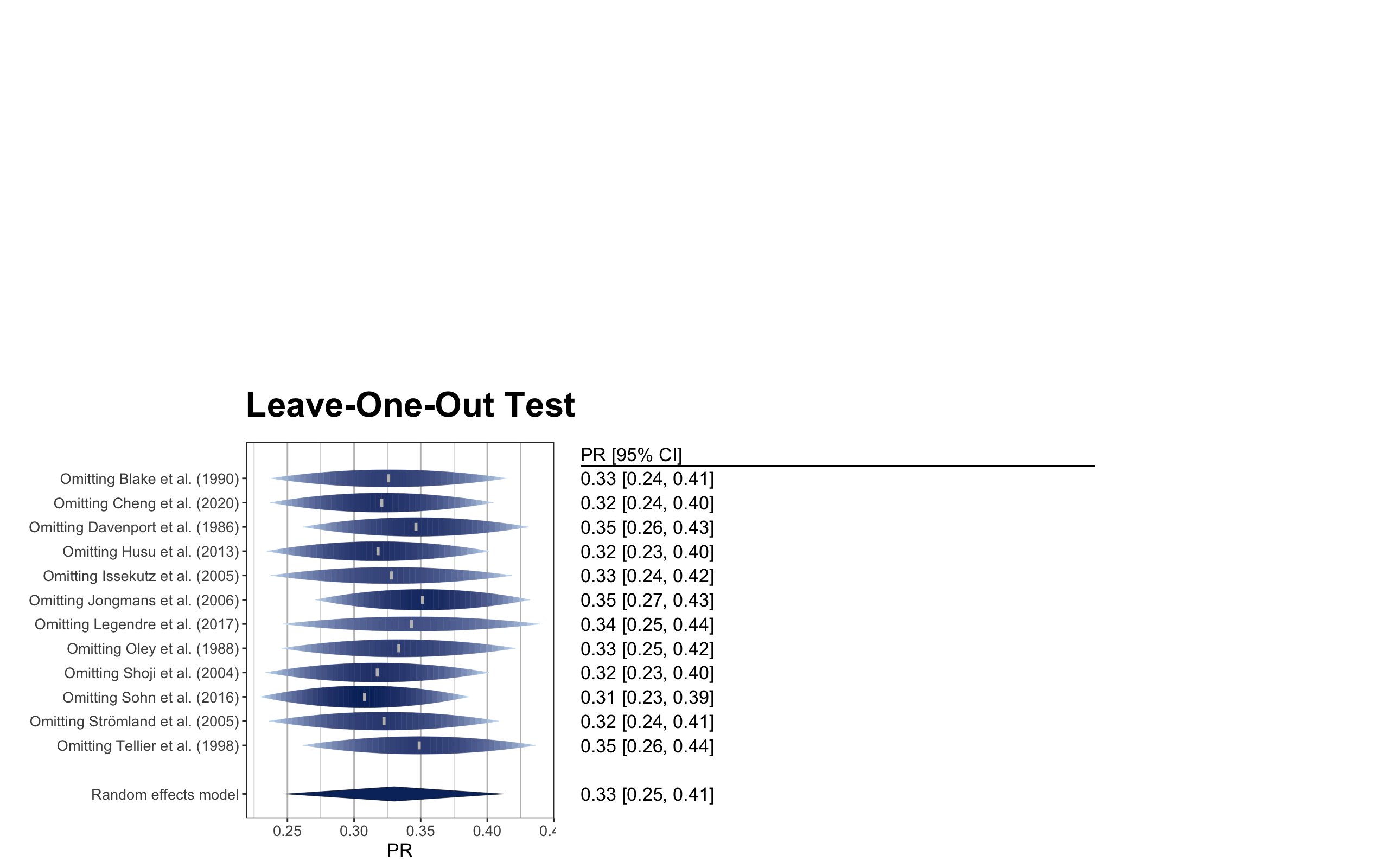


**Note:** Leave one out analysis indicating that no single study is exerting a disproportional influence on the pooled prevalence estimate.

## **Atrial Septal Defect**

###### **Figure A7.106** QQ Plot of The Distribution of Study Effects and Theoretical Quantities Based on A Normal Distribution Under the Random Effects Model for Studies Reporting Atrial Septal Defect

**Note:** Visual inspection of the *QQ* plot suggests an approximate normal distribution of study effects for the 12 studies reporting atrial septal defect in CHARGE Syndrome. On this basis the DerSimonian-Laird estimate was used to calculate between studies variance in the random-effects model.

###### **Figure A7.107** Random Effects Models of The Pooled Prevalence Estimate for Studies Reporting Atrial Septal Defect in CHARGE Syndrome

**Note:** The pooled prevalence estimate for atrial septal defect in CHARGE syndrome is 20% (95% CI, 14-27%; permuted p-value = 0.007; k = 12) with moderate heterogeneity (I^2^ = 64%). Random-effects model calculated using the inverse variance method and the DerSimonian-Laird estimator for τ^2^. Rosenthal Fail-safe N = 460 suggests that the observed effect is robust to potential publication biases.

| **Figure A7.108** Funnel Plot of Standard Error by Prevalence of Atrial Septal Defect | **Figure A7.109** Baujat Plot of Contribution to Heterogeneity by Influence on Overall Effect for Studies Reporting Atrial Septal Defect |
| --- | --- |
|  |  |
| **Note:** Visual inspection of the funnel plot conforms to normal expectations and there is weak evidence of substantial publication bias (Egger’s test p = 0.239). | **Note:** Studies in the top right quartile have the greatest contribution to overall heterogeneity and the greatest influence on the overall effect. Legendre et al. (2017) had the greatest contribution to overall heterogeneity and Husu et al. (2013) had the greatest influence on the overall effect. |

###### **Figure A7.110** Leave-One-Out Random Effects Model for Studies Reporting Atrial Septal Defect


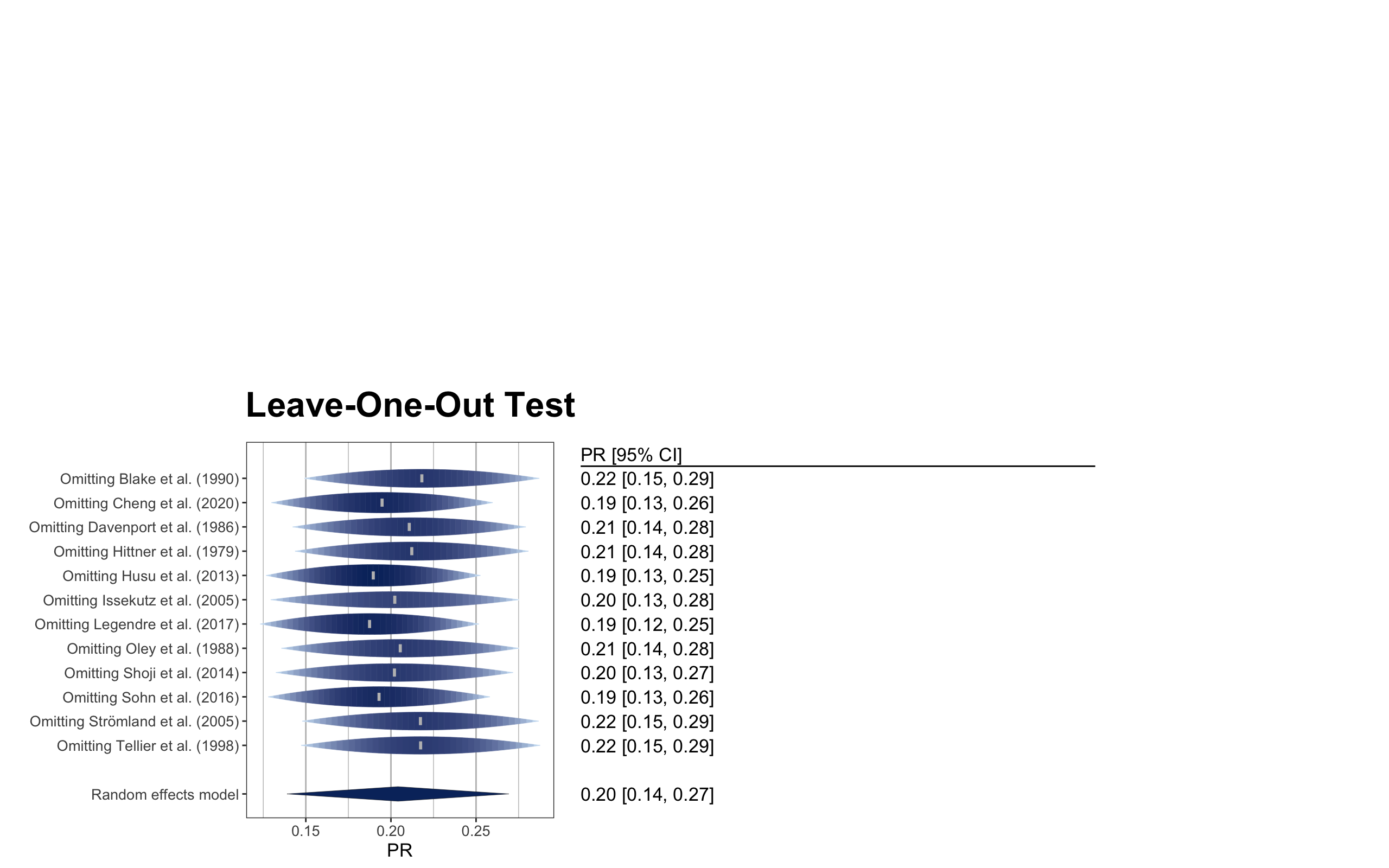


**Note:** Leave one out analysis indicating that no single study is exerting a disproportional influence on the pooled prevalence estimate.

## **Ventricular septal defect**

###### **Figure A7.111** QQ Plot of The Distribution of Study Effects and Theoretical Quantities Based on A Normal Distribution Under the Random Effects Model for Studies Reporting Ventricular Septal Defect

**Note:** Visual inspection of the *QQ* plot suggests an approximate normal distribution of study effects for the 11 studies reporting ventricular septal defect in CHARGE Syndrome. On this basis the DerSimonian-Laird estimate was used to calculate between studies variance in the random-effects model.

###### **Figure A7.112** Random Effects Models of The Pooled Prevalence Estimate for Studies Reporting Ventricular Septal Defect in CHARGE Syndrome

**Note:** The pooled prevalence estimate for ventricular septal defect in CHARGE syndrome is 17% (95% CI, 11-23%; permuted p-value = 0.001; k = 11) with moderate heterogeneity (I^2^ = 58%). Random-effects model calculated using the inverse variance method and the DerSimonian-Laird estimator for τ^2^. Rosenthal Fail-safe N = 333 suggests that the observed effect is robust to potential publication biases.

| **Figure A7.113** Funnel Plot of Standard Error by Prevalence of Ventricular Septal Defect | **Figure A7.114** Baujat Plot of Contribution to Heterogeneity by Influence on Overall Effect for Studies Reporting Ventricular Septal Defect |
| --- | --- |
|  |  |
| **Note:** Visual inspection of the funnel plot conforms to normal expectations and there is weak evidence of substantial publication bias (Egger’s test p = 0.055). | **Note:** Studies in the top right quartile have the greatest contribution to overall heterogeneity and the greatest influence on the overall effect. Issekutz et al. (2005) had the greatest contribution to overall heterogeneity and Tellier et al. (1998) had the greatest influence on the overall effect. |

###### **Figure A7.115** Leave-One-Out Random Effects Model for Studies Reporting Ventricular Septal Defect


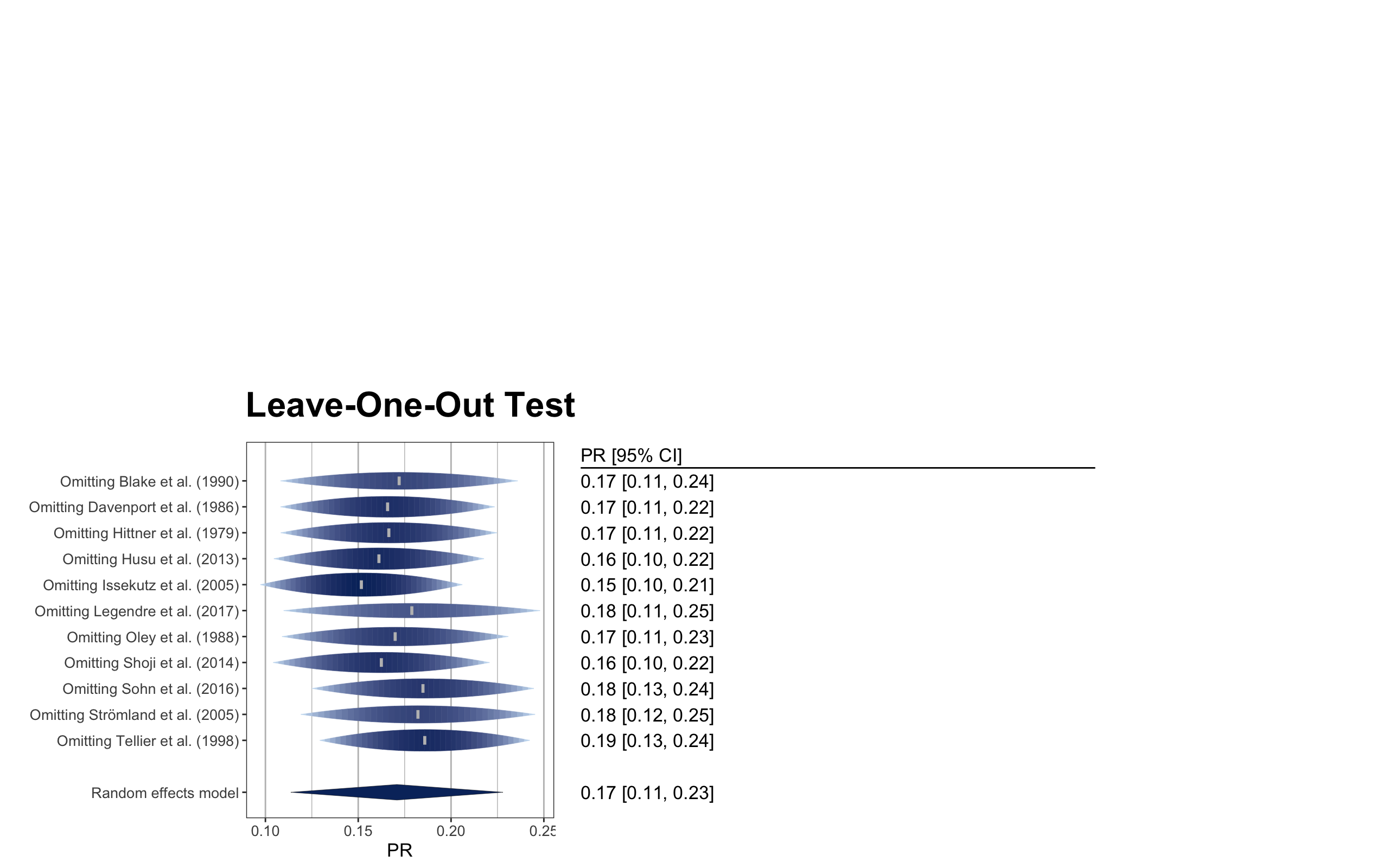


**Note:** Leave one out analysis indicating that no single study is exerting a disproportional influence on the pooled prevalence estimate

## **Cerebellar Anomalies**

###### **Figure A7.116** QQ Plot of The Distribution of Study Effects and Theoretical Quantities Based on A Normal Distribution Under the Random Effects Model for Studies Reporting Cerebellar Anomalies

**Note:** Visual inspection of the *QQ* plot suggests an approximate normal distribution of study effects for the 11 studies reporting cerebellar anomalies in CHARGE Syndrome. On this basis the DerSimonian-Laird estimate was used to calculate between studies variance in the random-effects model.

###### **Figure A7.117** Random Effects Models of The Pooled Prevalence Estimate for Studies Reporting Cerebellar Anomalies in CHARGE Syndrome


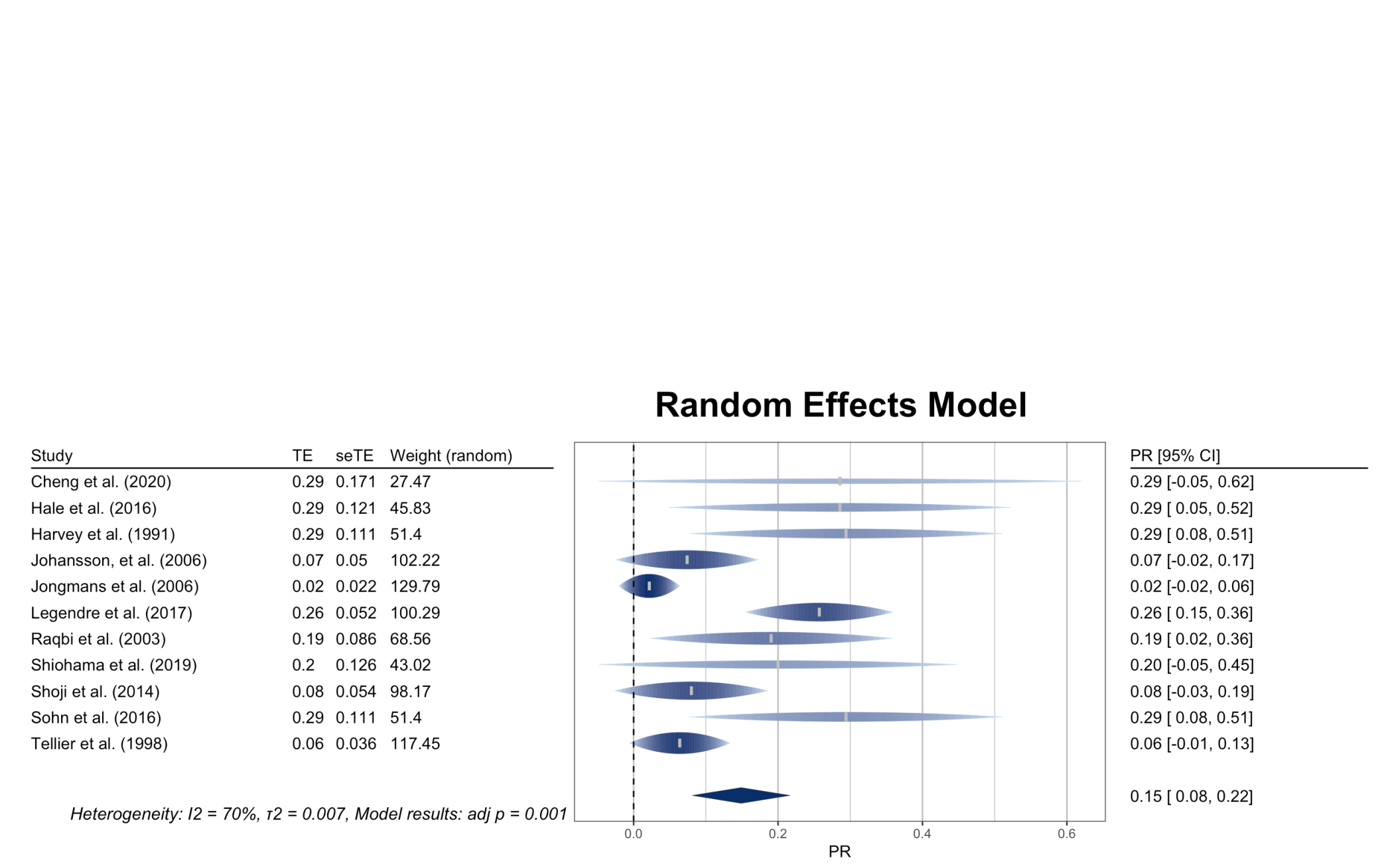


**Note:** The pooled prevalence estimate for cerebellar anomalies in CHARGE syndrome is 15% (95% CI, 8-22%; permuted p-value = 0.001; k = 11) with moderate heterogeneity (I^2^ = 70%). Random-effects model calculated using the inverse variance method and the DerSimonian-Laird estimator for τ^2^. Rosenthal Fail-safe N = 199 suggests that the observed effect is robust to potential publication biases.

| **Figure A7.118** Funnel Plot of Standard Error by Prevalence of Cerebellar Anomalies Following the Trim and Fill Procedure | **Figure A7.119** Baujat Plot of Contribution to Heterogeneity by Influence on Overall Effect for Studies Reporting Cerebellar Anomalies |
| --- | --- |
|  |  |
| **Note:** Publication bias [small study effect] was identified (Egger’s test p = 0.002). Using the trim and fill procedure it was estimated that 4 (SE = 2.15) studies were missing on the left side. Adjusted estimate = 11%, (95% *CI* = 5-17%, *p* = <.001; τ^2^ = 0.007, I^2^ = 65%). | **Note:** Studies in the top right quartile have the greatest contribution to overall heterogeneity and the greatest influence on the overall effect. Legendre et al. (2017) had the greatest contribution to overall heterogeneity and Jongmans et al. (2006) had the greatest influence on the overall effect. |

###### **Figure A7.120** Leave-One-Out Random Effects Model for Studies Reporting Cerebellar Anomalies


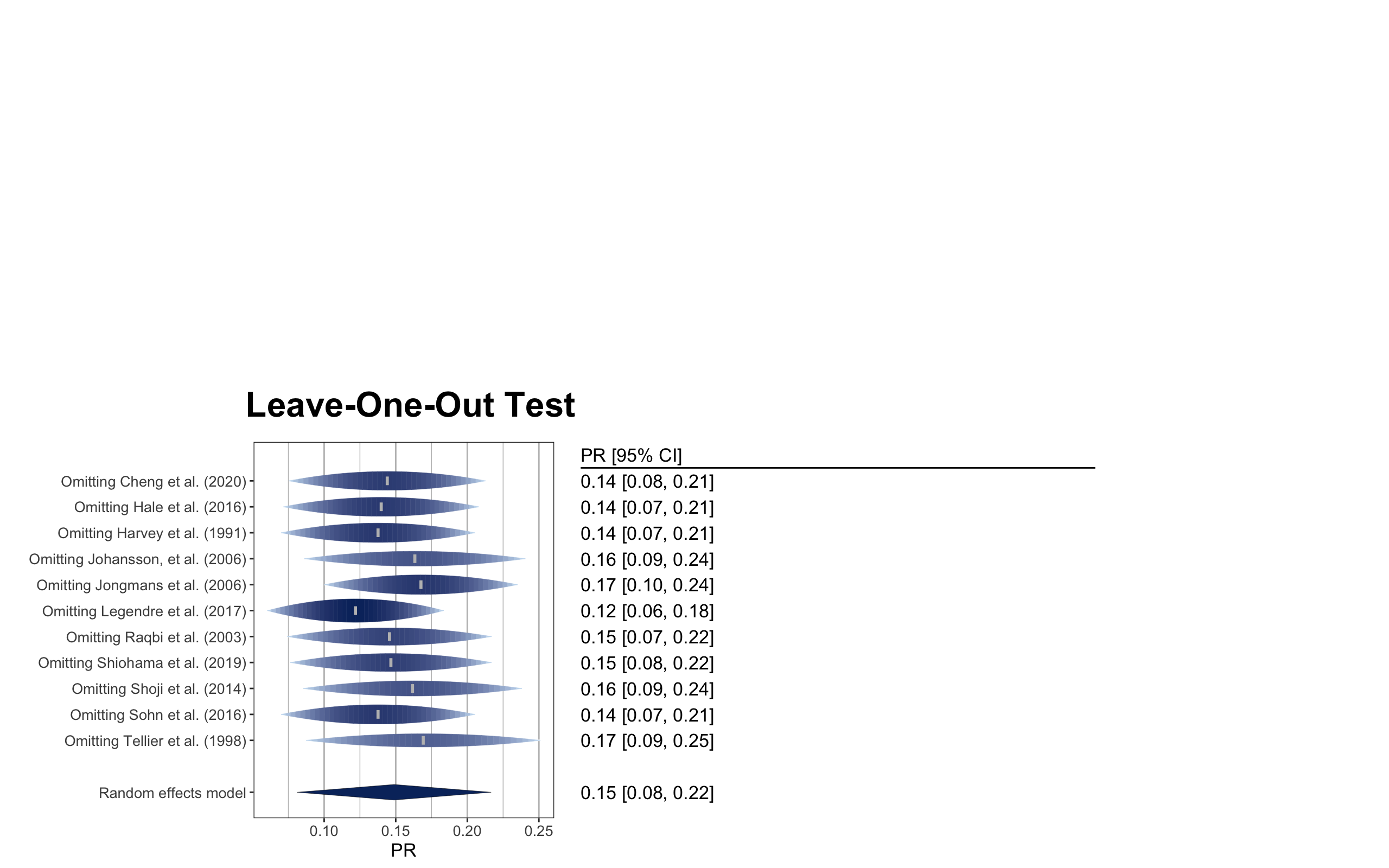


**Note:** Leave one out analysis indicating that no single study is exerting a disproportional influence on the pooled prevalence estimate.

## **Corpus Callosum**

###### **Figure A7.121** QQ Plot of The Distribution of Study Effects and Theoretical Quantities Based on A Normal Distribution Under the Random Effects Model for Studies Reporting Corpus Callosum Anomalies

**Note:** Visual inspection of the *QQ* plot suggests an approximate normal distribution of study effects for the 10 studies reporting corpus callosum anomalies in CHARGE Syndrome. On this basis the DerSimonian-Laird estimate was used to calculate between studies variance in the random-effects model.

###### **Figure A7.122** Random Effects Models of The Pooled Prevalence Estimate for Studies Reporting Corpus Callosum Anomalies in CHARGE Syndrome

**Note:** The pooled prevalence estimate for corpus callosum anomalies in CHARGE syndrome is 8% (95% CI, 4-12%; permuted p-value = 0.003; k = 10) with low heterogeneity (I^2^ = 48%). Random-effects model calculated using the inverse variance method and the DerSimonian-Laird estimator for τ^2^. Rosenthal Fail-safe N = 100 suggests that the observed effect is robust to potential publication biases.

| **Figure A7.123** Funnel Plot of Standard Error by Prevalence of Corpus Callosum Anomalies Following the Trim and Fill Procedure | **Figure A7.124** Baujat Plot of Contribution to Heterogeneity by Influence on Overall Effect for Studies Reporting Corpus Callosum Anomalies |
| --- | --- |
|  |  |
| **Note:** Publication bias [small study effect] was identified (Egger’s test p = 0.004). Using the trim and fill procedure it was estimated that 5 (SE = 1.97) studies were missing on the left side. Adjusted estimate = 4%, (95% CI = 0-8%, p = 0.001; τ2 = 0.004, I2 = 63%). | **Note:** Legendre et al. (2017) had the greatest contribution to overall heterogeneity and the greatest influence on the overall effect. |

###### **Figure A7.125** Leave-One-Out Random Effects Model for Studies Reporting Corpus Callosum Anomalies


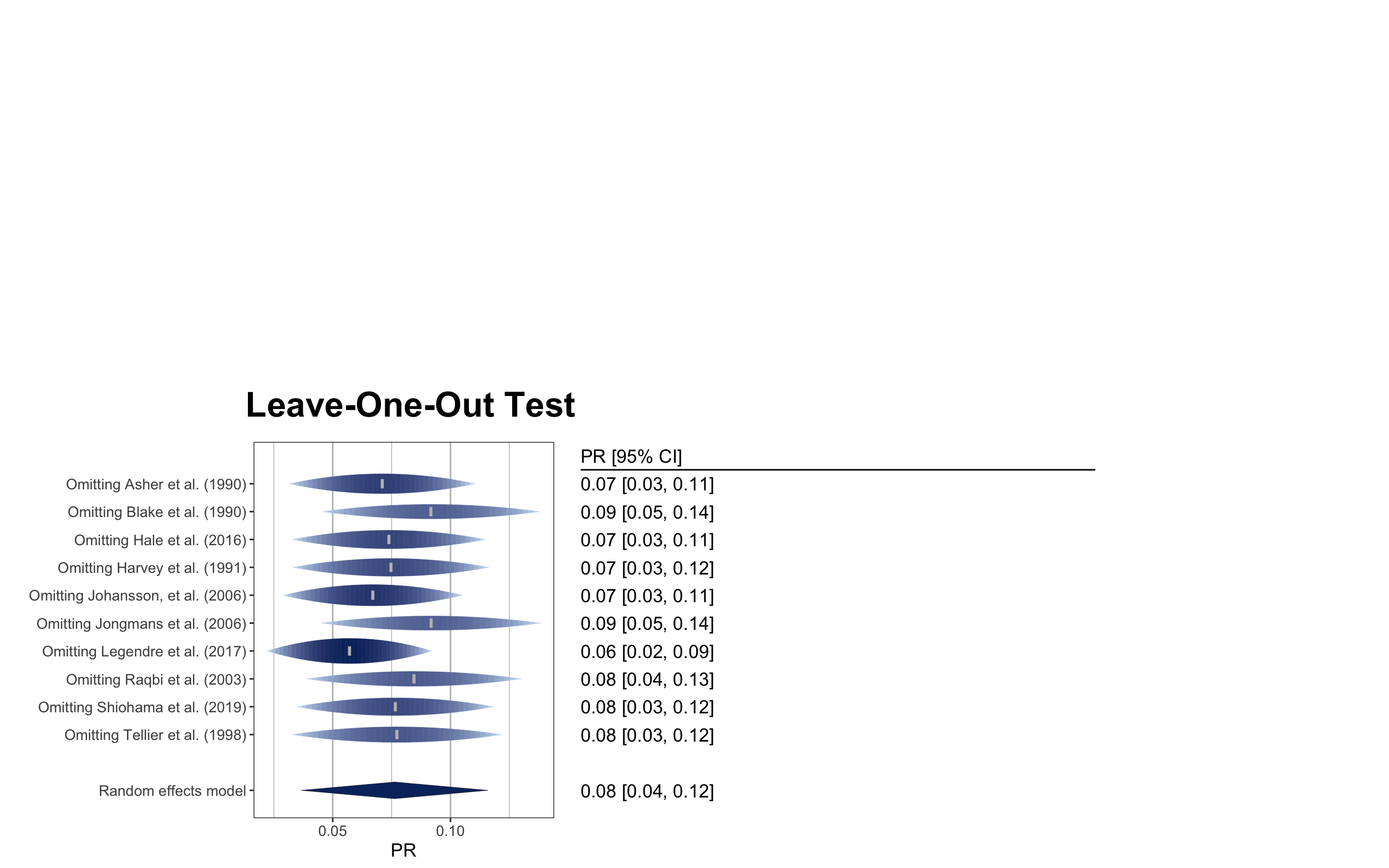


**Note:** Leave one out analysis indicating that no single study is exerting a disproportional influence on the pooled prevalence estimate

## **Severe or Profound Hearing Impairment**

###### **Figure A7.126** QQ Plot of The Distribution of Study Effects and Theoretical Quantities Based on A Normal Distribution Under the Random Effects Model for Studies Reporting Severe or Profound Hearing Impairment

**Note:** Visual inspection of the *QQ* plot suggests an approximate normal distribution of study effects for the 19 studies reporting severe or profound hearing impairment in CHARGE Syndrome. On this basis the DerSimonian-Laird estimate was used to calculate between studies variance in the random-effects model.

###### **Figure A7.127** Random Effects Models of The Pooled Prevalence Estimate for Studies Reporting Severe or Profound Hearing Impairment in CHARGE Syndrome

**Note:** The pooled prevalence estimate for severe or profound hearing impairment in CHARGE syndrome is 50% (95% CI, 40-59%; permuted p-value = 0.001; k = 19) with low heterogeneity (I^2^ = 48%). Random-effects model calculated using the inverse variance method and the DerSimonian-Laird estimator for τ^2^. Rosenthal Fail-safe N = 4750 suggests that the observed effect is robust to potential publication biases.

| **Figure A7.128** Funnel Plot of Standard Error by Prevalence of Severe or Profound Hearing Impairment | **Figure A7.129** Baujat Plot of Contribution to Heterogeneity by Influence on Overall Effect for Studies Reporting Severe or Profound Hearing Impairment |
| --- | --- |
|  |  |
| **Note:** Visual inspection of the funnel plot conforms to normal expectations and there is weak evidence of substantial publication bias (Egger’s test p = 0.126). | **Note:** Thelin & Fussner (2005) had the greatest contribution to overall heterogeneity and the greatest influence on the overall effect. |

###### **Figure A7.130** Leave-One-Out Random Effects Model for Studies Reporting Severe or Profound Hearing Impairment


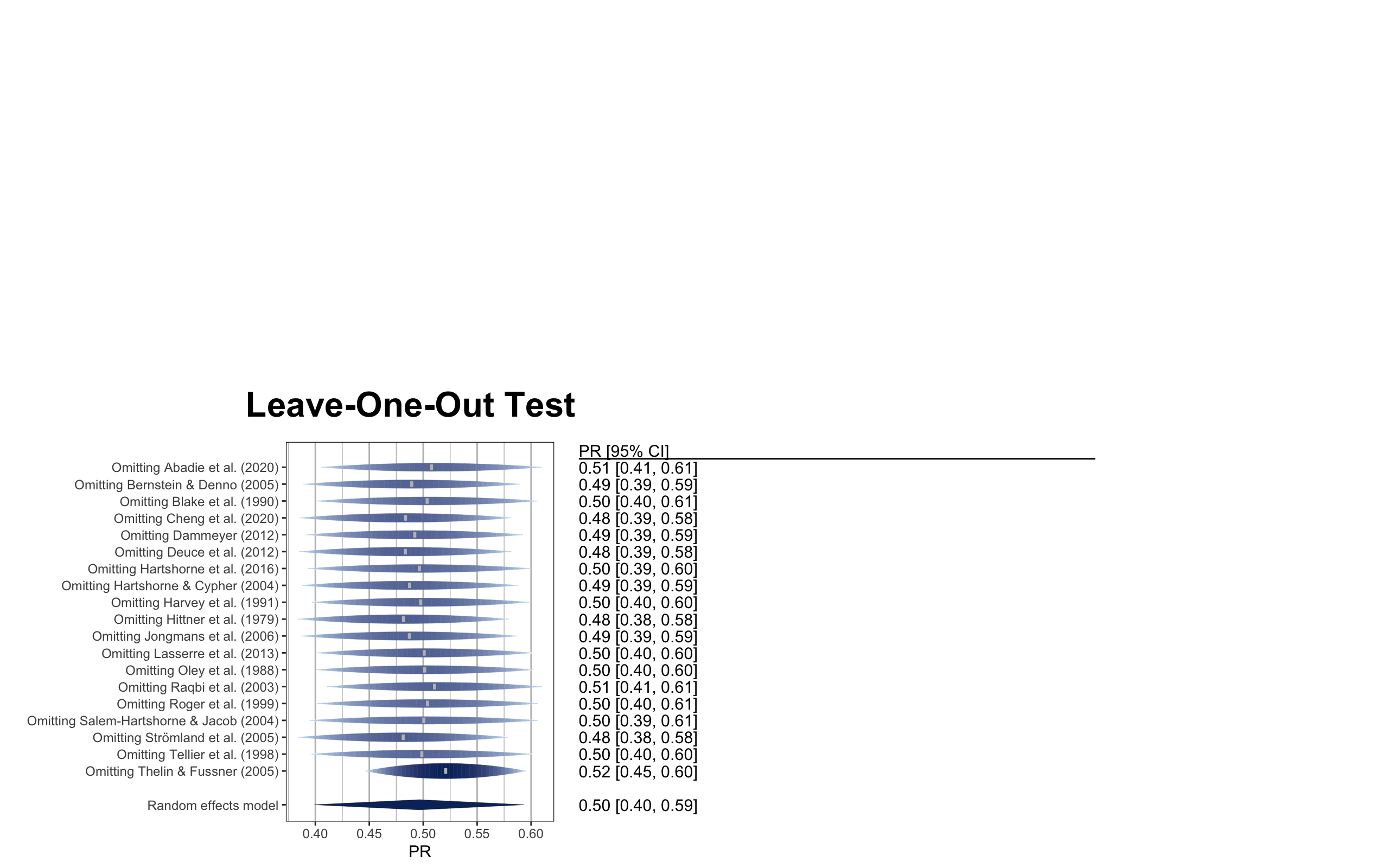


**Note:** Leave one out analysis indicating that no single study is exerting a disproportional influence on the pooled prevalence estimate

# **Appendix 8**. Incidence of physical characteristics and conditions associated with CHARGE syndrome reported in eligible studies

##### **Table A8.1** Physical Characteristics and Conditions Associated with CHARGE Syndrome in the Eligible Studies

|  | **Otitis Media** | **Gross Motor Difficulties** | **Gastro-esophageal Reflux** | **Micrognathia** | **Skeletal anomalies** | **Laryngeal anomalies** | **Microcephaly** | **Urinary tract anomaly** |
| --- | --- | --- | --- | --- | --- | --- | --- | --- |
| **Abadie et al. [1]** | 74% (37/50) | - | - | - | 57% (27/47) | - | 36% (20/55) | 32% (17/53) |
| **Asher et al. [2]** | - | - | - | - | - | - | - | - |
| **Bernstein & Denno [3]** | - | - | - | - | - | - | - | - |
| **Blake & Brown [4]** | - | - | - | - | 32% (12/38) | - | - | 31% (12/39) |
| **Blake et al. [5]** | - | - | 45% (20/44) | - | 40% (20/50) | 26% (13/50) | 18% (9/50) | 24% (12/50) |
| **Blake et al. [6]** | - | - | - | - | 27% (8/30) | - | - | 30% (9/30) |
| **Cheng et al. [7]** | - | 8% (1/12) | 20% (2/10) | 8% (1/12) | 89% (8/9) | 58% (7/12) | - | 11% (1/10) |
| **Dammeyer [8]** | - | 100% (17/17) | - | - | - | - | - | - |
| **Davenport et al. [9]** | 78% (7/9) | - | - | - | - | - | 44% (4/9) | 50% (3/6) |
| **Deuce et al. [10]** | - | 93% (40/43) | 45% (20/44) | - | - | - | - | 9% (4/44) |
| **Deuce [11]** | - | 92% (48/52) | - | - | - | - | - | - |
| **Dobbelsteyn et al. [12]** | - | - | 89% (32/36) | - | - | - | - | - |
| **Hale et al. [13]** | - | - | - | - | 86% (12/14) | - | - | 52% (11/21) |
| **Hartshorne et al. [14]** | - | 85% (74/87) | - | - | 31% (27/87) | - | - | 30% (26/87) |
| **Hartshorne et al. [15]** | - | - | - | - | 40% (21/53) | - | - | - |
| **Hartshorne & Cypher [16]** | 71% (71/100) | 63% (63/100) | - | - | 27% (27/100) | - | - | 23% (23/100) |
| **Hartshorne et al. [17]** | - | - | - | - | - | - | - | - |
| **Harvey et al. [18]** | 67% (6/9) | - | - | 65% (11/17) | 47% (8/17) | - | - | 55% (6/11) |
| **Hittner et al. [19]** | - | - | - | - | 30% (3/10) | - | - | 20% (2/10) |
| **Hsu et al. [20]** | 65% (13/20) | - | - | - | - | - | - | - |
| **Husu et al. [21]** | - | 100% (13/13) | - | - | 54% (7/13) | - | - | 23% (3/13) |
| **Issekutz et al. [22]** | - | 44% (7/16) | 70% (7/10) | - | 35% (27/77) | - | - | 36% (28/77) |
| **Johansson, et al. [23]** | - | - | - | - | - | - | - | - |
| **Jongmans et al. [24]** | - | 100% (46/46) | - | - | 22% (10/46) | - | - | 20% (9/46) |
| **Lasserre et al. [25]** | - | 100% (8/8) | - | - | - | - | - | - |
| **Legendre et al. [26]** | - | - | 72% (75/104) | - | 49% (50/103) | - | 29% (31/108) | 29% (30/103) |
| **Oley et al. [27]** | - | - | - | 10% (2/20) | 50% (10/20) | 5% (1/20) | 25% (5/20) | 30% (6/20) |
| **Raqbi et al. [28]** | - | - | - | - | 33% (7/21) | 22% (2/9) | 19% (4/21) | 20% (4/21) |
| **Reda & Hartshorne [29]** | 52% (13/25) | 96% (24/25) | - | - | 16% (4/25) | - | - | 48% (12/25) |
| **Roger et al. [30]** | - | - | 80% (36/46) | 39% (11/28) | 13% (6/45) | 58% (26/45) | - | - |
| **Salem-Hartshorne & Jacob [31]** | - | - | - | - | - | - | - | - |
| **Shiohama et al. [32]** | - | - | - | - | - | - | - | - |
| **Shoji et al. [33]** | - | - | 32% (8/25) | - | 8% (2/25) | 4% (1/25) | - | 12% (3/25) |
| **Smith et al. [34]** | - | - | - | - | - | - | - | - |
| **Sohn et al. [35]** | - | - | - | - | 28% (5/18) | 22% (4/18) | - | 11% (2/18) |
| **Souriau et al. [36]** | - | 86% (44/51) | - | - | 53% (36/68) | - | - | - |
| **Strömland et al. [37]** | - | 88% (21/24) | - | - | 42% (13/31) | - | 29% (9/31) | 13% (4/31) |
| **Tellier et al. [38]** | 91% (10/11) | - | - | 81% (38/47) | 53% (25/47) | 60% (18/30) | - | 19% (9/47) |
| **Thelin & Fussner** [**39]** | - | 36 (10/28) | - | - | - | - | - | - |
| **Wessels et al. [40]** | - | - | - | - | 50% (4/8) | - | - | 45% (5/11) |
| **Wincent et al. [41]** | - | - | - | - | - | - | - | - |
| **Wulffaert et al. [42]** | - | - | - | - | - | - | - | - |

**References:**

1. Abadie V, Hamiaux P, Ragot S, Legendre M, Malecot G, Burtin A, et al. Should autism spectrum disorder be considered part of CHARGE syndrome? A cross-sectional study of 46 patients. Orphanet J Rare Dis. 2020;15. doi:10.1186/s13023-020-01421-9.

2. Asher BF, McGill TJ, Kaplan L, Friedman EM, Healy GB. Airway complications in CHARGE association. Arch Otolaryngol - Head Neck Surg. 1990;116:15. http://ovidsp.ovid.com/ovidweb.cgi?T=JS&PAGE=reference&D=emed4&NEWS=N&AN=20045463.

3. Bernstein V, Denno LS. Repetitive behaviors in CHARGE syndrome: differential diagnosis and treatment options. Am J Med Genet. 2005;133A:232–9. doi:http://dx.doi.org/10.1002/ajmg.a.30542.

4. Blake KD, Brown D. CHARGE association looking at the future--the voice of a family support group. Child Care Health Dev. 1993;19:395–409. http://ovidsp.ovid.com/ovidweb.cgi?T=JS&PAGE=reference&D=emed5&NEWS=N&AN=127249873.

5. Blake KD, Russell-Eggitt IM, Morgan DW, Ratcliffe JM, Wyse RK. Who’s in CHARGE? Multidisciplinary management of patients with CHARGE association. Arch Dis Child. 1990;65:217–23. http://ovidsp.ovid.com/ovidweb.cgi?T=JS&PAGE=reference&D=emed4&NEWS=N&AN=20057815.

6. Blake KD, Salem-Hartshorne N, Daoud MA, Gradstein J. Adolescent and adult issues in CHARGE syndrome. Clin Pediatr (Phila). 2005;44:151–9. doi:http://dx.doi.org/10.1177/000992280504400207.

7. Cheng SSW, Luk H-M, Chan DKH, Lo IFM. CHARGE syndrome in nine patients from China. Am J Med Genet Part A. 2020;182:15–9. doi:10.1002/ajmg.a.61398.

8. Dammeyer J. Development and characteristics of children with Usher syndrome and CHARGE syndrome. Int J Pediatr Otorhinolaryngol. 2012;76:1292–6. doi:http://dx.doi.org/10.1016/j.ijporl.2012.05.021.

9. Davenport SLH, Hefner MA, Mitchell JA. The spectrum of clinical features in CHARGE syndrome. Clin Genet. 1986;29:298–310.

10. Deuce G, Howard S, Rose S, Fuggle C. A study of CHARGE Syndrome in the UK. Br J Vis Impair. 2012;30:91–100. doi:http://dx.doi.org/10.1177/0264619612443883.

11. Deuce G. The education of learners with CHARGE syndrome. Br J Spec Educ. 2017;44:376–93. doi:http://dx.doi.org/10.1111/1467-8578.12183.

12. Dobbelsteyn C, Peacocke SD, Blake K, Crist W, Rashid M. Feeding difficulties in children with CHARGE syndrome: Prevalence, risk factors, and prognosis. Dysphagia. 2008;23:127–35. doi:http://dx.doi.org/10.1007/s00455-007-9111-6.

13. Hale CL, Niederriter AN, Green GE, Martin DM. Atypical phenotypes associated with pathogenic CHD7 variants and a proposal for broadening CHARGE syndrome clinical diagnostic criteria. Am J Med Genet Part A. 2016;170:344–54.

14. Hartshorne TS, Heussler HS, Dailor AN, Williams GL, Papadopoulos D. Sleep disturbances in CHARGE syndrome: Types and relationships with behavior and caregiver well-being. Dev Med Child Neurol. 2009;51:143–50. doi:http://dx.doi.org/10.1111/j.1469-8749.2008.03146.x.

15. Hartshorne N, Hudson A, MacCuspie J, Kennert B, Nacarato T, Hartshorne T, et al. Quality of life in adolescents and adults with CHARGE syndrome. Am J Med Genet Part A. 2016;170:2012–21. doi:http://dx.doi.org/10.1002/ajmg.a.37769.

16. Hartshorne TS, Cypher AD. Challenging behavior in CHARGE Syndrome. Ment Heal Asp Dev Disabil. 2004;7:41–52. http://ovidsp.ovid.com/ovidweb.cgi?T=JS&PAGE=reference&D=emed8&NEWS=N&AN=38738142.

17. Hartshorne TS, Grialou TL, Parker KR. Autistic-like behavior in CHARGE syndrome. Am J Med Genet. 2005;133 A:257–61. doi:http://dx.doi.org/10.1002/ajmg.a.30545.

18. Harvey AS, Leaper PM, Bankier A. CHARGE association: Clinical manifestations and developmental outcome. Am J Med Genet. 1991;39:48–55. http://ovidsp.ovid.com/ovidweb.cgi?T=JS&PAGE=reference&D=med3&NEWS=N&AN=1867265.

19. Hittner HM, Hirsch NJ, Kreh GM, Rudolph AJ. Colobomatous microphthalmia, heart disease, hearing loss, and mental retardation-a syndrome. J Pediatr Ophthalmol Strabismus. 1979;16:122–8. https://search.proquest.com/docview/1490658587?pq-origsite=gscholar.

20. Hsu P, Ma A, Barnes EH, Wilson M, Hoefsloot LH, Rinne T, et al. The Immune Phenotype of Patients with CHARGE Syndrome. J Allergy Clin Immunol Pract. 2016;4:96. doi:http://dx.doi.org/10.1016/j.jaip.2015.09.004.

21. Husu E, Hove HD, Farholt S, Bille M, Tranebjaerg L, Vogel I, et al. Phenotype in 18 Danish subjects with genetically verified CHARGE syndrome. Clin Genet. 2013;83:125–34. doi:http://dx.doi.org/10.1111/j.1399-0004.2012.01884.x.

22. Issekutz KA, Graham Jr JM, Prasad C, Smith IM, Blake KD. An epidemiological analysis of CHARGE syndrome: Preliminary results from a Canadian study. Am J Med Genet. 2005;133 A:309–17. doi:http://dx.doi.org/10.1002/ajmg.a.30560.

23. Johansson M, Rastam M, Billstedt E, Danielsson S, Strömland K, Miller M, et al. Autism spectrum disorders and underlying brain pathology in CHARGE association. Dev Med Child Neurol. 2006;48:40–50. doi:http://dx.doi.org/10.1017/S0012162206000090.

24. Jongmans MCJ, Admiraal RJ, van der Donk KP, Vissers LELM, Baas AF, Kapusta L, et al. CHARGE syndrome: the phenotypic spectrum of mutations in the CHD7 gene. J Med Genet. 2006;43:306–14. doi:10.1136/jmg.2005.036061.

25. Lasserre E, Vaivre-Douret L, Abadie V. Psychomotor and cognitive impairments of children with CHARGE syndrome: common and variable features. Child Neuropsychol. 2013;19:449–65. doi:https://dx.doi.org/10.1080/09297049.2012.690372.

26. Legendre M, Abadie V, Attie-Bitach T, Philip N, Busa T, Bonneau D, et al. Phenotype and genotype analysis of a French cohort of 119 patients with CHARGE syndrome. Am J Med Genet Part C Semin Med Genet. 2017;175:417–30. doi:https://dx.doi.org/10.1002/ajmg.c.31591.

27. Oley CA, Baraitser M, Grant DB. A reappraisal of the CHARGE association. J Med Genet. 1988;25:147–56. http://ovidsp.ovid.com/ovidweb.cgi?T=JS&PAGE=reference&D=emed4&NEWS=N&AN=18082760.

28. Raqbi F, Le Bihan C, Morrisseau-Durand MP, Dureau P, Lyonnet S, Abadie V. Early prognostic factors for intellectual outcome in CHARGE syndrome. Dev Med Child Neurol. 2003;45:483–8. doi:10.1017/S0012162203000896.

29. Reda NM, Hartshorne TS. Attachment, bonding, and parental stress in CHARGE syndrome. Ment Heal Asp Dev Disabil. 2008;11:10–21. http://ovidsp.ovid.com/ovidweb.cgi?T=JS&PAGE=reference&D=psyc6&NEWS=N&AN=2008-02562-002.

30. Roger G, Morisseau-Durand MP, Van Den Abbeele T, Nicollas R, Triglia JM, Narcy P, et al. The CHARGE association: The role of tracheotomy. Arch Otolaryngol - Head Neck Surg. 1999;125:33–8. doi:http://dx.doi.org/10.1001/archotol.125.1.33.

31. Salem-Hartshorne N, Jacob S. Chracteristics and development of children with CHARGE association/syndrome. J Early Interv. 2004;26:292–301.

32. Shiohama T, McDavid J, Levman J, Takahashi E. Quantitative brain morphological analysis in CHARGE syndrome. NeuroImage Clin. 2019;23:101866.

33. Shoji Y, Ida S, Etani Y, Yamada H, Kayatani F, Suzuki Y, et al. Endocrinological characteristics of 25 Japanese patients with CHARGE syndrome. Clin Pediatr Endocrinol. 2014;23:45–51. doi:http://dx.doi.org/10.1297/cpe.23.45.

34. Smith IM, Nichols SL, Issekutz K, Blake K. Behavioral profiles and symptoms of autism in CHARGE syndrome: preliminary Canadian epidemiological data. Am J Med Genet. 2005;133A:248–56. doi:http://dx.doi.org/10.1002/ajmg.a.30544.

35. Sohn YB, Ko JM, Shin CH, Yang SW, Chae J-H, Lee K-A. Cerebellar vermis hypoplasia in CHARGE syndrome: Clinical and molecular characterization of 18 unrelated Korean patients. J Hum Genet. 2016;61:235–9. doi:http://dx.doi.org/10.1038/jhg.2015.135.

36. Souriau J, Gimenes M, Blouin C, Benbrik I, Benbrik E, Churakowskyi A, et al. CHARGE syndrome: Developmental and behavioral data. Am J Med Genet. 2005;133 A:278–81. doi:http://dx.doi.org/10.1002/ajmg.a.30549.

37. Strömland K, Sjögreen L, Johansson M, Joelsson BME, Miller M, Danielsson S, et al. CHARGE association in Sweden: Malformations and functional deficits. Am J Med Genet. 2005;133 A:331–9.

38. Tellier AL, Cormier-Daire V, Abadie V, Amiel J, Sigaudy S, Bonnet D, et al. CHARGE syndrome: Report of 47 cases and review. Am J Med Genet. 1998;76:402–9. doi:http://dx.doi.org/10.1002/%28SICI%291096-8628%2819980413%2976:5%3C402::AID-AJMG7%3E3.0.CO;2-O.

39. Thelin JW, Fussner JC. Factors related to the development of communication in CHARGE syndrome. Am J Med Genet. 2005;133 A:282–90. doi:http://dx.doi.org/10.1002/ajmg.a.30550.

40. Wessels K, Bohnhorst B, Luhmer I, Morlot S, Bohring A, Jonasson J, et al. Novel CHD7 mutations contributing to the mutation spectrum in patients with CHARGE syndrome. Eur J Med Genet. 2010;53:280–5. doi:10.1016/j.ejmg.2010.07.002.

41. Wincent J, Holmberg E, Strömland K, Soller M, Mirzaei L, Djureinovic T, et al. CHD7 mutation spectrum in 28 Swedish patients diagnosed with CHARGE syndrome. Clin Genet. 2008;74:31–8. doi:https://dx.doi.org/10.1111/j.1399-0004.2008.01014.x.

42. Wulffaert J, Scholte EM, Dijkxhoorn YM, Bergman JEH, van Ravenswaaij-Arts CMA, van Berckelaer-Onnes IA. Parenting stress in CHARGE syndrome and the relationship with child characteristics. J Dev Phys Disabil. 2009.

# **Appendix 9**. Forest plots and sensitivity analysis for physical characteristics and conditions

## **Recurrent Otitis Media**

###### **Figure A9.1** QQ Plot of The Distribution of Study Effects and Theoretical Quantities Based on A Normal Distribution Under the Random Effects Model for Studies Reporting Recurrent Otitis Media

**Note:** Visual inspection of the *QQ* plot suggests an approximate normal distribution of study effects for the 8 studies reporting recurrent otitis media in CHARGE Syndrome. On this basis the DerSimonian-Laird estimate was used to calculate between studies variance in the random-effects model.

###### **Figure A9.2** Random Effects Models of The Pooled Prevalence Estimate for Studies Reporting Recurrent Otitis Media in CHARGE Syndrome

**Note:** The pooled prevalence estimate for recurrent otitis media in CHARGE syndrome is 74% (95% CI, 67-80%; permuted p-value = 0.007; k = 8) with moderate heterogeneity (I^2^ = 40%). Random-effects model calculated using the inverse variance method and the DerSimonian-Laird estimator for τ^2^

###### **Figure A9.3** Baujat Plot of Contribution to Heterogeneity by Influence on Overall Effect for Studies Reporting Recurrent Otitis Media

**Note:** Reda & Hartshorne (2008) had the greatest contribution to overall heterogeneity and the greatest influence on the overall effect.

###### **Figure A9.4** Leave-One-Out Random Effects Model for Studies Reporting Recurrent Otitis Media


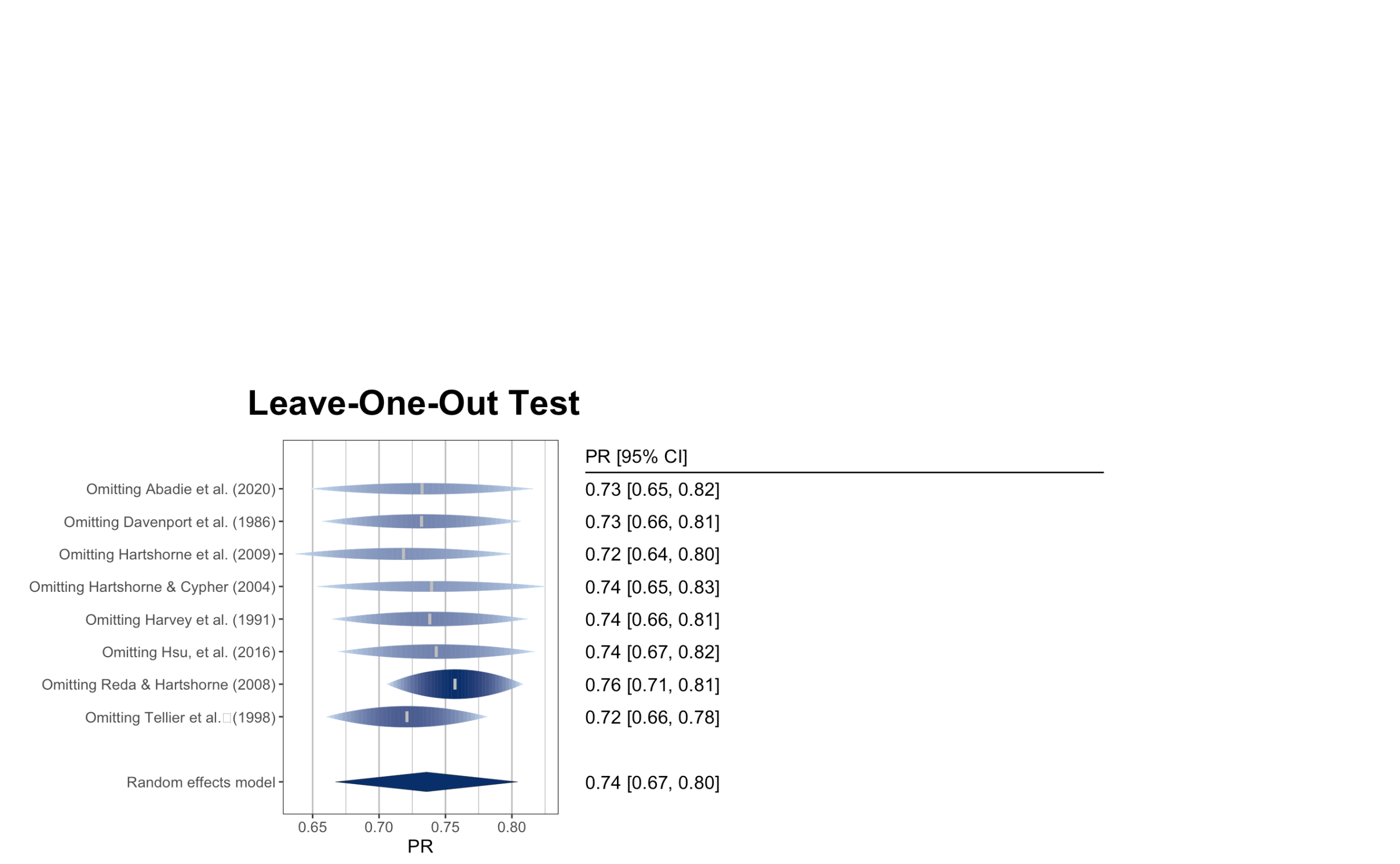


**Note:** Leave one out analysis indicating that no single study is exerting a disproportional influence on the pooled prevalence estimate

## **Gross Motor Difficulties**

###### **Figure A9.5** QQ Plot of The Distribution of Study Effects and Theoretical Quantities Based on A Normal Distribution Under the Random Effects Model for Studies Reporting Gross Motor Difficulties

**Note:** Visual inspection of the *QQ* plot suggests an approximate normal distribution of study effects for the 10 studies reporting gross motor difficulties in CHARGE Syndrome. On this basis the DerSimonian-Laird estimate was used to calculate between studies variance in the random-effects model.

###### **Figure A9.6** Random Effects Models of The Pooled Prevalence Estimate for Studies Reporting Recurrent Gross Motor Difficulties in CHARGE Syndrome

**Note:** The pooled prevalence estimate for gross motor difficulties in CHARGE syndrome was 71% (95% CI, 57-84%; permuted *p-*value = 0.004; *k* = 10) with high heterogeneity (I^2^ = 95%). Random-effects model calculated using the inverse variance method and the DerSimonian-Laird estimator for τ^2^. Rosenthal Fail-safe N = 8098 suggests that the observed effect is robust to potential publication biases.

| **Figure A9.7** Funnel Plot of Standard Error by Prevalence of Gross Motor Difficulties Following the Trim and Fill Procedure | **Figure A9.8** Baujat Plot of Contribution to Heterogeneity by Influence on Overall Effect for Studies Reporting Gross Motor Difficulties |
| --- | --- |
|  |  |
| **Note:** Publication bias [small study effect] was identified (Egger’s test p = 0.011). The trim and fill procedure did not impute any missing studies. | **Note:** Cheng et al. (2020) had the greatest contribution to overall heterogeneity and the greatest influence on the overall effect. |

###### **Figure A9.9** Leave-One-Out Random Effects Model for Studies Reporting Gross Motor Difficulties


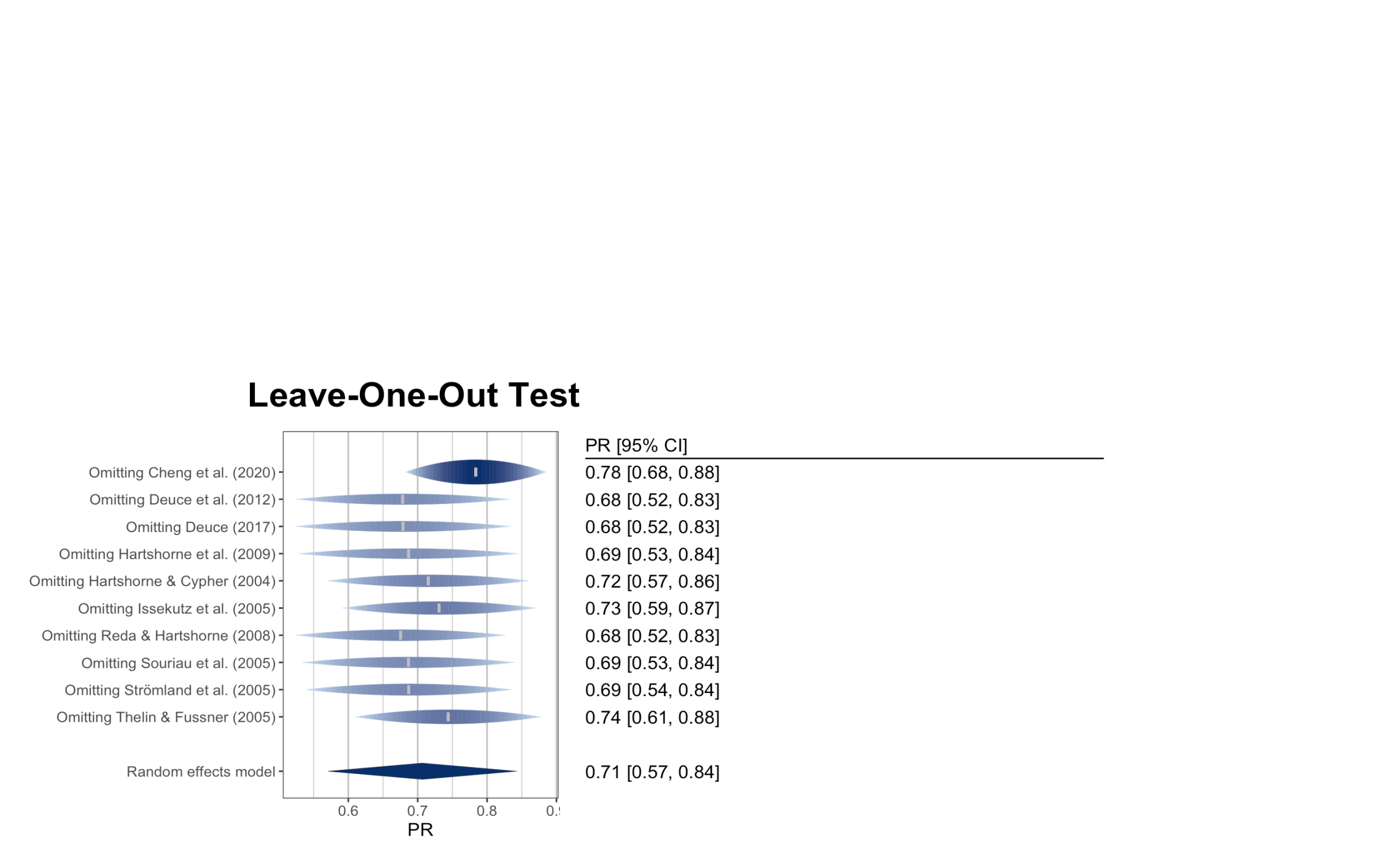


**Note:** Leave one out analysis indicating that no single study is exerting a disproportional influence on the pooled prevalence estimate

## **Gastroesophageal Reflux**

###### **Figure A9.10** QQ Plot of The Distribution of Study Effects and Theoretical Quantities Based on A Normal Distribution Under the Random Effects Model for Studies Reporting Gastroesophageal Reflux

**Note:** Visual inspection of the QQ plot suggests an approximate normal distribution of study effects for the 8 studies reporting gastroesophageal reflux in CHARGE Syndrome. On this basis the DerSimonian-Laird estimate was used to calculate between studies variance in the random-effects model.

###### **Figure A9.11** Random Effects Models of The Pooled Prevalence Estimate for Studies Reporting Recurrent Gastroesophageal Reflux in CHARGE Syndrome

**Note:** The pooled prevalence estimate for gastroesophageal reflux in CHARGE syndrome was 58% (95% CI, 42-73%; permuted *p-*value = 0.007; *k* = 8) with high heterogeneity (I^2^ = 90%). Random-effects model calculated using the inverse variance method and the DerSimonian-Laird estimator for τ^2^.

###### **Figure A9.12** Baujat Plot of Contribution to Heterogeneity by Influence on Overall Effect for Studies Reporting Gastroesophageal Reflux

**Note:** Cheng et al. (2020) had the greatest contribution to overall heterogeneity and the greatest influence on the overall effect.

###### **Figure A9.13** Leave-One-Out Random Effects Model for Studies Reporting Gastroesophageal Reflux


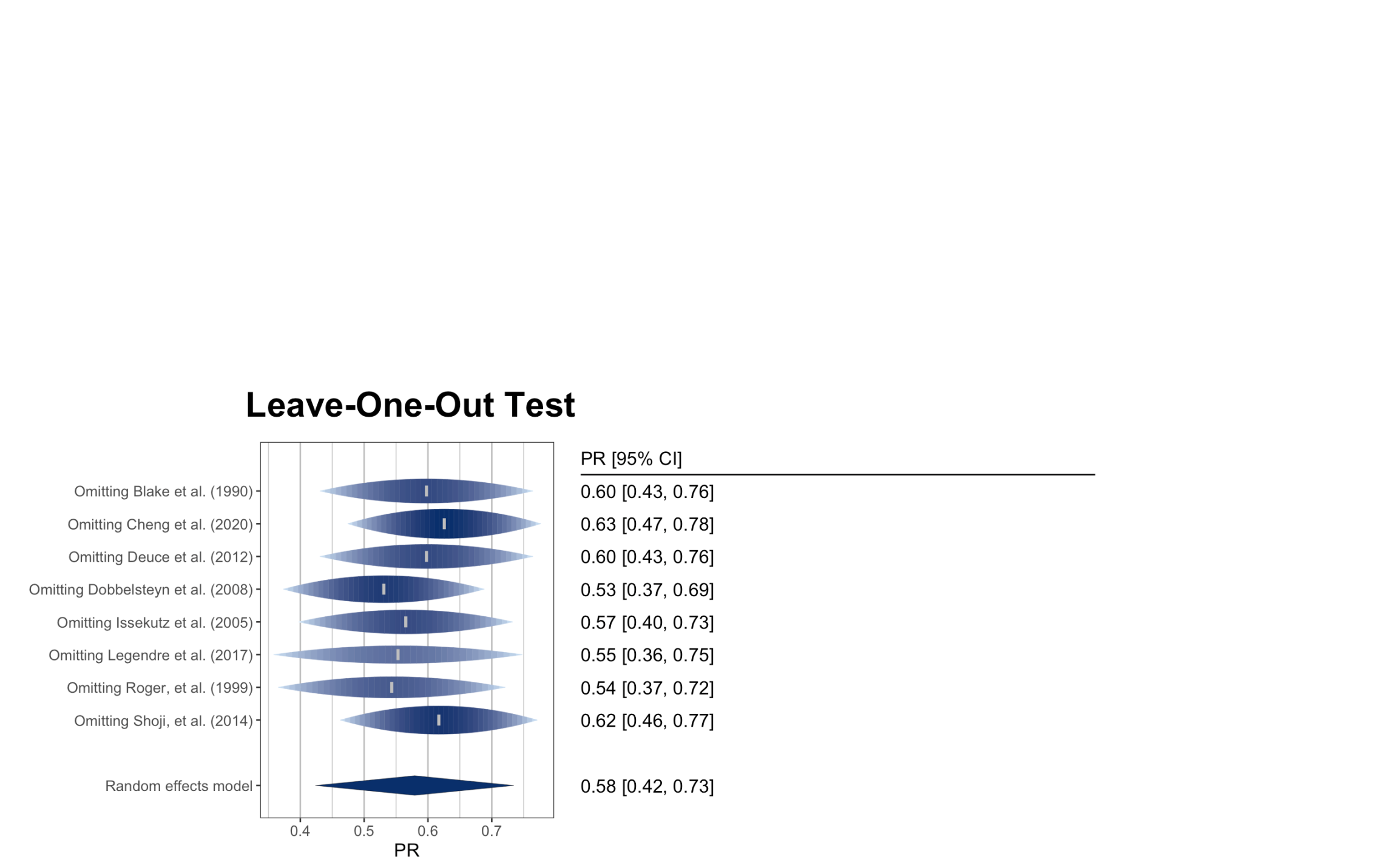


**Note:** Leave one out analysis indicating that no single study is exerting a disproportional influence on the pooled prevalence estimate

## **Micrognathia**

###### **Figure A9.14** QQ Plot of The Distribution of Study Effects and Theoretical Quantities Based on A Normal Distribution Under the Random Effects Model for Studies Reporting Micrognathia

**Note:** Visual inspection of the *QQ* plot suggests a non-Gaussian distribution of study effects for the 5 studies reporting micrognathia in CHARGE Syndrome. On this basis the restricted maximum likelihood estimator was used to calculate between studies variance in the random-effects model.

###### **Figure A9.15** Random Effects Models of The Pooled Prevalence Estimate for Studies Reporting Micrognathia in CHARGE Syndrome

**Note:** The random effects model for micrognathia in CHARGE syndrome suggest a non-significant pooled prevalence estimate of 41% (95% CI, 8-73%; permuted *p-*value = 0.063; *k* = 5) with high heterogeneity (I^2^ = 96%). Random-effects model calculated using the inverse variance method and the restricted maximum likelihood estimator for τ^2^

###### **Figure A9.16** QQ Plot of The Distribution of Study Effects and Theoretical Quantities Based on A Normal Distribution Under the Fixed Effects Model for Studies Reporting Micrognathia

**Note:** Revised QQ plot based on a fixed effects model: QQ plot of study effects for the 5 studies reporting on micrognathia in CHARGE Syndrome.

###### **Figure A9.17** Fixed Effects Models of The Pooled Prevalence Estimate for Studies Reporting Micrognathia in CHARGE Syndrome

**Note:** The pooled prevalence estimate for micrognathia in CHARGE syndrome was 43% (95% CI, 36-50%; p = <0.001; *k* = 5). Fixed-effects model calculated using the inverse variance method.

## **Skeletal Anomalies**

###### **Figure A9.18** QQ Plot of The Distribution of Study Effects and Theoretical Quantities Based on A Normal Distribution Under the Random Effects Model for Studies Reporting Skeletal Anomalies

**Note:** Visual inspection of the *QQ* plot suggests an approximate normal distribution of study effects for the 25 studies reporting skeletal anomalies in CHARGE Syndrome. On this basis the DerSimonian-Laird estimate was used to calculate between studies variance in the random-effects model.

###### **Figure A9.19** Random Effects Models of The Pooled Prevalence Estimate for Studies Reporting Skeletal Anomalies in CHARGE Syndrome

**Note:** The pooled prevalence estimate for skeletal anomalies in CHARGE syndrome was 39% (95% CI, 32-47%; permuted *p-*value = <0.001; *k* = 25) with high heterogeneity (I^2^ = 96%). Random-effects model calculated using the inverse variance method and the DerSimonian-Laird estimator for τ^2^. Rosenthal Fail-safe N = 5946 suggests that the observed effect is robust to potential publication biases

| **Figure A7.20** Funnel Plot of Standard Error by Prevalence of Skeletal Anomalies | **Figure A7.21** Baujat Plot of Contribution to Heterogeneity by Influence on Overall Effect for Studies Reporting Skeletal Anomalies |
| --- | --- |
|  |  |
| **Note:** Visual inspection of the funnel plot conforms to normal expectations and there is weak evidence of substantial publication bias (Egger’s test p = 0.051) | **Note:** Hale et al. (2016) and Cheng et al. (2020) had the greatest contribution to overall heterogeneity and the greatest influence on the overall effect |

###### **Figure A9.22** Leave-One-Out Random Effects Model for Studies Reporting Skeletal Anomalies


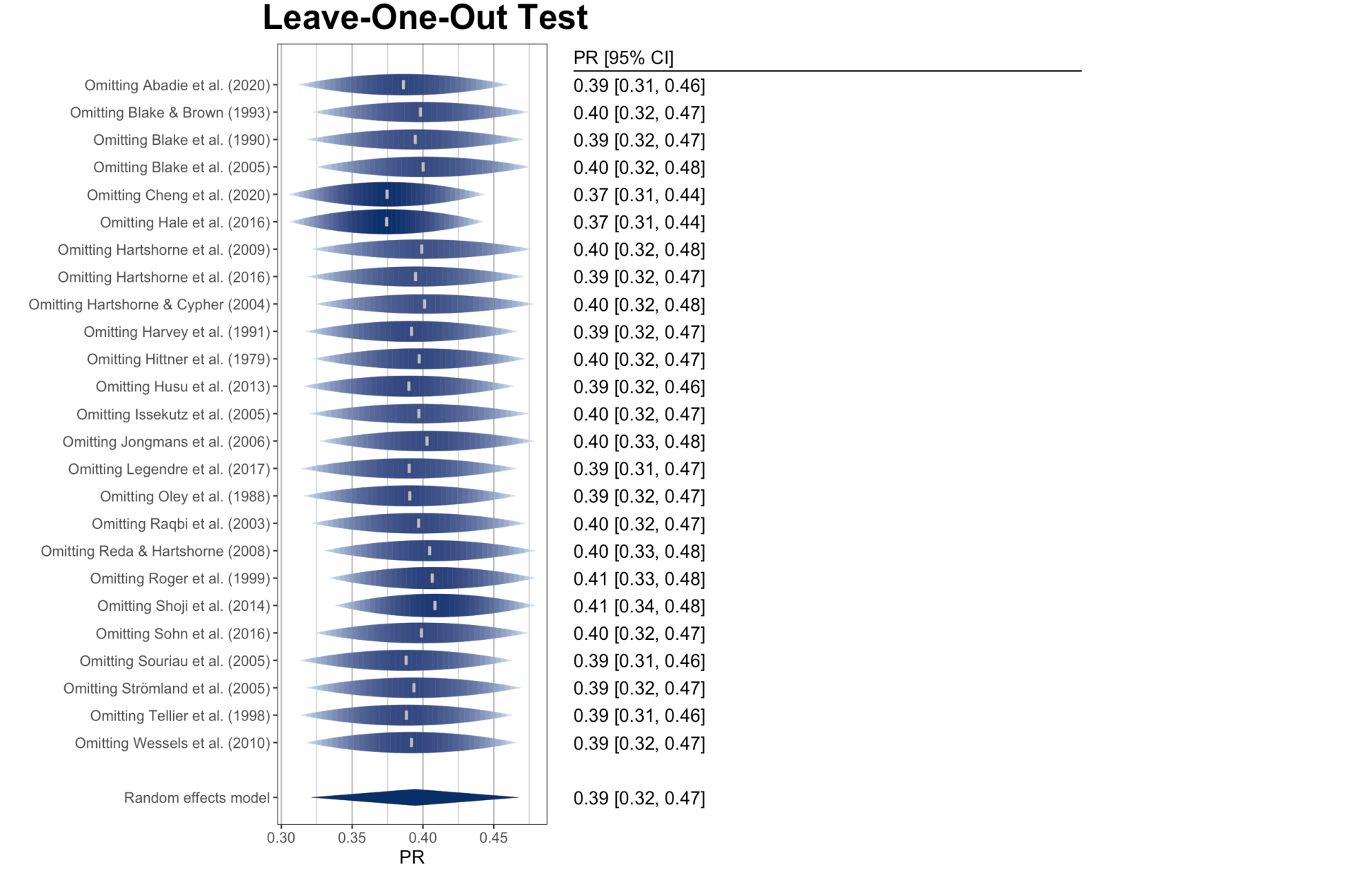


**Note:** Leave one out analysis indicating that no single study is exerting a disproportional influence on the pooled prevalence estimate

## **Laryngeal Anomalies**

###### **Figure A9.23** QQ Plot of The Distribution of Study Effects and Theoretical Quantities Based on A Normal Distribution Under the Random Effects Model for Studies Reporting Laryngeal Anomalies

**Note:** Visual inspection of the *QQ* plot suggests a non-Gaussian distribution of study effects for the 7 studies reporting laryngeal anomalies in CHARGE Syndrome. On this basis the restricted maximum likelihood estimator was used to calculate between studies variance in the random-effects model.

###### **Figure A9.24** Random Effects Models of The Pooled Prevalence Estimate for Studies Reporting Laryngeal Anomalies in CHARGE Syndrome

**Note:** The pooled prevalence estimate for laryngeal anomalies in CHARGE syndrome was 32% (95% CI, 13-51%; permuted *p-*value = 0.016; *k* = 7) with high heterogeneity (I^2^ = 93%). Random-effects model calculated using the inverse variance method and the restricted maximum likelihood estimator for τ^2^.

###### **Figure A7.25** Baujat Plot of Contribution to Heterogeneity by Influence on Overall Effect for Studies Reporting Laryngeal Anomalies

**Note:** Studies in the top right quartile have the greatest contribution to overall heterogeneity and the greatest influence on the overall effect. Roger et al. (1999) had the greatest contribution to overall heterogeneity and Shoji et al. (2014) had the greatest influence on the overall effect

###### **Figure A9.26** Leave-One-Out Random Effects Model for Studies Reporting Laryngeal Anomalies


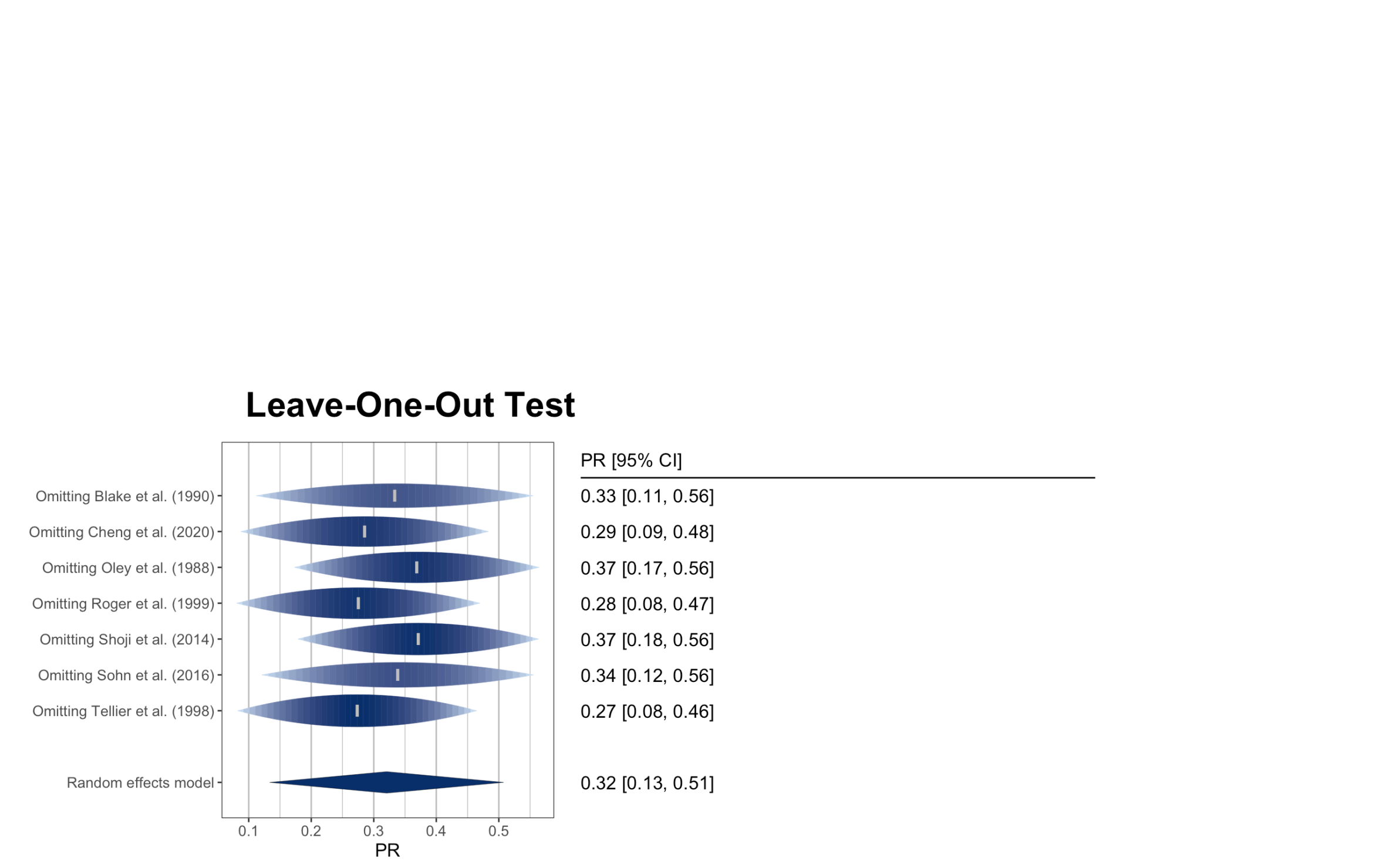


**Note:** Leave one out analysis indicating that no single study is exerting a disproportional influence on the pooled prevalence estimate

## Microcephaly

###### **Figure A9.27** QQ Plot of The Distribution of Study Effects and Theoretical Quantities Based on A Normal Distribution Under the Random Effects Model for Studies Reporting Microcephaly

**Note:** Visual inspection of the *QQ* plot suggests an approximate normal distribution of study effects for the 7 studies reporting microcephaly in CHARGE Syndrome. On this basis the DerSimonian-Laird estimate was used to calculate between studies variance in the random-effects model.

###### **Figure A9.28** Random Effects Models of The Pooled Prevalence Estimate for Studies Reporting Microcephaly in CHARGE Syndrome

**Note:** The pooled prevalence estimate for microcephaly in CHARGE syndrome was 27% (95% CI, 21-32%; permuted *p-*value = 0.016; *k* = 7) with low heterogeneity (I^2^ = 15%). Random-effects model calculated using the inverse variance method and the DerSimonian-Laird estimator for τ^2^.

###### **Figure A7.29** Baujat Plot of Contribution to Heterogeneity by Influence on Overall Effect for Studies Reporting Microcephaly

**Note:** Blake et al. (1990) had the greatest contribution to overall heterogeneity and the greatest influence on the overall effect

###### **Figure A9.30** Leave-One-Out Random Effects Model for Studies Reporting Microcephaly


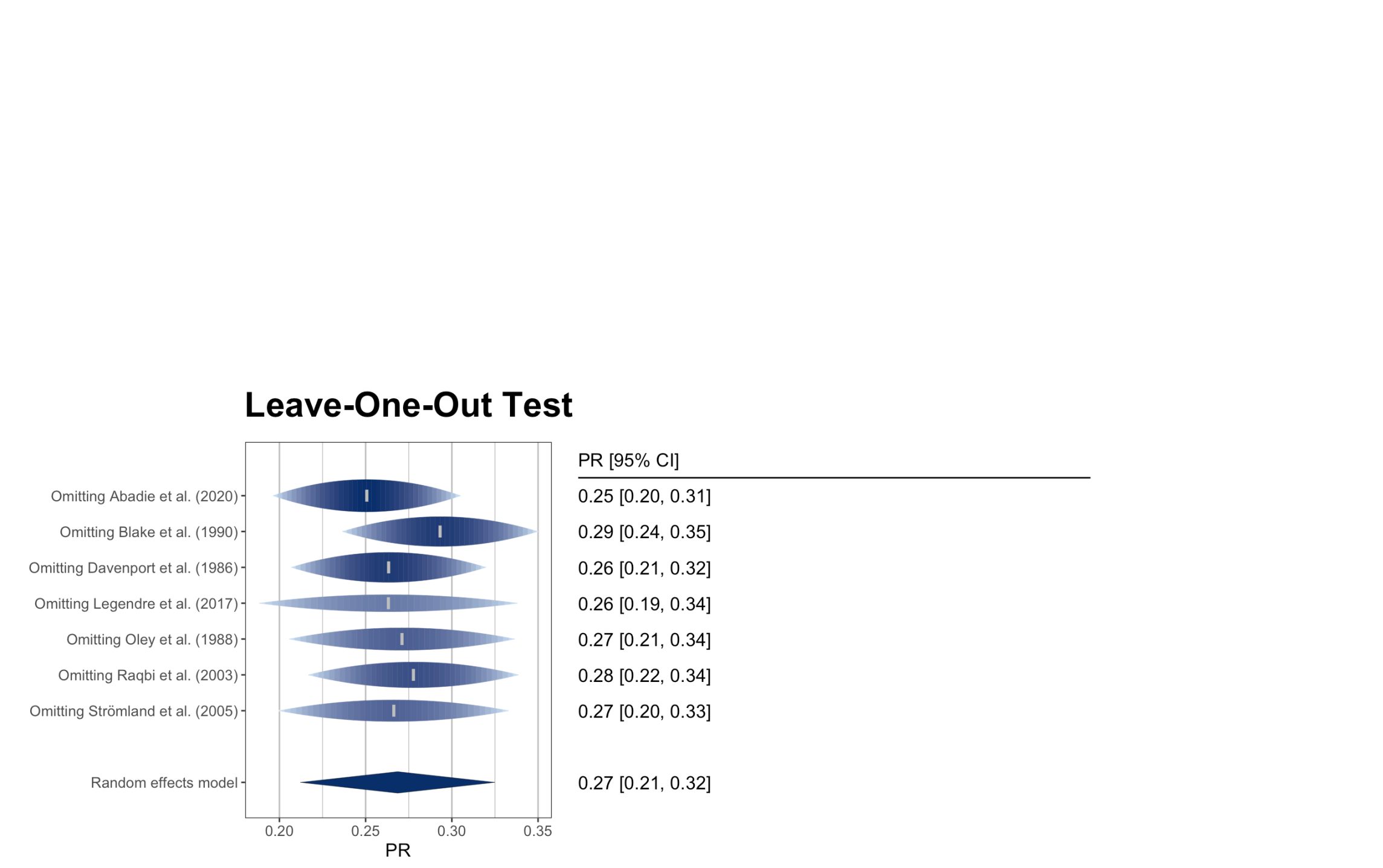


**Note:** Leave one out analysis indicating that no single study is exerting a disproportional influence on the pooled prevalence estimate

# **Appendix 10.** Details of Quality Weighted Pooled Prevalence Estimates and Subgroup and Sensitivity Analysis for Cognitive, Behavioural, Psychological and Sleep Characteristics

## **Developmental Delay**

The definition of developmental delay adopted (where stated in individual studies) was an early onset (<5 years) delay or deficit in two or more areas of functioning: cognitive skills, speech development, motor skills or social and emotional development [1]. Seven studies were not included in the meta-analysis because all participants presented with developmental delay [2–8]. Blake et al. [9] and Harvey et al. [10] reported an 82% and 71% prevalence of language delay in their respective samples, and speech impairment was reported in 81% and 100% in Johansson, et al. [11] and Oley et al. [12]. However, there was not enough data to synthesise these findings.

###### **Figure A10.1** QQ Plot of The Distribution of Study Effects and Theoretical Quantities Based on A Normal Distribution Under the Random Effects Model for Studies Reporting Developmental Delay

**Note:** Visual inspection of the *QQ* plot suggests an approximate normal distribution of study effects for the 7 studies reporting on developmental delay in CHARGE Syndrome. On this basis the DerSimonian-Laird estimate was used to calculate between studies variance in the random-effects model.

###### **Figure A10.2** Random Effects Models of The Pooled Prevalence Estimate for Studies Reporting Developmental Delay in CHARGE Syndrome

**Note:** The quality weighted pooled prevalence estimate for developmental delay in CHARGE syndrome was 84% (95% CI, 77-91%; permuted *p-*value = 0.016; *k* = 7) with moderate heterogeneity (I^2^ = 44%). Random-effects model calculated using the inverse variance method and the DerSimonian-Laird estimator for τ^2^. Rosenthal Fail-safe N = 8098 suggests that the observed effect is robust to potential publication biases

###### **Figure A10.3** Baujat Plot of Contribution to Heterogeneity by Influence on Overall Effect for Studies Reporting Developmental Delay

**Note:** Dobbelsteyn et al. (2008) had the greatest contribution to overall heterogeneity and the greatest influence on the overall effect

###### **Figure A10.4** Leave-One-Out Random Effects Model for Studies Reporting Developmental Delay


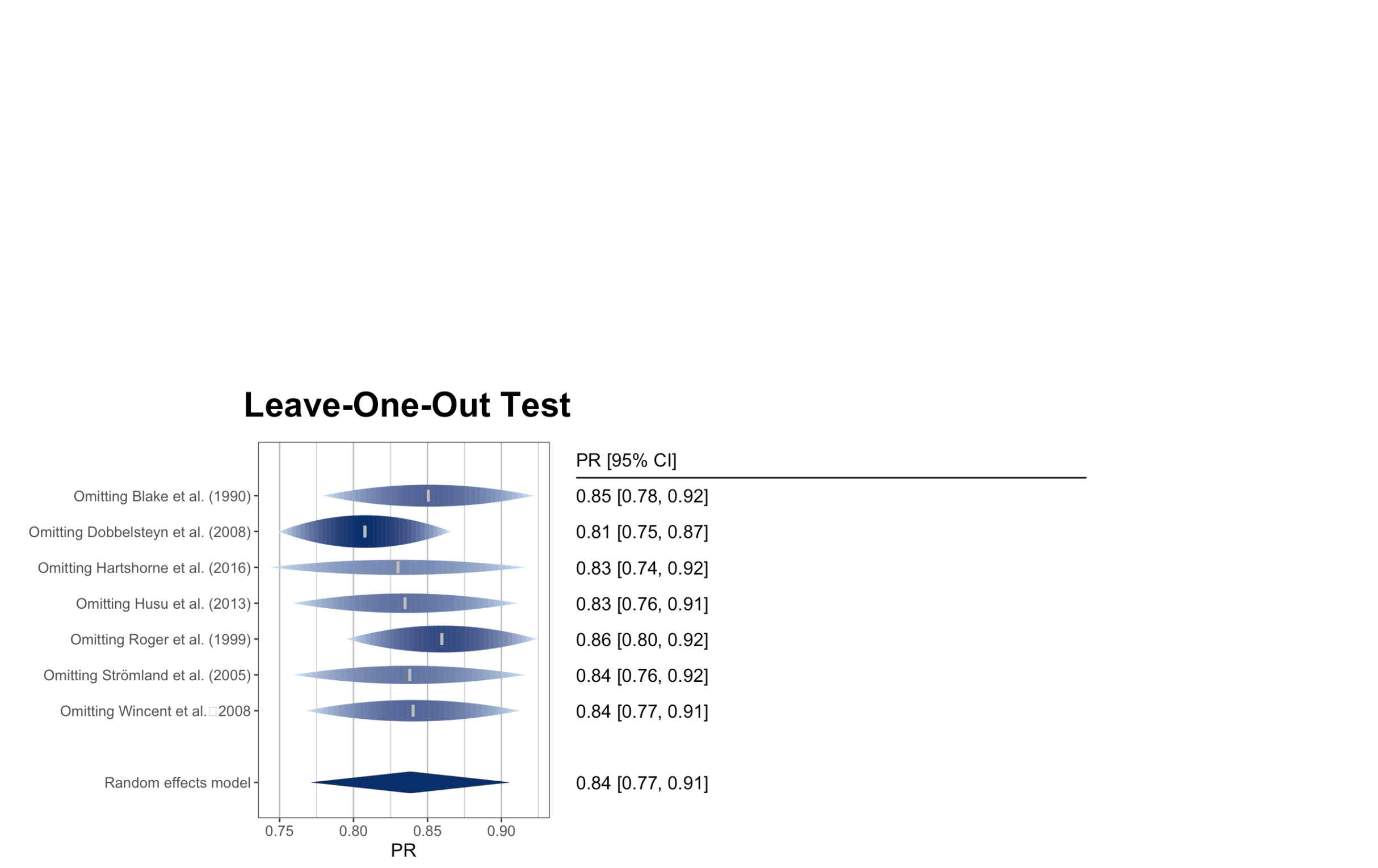


**Note:** Leave one out analysis indicating that no single study is exerting a disproportional influence on the pooled prevalence estimate

###### **Figure A10.5** Subgroup Analysis of Studies Reporting Developmental Delay that were Rated Adequate and Studies Rated Good for Sample Identification


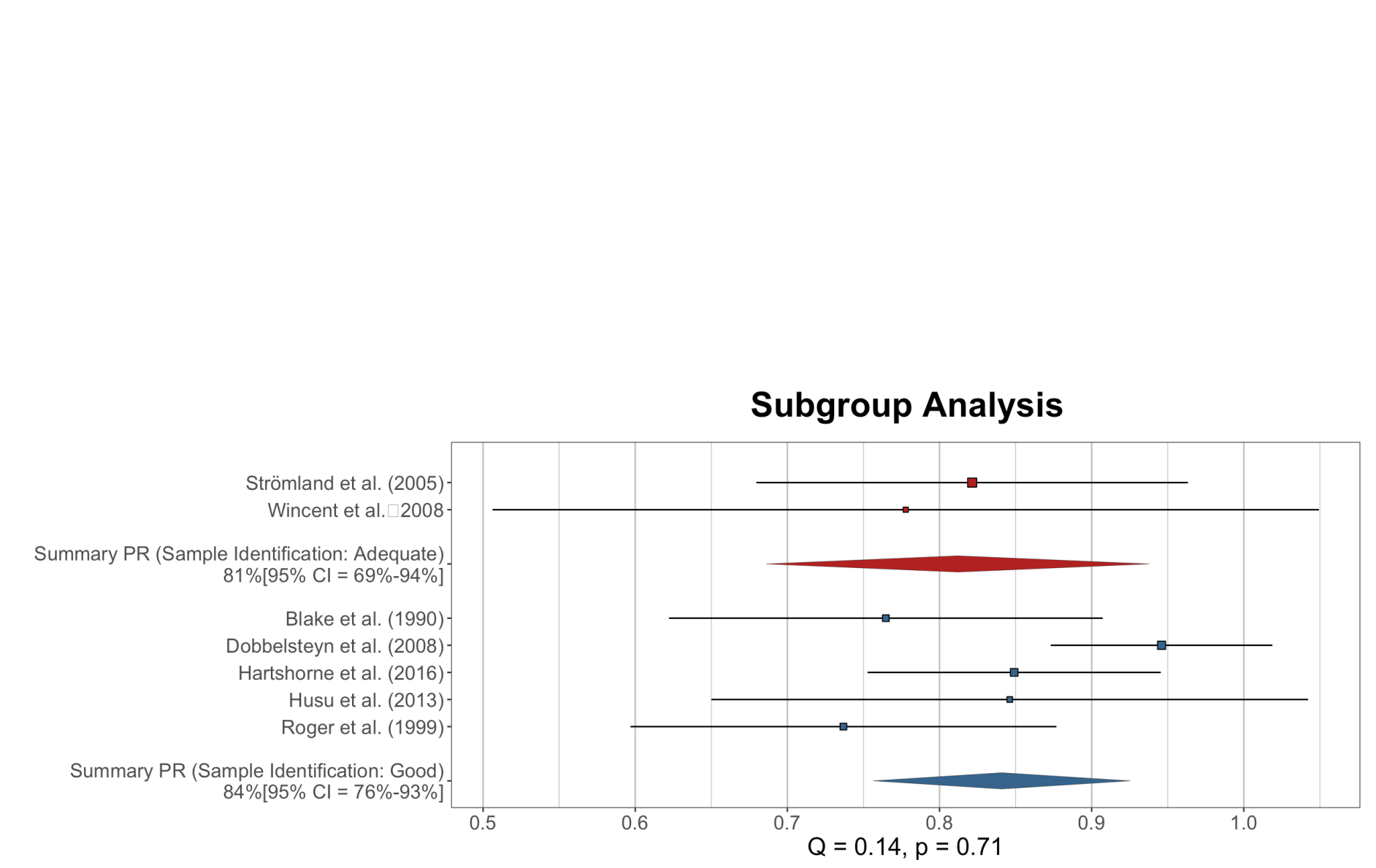


**Note:** Subgroup analysis found no statistical difference (p = 0.71) between studies rated adequate or good for method of sample identification. Subgroup analysis was evaluated by comparison of 95% CIs.

###### **Figure A10.6** Subgroup Analysis of Studies Reporting Developmental Delay that were Rated Adequate and Studies Rated Good for Sample Identification


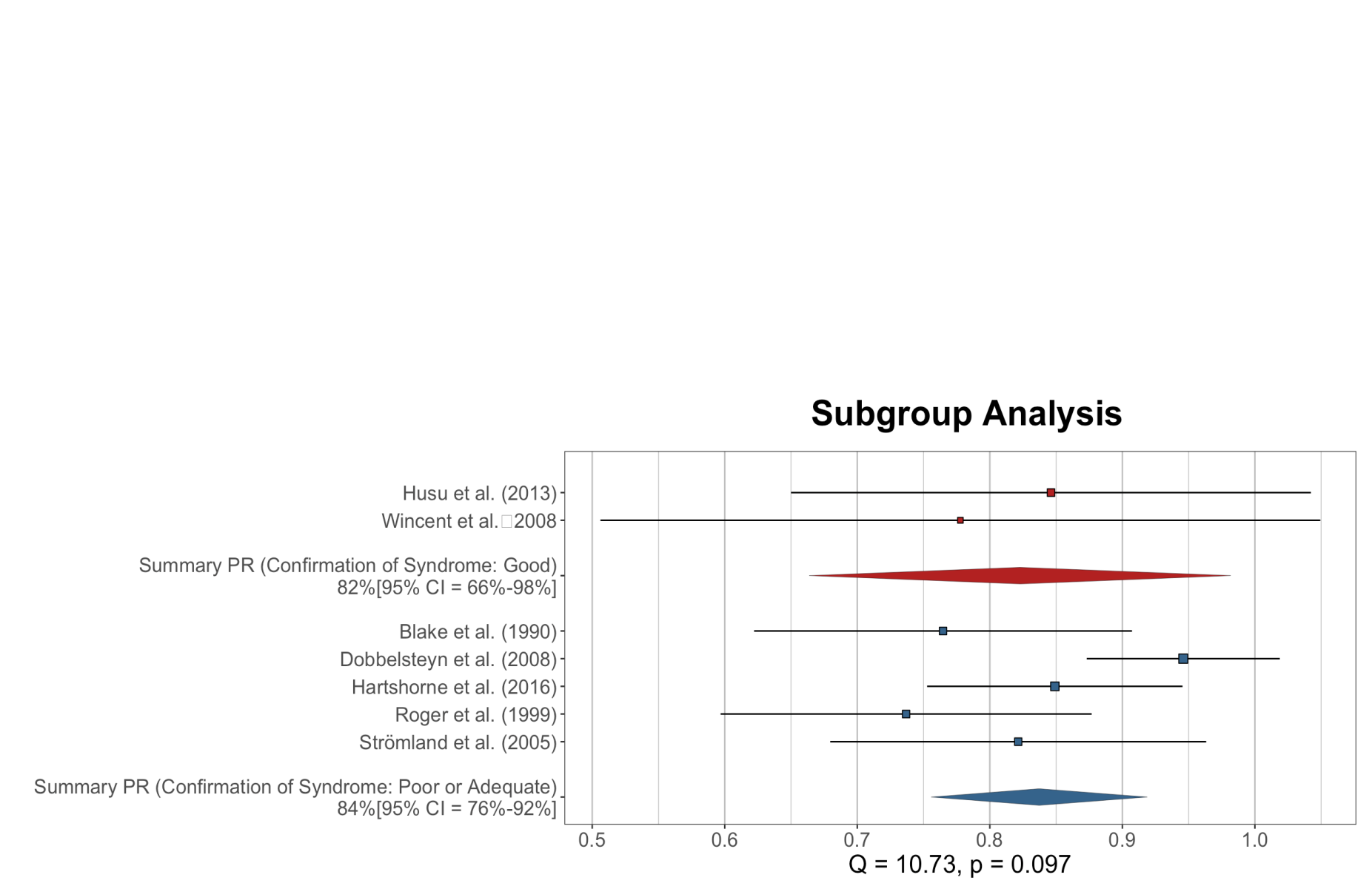


**Note:** Subgroup analysis found no statistical difference (p = 0.097) between studies rated adequate or good for confirmation of syndrome. Subgroup analysis was evaluated by comparison of 95% CIs.

## **Intellectual disability**

A diagnosis of intellectual disability requires deficits in both cognitive functioning and adaptive behaviour (DSM-5 [13]). It was not clear that any of the studies assessed both of these constructs across all participants. Asher et al. [14], Bernstein & Denno [15], Jongmans et al. [16] and Shoji et al. [17] gave no details of assessment, Legendre et al. [18] used estimates of ID for some participants, in Blake and Brown [19], Deuce [20], Oley et al. [12], and Raqbi et al. [21] ID was generally based on type of school provision, with a proportion of participants in Oley et al. [12] completing developmental tests. Hittner et al. [22] used unspecified formal testing, Dammeyer [23] and Davenport et al. [24] reported results from IQ tests only, and Harvey et al. [10], Johansson et al. [11], Lasserre et al. [25] and Sohn et al. [26] assessed cognitive and adaptive functioning but not consistently across the sample. Abadie et al. [27], Salem-Hartshorne & Jacob [28], Smith et al. [6] and Wulffaert et al. [29] used adaptive functioning measures that were completed by parents or carers. Two additional studies [2, 30] reported on skills of daily living in adolescents and adults with CHARGE syndrome. As these skills are only one component of adaptive functioning, they were not included in the meta-analysis. Both studies reported little to no independence in personal care for 19-59% of participants, and little to no independence for 55-93% of individuals in higher-order tasks of daily living including independent travel, cooking, cleaning, shopping and managing personal finances. A 100% prevalence of ID was reported by Asher et al. [14], Davenport et al. [24], Hittner et al. [22], Shoji et al. [17] and Smith et al. [6].

###### **Figure A10.7** QQ Plot of The Distribution of Study Effects and Theoretical Quantities Based on A Normal Distribution Under the Random Effects Model for Studies Reporting Intellectual Disability

**Note:** Visual inspection of the *QQ* plot suggests an approximate normal distribution of study effects for the 16 studies reporting on intellectual disability in CHARGE Syndrome. On this basis the DerSimonian-Laird estimate was used to calculate between studies variance in the random-effects model.

###### **Figure A10.8** Random Effects Models of The Pooled Prevalence Estimate for Studies Reporting Intellectual Disability in CHARGE Syndrome

**Note:** The quality weighted pooled prevalence estimate for intellectual disability in CHARGE syndrome was 64% (95% CI, 54-75%; permuted *p-*value = 0.001; *k* = 16) with high heterogeneity (I^2^ = 88%). Random-effects model calculated using the inverse variance method and the DerSimonian-Laird estimator for τ^2^. Rosenthal Fail-safe N = 6809 suggests that the observed effect is robust to potential publication biases

| **Figure A10.9** Funnel Plot of Standard Error by Prevalence of Intellectual Disability | **Figure A10.10** Baujat Plot of Contribution to Heterogeneity by Influence on Overall Effect for Studies Reporting Intellectual Disability |
| --- | --- |
|  |  |
| **Note:** Visual inspection of the funnel plot conforms to normal expectations and there is weak evidence of substantial publication bias (Egger’s test p = 0.429) | **Note:** Dammeyer (2012) had the greatest contribution to overall heterogeneity and the greatest influence on the overall effect |

###### **Figure A10.11** Leave-One-Out Random Effects Model for Studies Reporting Intellectual Disability


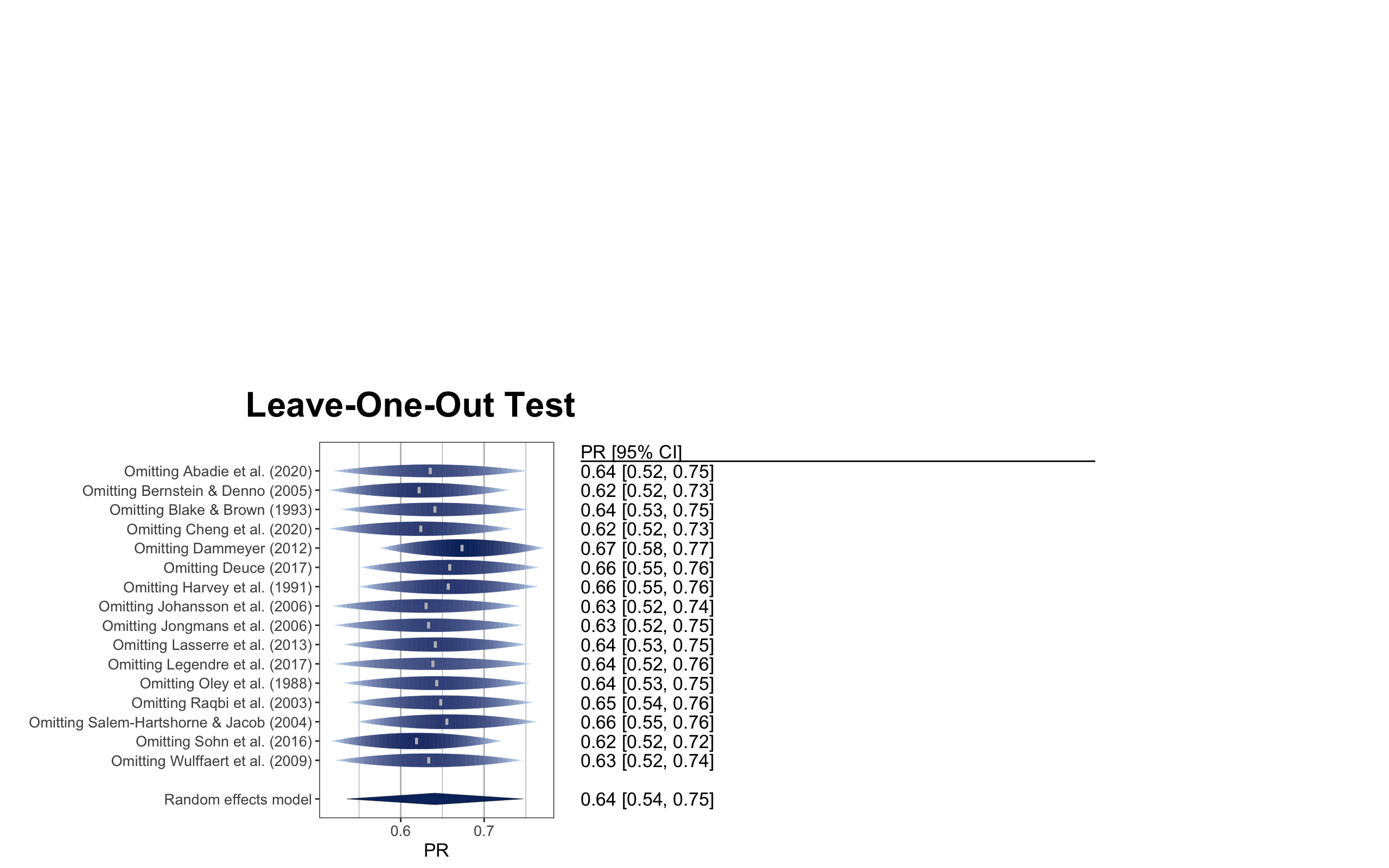


**Note:** Leave one out analysis indicating that no single study is exerting a disproportional influence on the pooled prevalence estimate.

###### **Figure A10.12** Subgroup Analysis of Studies Reporting Intellectual Disability that were Rated Adequate and Studies Rated Good/Excellent for Sample Identification


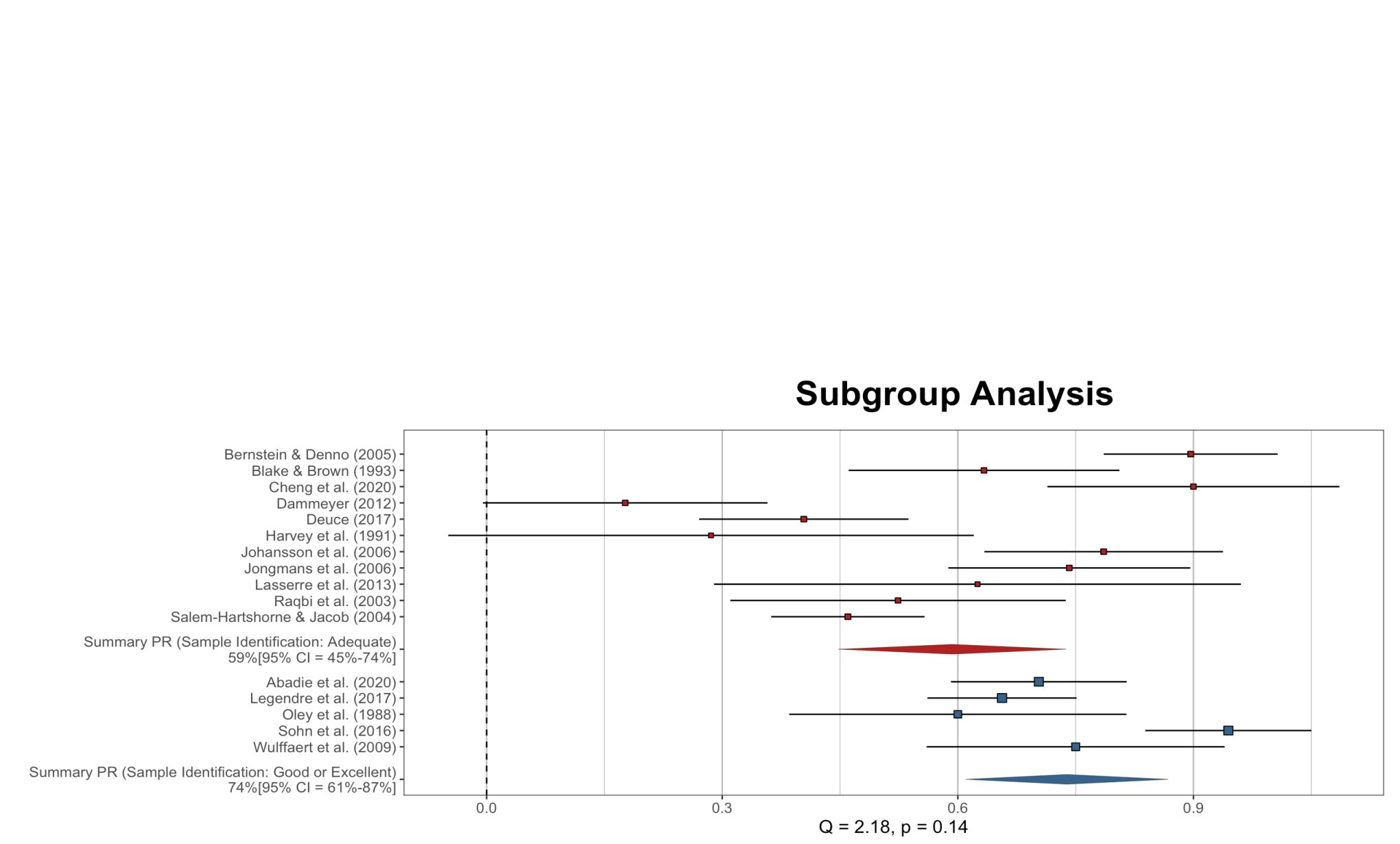


**Note:** Subgroup analysis found no statistical difference (p = 0.14) between studies rated adequate or good/excellent for method of sample identification. Subgroup analysis was evaluated by comparison of 95% CIs.

###### **Figure A10.13** Subgroup Analysis of Studies Reporting Intellectual Disability that were Rated Poor/Adequate and Studies Rated Good for Method of Assessment


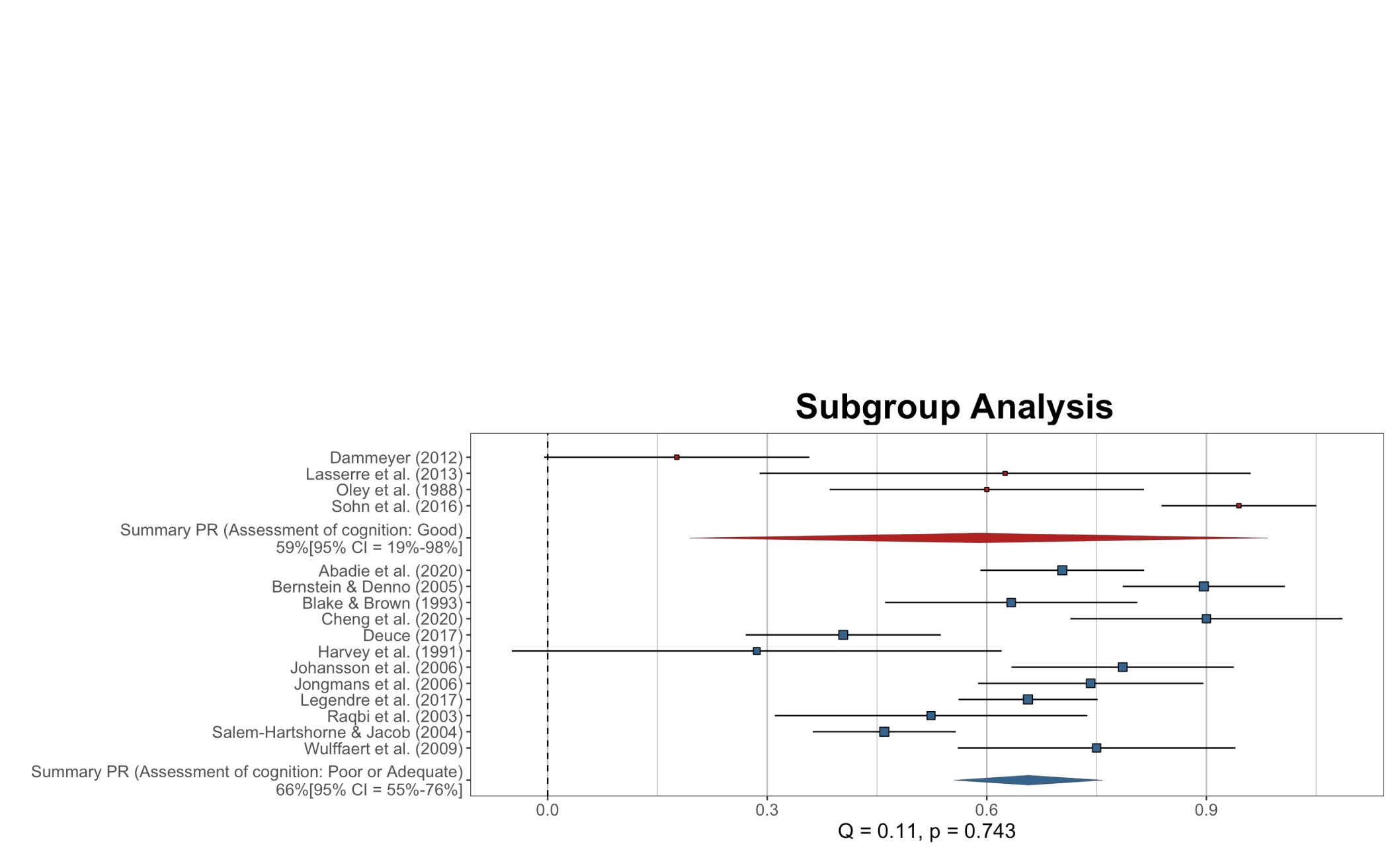


**Note:** Subgroup analysis found no statistical difference (p = 0.743) between studies rated poor/adequate or good for the method used to assess cognition. Subgroup analysis was evaluated by comparison of 95% CIs.

###### **Figure A10.14** QQ Plot of The Distribution of Study Effects and Theoretical Quantities Based on A Normal Distribution Under the Random Effects Model for Studies Reporting Mild or Moderate Intellectual Disability

**Note:** Visual inspection of the *QQ* plot suggests an approximate normal distribution of study effects for the 10 studies reporting on mild or moderate intellectual disability in CHARGE Syndrome. On this basis the DerSimonian-Laird estimate was used to calculate between studies variance in the random-effects model.

###### **Figure A10.15** Random Effects Models of The Pooled Prevalence Estimate for Studies Reporting Mild or Moderate Intellectual Disability in CHARGE Syndrome


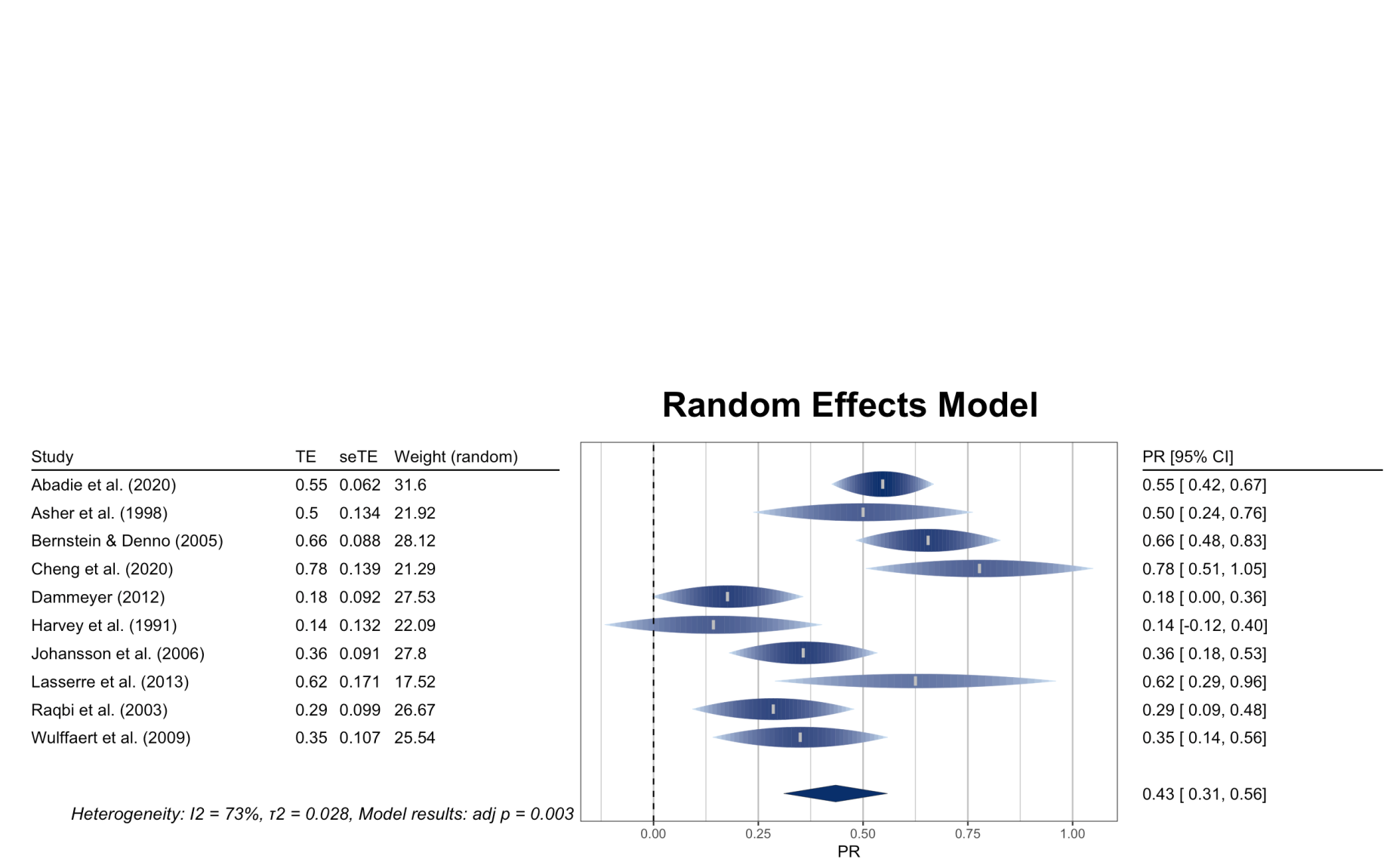


**Note:** The quality weighted pooled prevalence estimate for mild or moderate intellectual disability in CHARGE syndrome was 43% (95% CI, 31-56%; permuted *p-*value = 0.003; *k* = 10) with moderate heterogeneity (I^2^ = 73%). Random-effects model calculated using the inverse variance method and the DerSimonian-Laird estimator for τ^2^. Rosenthal Fail-safe N = 653 suggests that the observed effect is robust to potential publication biases

| Figure A10.16 Funnel Plot of Standard Error by Prevalence of Mild or Moderate Intellectual Disability | Figure A10.17 Baujat Plot of Contribution to Heterogeneity by Influence on Overall Effect for Studies Reporting Mild or Moderate Intellectual Disability |
| --- | --- |
|  |  |
| **Note:** Visual inspection of the funnel plot conforms to normal expectations and there is weak evidence of substantial publication bias (Egger’s test p = 0.839) | **Note:** Studies in the top right quartile have the greatest contribution to overall heterogeneity and the greatest influence on the overall effect. Dammeyer (2012) had the greatest contribution to overall heterogeneity and Cheng et al. (2020) had the greatest influence on the overall effect |

###### **Figure A10.18** Leave-One-Out Random Effects Model for Studies Reporting Mild or Moderate Intellectual Disability


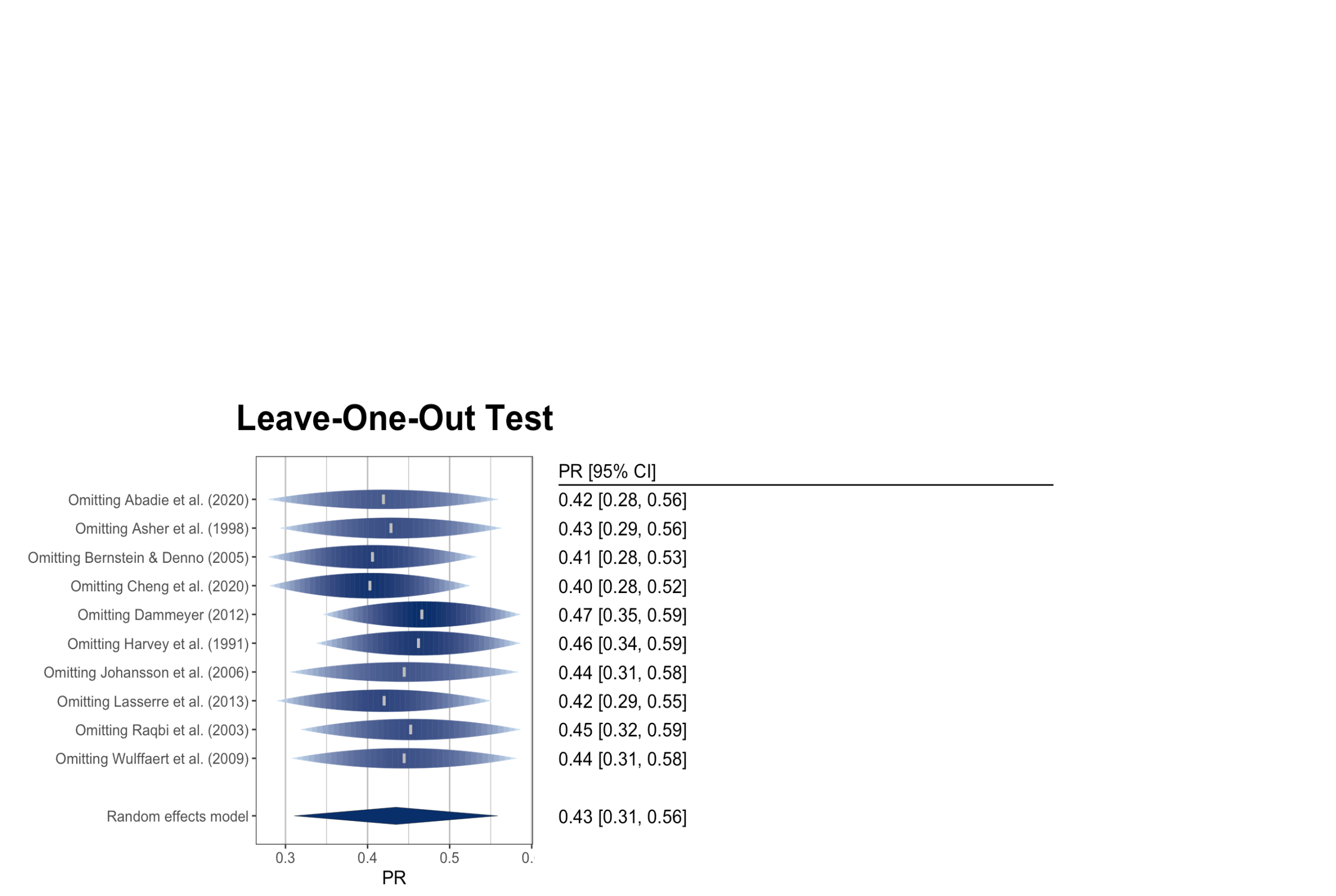


**Note:** Leave one out analysis indicating that no single study is exerting a disproportional influence on the pooled prevalence estimate

###### **Figure A10.19** Subgroup Analysis of Studies Reporting Mild or Moderate Intellectual Disability that were Rated Adequate and Studies Rated Good/Excellent for Sample Identification


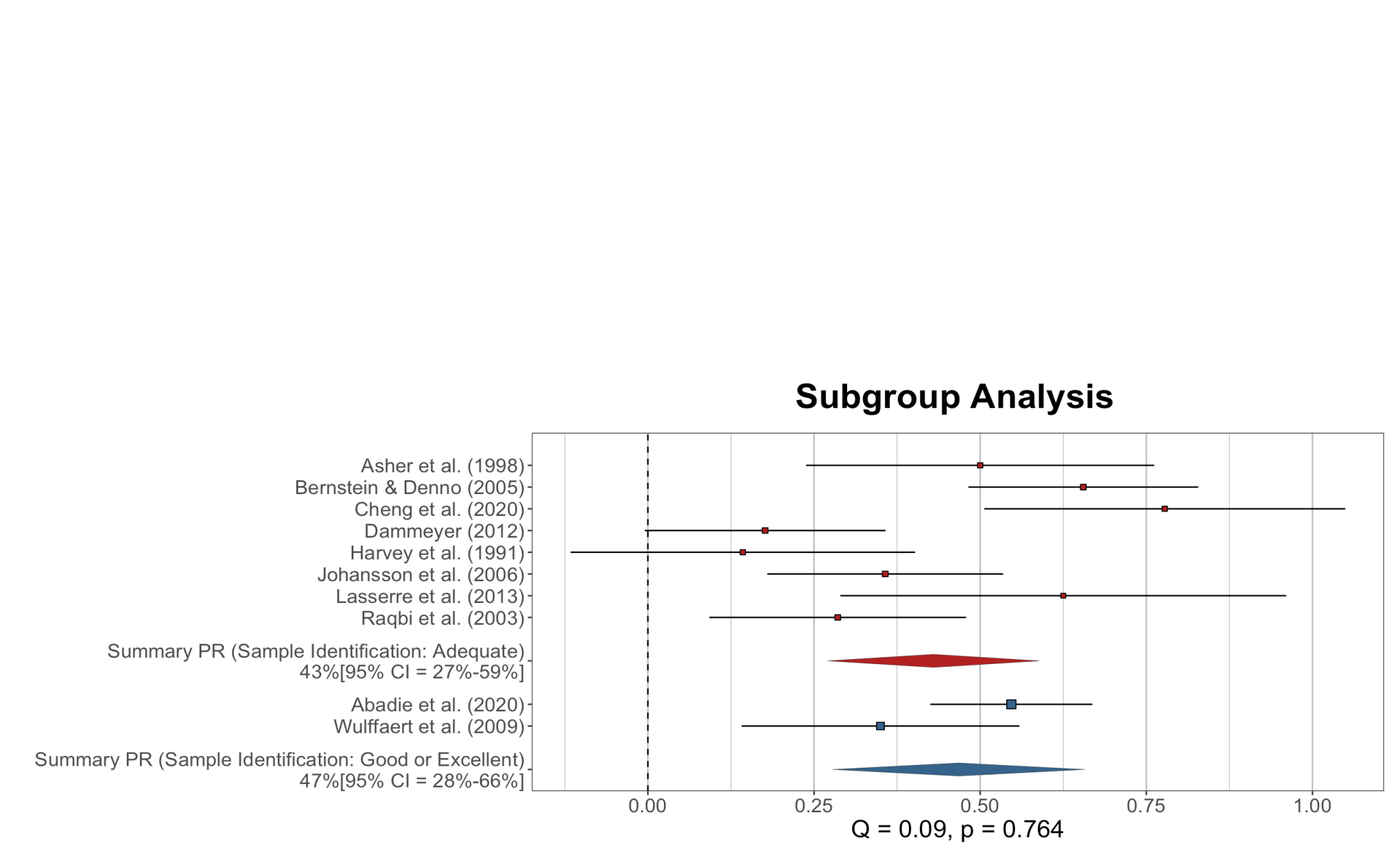


**Note:** Subgroup analysis found no statistical difference (p = 0.764) between studies rated adequate or good/excellent for method of sample identification. Subgroup analysis was evaluated by comparison of 95% CIs.

###### **Figure A10.20** Subgroup Analysis of Studies Reporting Mild or Moderate Intellectual Disability that were Rated Poor/Adequate and Studies Rated Good for Confirmation of Syndrome


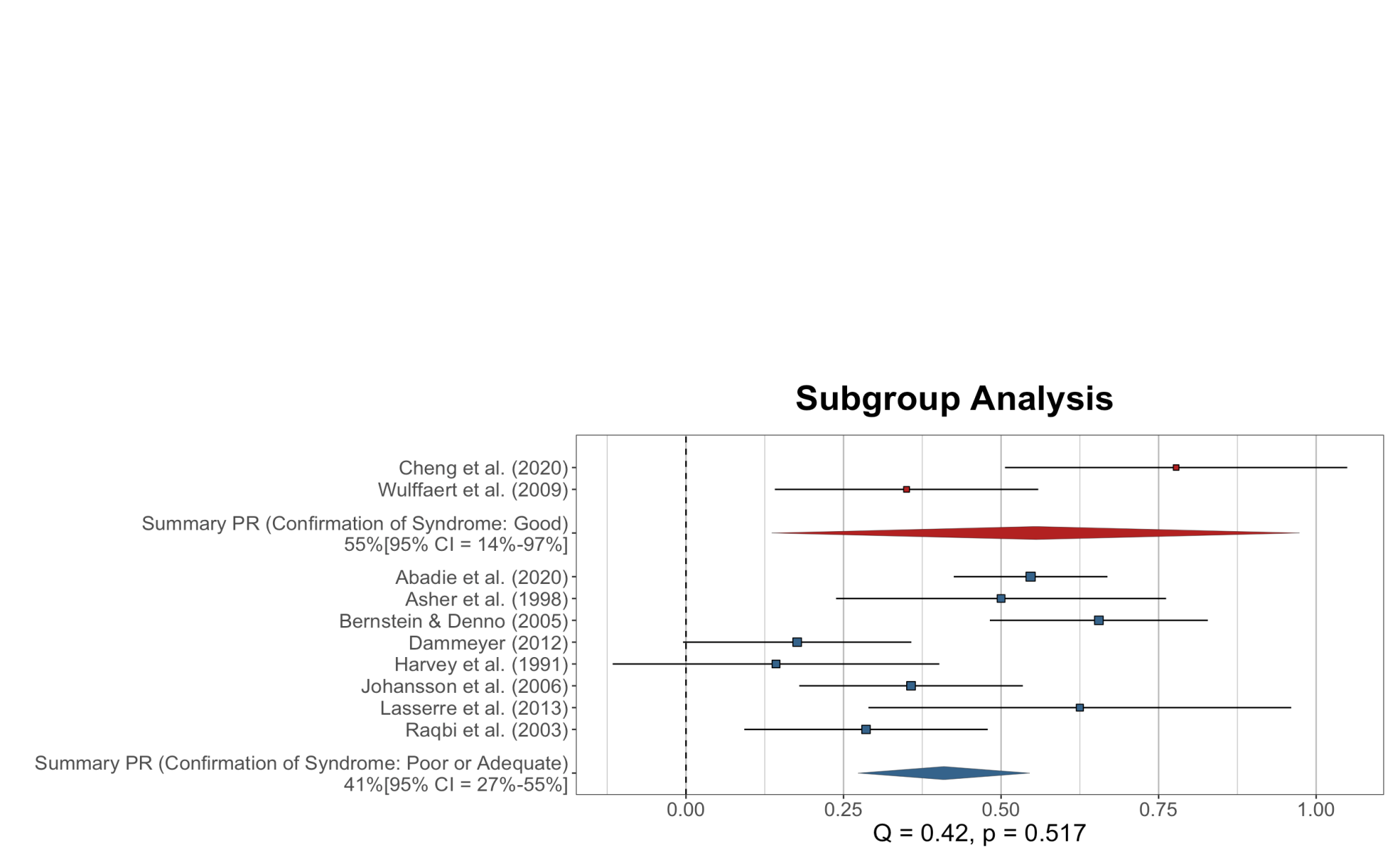


**Note:** Subgroup analysis found no statistical difference (p = 0.764) between studies rated poor/adequate or good quality for confirmation of syndrome. Subgroup analysis was evaluated by comparison of 95% CIs.

###### **Figure A10.21** Subgroup Analysis of Studies Reporting Mild or Moderate Intellectual Disability that were Rated Poor/Adequate and Studies Rated Good for Method of Assessment


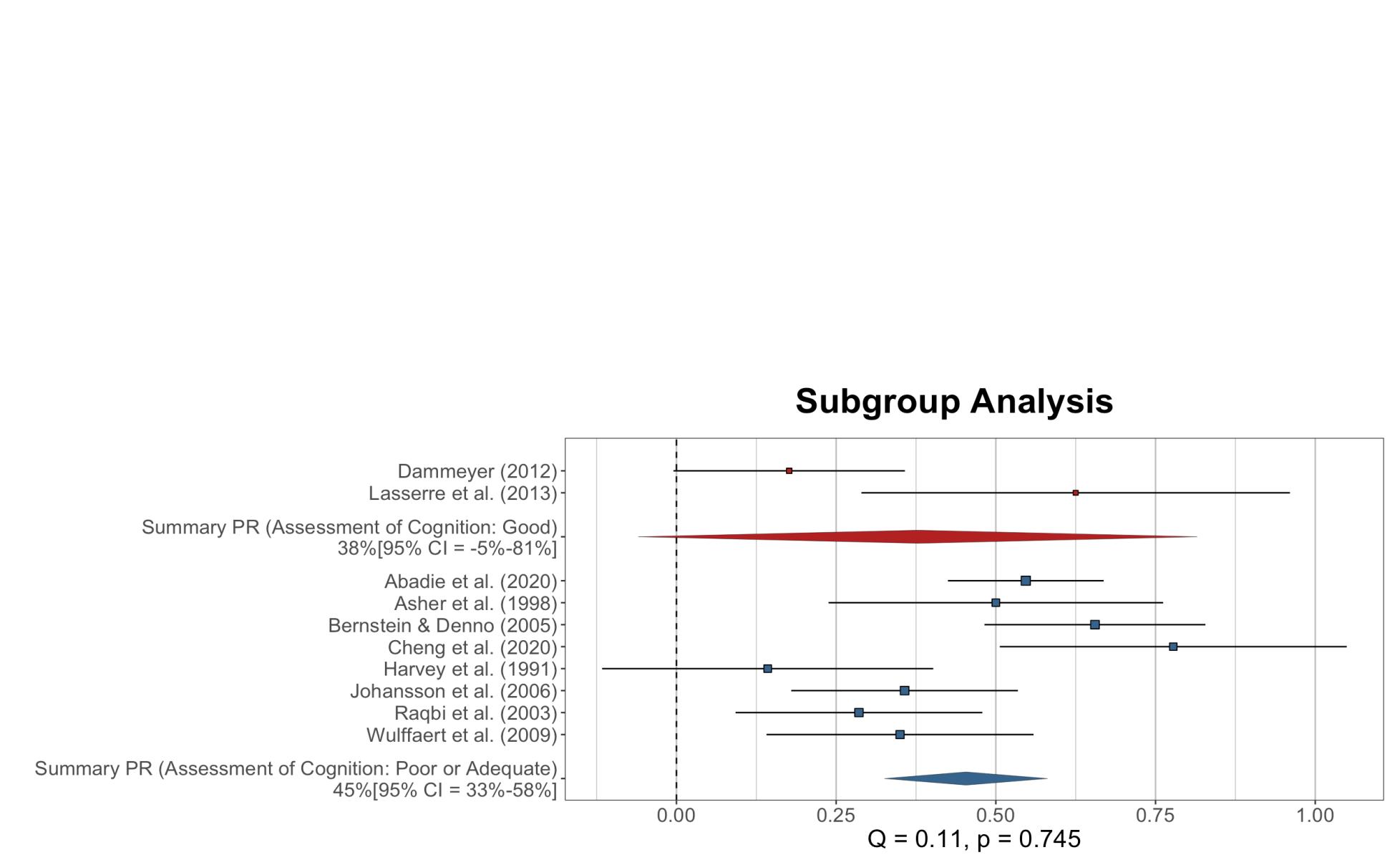


Subgroup analysis found no statistical difference (p = 0.745) between studies rated poor/adequate or good for the method used to assess cognition. Subgroup analysis was evaluated by comparison of 95% CIs.

###### **Figure A10.22** QQ Plot of The Distribution of Study Effects and Theoretical Quantities Based on A Normal Distribution Under the Random Effects Model for Studies Reporting Severe or Profound Intellectual Disability

**Note:** Visual inspection of the *QQ* plot suggests an approximate normal distribution of study effects for the 11 studies reporting on severe or profound intellectual disability in CHARGE Syndrome. On this basis the DerSimonian-Laird estimate was used to calculate between studies variance in the random-effects model.

###### **Figure A10.23** Random Effects Models of The Pooled Prevalence Estimate for Studies Reporting Severe or Profound Intellectual Disability in CHARGE Syndrome

**Note:** The quality weighted pooled prevalence estimate for severe or profound intellectual disability in CHARGE syndrome was 28% (95% CI, 19-37%; permuted *p-*value = 0.001; *k* = 11) with moderate heterogeneity (I^2^ = 61%). Random-effects model calculated using the inverse variance method and the DerSimonian-Laird estimator for τ^2^. Rosenthal Fail-safe N = 394 suggests that the observed effect is robust to potential publication biases

| **Figure A10.24** Funnel Plot of Standard Error by Prevalence of Severe or Profound Intellectual Disability Following the Trim and Fill Procedure | **Figure A10.25** Baujat Plot of Contribution to Heterogeneity by Influence on Overall Effect for Studies Reporting Severe or Profound Intellectual Disability |
| --- | --- |
|  |  |
| **Note:** Publication bias [small study effect] was identified (Egger’s test p = 0.029). The trim and fill procedure did not impute any missing studies. | **Note:** Hittner et al. (1979) had the greatest contribution to overall heterogeneity and the greatest influence on the overall effect. |

###### **Figure A10.26** Leave-One-Out Random Effects Model for Studies Reporting Severe or Profound Intellectual Disability


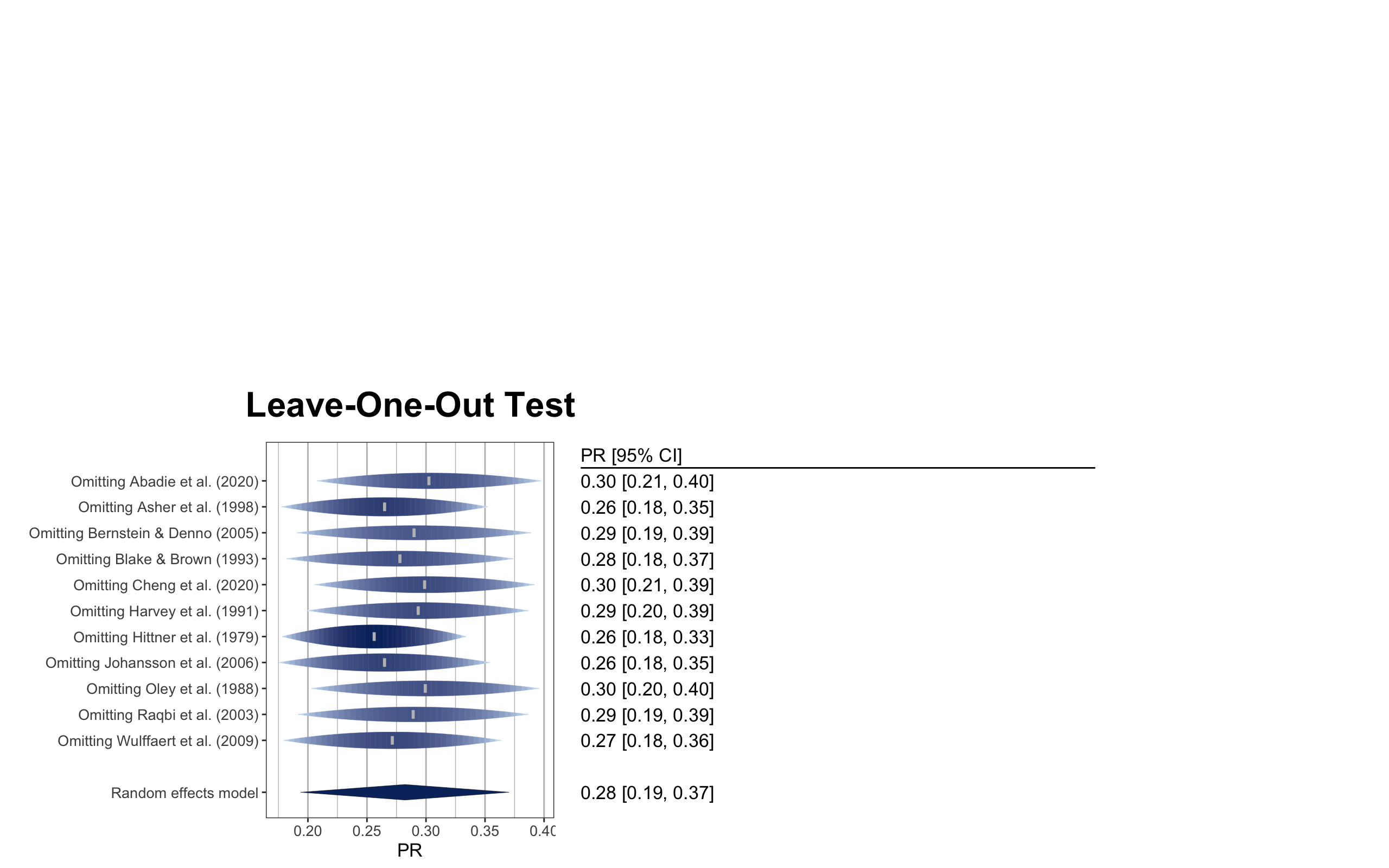


**Note:** Leave one out analysis indicating that no single study is exerting a disproportional influence on the pooled prevalence estimate

###### **Figure A10.27** Subgroup Analysis of Studies Reporting Severe or Profound Intellectual Disability that were Rated Adequate and Studies Rated Good/Excellent for Sample Identification


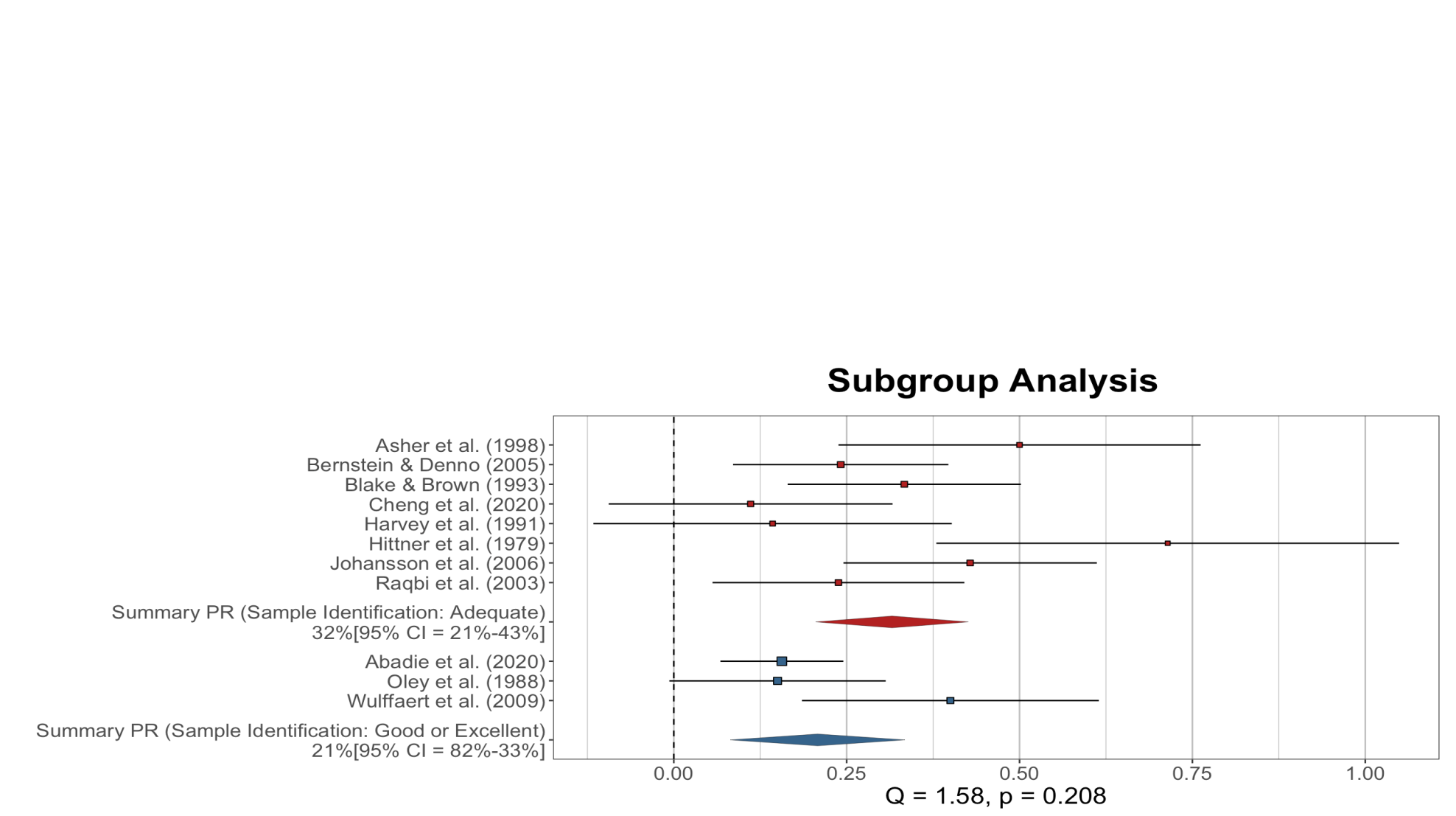


**Note:** Subgroup analysis found no statistical difference (p = 0.208) between studies rated adequate or good/excellent for method of sample identification. Subgroup analysis was evaluated by comparison of 95% CIs.

###### **Figure A10.28** Subgroup Analysis of Studies Reporting Severe or Profound Intellectual Disability that were Rated Poor/Adequate and Studies Rated Good for Confirmation of Syndrome


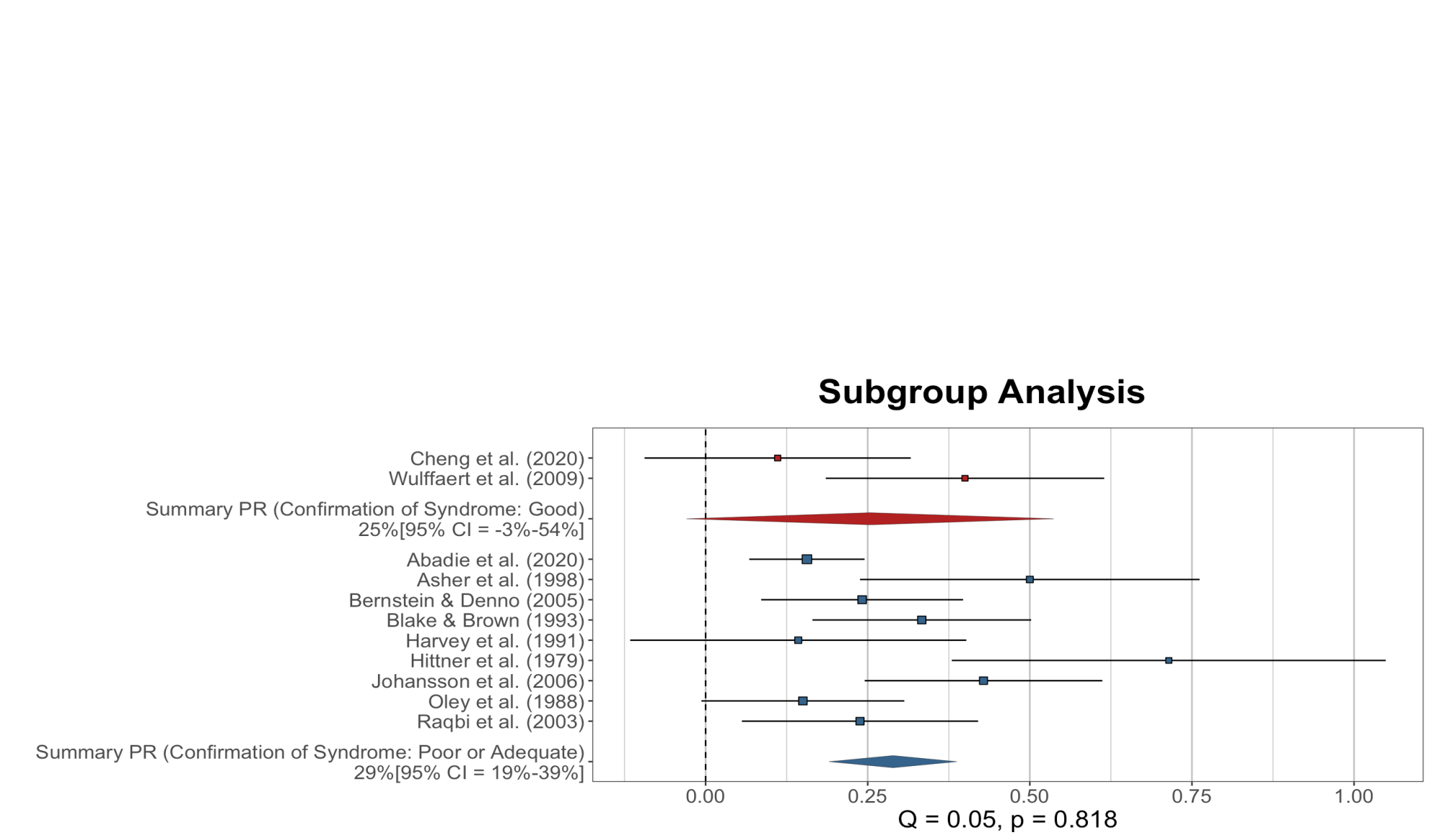


**Note:** Subgroup analysis found no statistical difference (p = 0.818) between studies rated poor/adequate or good quality for confirmation of syndrome. Subgroup analysis was evaluated by comparison of 95% CIs.

## **Autism diagnosis**

A number of studies also reported autistic behaviours that could not be included in the meta-analysis because they lacked continuity. Deuce et al. [31], Hartshorne & Cypher [32] and Harvey et al. [10] reported a 5%, 6% and 33% prevalence of ‘autistic traits’ respectively. Blake et al. [2] suggested a 53% prevalence of socialising problems, Deuce et al. [31] reported 48% of their sample had greater ease and more success in forming relationships with adults than with peers, and in Souriau et al. [33], 42% of individuals with CHARGE syndrome had difficulty with social rules. Further, Hartshorne & Cypher [32] accrued 100 survey responses regarding 25 behaviours indicative of autism spectrum disorder presented by individuals with CHARGE syndrome and reported a prevalence rate from 16% to 66% for these behaviours.

###### **Figure A10.29** QQ Plot of The Distribution of Study Effects and Theoretical Quantities Based on A Normal Distribution Under the Random Effects Model for Studies Reporting Autism

**Note:** Visual inspection of the *QQ* plot suggests an approximate normal distribution of study effects for the 8 studies reporting on Autism diagnosis in CHARGE Syndrome. On this basis the DerSimonian-Laird estimate was used to calculate between studies variance in the random-effects model.

###### **Figure A10.30** Random Effects Models of The Pooled Prevalence Estimate for Studies Reporting Autism in CHARGE Syndrome

**Note:** The quality weighted pooled prevalence estimate for an Autism diagnosis in CHARGE syndrome was 28% (95% CI, 16-41%; permuted *p-*value = 0.007; *k* = 8) with high heterogeneity (I^2^ = 90%). Random-effects model calculated using the inverse variance method and the DerSimonian-Laird estimator for τ^2^

###### **Figure A10.31** Baujat Plot of Contribution to Heterogeneity by Influence on Overall Effect for Studies Reporting Autism

**Note:** Abadie at al. (2020) had the greatest contribution to overall heterogeneity and the greatest influence on the overall effect

###### **Figure A10.32** Leave-One-Out Random Effects Model for Studies Reporting Autism


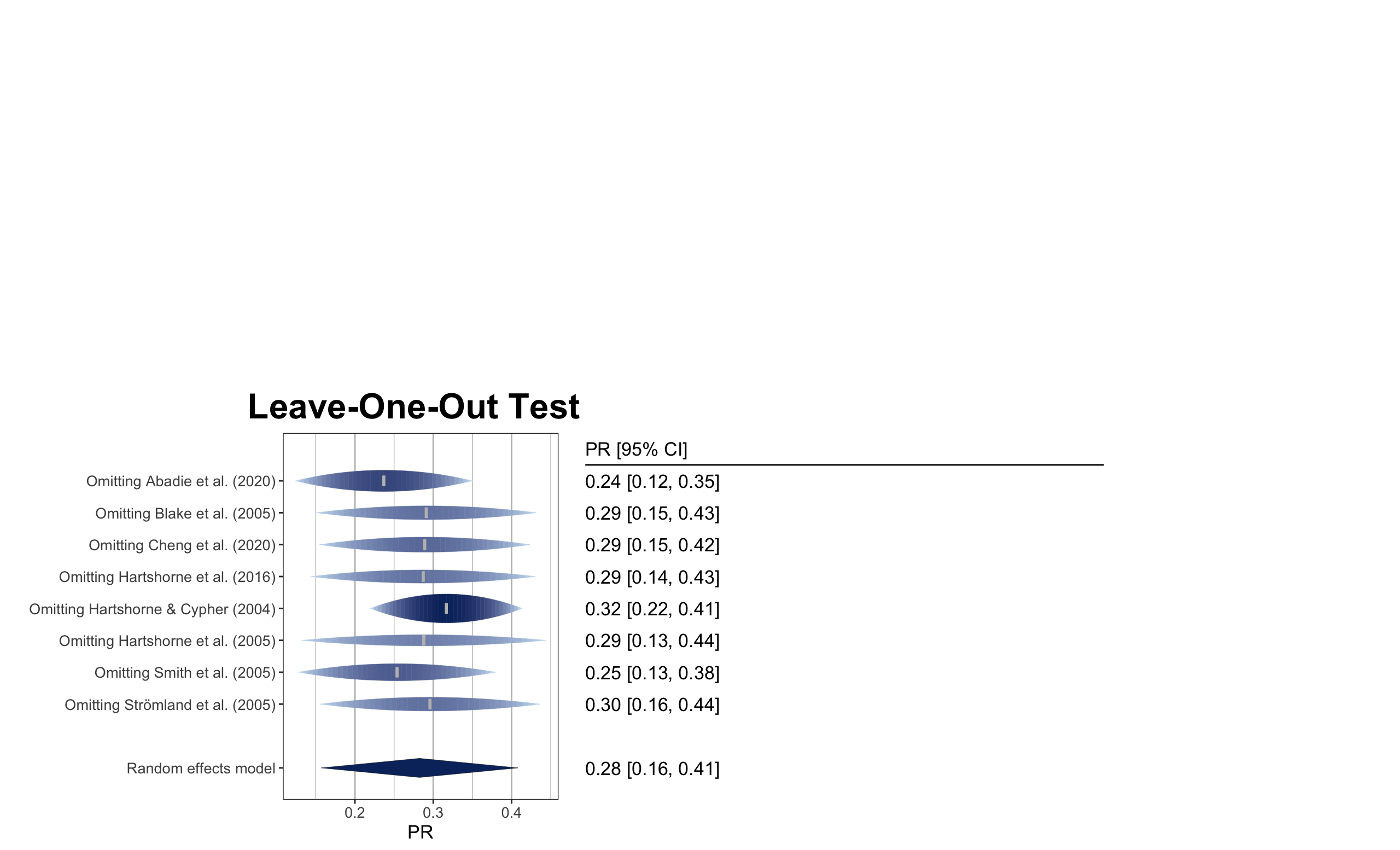


**Note:** Leave one out analysis indicating that no single study is exerting a disproportional influence on the pooled prevalence estimate

###### **Figure A10.33** Subgroup Analysis of Studies Reporting Autism that were Rated Adequate and Studies Rated Good/Excellent for Sample Identification


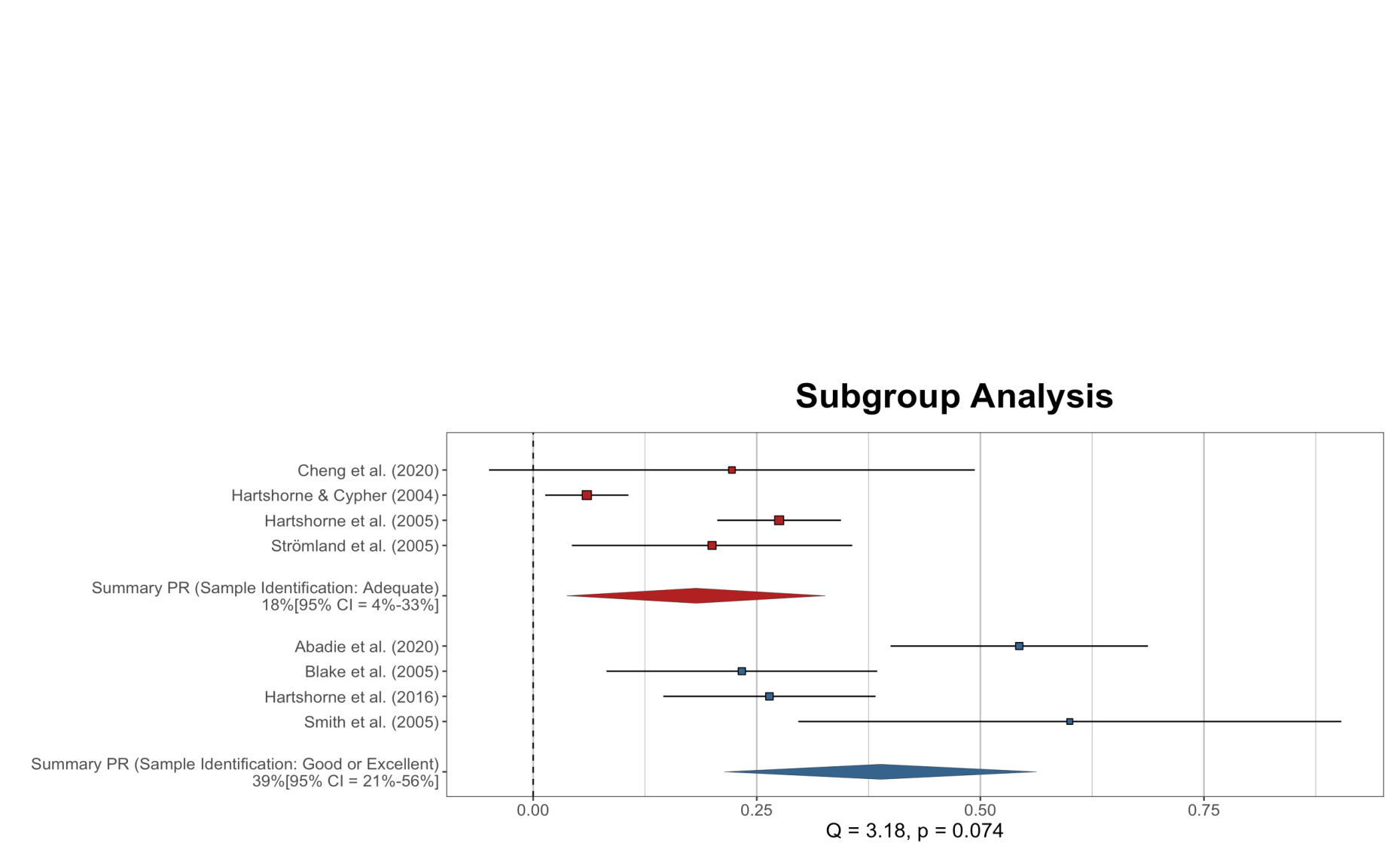


**Note:** Subgroup analysis found no statistical difference (p = 0.074) between studies rated adequate or good/excellent for method of sample identification. Subgroup analysis was evaluated by comparison of 95% CIs.

## **Aggressive Behaviours**

To maintain consistency, estimates excluded from the analysis included Blake et al. [2], who reported a 13% prevalence of conduct disorder in a sample of 30 adolescents and adults with CHARGE syndrome and Hartshorne et al. [34] reported disruptive behaviour which commonly includes aggression.

###### **Figure A10.34** QQ Plot of The Distribution of Study Effects and Theoretical Quantities Based on A Normal Distribution Under the Random Effects Model for Studies Reporting Aggressive Behaviour

**Note:** Visual inspection of the *QQ* plot suggests an approximate normal distribution of study effects for the 6 studies reporting on aggressive behaviour in CHARGE Syndrome. On this basis the DerSimonian-Laird estimate was used to calculate between studies variance in the random-effects model

###### **Figure A10.35** Random Effects Models of The Pooled Prevalence Estimate for Studies Reporting Aggressive Behaviour in CHARGE Syndrome

**Note:** The quality weighted pooled prevalence estimate for aggressive behaviour in CHARGE syndrome was 48% (95% CI, 40-57%; permuted *p-*value = 0.031; *k* = 6) with moderate heterogeneity (I^2^ = 52%). Random-effects model calculated using the inverse variance method and the DerSimonian-Laird estimator for τ^2^

###### **Figure A10.36** Baujat Plot of Contribution to Heterogeneity by Influence on Overall Effect for Studies Reporting Aggressive Behaviour

**Note:** Johansson et al. (2006) had the greatest contribution to overall heterogeneity and the greatest influence on the overall effect

###### **Figure A10.37** Leave-One-Out Random Effects Model for Studies Reporting Aggressive Behaviour


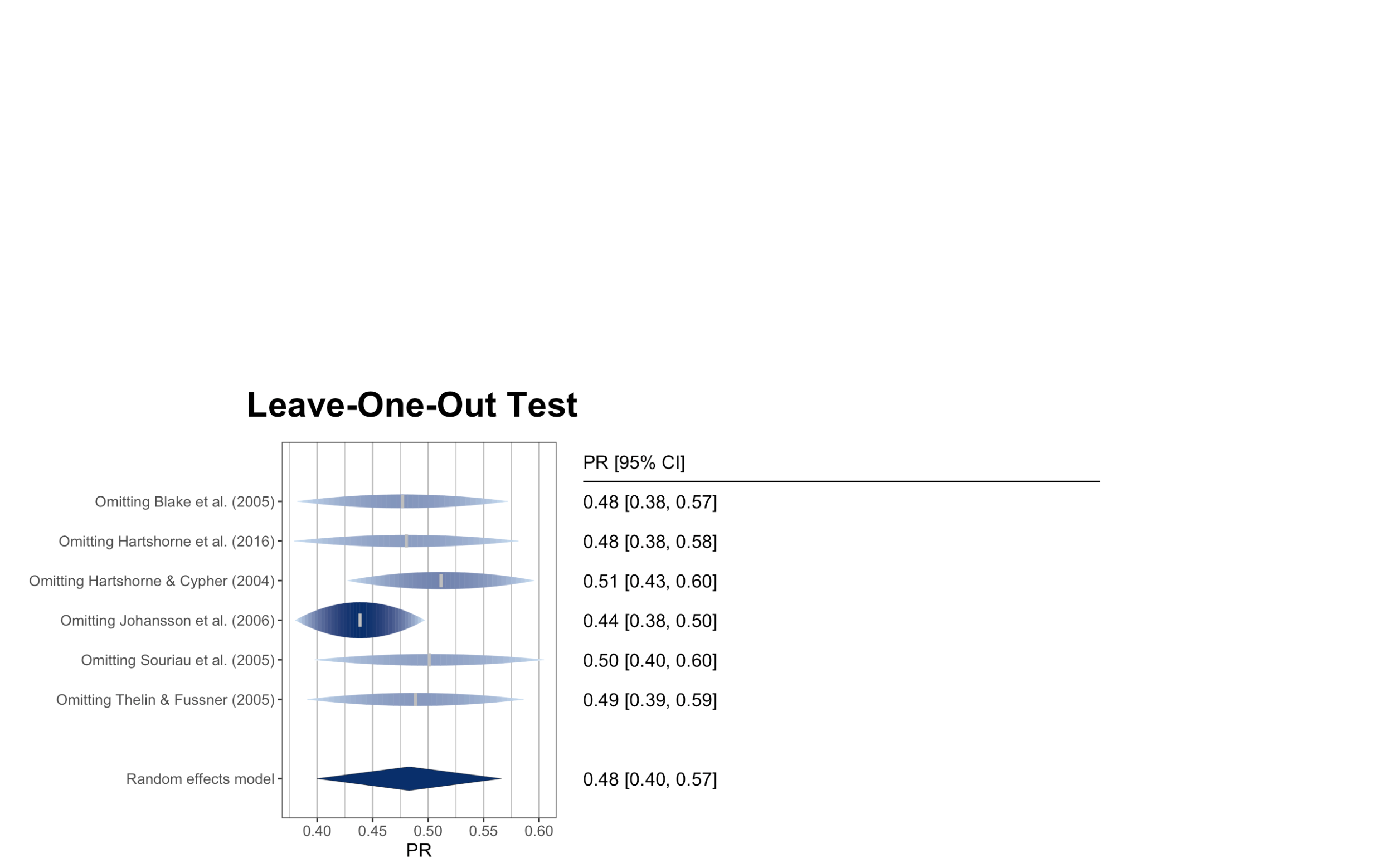


**Note:** Leave one out analysis indicating that no single study is exerting a disproportional influence on the pooled prevalence estimate

###### **Figure A10.38** Subgroup Analysis of Studies Reporting Aggressive Behaviour that were Rated Adequate and Studies Rated Good for Sample Identification


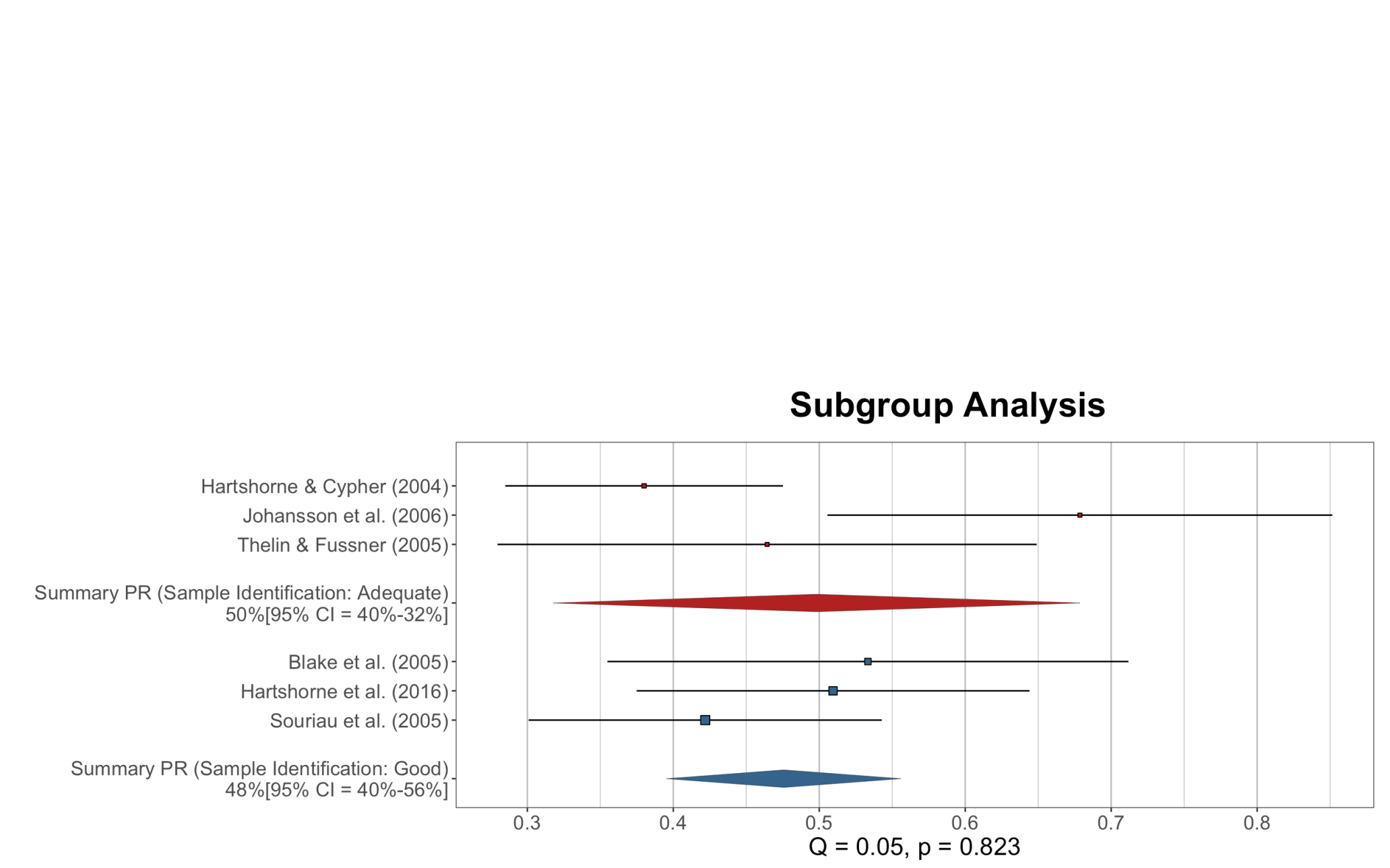


Note: Subgroup analysis found no statistical difference (p = 0.823) between studies rated adequate or good for method of sample identification. Subgroup analysis was evaluated by comparison of 95% CIs.

## **Self-Injurious Behaviour**

Given the small number of studies included in this meta-analysis and the use of a fixed effects model, caution should be taken in generalising results beyond the five included studies.

###### **Figure A10.39** QQ Plot of The Distribution of Study Effects and Theoretical Quantities Based on A Normal Distribution Under the Random Effects Model for Studies Reporting Self-Injurious Behaviour

**Note:** Visual inspection of the *QQ* plot suggests a non-Gaussian distribution of study effects for the 5 studies reporting on self-injurious behaviour in CHARGE Syndrome. On this basis the restricted maximum likelihood estimator was used to calculate between studies variance in the random-effects model

###### **Figure A10.40** Random Effects Models of The Pooled Prevalence Estimate for Studies Reporting Self-Injurious Behaviour in CHARGE Syndrome

**Note:** The quality weighted random effects model for self-injurious behaviour in CHARGE syndrome suggest a non-significant pooled prevalence estimate of 44% (95% CI, 36-51%; permuted *p-*value = 0.063; *k* = 5) with low heterogeneity (I^2^ = 31%). Random-effects model calculated using the inverse variance method and the restricted maximum likelihood estimator for τ^2^

###### **Figure A10.41** QQ Plot of The Distribution of Study Effects and Theoretical Quantities Based on A Normal Distribution Under the Fixed Effects Model for Studies Reporting Self-Injurious Behaviour

**Note:** Revised QQ plot based on a fixed effects model: QQ plot of study effects for the 5 studies reporting on self-injurious behaviour in CHARGE Syndrome

###### **Figure A10.42** Fixed Effects Models of The Pooled Prevalence Estimate for Studies Reporting Self-Injurious Behaviour in CHARGE Syndrome

**Note:** The pooled prevalence estimate for self-injurious behaviour in CHARGE syndrome was 44% (95% CI, 36-51%; p = <0.001; *k* = 5). Fixed-effects model calculated using the inverse variance method.

## **Obsessive or Compulsive Behaviour**

###### **Figure A10.43** QQ Plot of The Distribution of Study Effects and Theoretical Quantities Based on A Normal Distribution Under the Random Effects Model for Studies Reporting Obsessive or Compulsive Behaviour

**Note:** Visual inspection of the *QQ* plot suggests a non-Gaussian distribution of study effects for the 6 studies reporting obsessive or compulsive behaviour in CHARGE Syndrome. On this basis the restricted maximum likelihood estimator was used to calculate between studies variance in the random-effects model.

###### **Figure A10.44** Random Effects Models of The Pooled Prevalence Estimate for Studies Reporting Obsessive or Compulsive Behaviour in CHARGE Syndrome

**Note:** The quality weighted pooled prevalence estimate for obsessive or compulsive behaviour in CHARGE syndrome was 36% (95% CI, 14-57%; permuted *p-*value = 0.031; *k* = 6) with high heterogeneity (I^2^ = 96%). Random-effects model calculated using the inverse variance method and the restricted maximum likelihood estimator for τ^2^

###### **Figure A10.45** Baujat Plot of Contribution to Heterogeneity by Influence on Overall Effect for Studies Reporting Obsessive or Compulsive Behaviour

**Note:** Bernstein and Denno (2005) had the greatest contribution to overall heterogeneity and the greatest influence on the overall effect

###### **Figure A10.46** Leave-One-Out Random Effects Model for Studies Reporting Obsessive or Compulsive Behaviour


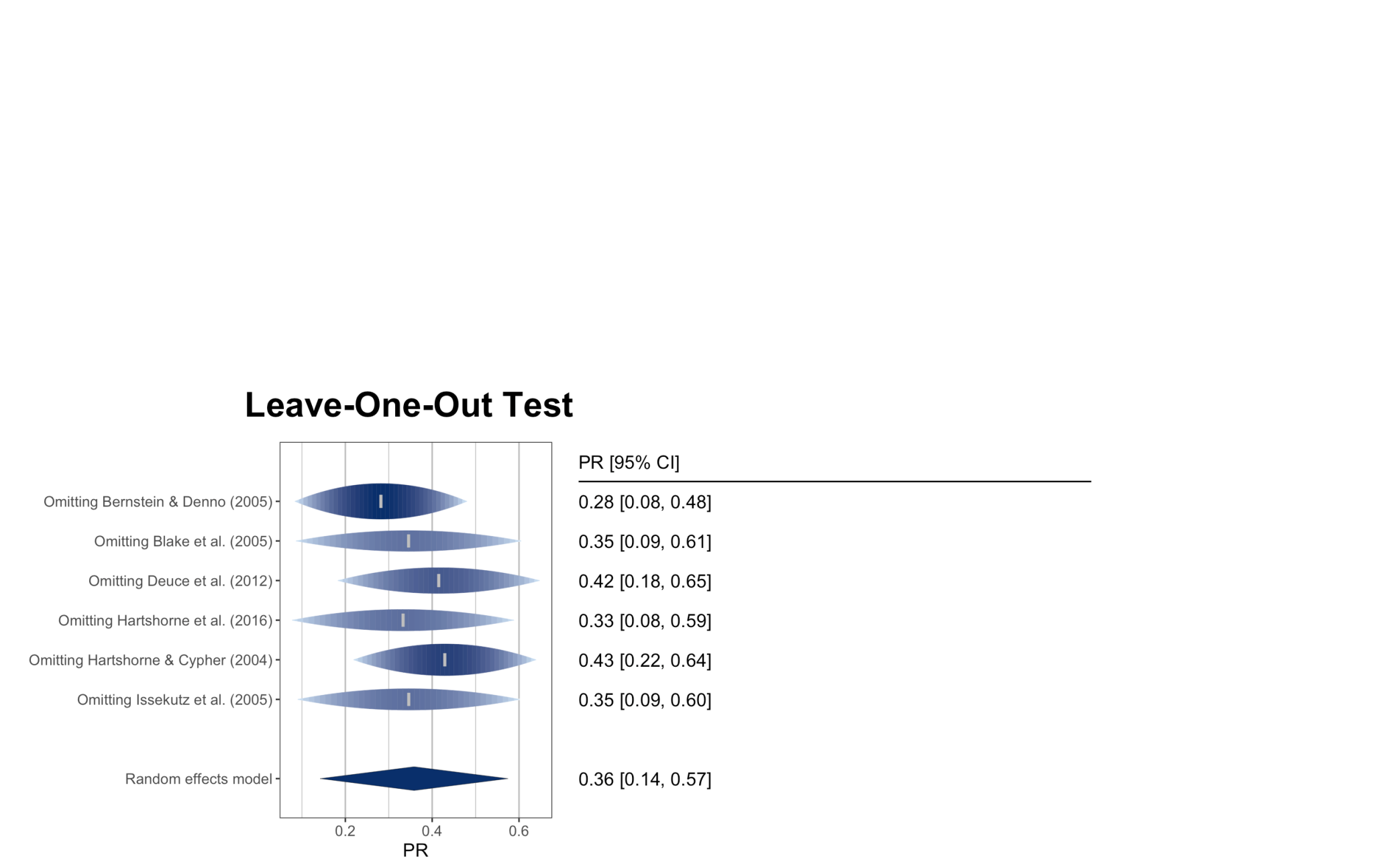


**Note:** Leave one out analysis indicating that no single study is exerting a disproportional influence on the pooled prevalence estimate

###### **Figure A10.47** Subgroup Analysis of Studies Reporting Obsessive or Compulsive Behaviour that were Rated Adequate and Studies Rated Good/Excellent for Sample Identification


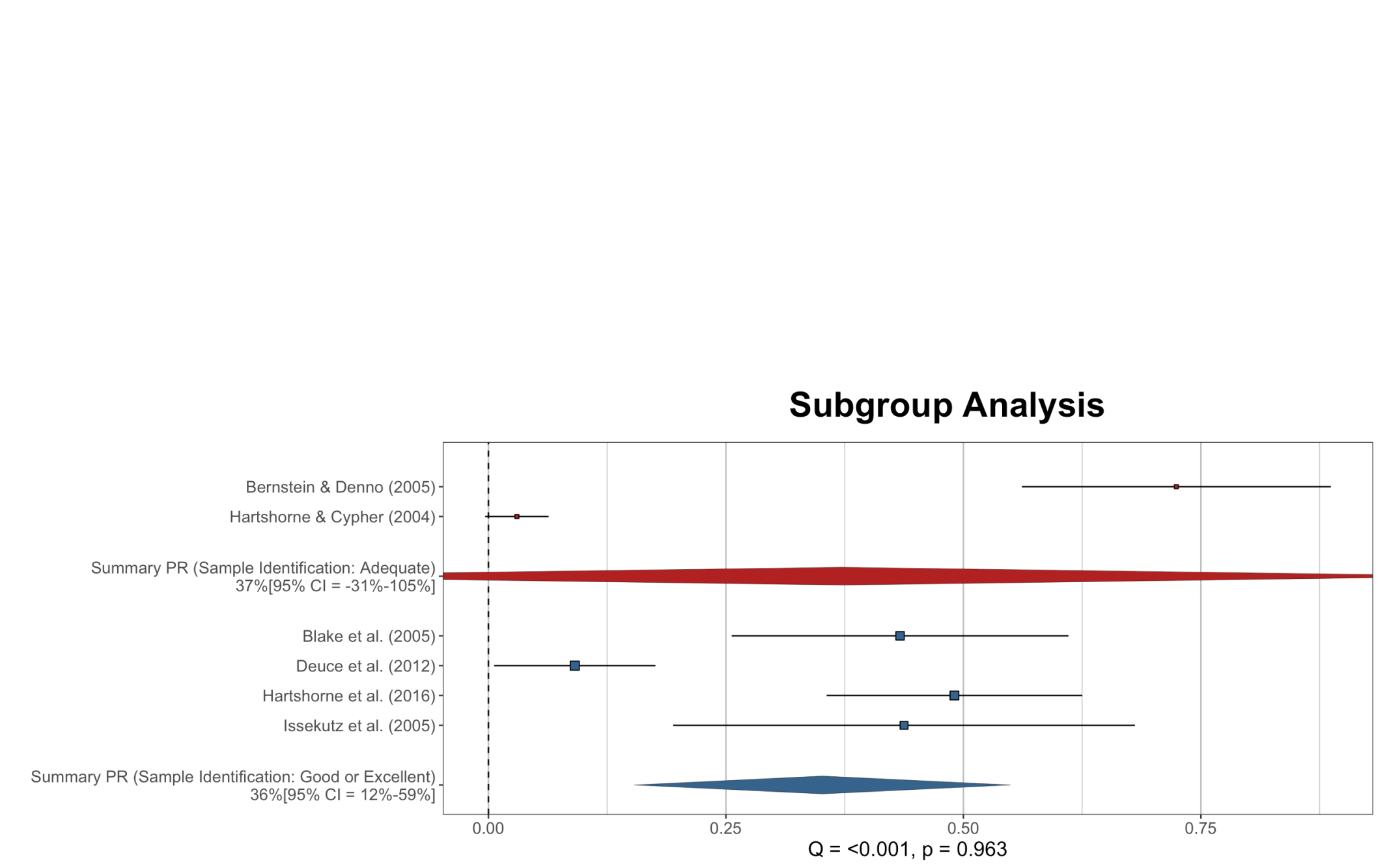


**Note:** Subgroup analysis found no statistical difference (p = 0.963) between studies rated adequate or good/excellent for method of sample identification. Subgroup analysis was evaluated by comparison of 95% CIs.

## **Tactile Defensiveness**

Given the small number of studies included in this meta-analysis it is unclear how representative the generated prevalence estimate is for the CHARGE population. Four additional studies reported different typologies of sensory behaviour including Deuce [20] who reported difficulty with sensory overload or under-stimulation (75%), Hartshorne & Cypher [32] and Johansson et al. [11] who reported unusual interest in auditory, visual or tactile stimuli (14-52%), Hartshorne et al. [30] and Johansson et al. [11] who reported movement stimulation including stereotyped body movements (50-54%), Hartshorne & Cypher [32] reported spinning in circles (27%) and rocking behaviours (33%) and Johansson et al. [11] and Souriau et al. [33] reported an oversensitivity to noises or the feeling of certain substances (18-59%). However, given the idiosyncratic nature of these descriptions they could not be reliably synthesised.

###### **Figure A10.48** QQ Plot of The Distribution of Study Effects and Theoretical Quantities Based on A Normal Distribution Under the Random Effects Model for Studies Reporting Tactile Defensiveness

**Note:** Visual inspection of the *QQ* plot suggests a non-Gaussian distribution of study effects for the 5 studies reporting on tactile defensiveness in CHARGE Syndrome. On this basis the restricted maximum likelihood estimator was used to calculate between studies variance in the random-effects model.

###### **Figure A10.49** Random Effects Models of The Pooled Prevalence Estimate for Studies Reporting Tactile Defensiveness in CHARGE Syndrome

**Note:** The quality weighted random effects model for tactile defensiveness in CHARGE syndrome suggest a non-significant pooled prevalence estimate of 48% (95% CI, 42-55%; permuted *p-*value = 0.063; *k* = 5). Heterogeneity could not be detected. Random-effects model calculated using the inverse variance method and the restricted maximum likelihood estimator for τ^2^

###### **Figure A10.50** QQ Plot of The Distribution of Study Effects and Theoretical Quantities Based on A Normal Distribution Under the Fixed Effects Model for Studies Reporting Tactile Defensiveness

**Note:** Revised QQ plot based on a fixed effects model: QQ plot of study effects for the 5 studies reporting on tactile defensiveness in CHARGE Syndrome

###### **Figure A10.51** Fixed Effects Models of The Pooled Prevalence Estimate for Studies Reporting Tactile Defensiveness in CHARGE Syndrome

**Note:** The pooled prevalence estimate for tactile defensiveness in CHARGE syndrome was 48% (95% CI, 42-55%; p = <0.001; *k* = 5). Fixed-effects model calculated using the inverse variance method.

**Sleep Difficulties**

Prevalence rates for sleep difficulties in CHARGE syndrome were collated from 11 eligible studies. Sleep difficulties consist of any combination of insomnia, hypersomnolence or parasomnia disorders, sleep-related breathing disorders, circadian rhythm sleep-wake disorders or sleep-related movement disorders [35].

###### **Figure A10.52** QQ Plot of The Distribution of Study Effects and Theoretical Quantities Based on A Normal Distribution Under the Random Effects Model for Studies Reporting Sleep Difficulties

**Note:** Visual inspection of the *QQ* plot suggests an approximate normal distribution of study effects for the 11 studies reporting on sleep problems in CHARGE Syndrome. On this basis the DerSimonian-Laird estimate was used to calculate between studies variance in the random-effects model.

###### **Figure A10.53** Random Effects Models of The Pooled Prevalence Estimate for Studies Reporting Sleep Difficulties in CHARGE Syndrome

**Note:** The quality weighted pooled prevalence estimate for sleep problems in CHARGE syndrome was 45% (95% CI, 31-59%; permuted *p-*value = 0.002; *k* = 11) with high heterogeneity (I^2^ = 89%). Random-effects model calculated using the inverse variance method and the DerSimonian-Laird estimator for τ^2^. Rosenthal Fail-safe N = 1481 suggests that the observed effect is robust to potential publication biases

| **Figure A10.54** Funnel Plot of Standard Error by Prevalence of Sleep Difficulties Following the Trim and Fill Procedure | **Figure A10.55** Baujat Plot of Contribution to Heterogeneity by Influence on Overall Effect for Studies Reporting Sleep Difficulties |
| --- | --- |
|  |  |
| **Note:** Visual inspection of the funnel plot conforms to normal expectations and there is weak evidence of substantial publication bias (Egger’s test p = 0.933) | **Note:** Dammeyer (2012) had the greatest contribution to overall heterogeneity and the greatest influence on the overall effect |

###### **Figure A10.56** Leave-One-Out Random Effects Model for Studies Reporting Sleep Difficulties


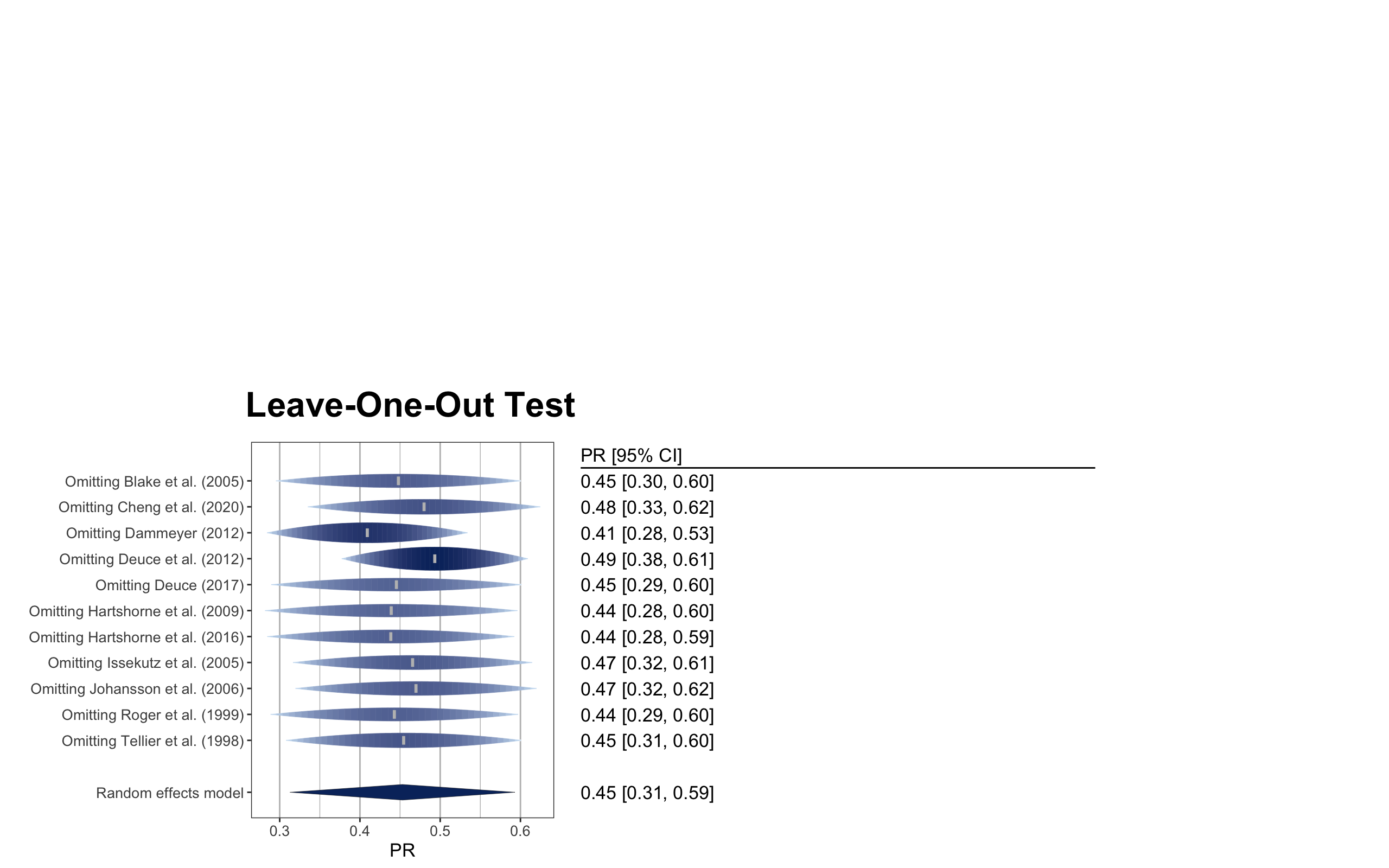


**Note:** Leave one out analysis indicating that no single study is exerting a disproportional influence on the pooled prevalence estimate

###### **Figure A10.57** Subgroup Analysis of Studies Reporting Sleep Difficulties that were Rated Adequate and Studies Rated Good/Excellent for Sample Identification


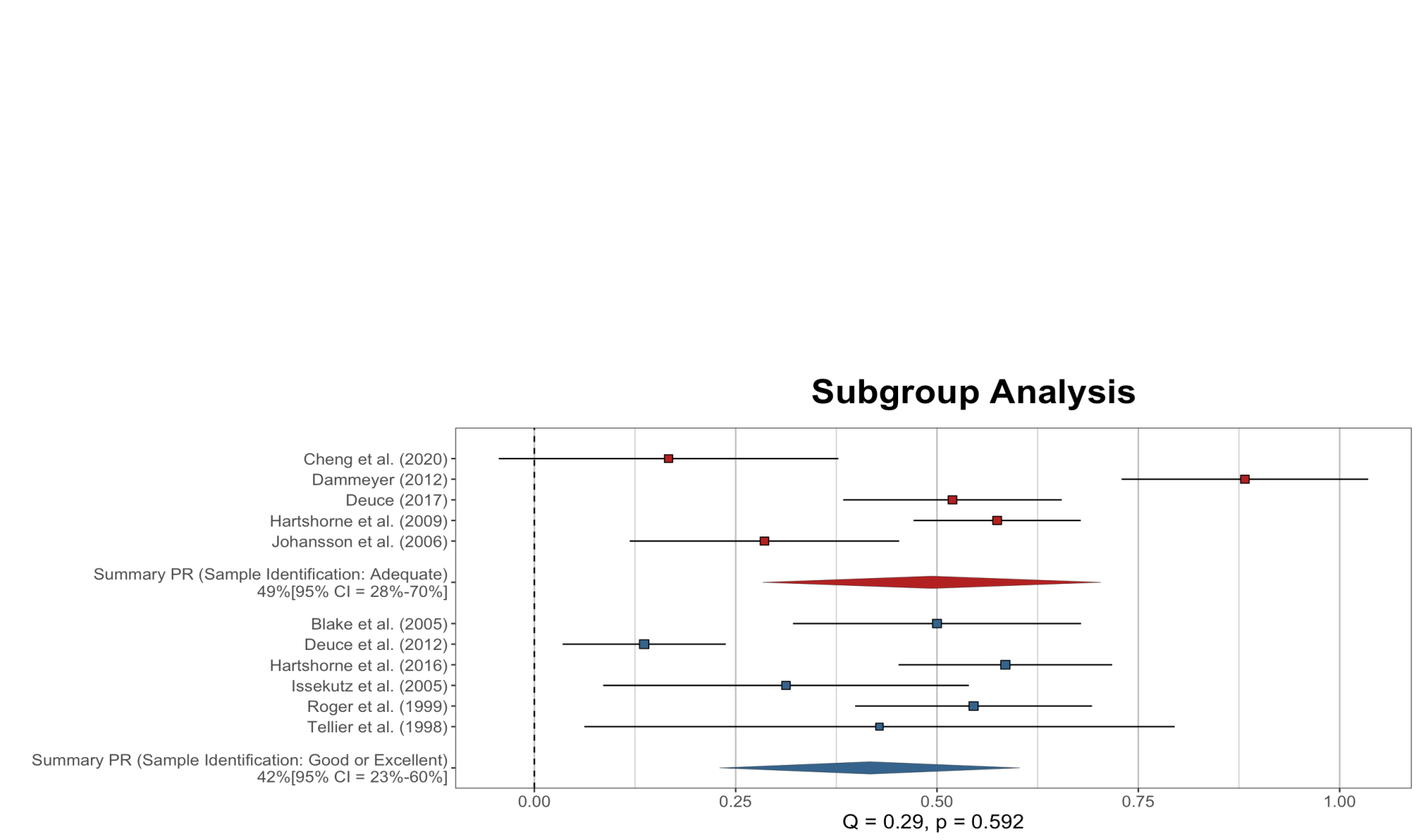


**Note:** Subgroup analysis found no statistical difference (p = 0.592) between studies rated adequate or good/excellent for method of sample identification. Subgroup analysis was evaluated by comparison of 95% CIs.

###### **Figure A10.58** Subgroup Analysis of Studies Reporting Sleep Difficulties that were Rated Poor/Adequate and Studies Rated Excellent for Method of Sleep Assessment


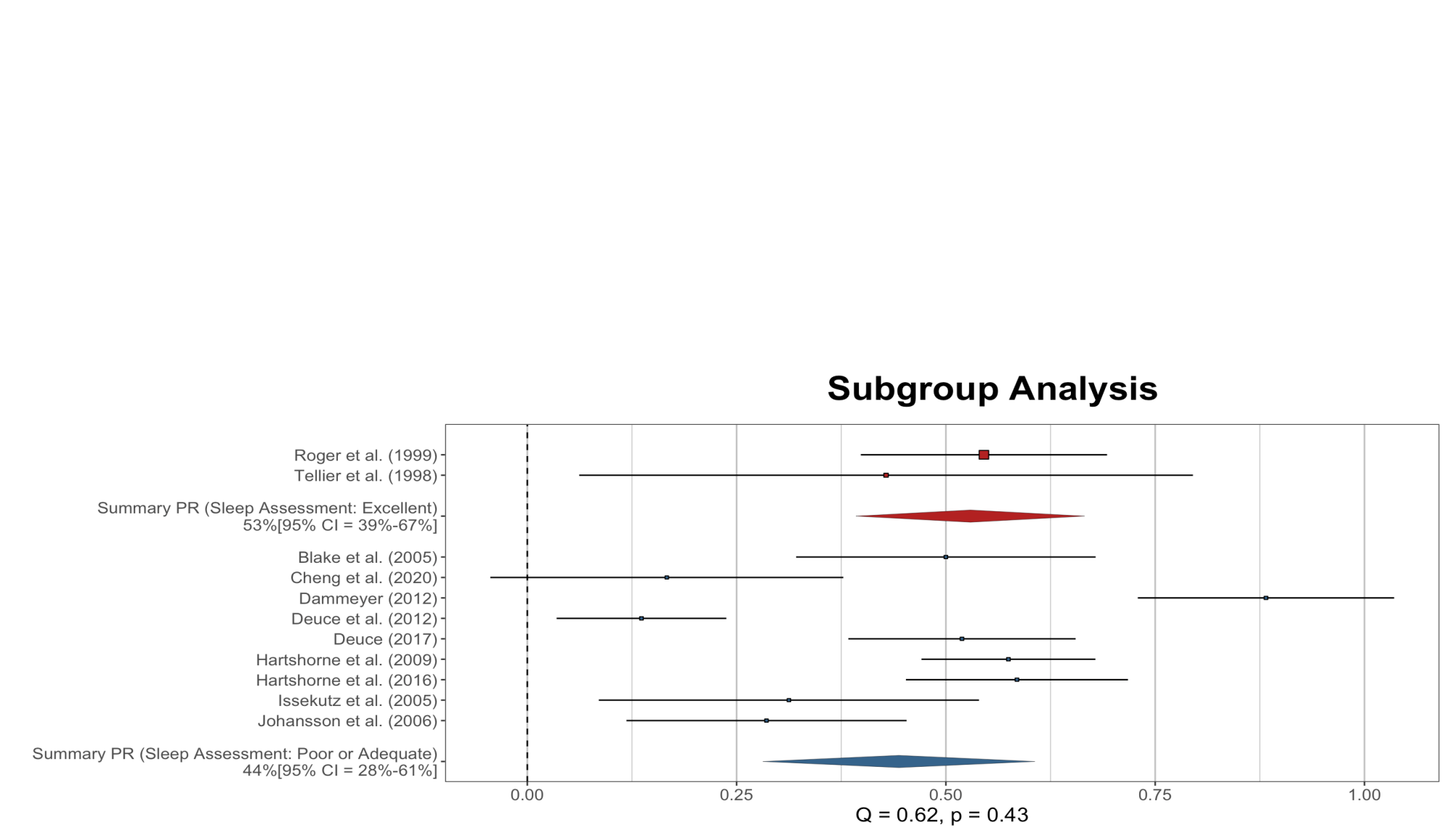


**Note:** Subgroup analysis found no statistical difference (p = 0.43) between studies rated poor/adequate or excellent for method of sleep assessment. Subgroup analysis was evaluated by comparison of 95% CIs.

**References:**

1. Pinchefsky E, Shevell M. Intellectual Disabilities and Global Developmental Delay. In: Handbook of DSM-5 Disorders in Children and Adolescents. 2017. p. 19–55. doi:10.1007/978-3-319-57196-6_2.

2. Blake KD, Salem-Hartshorne N, Daoud MA, Gradstein J. Adolescent and adult issues in CHARGE syndrome. Clin Pediatr (Phila). 2005;44:151–9. doi:http://dx.doi.org/10.1177/000992280504400207.

3. Hale CL, Niederriter AN, Green GE, Martin DM. Atypical phenotypes associated with pathogenic CHD7 variants and a proposal for broadening CHARGE syndrome clinical diagnostic criteria. Am J Med Genet Part A. 2016;170:344–54.

4. Hsu P, Ma A, Barnes EH, Wilson M, Hoefsloot LH, Rinne T, et al. The Immune Phenotype of Patients with CHARGE Syndrome. J Allergy Clin Immunol Pract. 2016;4:96. doi:http://dx.doi.org/10.1016/j.jaip.2015.09.004.

5. Shiohama T, McDavid J, Levman J, Takahashi E. Quantitative brain morphological analysis in CHARGE syndrome. NeuroImage Clin. 2019;23:101866.

6. Smith IM, Nichols SL, Issekutz K, Blake K. Behavioral profiles and symptoms of autism in CHARGE syndrome: preliminary Canadian epidemiological data. Am J Med Genet. 2005;133A:248–56. doi:http://dx.doi.org/10.1002/ajmg.a.30544.

7. Tellier AL, Cormier-Daire V, Abadie V, Amiel J, Sigaudy S, Bonnet D, et al. CHARGE syndrome: Report of 47 cases and review. Am J Med Genet. 1998;76:402–9. doi:http://dx.doi.org/10.1002/%28SICI%291096-8628%2819980413%2976:5%3C402::AID-AJMG7%3E3.0.CO;2-O.

8. Wessels K, Bohnhorst B, Luhmer I, Morlot S, Bohring A, Jonasson J, et al. Novel CHD7 mutations contributing to the mutation spectrum in patients with CHARGE syndrome. Eur J Med Genet. 2010;53:280–5. doi:10.1016/j.ejmg.2010.07.002.

9. Blake KD, Russell-Eggitt IM, Morgan DW, Ratcliffe JM, Wyse RK. Who’s in CHARGE? Multidisciplinary management of patients with CHARGE association. Arch Dis Child. 1990;65:217–23. http://ovidsp.ovid.com/ovidweb.cgi?T=JS&PAGE=reference&D=emed4&NEWS=N&AN=20057815.

10. Harvey AS, Leaper PM, Bankier A. CHARGE association: Clinical manifestations and developmental outcome. Am J Med Genet. 1991;39:48–55. http://ovidsp.ovid.com/ovidweb.cgi?T=JS&PAGE=reference&D=med3&NEWS=N&AN=1867265.

11. Johansson M, Rastam M, Billstedt E, Danielsson S, Strömland K, Miller M, et al. Autism spectrum disorders and underlying brain pathology in CHARGE association. Dev Med Child Neurol. 2006;48:40–50. doi:http://dx.doi.org/10.1017/S0012162206000090.

12. Oley CA, Baraitser M, Grant DB. A reappraisal of the CHARGE association. J Med Genet. 1988;25:147–56. http://ovidsp.ovid.com/ovidweb.cgi?T=JS&PAGE=reference&D=emed4&NEWS=N&AN=18082760.

13. American Psychiatric Association. Diagnostic and Statistical Mental Disorders DSM-5. 5th edition. Arlington, VA: American Psychiatric Publishing; 2013.

14. Asher BF, McGill TJ, Kaplan L, Friedman EM, Healy GB. Airway complications in CHARGE association. Arch Otolaryngol - Head Neck Surg. 1990;116:15. http://ovidsp.ovid.com/ovidweb.cgi?T=JS&PAGE=reference&D=emed4&NEWS=N&AN=20045463.

15. Bernstein V, Denno LS. Repetitive behaviors in CHARGE syndrome: differential diagnosis and treatment options. Am J Med Genet. 2005;133A:232–9. doi:http://dx.doi.org/10.1002/ajmg.a.30542.

16. Jongmans MCJ, Admiraal RJ, van der Donk KP, Vissers LELM, Baas AF, Kapusta L, et al. CHARGE syndrome: the phenotypic spectrum of mutations in the CHD7 gene. J Med Genet. 2006;43:306–14. doi:10.1136/jmg.2005.036061.

17. Shoji Y, Ida S, Etani Y, Yamada H, Kayatani F, Suzuki Y, et al. Endocrinological characteristics of 25 Japanese patients with CHARGE syndrome. Clin Pediatr Endocrinol. 2014;23:45–51. doi:http://dx.doi.org/10.1297/cpe.23.45.

18. Legendre M, Abadie V, Attie-Bitach T, Philip N, Busa T, Bonneau D, et al. Phenotype and genotype analysis of a French cohort of 119 patients with CHARGE syndrome. Am J Med Genet Part C Semin Med Genet. 2017;175:417–30. doi:https://dx.doi.org/10.1002/ajmg.c.31591.

19. Blake KD, Brown D. CHARGE association looking at the future--the voice of a family support group. Child Care Health Dev. 1993;19:395–409. http://ovidsp.ovid.com/ovidweb.cgi?T=JS&PAGE=reference&D=emed5&NEWS=N&AN=127249873.

20. Deuce G. The education of learners with CHARGE syndrome. Br J Spec Educ. 2017;44:376–93. doi:http://dx.doi.org/10.1111/1467-8578.12183.

21. Raqbi F, Le Bihan C, Morrisseau-Durand MP, Dureau P, Lyonnet S, Abadie V. Early prognostic factors for intellectual outcome in CHARGE syndrome. Dev Med Child Neurol. 2003;45:483–8. doi:10.1017/S0012162203000896.

22. Hittner HM, Hirsch NJ, Kreh GM, Rudolph AJ. Colobomatous microphthalmia, heart disease, hearing loss, and mental retardation-a syndrome. J Pediatr Ophthalmol Strabismus. 1979;16:122–8. https://search.proquest.com/docview/1490658587?pq-origsite=gscholar.

23. Dammeyer J. Development and characteristics of children with Usher syndrome and CHARGE syndrome. Int J Pediatr Otorhinolaryngol. 2012;76:1292–6. doi:http://dx.doi.org/10.1016/j.ijporl.2012.05.021.

24. Davenport SLH, Hefner MA, Mitchell JA. The spectrum of clinical features in CHARGE syndrome. Clin Genet. 1986;29:298–310.

25. Lasserre E, Vaivre-Douret L, Abadie V. Psychomotor and cognitive impairments of children with CHARGE syndrome: common and variable features. Child Neuropsychol. 2013;19:449–65. doi:https://dx.doi.org/10.1080/09297049.2012.690372.

26. Sohn YB, Ko JM, Shin CH, Yang SW, Chae J-H, Lee K-A. Cerebellar vermis hypoplasia in CHARGE syndrome: Clinical and molecular characterization of 18 unrelated Korean patients. J Hum Genet. 2016;61:235–9. doi:http://dx.doi.org/10.1038/jhg.2015.135.

27. Abadie V, Hamiaux P, Ragot S, Legendre M, Malecot G, Burtin A, et al. Should autism spectrum disorder be considered part of CHARGE syndrome? A cross-sectional study of 46 patients. Orphanet J Rare Dis. 2020;15. doi:10.1186/s13023-020-01421-9.

28. Salem-Hartshorne N, Jacob S. Chracteristics and development of children with CHARGE association/syndrome. J Early Interv. 2004;26:292–301.

29. Wulffaert J, Scholte EM, Dijkxhoorn YM, Bergman JEH, van Ravenswaaij-Arts CMA, van Berckelaer-Onnes IA. Parenting stress in CHARGE syndrome and the relationship with child characteristics. J Dev Phys Disabil. 2009.

30. Hartshorne N, Hudson A, MacCuspie J, Kennert B, Nacarato T, Hartshorne T, et al. Quality of life in adolescents and adults with CHARGE syndrome. Am J Med Genet Part A. 2016;170:2012–21. doi:http://dx.doi.org/10.1002/ajmg.a.37769.

31. Deuce G, Howard S, Rose S, Fuggle C. A study of CHARGE Syndrome in the UK. Br J Vis Impair. 2012;30:91–100. doi:http://dx.doi.org/10.1177/0264619612443883.

32. Hartshorne TS, Cypher AD. Challenging behavior in CHARGE Syndrome. Ment Heal Asp Dev Disabil. 2004;7:41–52. http://ovidsp.ovid.com/ovidweb.cgi?T=JS&PAGE=reference&D=emed8&NEWS=N&AN=38738142.

33. Souriau J, Gimenes M, Blouin C, Benbrik I, Benbrik E, Churakowskyi A, et al. CHARGE syndrome: Developmental and behavioral data. Am J Med Genet. 2005;133 A:278–81. doi:http://dx.doi.org/10.1002/ajmg.a.30549.

34. Hartshorne TS, Heussler HS, Dailor AN, Williams GL, Papadopoulos D. Sleep disturbances in CHARGE syndrome: Types and relationships with behavior and caregiver well-being. Dev Med Child Neurol. 2009;51:143–50. doi:http://dx.doi.org/10.1111/j.1469-8749.2008.03146.x.

35. World Health Organization. ICD-11: International Classification of Diseases 11th Revision. [https://icd.who.int/en. Accessed 1 Dec 2021](https://icd.who.int/en.%20Accessed%201%20Dec%202021).

# **Appendix 11.** Operational Definitions for Behavioural, Psychological, Cognitive and Sleep Characteristics in CHARGE Syndrome

##### **Table A11.1** Operational Definitions Used to Identify Behavioural, Psychological, Cognitive and Sleep Characteristics Reported in the Literature

| **Characteristic** | **Definition** |
| --- | --- |
| **Developmental delay** | Early onset (<5 years) delay or deficit in two or more areas of functioning: cognitive skills, speech development, motor skills or social and emotional development [1] |
| **Intellectual disability** | IQ: <69; Daily skills: Daily skills: Require at least minimum support for independent living (DSM-IV; DSM-5) |
| **Mild or moderate intellectual disability** | IQ: 36-69; Daily skills: Independent living with minimum or moderate level of support |
| **Severe or profound intellectual disability** | IQ: <35; Daily living: Requires daily assistance with self-care activities and safety supervision or requires 25-hour care |
| **Autism** | A clinical diagnosis of Autism, inclusive of all previous terms |
| **Aggression** | Outward physical or verbal aggression |
| **Self-injurious behaviour** | Self-directed behaviors that could cause physical injury |

**References:**

1. Pinchefsky E, Shevell M. Intellectual Disabilities and Global Developmental Delay. In: Handbook of DSM-5 Disorders in Children and Adolescents. 2017. p. 19–55. doi:10.1007/978-3-319-57196-6_2.

# **Appendix 12.** Meta-Regression Estimates of The Effects of Co-Occurring Characteristics on Behavioural, Psychological, Cognitive and Sleep Characteristics in CHARGE Syndrome. Statistical Significance Evaluated Using Benjamini-Hochberg Adjustment for Multiple Comparisons

##### **Table A12.1** Meta-Regression Estimates for of the Effects of Co-Occurring Characteristics on Behavioural, Psychological, Cognitive and Sleep Characteristics in the Eligible CHARGE Syndrome Literature

| Moderator variable | | N | k | β | Z | SE | p | R^2^ | Adj sig |  |
| --- | --- | --- | --- | --- | --- | --- | --- | --- | --- | --- |
| Developmental Delay | | | | | | | | | |  |
|  | Year | 212 | 7 | .005 | 1.24 | .004 | .213 | 18% | 0.509 |  |
| Intellectual Disability | | | | | | | | | |  |
|  | Year | 515 | 16 | .005 | .926 | .006 | .354 | 0% | 0.721 |  |
|  | Mean Age | 300 | 10 | .002 | .045 | .034 | .964 | 0% | 0.964 |  |
|  | Male | 401 | 12 | .199 | .484 | .411 | .628 | 0% | 0.886 |  |
|  | Coloboma | 295 | 10 | -.761 | -1.18 | .645 | .238 | 9% | 0.545 |  |
| p = 0.005 | Choanal Atresia | 299 | 8 | -.96 | -4.61 | .208 | <.001 | 91% | 0.014 |  |
|  | CN-VII | 184 | 11 | -.393 | -1.26 | .312 | .208 | 0% | 0.509 |  |
|  | CN-IX / X | 86 | 6 | -.117 | -.2 | .593 | .843 | 0% | 0.963 |  |
| p = 0.0167 | Inner Ear | 194 | 5 | -1.15 | -4.48 | .257 | <.001 | 100% | 0.014 |  |
|  | Heart Defect | 360 | 13 | -.073 | -.113 | .642 | .91 | 0% | 0.963 |  |
|  | Growth Deficiency | 351 | 12 | -.958 | -2.51 | .381 | .012 | 53% | 0.073 |  |
| p = 0.059 | Genital Hypoplasia | 269 | 7 | 1.282 | 2.79 | .46 | .005 | 70% | 0.034 |  |
|  | Cleft Lip / Palate | 273 | 7 | -.755 | -1.54 | .489 | .123 | 8% | 0.397 |  |
| p = 0.825 | Urinary Tract Anomaly | 171 | 6 | -1.76 | -2.87 | .612 | .004 | 80% | 0.031 |  |
|  | Skeletal Anomalies | 134 | 7 | .272 | .712 | .382 | .477 | 0% | 0.846 |  |
|  | Hearing Loss (HL) | 372 | 11 | 1.804 | 2.05 | .88 | .04 | 38% | 0.169 |  |
|  | Severe or Profound HL | 194 | 7 | .226 | .53 | .428 | .597 | 21% | 0.884 |  |
| Mild or Moderate ID | | | | | | | | | |  |
|  | Year | 217 | 10 | .012 | 1.666 | .007 | .096 | 11% | 0.374 |  |
|  | Male | 154 | 7 | .596 | 1.486 | .401 | .137 | 7% | 0.397 |  |
|  | Hearing Loss | 74 | 5 | 3.024 | 1.63 | 1.847 | .102 | 30% | 0.374 |  |
| Severe/Profound ID | | | | | | | | | |  |
|  | Year | 249 | 11 | -.005 | -1.434 | .004 | .152 | 8% | 0.418 |  |
|  | Male | 156 | 7 | -0.841 | -2.049 | .41 | .040 | 0% | 0.169 |  |
|  | Hearing Loss | 93 | 6 | 1.08 | .817 | 1.322 | .414 | 0% | 0.808 |  |
| Autism Spectrum Disorder | | | | | | | | | | |
|  | Year | 433 | 8 | .011 | 1.311 | .008 | .19 | 35% | 0.498 |  |
|  | Age | 280 | 6 | -.014 | -.621 | .023 | .535 | 0% | 0.865 |  |
|  | Male | 433 | 8 | .103 | .185 | .554 | .853 | 0% | 0.963 |  |
|  | Coloboma | 387 | 7 | .134 | .165 | .812 | .869 | 0% | 0.963 |  |
|  | Choanal Atresia | 387 | 7 | .2 | .669 | .299 | .504 | 0% | 0.865 |  |
|  | CN-VII | 302 | 5 | .11 | .152 | .699 | .875 | 0% | 0.963 |  |
|  | Heart Defect | 387 | 7 | .238 | .449 | .53 | .654 | 0% | 0.899 |  |
|  | Growth Deficiency | 384 | 7 | -.398 | -.557 | .715 | .578 | 0% | 0.884 |  |
|  | Genital Hypoplasia | 333 | 6 | .264 | .796 | .331 | .426 | 22% | 0.808 |  |
|  | Cleft Lip / Palate | 387 | 7 | -.054 | -.122 | .44 | .903 | 0% | 0.963 |  |
|  | Tracheoesophageal fistula | 377 | 6 | .718 | .745 | .964 | .457 | 3% | 0.838 |  |
|  | Skeletal Anomalies | 377 | 6 | .114 | .362 | .315 | .717 | 10% | 0.939 |  |
|  | Gross Motor Difficulties | 300 | 5 | .104 | .398 | .263 | .691 | 12% | 0.927 |  |
| Aggression | | | | | | | | | | |
|  | Year | 303 | 6 | .005 | .511 | .011 | .609 | 0% | 0.884 |  |
|  | Male | 239 | 5 | .446 | .646 | .69 | .519 | 0% | 0.865 |  |
|  | Choanal Atresia | 239 | 5 | -.408 | -1.01 | .402 | .31 | 0% | 0.656 |  |
|  | Heart Defect | 239 | 5 | -.904 | -2.21 | .409 | .027 | 85% | 0.149 |  |
|  | Growth Deficiency | 239 | 5 | -.238 | -.51 | .469 | .611 | 0% | 0.884 |  |
|  | Cleft Lip / Palate | 239 | 5 | -.03 | -.06 | .5 | .953 | 0% | 0.964 |  |
| Obsessive or Compulsive Behaviour | | | | | | | | | |  |
|  | Year | 272 | 6 | -.002 | -.049 | .03 | .961 | 0% | 0.964 |  |
|  | Growth Deficiency | 243 | 5 | .581 | 1.12 | .581 | .263 | 0% | 0.579 |  |
|  | Hearing Loss | 242 | 5 | .263 | .224 | 1.173 | .823 | 0% | 0.963 |  |
| Sleep Problems | | | | | | | | | |  |
|  | Year | 390 | 11 | -.002 | -.201 | .011 | .841 | 0% | 0.963 |  |
|  | Male | 215 | 6 | -1.243 | -3.04 | .41 | .002 | 66% | 0.018 |  |
|  | Coloboma | 198 | 5 | 1.363 | 1.496 | .911 | .135 | 19% | 0.397 |  |
|  | Choanal Atresia | 198 | 5 | .616 | 3.05 | .202 | .002 | 89% | 0.018 |  |
|  | Heart Defect | 262 | 5 | .588 | 1.502 | .391 | .133 | 52% | 0.397 |  |
|  | Growth Deficiency | 238 | 6 | .678 | 6.67 | .102 | <.001 | 100% | 0.014 |  |
|  | Cleft Lip / Palate | 224 | 5 | .85 | 2.16 | .394 | .031 | 61% | 0.155 |  |
|  | Urinary Tract Anomaly | 186 | 5 | -.137 | -.136 | 1.01 | .892 | 0% | 0.963 |  |
|  | Gross Motor Difficulties | 184 | 5 | .663 | 3.41 | .195 | .0007 | 76% | 0.014 |  |
|  | Hearing Impairment | 264 | 6 | -.137 | -.136 | 1.01 | .892 | 0% | 0.963 |  |

**Note:** Each meta-regression was calculated separately using one moderator variable

# **Appendix 13.** Meta-Regression Estimates of Genotype-Phenotype Correlations in CHARGE Syndrome. Statistical Significance Evaluated Using Benjamini-Hochberg Adjustment for Multiple Comparisons

##### **Table A13.1** Meta-Regression Estimates of Genotype-Phenotype Correlations in the Eligible CHARGE Syndrome Literature

|  | Moderator variable | N | k | β | Z | SE | p | R^2^ | Adj sig |
| --- | --- | --- | --- | --- | --- | --- | --- | --- | --- |
| CHD7 | |  |  |  |  |  |  |  |  |
|  | Year | 357 | 10 | 0.013 | 1.00 | 0.013 | 0.317 | 0% | 1.000 |
|  | Male | 179 | 7 | -0.128 | -0.287 | 0.447 | 0.774 | 0% | 1.000 |
|  | Coloboma | 92 | 5 | 0.355 | 0.737 | 0.483 | 0.461 | 0% | 1.000 |
|  | Choanal Atresia | 93 | 5 | 0.173 | 0.384 | 0.451 | 0.701 | 0% | 1.000 |
|  | Inner Ear Anomaly | 128 | 5 | -0.007 | -0.02 | 0.335 | 0.984 | 0% | 1.000 |
| Truncating CHD7 | |  |  |  |  |  |  |  |  |
|  | Year | 305 | 10 | -0.001 | -0.001 | -0.111 | 0.994 | 0% | 1.000 |
|  | Male | 260 | 10 | -0.444 | -1.707 | 0.26 | 0.088 | 18% | 1.000 |
|  | Coloboma | 216 | 9 | -0.005 | -0.013 | 0.46 | 0.99 | 0% | 1.000 |
|  | Choanal Atresia | 244 | 9 | -0.07 | -0.233 | 0.299 | 0.816 | 0% | 1.000 |
|  | CNVII – Facial Palsy | 134 | 6 | 0.222 | 0.506 | 0.438 | 0.613 | 0% | 1.000 |
|  | External Ear | 107 | 6 | -0.002 | -0.005 | 0.351 | 0.996 | 0% | 1.000 |
|  | Inner Ear | 97 | 7 | -0.148 | -0.567 | 0.28 | 0.598 | 0% | 1.000 |
|  | Heart Defect | 239 | 9 | -0.993 | -2 | 0.496 | 0.045 | 30% | 0.945 |
|  | Growth Deficiency | 174 | 8 | -1.353 | -2.831 | 0.478 | 0.005 | 75% | 0.115 |
|  | Genital | 222 | 9 | -0.153 | -0.627 | 0.243 | 0.531 | 0% | 1.000 |
|  | Cleft | 217 | 8 | 0.215 | 0.493 | 0.436 | 0.622 | 0% | 1.000 |
|  | Tracheoesophageal Fistula | 171 | 5 | -0.29 | -0.117 | 2.48 | 0.907 | 0% | 1.000 |
|  | Urinary Tract Anomaly | 139 | 7 | -0.808 | -2.784 | 0.29 | 0.005 | 72% | 0.115 |
|  | Brain Anomaly | 119 | 6 | -0.318 | -1.426 | 0.223 | 0.154 | 0% | 1.000 |
|  | Skeletal Anomaly | 133 | 7 | -0.311 | -1.817 | 0.171 | 0.069 | 48% | 1.000 |
|  | Hearing Impairment | 210 | 8 | 1.01 | 1.977 | 0.509 | 0.048 | 0% | 0.960 |
|  | Intellectual Disability | 141 | 5 | -0.076 | -0.427 | 0.178 | 0.669 | 0% | 1.000 |
|  | Developmental Delay | 74 | 5 | 0.521 | 0.378 | 1.378 | 0.706 | 0% | 1.000 |

Each meta-regression was calculated separately using one moderator variable

# **Appendix 14.** Meta-Regression Estimates for Each Characteristic Identified to be Associated with CHARGE syndrome using Year of Publication as the Moderator Variable. Statistical Significance Evaluated Using Benjamini-Hochberg Adjustment for Multiple Comparisons

##### **Table A14.1** Meta-Regression Estimates for Each Characteristic Identified to be Associated with CHARGE syndrome using Year of Publication as the Moderator Variable

| **Moderator variable = Year** | **N** | **k** | **β** | **Z** | **SE** | **p** | **R^2^** | **Adj. sig** |
| --- | --- | --- | --- | --- | --- | --- | --- | --- |
| **Anosmia** | **271** | **11** | **0.011** | **1.103** | **0.01** | **0.27** | **17%** | **0.574** |
| **Atrial Septal Defects (permuted p-value = 0.002)** | **434** | **10** | **0.007** | **3.708** | **0.002** | **<0.001** | **87%** | **0.018** |
| **Bilateral Choanal Atresia** | **489** | **12** | **-0.014** | **-2.271** | **0.006** | **0.023** | **14%** | **0.207** |
| **Bilateral Coloboma** | **364** | **12** | **-0.006** | **-1.454** | **0.004** | **0.146** | **2%** | **0.404** |
| **Brain Anomaly** | **562** | **18** | **0.003** | **0.379** | **0.007** | **0.705** | **0%** | **0.868** |
| **Cerebellar Anomalies** | **301** | **11** | **0.004** | **1.079** | **0.004** | **0.28** | **11%** | **0.574** |
| **Characteristic Face** | **405** | **9** | **-0.002** | **-0.012** | **0.018** | **0.903** | **0%** | **0.96** |
| **Choanal Atresia** | **1216** | **30** | **-0.007** | **-2.433** | **0.003** | **0.015** | **25%** | **0.18** |
| **Cleft Lip / Palate** | **1176** | **28** | **<0.001** | **0.104** | **0.003** | **0.918** | **0%** | **0.96** |
| **Coloboma** | **1212** | **31** | **-0.003** | **-1.59** | **0.002** | **0.112** | **2%** | **0.404** |
| **Corpus Callosum** | **321** | **10** | **0.002** | **1.133** | **0.002** | **0.257** | **1%** | **0.574** |
| **External Ear Anomalies** | **435** | **14** | **-0.001** | **-0.354** | **0.005** | **0.723** | **0%** | **0.868** |
| **Facial Palsy** | **890** | **23** | **<0.001** | **0.057** | **0.004** | **0.954** | **0%** | **0.96** |
| **Feeding and Swallowing** | **817** | **21** | **0.002** | **0.618** | **0.003** | **0.536** | **0%** | **0.772** |
| **Gastrointestinal Reflux** | **318** | **8** | **-0.007** | **-0.724** | **0.009** | **0.469** | **0%** | **0.765** |
| **Genital Hypoplasia** | **1011** | **26** | **-0.003** | **-0.693** | **0.005** | **0.489** | **7%** | **0.765** |
| **Gross Motor Difficulties** | **438** | **10** | **-0.011** | **-0.773** | **0.014** | **0.439** | **0%** | **0.765** |
| **Growth Deficiency** | **1059** | **29** | **-0.006** | **-1.618** | **0.004** | **0.106** | **14%** | **0.404** |
| **Hand Anomalies** | **461** | **12** | **0.001** | **0.199** | **0.003** | **0.842** | **0%** | **0.947** |
| **Hearing Impairment** | **1070** | **25** | **0.004** | **2.183** | **0.002** | **0.029** | **10%** | **0.209** |
| **Heart Defects** | **1221** | **30** | **-0.004** | **-1.536** | **0.002** | **0.127** | **17%** | **0.404** |
| **Inner Ear Anomalies (permuted p-value = 0.005)** | **458** | **13** | **0.013** | **3.831** | **0.003** | **<0.001** | **70%** | **0.018** |
| **Iris Coloboma** | **354** | **10** | **-0.006** | **-1.561** | **0.004** | **0.118** | **0%** | **0.404** |
| **Laryngeal Anomalies** | **200** | **7** | **0.002** | **0.218** | **0.009** | **0.827** | **0%** | **0.947** |
| **Microcephaly** | **294** | **7** | **0.003** | **1.688** | **0.002** | **0.091** | **100%** | **0.404** |
| **Micrognathia** | **124** | **5** | **-0.009** | **-0.536** | **0.017** | **0.592** | **0%** | **0.789** |
| **Middle Ear Anomalies** | **273** | **7** | **-0.006** | **-0.716** | **0.008** | **0.474** | **0%** | **0.765** |
| **Otitis Media** | **311** | **8** | **-0.003** | **-0.63** | **0.004** | **0.528** | **0%** | **0.772** |
| **Patent Ductus Arteriosus** | **470** | **12** | **0.006** | **1.462** | **0.004** | **0.144** | **0%** | **0.404** |
| **Posterior Segment Coloboma** | **442** | **11** | **-0.002** | **-0.472** | **0.003** | **0.637** | **0%** | **0.819** |
| **Urinary Tract Anomalies** | **897** | **24** | **-0.002** | **-0.708** | **0.002** | **0.479** | **0%** | **0.765** |
| **Skeletal Anomalies** | **1002** | **25** | **0.005** | **1.334** | **0.004** | **0.182** | **1%** | **0.468** |
| **Severe Profound Hearing Im.** | **687** | **19** | **0.002** | **0.051** | **0.005** | **0.96** | **0%** | **0.96** |
| **Scoliosis** | **678** | **15** | **0.009** | **1.745** | **0.005** | **0.081** | **0%** | **0.404** |
| **Tracheoesophageal Fistula** | **983** | **21** | **0.002** | **1.066** | **0.002** | **0.287** | **1%** | **0.574** |
| **Ventricular Septal Defect** | **422** | **11** | **-0.002** | **-0.55** | **0.003** | **0.582** | **0%** | **0.789** |

Each meta-regression was calculated separately using one moderator variable
